# Gene expression studies for the analysis of domoic acid production in the marine diatom *Pseudo-nitzschia multiseries*

**DOI:** 10.1186/1471-2199-14-25

**Published:** 2013-11-01

**Authors:** Katie Rose Boissonneault, Brooks M Henningsen, Stephen S Bates, Deborah L Robertson, Sean Milton, Jerry Pelletier, Deborah A Hogan, David E Housman

**Affiliations:** 1 Department of Biological Sciences, Plymouth State University, MSC 64, 17 High St., Plymouth, NH 03264, USA; 2 Koch Institute, Massachusetts Institute of Technology, 76-553, 77 Massachusetts Avenue, Cambridge, MA 02139, USA; 3 Present address: Mascoma Corporation, 67 Etna Road Suite 300, Lebanon, NH 03766, USA; 4 Fisheries and Oceans Canada, Gulf Fisheries Centre, P.O. Box 5030, Moncton, New Brunswick E1C 9B6, Canada; 5 Biology Department, Clark University, 950 Main Street, Worcester, MA 01610, USA; 6 Present address: Vertex Pharmaceuticals, 130 Waverly Street, Cambridge, MA 02139, USA; 7 Department of Biochemistry, McGill University, 3655 Promenade Sir William Osler, Montreal, Quebec H3G 1Y6, Canada; 8 Department of Microbiology and Immunology, Vail Building Room 208, Dartmouth Medical School, Hanover, NH 03755, USA

**Keywords:** Gene expression, Gene regulation, cDNA microarray, RT-qPCR, Normalization, Reference gene, Domoic acid, *Pseudo-nitzschia multiseries*, Bacillariophyceae, Diatom

## Abstract

**Background:**

*Pseudo-nitzschia multiseries* Hasle (Hasle) (*Ps-n*) is distinctive among the ecologically important marine diatoms because it produces the neurotoxin domoic acid. Although the biology of *Ps-n* has been investigated intensely, the characterization of the genes and biochemical pathways leading to domoic acid biosynthesis has been limited. To identify transcripts whose levels correlate with domoic acid production, we analyzed *Ps-n* under conditions of high and low domoic acid production by cDNA microarray technology and reverse-transcription quantitative PCR (RT-qPCR) methods. Our goals included identifying and validating robust reference genes for *Ps-n* RNA expression analysis under these conditions.

**Results:**

Through microarray analysis of exponential- and stationary-phase cultures with low and high domoic acid production, respectively, we identified candidate reference genes whose transcripts did not vary across conditions. We tested eleven potential reference genes for stability using RT-qPCR and GeNorm analyses. Our results indicated that transcripts encoding JmjC, dynein, and histone H3 proteins were the most suitable for normalization of expression data under conditions of silicon-limitation, in late-exponential through stationary phase. The microarray studies identified a number of genes that were up- and down-regulated under toxin-producing conditions. RT-qPCR analysis, using the validated controls, confirmed the up-regulation of transcripts predicted to encode a cycloisomerase, an SLC6 transporter, phosphoenolpyruvate carboxykinase, glutamate dehydrogenase, a small heat shock protein, and an aldo-keto reductase, as well as the down-regulation of a transcript encoding a fucoxanthin-chlorophyll a-c binding protein, under these conditions.

**Conclusion:**

Our results provide a strong basis for further studies of RNA expression levels in *Ps-n*, which will contribute to our understanding of genes involved in the production and release of domoic acid, an important neurotoxin that affects human health as well as ecosystem function.

## Background

The marine diatom *Pseudo-nitzschia multiseries* Hasle (Hasle) (*Ps-n*) produces the neurotoxin domoic acid (DA), which causes amnesic shellfish poisoning (ASP) [1-4]. DA is a neuroexcitatory, water-soluble amino acid that exhibits structural similarity with the neurotransmitter glutamate [5]. DA binds with high affinity to glutamate receptors, leading to excitation and ultimately cell death of neurons exposed to this toxin [6]. Production of DA by *Ps-n*, and at least 14 other members of the genus *Pseudo-nitzschia*, has been verified in oceanic regions throughout the world, primarily in coastal and upwelling zones [7,8]. The documented effects of DA on humans, birds, finfish, cephalopods, and marine mammals, and the economic costs of shellfishery closures due to DA contamination, has generated ongoing interest in understanding the regulation and control of DA production in this genus [7,9-11]. Yet, the biosynthetic pathways leading to DA production and the genes that govern these pathways remain unresolved [12,13].

Numerous studies on *Ps-n* growth dynamics have shown that DA production does not begin until early stationary phase, i.e. toxin is not typically produced in detectable amounts during the exponential growth phase (reviewed in [9]). In other studies that exposed *Ps-n* to conditions that slowed cell division during the mid-exponential phase, cells produced low levels of toxin. Therefore, toxin production appears to be associated with stages in the cell cycle when cell division has slowed or stopped due to some limiting nutrient factor, most notably silicon (Si) [10,14]. In addition, several bacterial isolates have been shown to enhance DA production by *Ps-n*[15-17]. *Ps-n* can produce DA in axenic cultures [2,18], yet, reintroduction of bacteria to axenic cultures results in increased *Ps-n* DA production [15-17].

In this study, we developed a *Ps-n* cDNA library and used it to construct a microarray in order to screen for genes that were differentially expressed under high-toxin-producing versus low-toxin-producing conditions. A total of 5,265 *Ps-n* cDNAs were printed in replicate, and mRNAs from cells that were in late-exponential growth phase were compared to those that were in stationary phase in both axenic and non-axenic cultures. Using these array data, we identified candidate reference and target genes for further study. Eleven reference genes were evaluated for stability in reverse-transcription quantitative PCR (RT-qPCR) analyses of *Ps-n* mRNA from Si-limited cultures. We performed a GeNorm analysis to validate transcripts that did not vary across conditions. Using the validated reference transcripts, we then confirmed the differential regulation of several transcripts whose expression correlates with DA production. These findings will facilitate future work aimed at elucidating the DA biosynthesis pathway and identifying transcriptional biomarkers indicative of DA production.

## Results

### *Pseudo-nitzschia* growth and toxin production for microarray studies

**Figure 1:**
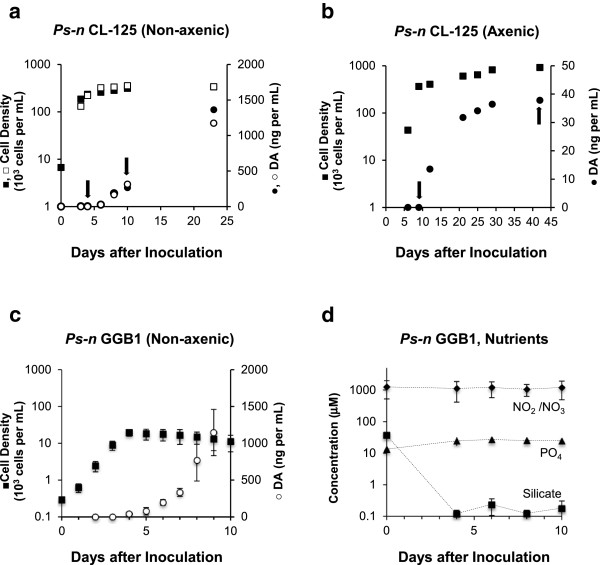
**Change in cell number and DA production as a consequence of growth under non-axenic and axenic conditions. a)***Ps-n* strain CL-125, Non-axenic culture experiments 1 (solid) and 2 (open). **b)***Ps-n* strain CL-125, Axenic culture experiment. Cells were harvested for RNA extraction on the days indicated by arrows. **c)** Increase in cell number (squares) and in DA concentration (circles) of *Ps-n* strain GGB1 in non-axenic, triplicate cultures. Cells were harvested for RNA extraction on days 3–10. **d)** Nutrient concentrations over time in GGB1 cultures (nitrite/nitrate, phosphate, silicate). Data for **(c)** and **(d)** represent the mean change of triplicate samples (± 1 SD).

Samples for microarray analysis were obtained from three biological experiments using *Ps-n* strain CL-125. These trials included one axenic and two non-axenic cultures, all grown in standard medium f/2. DA production began at the onset of stationary phase and continued to increase over time in all three experiments (Figure 1). Final DA concentrations, expressed on a per mL basis, were ~30 times lower in the axenic growth experiment compared to the non-axenic growth experiments, as expected based on previous studies [2,15-18]. Previous studies also indicated that Si is the limiting nutrient for *Ps-n* cells grown in batch cultures with medium f/2 [9,10,14]; therefore, we presume that the cells in these experiments were Si-limited during stationary phase. Samples were harvested for microarray analysis during the late-exponential and stationary phases to compare gene expression between low-toxin-producing vs. high-toxin-producing cells. These time points are indicated by arrows in Figure 1a, 1b.

### Identification and validation of reference transcripts

Our initial goal was the identification of transcripts whose expression levels were stable between late-exponential and stationary phases, which could then be used for normalization of other transcripts’ expression levels under these conditions. We selected eleven candidate reference genes to evaluate in RT-qPCR studies based on their stability in the microarray results as well as their biological roles and use as controls in previous studies (Table 1; Additional file 1). These included transcripts encoding: dynein, histone H3, cyclophilin, ubiquitin, elongation factor 1 alpha (EF-1α), phosphoglycerate kinase 1 (PGK), eukaryotic initiation factor 2 (eIF-2), a JmjC-domain containing protein (JmjC), an AAA-domain containing ATPase, glyceraldehyde-3-phosphate dehydrogenase (GAPDH), and 18s rRNA. RT-qPCR primer sets for each candidate reference gene were designed and tested, and exhibited high sequence specificity and PCR efficiency under our assay conditions with an annealing temperature of 60°C (Table 2).

**Table 1:** **Expression data for candidate reference genes from** ***Pseudo-nitzschia multiseries*** **(*Ps-n*) cDNA microarray analysis**^**a**^

		**Fold change**^**b**^			
		**Stationary versus late-exponential phase**			
***Ps-n*** **NR Identifier**	**JGI** ***Ps-n*** **Genome hit**	**Non-axenic**	**Non-axenic**	**Axenic**	**Predicted gene product**
	***Scaffold:Start-End***	**Expt. 1**	**Expt. 2**	**Expt.**	
53B6	*41:203449-205143*	1.05 ± 0.01	1.04 ± 0.05	1.05 ± 0.03	JmjC-domain family protein (JmjC)
45E3	*55:316954-329784*	1.10 ± 0.02	1.24 ± 0.07	1.36 ± 0.05	Dynein heavy chain, cytosolic
177F1	*198:180888-181958*	1.05 ± 0.02	0.93 ± 0.01	0.81 ± 0.01	Histone H3
PSN0918	*2485:4610-6293*	1.30 ± 0.00	1.29 ± 0.37	0.99 ± 0.01	Cyclophilin
PSN0001	*10:398258-400584*	1.37 ± 0.10	1.19 ± 0.07	1.10 ± 0.17	Elongation factor 1-alpha (EF-1α)
PSN0547	*210:148023-149837*	0.90 ± 0.08	0.78 ± 0.05	1.26 ± 0.03	Phosphoglycerate kinase (PGK)
PSN1327	*890:26709-27681*	1.00 ± 0.03	1.07 ± 0.00	1.24 ± 0.03	Elongation initiation factor 2 (eIF-2)
PSN0332	*2:525315-527491*	1.13 ± 0.05	1.29 ± 0.03	1.19 ± 0.17	ATPase with AAA domain
PSN0032	*18:343323-346000*	0.74 ± 0.04	0.68 ± 0.06	0.44 ± 0.03^c^	Ubiquitin
PSN1138	*68:114178-115516*	0.87 ± 0.08	0.92 ± 0.09	1.73 ± 0.23^c^	Glyceraldehyde-3-phosphate dehydrogenase (GAPDH)

^**a**^Potential reference genes for RT-qPCR studies were selected based on their stability in the microarray results or their use as controls in previous studies.

^b^The fold-change data presented in Table 1 represents the average differences across all of the cDNA clones that were printed on the array for each transcript. As follows, the number of independent cDNA clones for each transcript was: 53B6 (1), 45E3 (1), 177F1 (1), PSN0918 (1), PSN0001 (58), PSN0547 (2), PSN1327 (1), PSN0032 (7), PSN0332 (2), PSN1138 (6). Each clone was printed in duplicate on array. 18s not printed on array.

^c^Transcript levels showed statistically significant differences between samples.

**Table 2:** RT-qPCR reference gene primer sequences and characteristics for all candidate reference genes

**Predicted gene product**	**Primer sequence**	**GC (%)**	**Tm(°C)**^**a**^	**Amplicon (bp)**	**Ex-ExSpanning**^**b**^	**Efficiency (%)**	**R**^**2**^
JmjC	F: CCAGTTATGATTTCGGCAATAATGG	40.0	54.5	139	No	96.8	0.991
	R: GGTGTCAGTTCATCGTCTTCAG	50.0	55.4				
Dynein	F: CGAAGCCAGTAGTGGTATCAAGG	52.2	57.1	84	No	98.0	0.991
	R: CGAATCAGGTTGTTCTGGAGTCG	52.2	57.6				
Histone H3	F: GAAGCCTACCTGGTGGGTCTC	61.9	59.3	151	No	101.4	0.999
	R: CGTCCGATCACCTTCCGTCTC	61.9	59.5				
Cyclophilin	F: GTAGGACAAAGCCAGCACAACAGG	54.2	60.2	83	No	99.0	0.997
	R: GAATGAATCGGTGCTCGTAGGAGG	54.2	59.2				
Cyclophilin Ex-Ex	F: CTGGGTTTCAAGAGCCAACGAC	54.5	58.5	105	Yes	98.3	0.997
	R: CATCAATGCCGACGGACTGAAT	50.0	57.7				
EF-1á	F: GGACTCTCCATCAAGGGTATTGC	52.2	57.3	150	No	98.4	1.000
	R: GTATCCAGGCTTGAGGACACC	57.1	57.4				
PGK	F: GATGCCGAGAAGAAGGGTGTG	57.1	58.0	69	No	98.7	0.996
	R: CGAAGGAAATGCTTGTGTTGCGAC	50.0	59.2				
eIF-2	F: GTGATGCGTGCTTGATTGCTTG	50.0	57.6	78	No	99.6	0.997
	R: CCTTCATGTCGTGGCGAAGC	60.0	59.0				
ATPase^c^	F: GGTGGTGATATTGCTCCCTTG	52.4	55.7	164	No	98.4	0.996
	R: CGTTGATCTTCACTGATCTTTAGTCG	42.3	55.4				
Ubiquitin	F: CCTTCGTCGGAACATCACTACC	54.5	57.3	126	No	94.1	0.997
	R: CGTCAAGGGTGATAGTCTTGC	52.4	55.5				
GAPDH	F: GACAACTTCCACAAGGTCATCTCC	50.0	57.5	83	No	100.3	0.999
	R: CTGGTGTAGACAGCCAAGTCG	57.1	57.6				
18s rRNA	F: GTTGCCCGCCACTCTTTACGATTG	54.2	60.6	81	No	98.0	0.998
	R: GTATCAGTGCCAAGCCTCTGC	57.1	58.3				
β-tubulin Ex-Ex^d^	F: CCAAATTCTGGCAGGTCATG	50.0	54.2	114	Yes	100.8	0.998
	R: CTTGTCCCTCGTTGAAGTACAC	50.0	55.4				

^a^The annealing temperature for all standard curve analyses was performed at 60°C to demonstrate the efficiency of the primer sets under our assay conditions; the calculated Tm values are provided for reference.

^b^'Ex-Ex spanning’ refers to primers that span an exon-exon junction; these primer sets did not yield a product using gDNA as a template.

^c^The PSN0332 contig sequence had an extra “t” in the reverse primer region as compared to the *Pseudo-nitzschia multiseries* genome sequence, yet the primer set demonstrated high efficiency.

^d^β-tubulin was not used in the studies presented as it was not printed on the microarray. It is a validated exon-spanning primer set that demonstrated good efficiency, and may have value as a reference gene for future studies.

To validate the stability of candidate reference genes, biological triplicates of *Ps-n* strain GGB1 were grown under non-axenic, Si-limited conditions. RNA was harvested at multiple time points during the late-exponential and stationary phases (Figure 1c). The initiation of DA production again corresponded with the onset of stationary phase, which in this study was on day four. Initial silicate concentrations were reduced to 37.2 μM in the culture medium (vs. 107 μM in the standard f/2 medium). The measured silicate concentration was below 1.0 μM by day four (Figure 1d), corresponding with the entry into stationary phase. Nitrate and phosphate appear to be present in sufficient quantities throughout the experiment (Figure 1d) [9,10,14], further supporting that growth of these cultures was Si-limited.

**Figure 2:**
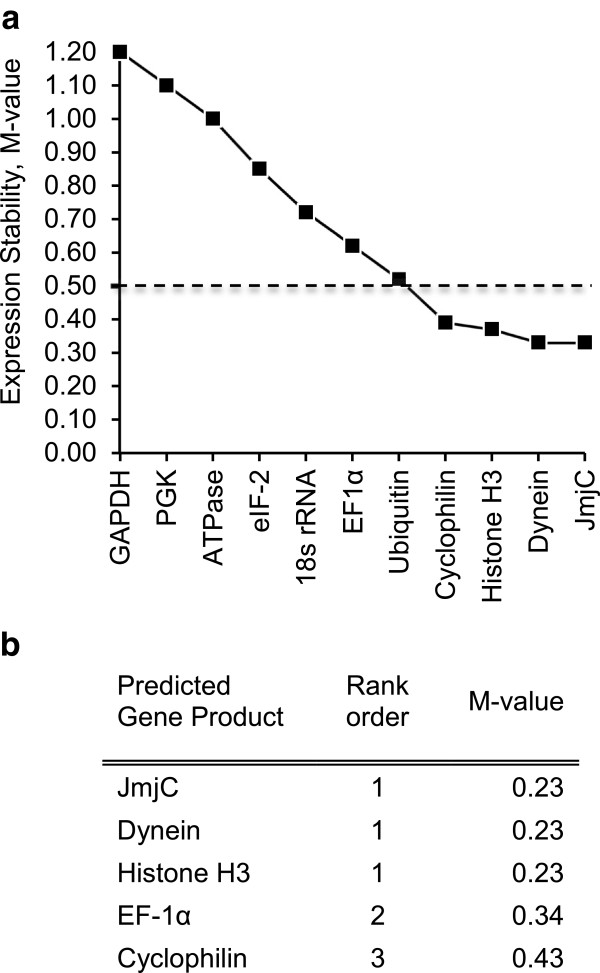
**Average expression stability (M-value) of the reference genes determined by GeNorm analysis.** An individual reference gene is tested against the other reference genes in a pairwise variation that serially excludes the least stable genes from the analysis. The most stable reference genes exhibit the lowest M-values. The accepted cut-off for stability of reference genes is an M-value of 0.50. **a)** In the initial analysis, which tested a subset of mRNA samples under Si-limited conditions for all eleven of our potential reference genes, four reference genes were determined to be acceptably stable. **b)** In the analysis of the Si-limited growth experiment, these four reference genes and EF1-alpha were tested. All five showed acceptable stability across the mRNAs, and JmjC, Dynein, and Histone H3 were tied for the 1st rank.

**Figure 3:**
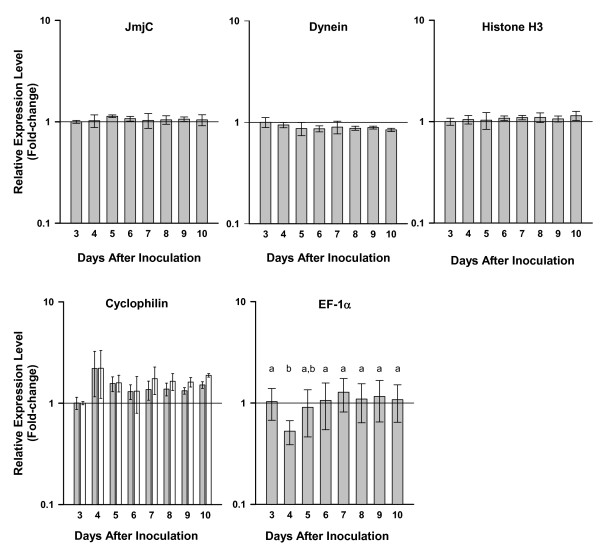
**RT-qPCR analysis of candidate reference genes for normalization from *Pseudo-nitzschia multiseries *.** Bars represent the mean change (± 1 SD) in expression relative to Day 3. Open bars represent measurements using primers that spanned an exon-exon junction; grey bars represent measurements using standard primers that did not span an exon-exon junction. Means were not significantly different (p < 0.05), except EF-1α. Means with different letters were significantly different (p < 0.05). Statistical analyses were performed using a general linear model ANOVA with Bonferroni *post-hoc* test, 95% confidence intervals.

In an initial GeNorm analysis, all eleven candidate genes were tested for stability across a subset of samples from the Si-limitation experiment, including replicates from days 3, 4, and 10 (Figure 2a). The four genes with the best stability values (M-value), i.e. JmjC (0.33), dynein (0.33), histone H3 (0.37) and cyclophilin (0.39), along with EF-1α (0.62), were further evaluated for stability using GeNorm analysis across the complete set of expression data for the Si-limitation experiment (Figure 2b). All five genes showed acceptable stability (M-value <0.5) when evaluated across the complete set of data. GeNorm pairwise-variation analysis determined that only two genes (JmjC, dynein) were necessary for subsequent normalization. However, since the JmjC, dynein, and histone H3 genes had equivalent M-values and were matched for 1st rank, we used all three for normalization of the expression data as described below. The expression profiles of the reference genes show the stability of these top-ranked genes, and the slight variability of the EF-1α and cyclophilin genes (Figure 3).

### Identification and verification of differentially expressed transcripts

In the microarray study, only those transcripts that were up- or down-regulated in all three trials were considered further (Tables 3 and 4; Additional file 1). Higher transcript levels in stationary (high-toxin-producing) as compared to late-exponential (low-toxin-producing) phase were observed for 12 transcripts, corresponding to 76 cDNA clones printed on the *Ps-n* array (Table 3; Additional file 1); reduced transcript levels under these conditions were observed for six genes, corresponding to 17 cDNA clones printed on the array (Table 4; Additional file 1). In addition to those genes identified based on the overall false discovery rate (FDR), we also included genes predicted to encode glutamate dehydrogenase and fucoxanthin-chlorophyll a-c binding protein (FCP) in our results, as both were of interest in this study, and the local false discovery rates (LFDR) indicated valid changes in gene expression. Fold-change differences were consistently lower in the axenic growth experiment; however, the patterns of expression were comparable across the three growth experiments.

**Table 3:** **Transcripts at higher levels in stationary (high-toxin-producing) as compared to late-exponential (low-toxin-producing) phase in** ***Pseudo-nitzschia multiseries*** **(*Ps-n*) as determined by cDNA microarray analysis**

		**Fold change**^**a**^			
		**Stationary versus late-exponential phase**			
***Ps-n *** **NR Identifier**	**JGI *Ps-n *** **Genome hit**	**Non-axenic**	**Non-axenic**	**Axenic**	**Predicted gene product**
	***Scaffold:Start-End***	**Expt. 1**	**Expt. 2**	**Expt.**	
PSN0011	*481:47438-49830*	3.99 ± 0.96	3.46 ± 1.90	2.19 ± 0.37	Cycloisomerase (pfam10282 lactonase/lactonizing enzyme),
					COG2706 (3-carboxymuconate cyclase)
PSN0072	*269:13929-16593*	3.40 ± 0.38	3.39 ± 0.32	2.01 ± 0.24	SLC6, Sodium and Chloride-dependent amino acid transporter
PSN0014	*2396:2127-4845*	4.64 ± 0.89	4.10 ± 0.89	2.14 ± 0.27	Acyl-CoA synthetase with transit peptide
PSN0016	*37:106113-109070*	3.78 ± 0.27	3.01 ± 0.29	3.11 ± 0.50	Phosphoenolpyruvate carboxykinase, ATP-
					dependent with transit peptide (PEPCK)
PSN0025	*155:75271-76268*	6.65 ± 1.56	7.07 ± 1.75	4.18 ± 0.57	Small heat shock protein with alpha-crystallin
					domain, chloroplastic (sHSP)
PSN0052	*70:256654-258177*	3.89 ± 0.59	2.61 ± 0.21	1.57 ± 0.01	Mitochondrial carrier protein
PSN0015	*66:296511-298386*	3.19 ± 0.57	3.15 ± 0.43	1.86 ± 0.17	Aldo-keto reductase with signal peptide
PSN0042	*21:457033-458813*	5.47 ± 0.75	7.52 ± 1.69	3.37 ± 0.50	Predicted protein with signal peptide
6H1	*117:15135-16102*	5.45 ± 0.55	3.76 ± 0.00	2.57 ± 0.04	Predicted protein with signal or transit peptide
73D12	*1312:10048-11278*	3.45 ± 0.11	4.01 ± 0.03	1.63 ± 0.07	*Ps-n* specific, no hits in NR or Swissprot
46A5	*447:56474-57752*	4.36 ± 0.00	4.61 ± 0.11	1.73 ± 0.02	*Ps-n* specific, no hits in NR or Swissprot
17F11	*303:41858-44529*	5.42 ± 0.18	6.02 ± 0.85	2.07 ± 0.06	Predicted protein with glycosyltransferase domain
PSN1428^b^	*95:293619-297233*	2.22 ± 0.26	1.86 ± 0.20	1.81 ± 0.37	NAD-specific glutamate dehydrogenase (GDH)

^a^The fold-change data presented are the average of all of the cDNA clones that were printed on the array for each transcript. The number of cDNA clones for each transcript was: PSN0011 (21), PSN0072 (3), PSN0014 (14), PSN0016 (14), PSN0025 (5), PSN0052 (4), PSN0015 (7), PSN0042 (4), 6H1 (1), 73D12 (1), 46A5 (1), 17F11 (1), PSN1428 (2). Each clone was printed on the array twice.

^b^PSN1428 was included as a gene of interest in RT-qPCR analysis, below, although it did not meet our statistical criteria for the original microarray analysis. Please see Methods for statistical analysis, and Additional file 1 for FDR and LDFR data.

**Table 4:** **Transcripts at lower levels in stationary (high-toxin-producing) as compared to late-exponential (low-toxin-producing) phase in** ***Pseudo-nitzschia multiseries*** **(*Ps-n*) as determined by cDNA microarray analysis**

		**Fold change**^**a**^			
		**Stationary versus late-exponential phase**			
***Ps-n *** **NR Identifier**	**JGI *Ps-n *** **Genome Hit**	**Non-axenic**	**Non-axenic**	**Axenic**	**Predicted gene product**
	***Scaffold:Start-End***	**Expt. 1**	**Expt. 2**	**Expt.**	
PSN0100	*32:397085-398987*	0.34 ± 0.01	0.33 ± 0.05	0.39 ± 0.01	Pyrophosphate-dependent phosphofructokinase (PFK)
PSN0060	*133:16659-18505*	0.20± 0.02	0.16 ± 0.03	0.44 ± 0.04	Predicted protein with signal or transit peptide
PSN0048	*188:179178-180986*	0.36± 0.08	0.25 ± 0.06	0.52 ± 0.05	Predicted protein with signal or transit peptide
PSN0080	*461:123066-124152*	0.32 ± 0.02	0.33 ± 0.03	0.57 ± 0.07	Predicted protein with mitochondrial transit peptide
135E4	*1441:17433-18891*	0.17 ± 0.00	0.19 ± 0.02	0.45 ± 0.02	Predicted protein
165G9	*8:175639-176910*	0.37 ± 0.02	0.37 ± 0.02	0.62 ± 0.04	Tetratricopeptide repeat protein
135H6^b^	*214:78592-7971*	0.41 ± 0.00	0.22 ± 0.02	0.59 ± 0.00	Fucoxanthin-chlorophyll a-c binding protein,
					chloroplastic (FCP)

^a^The fold-change data presented are the average of all of the cDNA clones that were printed on the array for each transcript. The number of cDNA clones for each transcript was: PSN0100 (2), PSN0060 (5), PSN0048 (5), PSN0080 (3), 135E4 (1), 165G9 (1), 135H6 (1), and each clone was printed twice.

^b^135H6 was included as a gene of interest in RT-qPCR analysis, below, although it did not meet our fold-change cut-off for the original FDR microarray analysis (yet, all LFDRs were <10%). Please see Methods for statistical analysis, and Additional file 1 for FDR and LDFR data.

**Figure 4:**
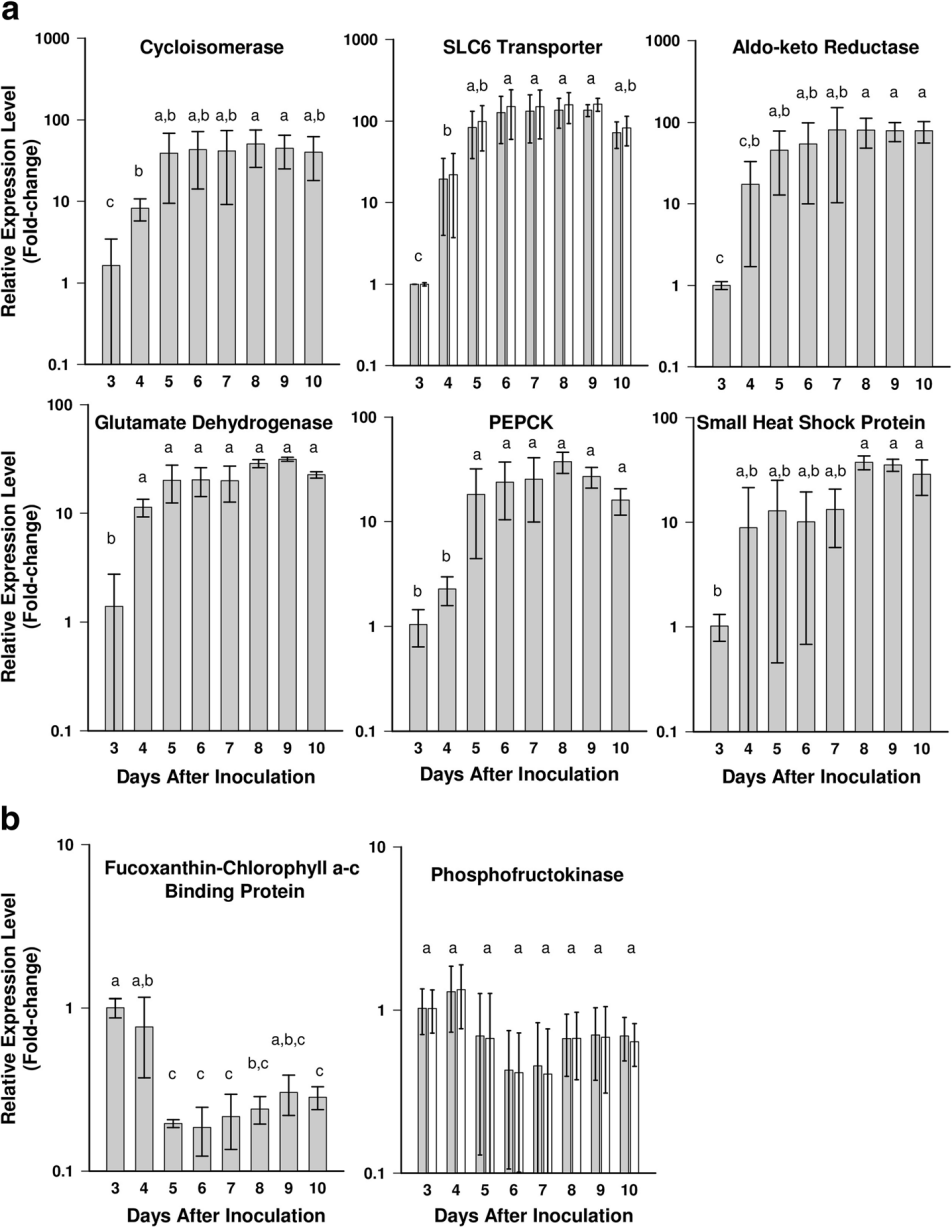
**RT-qPCR analysis of *Pseudo-nitzschia multiseries *genes whose expression was up-regulated (a) or down-regulated (b) in microarray analysis.** Bars represent the mean change (± 1 SD) in expression relative to Day 3. Open bars represent measurements using primers that spanned an exon-exon junction; grey bars represent measurements using standard primers that did not span an exon-exon junction. Means with different letters were significantly different (p < 0.05). Statistical analyses were performed using a general linear model ANOVA with Bonferroni *post-hoc* test, 95% confidence intervals.

Eight genes were selected for further study using RT-qPCR (Table 5). Of these, six genes had higher transcript levels during the stationary phase (Figure 4a), and were predicted to encode a cycloisomerase, an SLC6 transporter, an aldo-keto reductase, glutamate dehydrogenase, phosphoenolpyruvate carboxykinase (PEPCK), and a small heat shock protein. The cycloisomerase, SLC6 transporter, and aldo-keto reductase genes all had a statistically significant step-wise increase in transcript abundance from days 3 to 5 that correlated with the gradual increase in DA production (Figures 1c, and 4a). The other up-regulated genes showed similar trends during this time period. FCP showed decreased transcript levels during the transition from exponential to stationary phase; the phosphofructokinase (PFK) transcript levels, which were down-regulated in the microarray experiment, were not statistically different as measured by RT-qPCR (Figure 4b). Of note, the absence of DNA contamination in these studies is shown visually by the parallel results from amplification of cDNA using both standard and exon-exon spanning primer sets for the cyclophilin, SLC6 transporter, and PFK genes (Figures 3 and 4). Potential connections between these transcripts and DA production are discussed below.

**Table 5:** RT-qPCR target gene primer sequences and characteristics

**Predicted gene product**	**Primer sequence**	**GC (%)**	**Tm (°C)**^**a**^	**Amplicon (bp)**	**Ex-Ex Spanning**^**b**^	**Efficiency (%)**	**R**^**2**^
Cycloisomerase	F: TCATAGGTGGCGTCAAGAACGTGT	50.0	60.3	127	No	99.5	0.995
	R: TCAGCTTGTCGTGCCGAAATTGTG	50.0	60.3				
SLC6	F: TCGGACACTACGGAGACTACG	57.1	57.1	73	No	104.2	0.997
	R: ACCAAGGTGAAGGCGACG	61.1	58.0				
SLC6 Ex-Ex	F: CATGCACGATACTGTCTATTTCG	43.5	53.6	122	Yes	100.0	0.998
	R: CGTCCAACCAAAATAAGCCAGC	50.0	57.0				
Aldo-keto reductase	F: GAATGGGCTACGGAGAGACG	60.0	57.3	114	No	99.5	0.998
	R: GTACAGGCGTGAATTTGGTAGC	50.0	56.2				
sHSP	F: GACGAAGGATTCATCACCGTCG	54.5	57.7	141	No	102.9	0.998
	R: GACACCGTTGTCGAGGGTAG	60.0	57.4				
PEPCK	F: GCATTGCTCTGCAAACGTCG	55.0	57.7	107	No	100.0	0.997
	R: CAATCAAGGCTCGGTGAGGATC	54.5	57.7				
GDH	F: CAATGCCATCAACGCCATCAAGGA	50.0	60.2	128	No	98.4	0.998
	R: CAAAGCCGAGGTTGGCAAGAGTTT	50.0	60.3				
PFK	F: CGAGGTGGCATCCAAACGATTGC	56.5	61.1	84	No	105.1	0.998
	R: GCAGCCTGTGTATTGGTATCGTCG	54.1	59.7				
PFK Ex-Ex	F: GGAGAAAATCCGCTCGAGGTG	57.1	57.9	111	Yes	99.4	0.997
	R: CTTTGAGAGAACCGCAGCCTG	57.1	58.4				
FCP	F: CGTCTCATACCACGGCAC	61.1	55.7	184	No	97.8	0.997
	R: CTTGGATTGATGGTCCACGAG	52.3	55.6				

^a^The annealing temperature for all standard curve analyses was performed at 60°C to demonstrate the efficiency of the primer sets under our assay conditions; the calculated Tm values are provided for reference.

^b^'Ex-Ex spanning’ refers to primers that span an exon-exon junction; these primer sets did not yield a product using gDNA as a template.

## Discussion

Our data support the validity of the reference genes, JmjC, dynein, and histone H3, as suitable controls for normalization of *Ps-n* mRNAs under conditions of Si-limitation, as cells transition from late-exponential to stationary phase (i.e. from minimal to high DA production). Multiple reference genes are typically more effective for accurate normalization [19,20]; therefore, we recommend the use of the geometric mean of these three reference genes for normalization of *Ps-n* RT-qPCR expression data under these conditions. The stability of a histone H3 gene, whose expression often varies with growth phase in other species, may be attributed to regulation at the level of translation vs. transcription [21]. Alternatively, the presence of four histone H3 homologs in the *Ps-n* genome [22], as revealed by BLAST analysis, presents the possibility that we have identified a replication-independent family member, as shown in other studies [23-27]. Of note, our microarray data indicate that the standard “housekeeping” genes, GAPDH and actin, may not be suitable reference genes in this experimental system as their transcript levels varied (Table 1, Additional file 1). We tested GAPDH for stability in the RT-qPCR GeNorm analysis, and it showed the least stable M-value of the candidate reference genes tested. Similar results were observed in the diatom *Phaeodactylum tricornutum*[28]. These results highlight the importance of validating reference genes prior to use for normalization. The primer sets provided in Table 2 should prove useful for examining changes in *Ps-n* gene expression as populations transition from late-exponential to stationary phase, when DA production is stimulated, and for testing these candidate genes for use as controls under other growth conditions.

The overall correlation between the array and RT-qPCR data in these studies offers proof of concept for the reliability of these expression data. Also, the differential expression of these genes was evidenced in two different *Ps-n* strains, CL-125 and GGB1, which were isolated from Atlantic and Pacific coastal regions, respectively. The down-regulation of an FCP gene during stationary phase, when photosynthesis and chlorophyll presumably decline e.g. [29], also supports the validity of these results. The down-regulation of FCP gene expression has been correlated with stationary phase and decreases in light-harvesting pigments in the related pennate diatom *Phaeodactylum tricornutum*[30], as well as other marine algae [31,32]. The pathways leading to chlorophyll and DA production are both predicted to draw on a pool of glutamate [12,33], so the down-regulation of FCP in *Ps-n* correlates well with the onset of DA production.

Our results provide a framework to further investigate the control of toxin production in *Ps-n*. Previous ^13^C- and ^14^C-labeling studies suggested a model involving condensation of an activated glutamate intermediate derived from the citric acid cycle with an isoprenoid intermediate, and subsequent cyclization as a mechanism to generate DA [12]. These studies allow us to generate hypotheses regarding the biological function of the genes identified in our study relative to DA production. For example, up-regulation of a gene encoding a putative *Ps-n* cycloisomerase is intriguing as its product may be directly involved in the proposed cyclization step leading to the pyrrolidine ring in DA. Alternatively, the cycloisomerase, similar to other enzymes in the related pfam20282 group, may be involved in converting aromatic compounds into citric acid cycle intermediates, proposed to feed the pathway leading to DA synthesis [34-38]. The identification of a differentially expressed transcript encoding a member of the SLC6 amino acid transporter family is also interesting. The translated *Ps-n* open reading frame aligns most closely with characterized γ-aminobutyric (GABA) neurotransmitter transporters [39], suggesting the hypothesis that the *Ps-n* transporter is involved in movement of DA, or a synthetic precursor, into or out of cells.

Our discovery of the up-regulation of a predicted cycloisomerase belonging to the lactonase/lactonizing family, as well as the SLC6 transporter, entertains the speculation that these gene products are involved in communication between *Ps-n* cells, or *Ps-n* and bacteria. The parallels with GABA in plant signaling pathways [40-42] pose a potential role for DA in *Ps-n* biology, which has not yet been defined [7,9]. For example, GABA produced by wounded plant tissues appears to control the lactone quorum-sensing signal in *Agrobacterium tumefaciens* by regulating the *A. tumefaciens* lactonase gene [41]. Bacterial production of lactones in *Ps-n* cultures is correlated with increased DA production [16,17,43], suggesting a possible relationship between DA and quorum sensing [44]. Characterization of the predicted cycloisomerase’s enzymatic properties will be of significant interest in relation to these hypotheses. Similarly, demonstration that the SLC6 transporter is involved in movement of DA into or out of the cell would be a valuable contribution to understanding the role of DA in *Ps-n* biology. While we have taken the perspective that DA may function in signaling pathways, including quorum sensing or pheromone communication [7,8,42], some studies suggest that DA may function as a chelating agent [45-47]. Hence, studying the transport of DA into and out of *Ps-n* cells directly would contribute to describing the role(s) of DA in *Ps-n* biology. A family of four SLC6 transporters was identified in the recently released *Ps-n* draft genome [22,39], so characterization of this entire family should advance our understanding of *Ps-n* biology.

Many of the differentially expressed genes in this study relate to general metabolic pathways. Therefore, further investigation is needed to resolve the role of these genes in relation to both the growth state and DA synthesis in *Ps-n*. For example, the up-regulation of PEPCK, as well as the potential down-regulation of PFK, suggests a change in energy metabolizing pathways as *Ps-n* cells transition from exponential to stationary phase, consistent with a shift to gluconeogenesis and carbon metabolism through the citric acid cycle [48,49]. Similarly, glutamate dehydrogenase, which catalyzes the reversible conversion of glutamate and the citric acid cycle intermediate α-ketoglutarate, is a key enzyme involved in nitrogen and energy metabolism [50,51]. In addition, the differential expression of a predicted acyl-CoA synthetase (Table 3) suggests the possibility that lipids and fatty acids are being broken down, and while this may be a physiological response to growth-limiting conditions, the products could then be channeled as precursors into DA synthesis. Previous studies have shown that *Ps-n* lipid content decreases in response to Si deficiency during stationary phase [9,52]. Acyl-CoA synthetases are also involved in amino acid acylation, so could be directly involved in the condensation of the glutamate and isoprenoid-like moieties [13,53,54].

A small heat shock protein gene was most highly up-regulated later in the stationary phase as determined by RT-qPCR, suggestive of its expression relative to physiological stress. The aldo-keto reductase transcript levels showed a step-wise progression from the exponential into the stationary phase, with the highest expression levels later in stationary phase, as well. The expression patterns of these genes may be useful for monitoring the physiological state of *Ps-n* cells. The aldo-keto reductase may also have a functional role in DA synthesis, as the labeling studies indicated that the C7’ in DA is selectively oxidized to a carboxyl group [12]. Several of the genes that were identified as being up-regulated in this study have not been previously characterized from diatoms and represent potential targets for further studies of DA synthesis.

The enhancement of DA production by co-existing microbes is a complex and fascinating aspect of DA biology [7]. A limited number of genes in our study indicated significantly different expression patterns between the non-axenic vs. axenic growth experiments. For example, a subtilisin-like gene, predicted to encode a secreted protease, was up-regulated in the non-axenic cultures relative to the axenic culture (Additional file 2). In addition, microbes may influence the metabolic pathways predicted to be involved in fatty acid production. Ramsey et al. [12] suggested that the principal pathway to the isoprenoid portion of DA is via an alternative glyceraldehyde 3-phosphate (G3P)-independent route, and it is interesting to note that glyceraldehyde-3-phosphate dehydrogenase (GAPDH) was up-regulated only in the axenic growth experiment (Table 3), suggesting that bacteria may influence this pathway. Future studies will focus on the specific roles that co-existing microbes play in the regulation of *Ps-n* genes and domoic acid production.

The *Ps-n* genome is predicted to include 19,703 genes [22]; thus, the estimated 3,675 non-redundant transcripts monitored via this microarray represent ~20% of the genome. Future studies using RNA sequencing methods will determine if other transcripts related to those highlighted here are also differentially expressed in correlation with DA production.

## Conclusions

Our study identified a number of significantly up- and down-regulated genes that provide the basis for future studies on DA production, growth state, stress, and amino acid transport in *Ps-n*. The identified transcripts may be particularly useful as early indicators of toxin production and the switch of *Ps-n* cells to an alternative growth state. The reliability of RT-qPCR data will be enhanced by use of the validated internal reference genes presented in this study. To our knowledge, this is the first identification and validation of reference genes for RT-qPCR studies in *Ps-n*.

## Methods

### *Pseudo-nitzschia multiseries* strains and culture conditions

*Ps-n* strain CL-125 was isolated by Claude Léger (Fisheries and Oceans Canada, Gulf Fisheries Centre, Moncton, New Brunswick, Canada) from a sample collected on September 23, 2000, in Mill River (a brackish water estuary), Prince Edward Island, Canada. Cultures for the microarray studies were grown in 0.2 μm-filtered, autoclaved seawater (from Woods Hole, MA) enriched with f/2 nutrients [55] and amended with 10^-8^ μM Se. These batch cultures were grown in 15 L of f/2 medium in 19 L borosilicate carboys, and incubated at 20°C. The irradiance was maintained at 100 μmol photons m^-2^ s^-1^, with a 14:10-h light:dark (L:D) cycle for the cDNA library cultures, and continuous light for the experimental cultures. The cultures were aerated using aquarium pumps with sterile cotton and activated carbon filters and were constantly mixed with magnetic stirrers. An axenic culture of CL-125 was obtained by antibiotic treatment for 72 h, using 1.6:0.8 mg mL^-1^ penicillin:streptomycin [2]. These were tested for culturable bacteria by incubation in Bacto-peptone broth (Difco Laboratories, Detroit, MI, USA; 1 g L^-1^ seawater) and 2216 Marine Agar (Difco) at ~20°C for at least 20 d.

*Ps-n* strain GGB1 was isolated by Michael Carlson and Kyle Frishkorn (University of Washington, Seattle, WA, USA) in July 2010, from Puget Sound, WA, USA. Cultures for the RT-qPCR study were grown in 0.45 μm-filtered, autoclaved seawater (from Portsmouth Harbor, Newcastle, NH, USA) enriched with f/2 nutrients [55], except that the initial Si was lowered to 37.2 μM and ferric sequestrene was replaced with Na_2_EDTA • 2H_2_O and FeCl_3_ • 6H_2_O (Provasoli-Guillard National Center for Marine Algae and Microbiota). Before inoculation of experimental cultures, cells were maintained in exponential growth for at least two preceding transfers. Triplicate GGB1 experimental cultures were grown in 2.6 L of f/2 medium in 3-L polycarbonate baffled flasks, and incubated at 15°C. The irradiance was maintained at ~100 μmol photons m^-2^ s^-1^, with a 16:8-h L:D cycle. Flasks were aerated by constant mixing supplied by magnetic stirrers.

### Sampling, toxin, and nutrient analysis

The microarray study included three biological replicates: two non-axenic cultures and one axenic culture. Samples were taken every two to three days for cell counts and domoic acid (DA) analysis. Cell concentrations were estimated by averaging the number of cells enumerated by light microscopy, using a Neubauer hemacytometer chamber in triplicate counts of individual samples preserved in Lugol’s iodine. DA was analyzed in whole-culture samples (cells plus medium [1]), using HPLC of the FMOC (fluorenylmethoxycarbonyl) derivative [56]. The lower limit of detection was 15 ng mL^-1^ for the first non-axenic experiment, 3 ng mL^-1^ for the second non-axenic experiment, and 7.5 ng mL^-1^ for the axenic experiment. RNA was prepared from cells harvested during an initial time point from the late-exponential (low-toxin-producing) growth phase, and a final time point during the stationary (high-toxin-producing) phase (Figure 1a, 1b; RNA extraction protocol outlined below).

For RT-qPCR evaluation, three non-axenic biological replicate cultures were sampled daily, from the time of inoculation until day 10 of growth, for cell counts, whole-culture DA and nutrients. Cell count samples were taken by preserving 5 mL of culture with 250 μL of formalin and stored at 4°C until cells were counted (400 cells or the entire slide) on a Sedgwick-Rafter slide. Whole-culture DA samples were taken by freezing 15 mL of culture at -20°C; samples were sonicated at 50% power on ice for 2 min and filtered through a 0.2-μm filter prior to analysis. DA samples were analyzed using the Abraxis Domoic Acid ELISA kit ( [57], Warminster, PA, USA). The limit of detection was 0.06 ng mL^-1^. Filtered samples were stored at -80°C for nutrient analyses. Silicate was measured using the molybdate method [58-60]; phosphate was measured by the ascorbic acid-molybdate method [61,62]. Nitrite and nitrate were measured on an auto-analyzer (Lachat Instruments, Loveland, CO, USA) using a copper-cadmium reduction and colorimetric assay [61,63,64]. Total RNA was extracted daily from each flask beginning on day three of growth (Figures 1c, 3, 4; RNA extraction protocol outlined below). One RNA sample was lost on the initial day of extraction, so this resulted in two biological replicates for this time point (Day 3). The remaining RNA samples for the profile through to Day 10 included three biological replicates for each time point.

### Microarray and cDNA library construction

*Ps-n* strain CL-125 cells from non-axenic cultures were harvested during the late-exponential through mid-stationary phases, under predominantly toxin-producing conditions. Cultures were split into 250–500 mL aliquots that were centrifuged for 15 min at 1000 *g*. The loose pellets were pooled, and centrifuged again briefly to remove any residual culture medium. Total RNA was extracted immediately by homogenizing the cells in TRIzol® (Invitrogen Corporation, Carlsbad, CA, USA). Insoluble material was removed by low-speed centrifugation of the samples, which increased quality and yield of the resulting total RNA. Precipitating twice with salt and ethanol also contributed to high-quality total RNA, as indicated by both 260/280 O.D. ratios and gel electrophoresis. Poly (A)+ RNA was then isolated from total RNA using a biotin-labeled oligo(dT)20-streptavidin kit (Roche Molecular Biochemicals, Indianapolis, IN, USA) following the manufacturer’s instructions.

First-strand cDNA was prepared from 5 μg poly (A)+ RNA using Superscript II (Invitrogen, Grand Island, NY, USA), NC-p7 (an RNA chaperone), and oligo pd(TZ) (an oligo-dT primer with some of the internal thymidine residues replaced with 3-nitropyrrole to minimize mispriming to internal A-rich sequences). Double-stranded cDNA was generated using RNase H, *E. coli* DNA polymerase I, and *E. coli* ligase. The ends of the cDNAs were polished with T4 DNA polymerase, and BstXI adaptors were ligated to the cDNA ends. The cDNAs were then fractionated on sucrose gradients, ligated into pMD1 (a pUC-based vector) and transformed, by electroporation, into *E. coli* DH10B cells [65]. Following an initial library plating, 19,200 individual colonies were picked and stored at -80°C in 15% glycerol for further analysis.

### EST sequencing, assembly, and annotation

2,220 cDNA inserts were sequenced to verify the quality of the library and to begin gene discovery. Many of the cDNAs were sequenced more than once in the 5′- and 3′- direction, yielding a set of 3,533 *Ps-n* ESTs. These sequences are deposited in the NCBI dbEST database [GenBank accession numbers FD476666-FD480212]. (Note: Of the sequenced cDNAs, 1,889 were a subset of the 5,265 *Ps-n* cDNAs printed on the microarray (see below)). Sequence reactions were run on an automated DNA sequencer (ABI 3700 with dye terminators); and selected cDNAs were sequenced at ACGT, Inc. (Wheeling, IL, USA). ESTs were edited using Seqman (DNAStar, Inc., Madison, WI, USA), and ContigExpress (VectorNTI, Carlsbad, CA, USA), as well as manually edited to remove low quality data, poly (A) tails, and vector sequence. The *Ps-n* ESTs were assembled into consensus or contig sequences, using a criterion of 95% identity over more than 50 nucleotides (Seqman, DNAStar). Average sequence length of the individual reads was 639 bp (after editing). The ESTs were assembled into 1,550 non-redundant (NR) sequences, indicating a redundancy of ~43% within our *Ps-n* library. From this, we estimate that approximately 3,675 unique genes were printed on the *Ps-n* microarray since 5,265 *Ps-n* cDNAs from the *Ps-n* library were printed on the microarray. The ESTs, final assembled NR sequences, and annotations are provided in Additional files 2, 3, 4 and 5.

Assembled sequences were compared against NCBI’s NR and Swissprot databases using the Basic Local Alignment Search tools, blastx and tblastn, via theBlast2Go application [66-68]. The *Ps-n* sequences from this study were also compared against the unpublished *Pseudo-nitzschia multiseries* CLN-47 genome sequence, Assembly v1 (October 2011), sequenced by the US Department of Energy Joint Genome Institute (JGI) [22]. The reference and target genes specifically discussed in this paper were further annotated using NCBI’s ORF Finder [69] and BlastP [66,67], and the Center for Biological Sequence Analysis’ (Technical University of Denmark) ChloroP 1.1, TargetP 1.1, and SignalP 4.1 [70], as well as searching for the conserved diatom AFAP chloroplast targeting domain [71-73].

### Microarray construction

cDNA preparation: 5,265 clones from the *Ps-n* cDNA library were grown overnight in Luria broth with carbenicillin (50 μg mL^-1^) at 37°C on a shaker table. Three “vector-only” and twelve *Homo sapiens* cDNA control clones (J. Pelletier) were also grown under these conditions. Ten microliters of bacterial culture were used in 100-μL PCR reactions with primers T7 forward (TAATACGACTCACTATAGGG) and M13 reverse (CAGGAAACAGCTATGAC), which flanked the cloning site of the pMD1 vector. PCR amplification was performed using HiFI Taq polymerase (Invitrogen Corporation). An initial DNA denaturation step at 94°C for 2 min was followed by 35 amplification cycles (0:30 melting at 94°C, 0:30 annealing at 55°C, 1:00 extension at 68°C). PCR products were purified using MultiScreen size-exclusion filter plates (Millipore, Billerica, MA, USA). The DNA was then resuspended in 100 μL of nuclease-free de-ionized water and transferred to clean plates using a mechanical pipetting station. DNA quality was verified by 1% agarose gel electrophoresis for eight samples per 96-well plate; DNA concentration was determined by PicoGreen fluorescent staining [63]. Fifty μL of each PCR product was dried by vacuum centrifugation and then resuspended in 10 μL of 1.5 M Betain /3X SSC print buffer, yielding an average final concentration of 600 ng μL^-1^.

**Figure 5:**
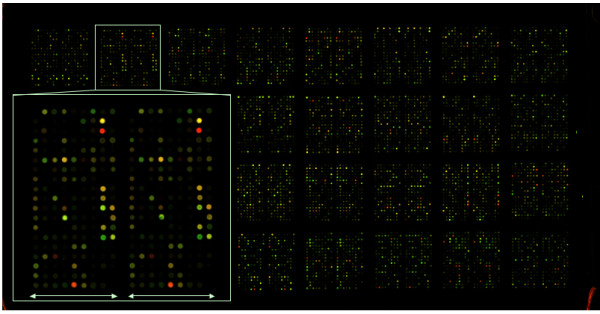
**Scanning fluorescence image of the *Pseudo-nitzschia multiseries *(*Ps-n*) cDNA microarray hybridized with Cy3- and Cy5-labeled cDNAs from non-toxin-producing vs. toxin-producing cells**. 5,169 individual *Ps-n* cDNAs and 25 control cDNAs were printed in replicate, and 96 *Ps-n* cDNAs were printed in quadruplicate, yielding a final chip including 10,772 features. A representative grid is enlarged to illustrate the sub-division of each 8 × 4 grid into two replicate sections, differentiated by the arrowed lines.

*Ps-n* cDNA probes were printed onto CMT-GAPS slides (Corning, Corning, NY, USA), using a MicroGrid 610 TAS array printer (Biorobotics, Woburn, MA, USA) with quill pins. A total of 5,169 *Ps-n* cDNAs were printed in duplicate, and 96 were printed in quadruplicate; in addition, 3 “vector-only” cDNAs, 12 *H. sapiens* control cDNAs, and 10 control cDNAs from the SpotReport Alien Array Validation System (Stratagene, La Jolla, CA, USA) were printed in duplicate, resulting in a final chip that included 10,772 features. Spots were printed with a 32 print-tip head, producing a lay-out represented by 8 × 4 grids. Each grid was sub-divided into two sections, representing replicate spots. Individual features were 13 μm in diameter and were separated by 130 μm (from one spot to the next). 0.005 μL of ~600 ng μL^-1^ DNA (2–3 ng) was transferred to each spot. Final *Ps-n* arrays displayed a strong signal-to-noise ratio, with virtually no background, as demonstrated visually (Figure 5). Experimental hybridization results also confirmed the high degree of reproducibility between replicate spots on the *Ps-n* chip (See Additional files 1 and 2; and, the corresponding Gene Expression Omnibus (GEO) [74] file, accession number GSE46845).

### RNA preparation and microarray hybridizations

RNA was prepared from cells harvested during both the late-exponential (low-toxin-producing) and stationary (high-toxin-producing) phases for all three biological replicates. Eight liters of culture were harvested at an initial time point during the mid- to late-exponential growth phase and the remaining 7 L were harvested at a final time point during the stationary phase (Figure 1a, 1b). Cell suspensions were centrifuged in 0.5-L aliquots for 15 min at 1,000 *g*, which resulted in loose pellets that were pooled, split among 2–4, 50-mL conical tubes and spun again briefly to remove any remaining liquid. Ten to 20 mL of TRIzol (depending on cell pellet volume) were added to the conical tubes, and the pellets were homogenized for 60 s, frozen in liquid N, and stored at -80°C until RNA extraction. Total RNA was extracted, as above, cleaned with RNeasy columns (Qiagen, Valencia, CA, USA) and run on formaldehyde agarose gels to confirm the quality of the RNA.

Ten micrograms of *Ps-n* RNA from each harvest were spiked with mRNA from the SpotReport Alien Array Validation System, incubated for 10 min at 65°C with oligo-dT and then cooled at 25°C for 5 min. Four microliters of 1 mM Cy3- or Cy5-conjugated dUTPs were added to each RNA sample and the mixtures were incubated at 42°C for 2 min. A master mix, including 4.5 μL of 0.2 M DTT, 18 μL of 5X 1st strand buffer, 1.8 μL of 25 mM dATP, dGTP and dCTP, 1.8 μL of 10 mM dTTP, and 2 μL of Superscript II reverse transcriptase, was added to each RNA mixture and incubated for 1 h at 42°C. After 1 h, an additional 1 μL of Superscript II was added to each and the reactions were incubated at 42°C for another hour. Starting RNA was degraded by addition of stop solution (3 μL of 0.5 M EDTA, pH 8; 3 μL of 1 N NaOH) and incubated for 30 min at 60°C. Labeled cDNA was cleaned using RNeasy columns (Qiagen); Cy3-labeled cDNA and the corresponding Cy5-labeled cDNA that were to be compared were combined and loaded onto the same column. The labeled target cDNA pools were then hybridized to the probe cDNAs on the *Ps-n* cDNA microarrays (construction described above). *Ps-n* microarrays were processed before hybridization by holding them face-down over a steaming water bath for a few seconds, and then snap-drying them on a 95°C heat block. The DNA was immobilized onto the slides by UV cross-linking at 65 mJoules. Cross-linked slides were soaked for 15 min in freshly prepared succinic anhydride/sodium borate solution with gentle agitation, soaked for 2 min in boiling nuclease-free, de-ionized water and finally, rinsed in 95% ethanol, and spun dry. Processed microarrays were pre-hybridized at room temperature for 1 h. Pre-hybridization solution was composed of 50% formamide, 5X SSC, 0.1% SDS, and 1% BSA. Hybridization buffer was composed of 50% formamide, 10X SSC, 0.2% SDS, and 0.26% salmon sperm. Labeled cDNA was denatured prior to hybridization by heating for 2 min at 80°C, while the cassette and microarray were pre-warmed at 42°C. The cDNA was then loaded onto the array, and arrays were hybridized for 16 h at 42°C in humidified chambers. Hybridized arrays were washed successively in 1X SSC, 0.03% SDS, 0.1X SSC, 0.01% SDS, and 0.1X SSC, and dried by brief centrifugation.

Replicate hybridizations were repeated within each biological experiment and dye-swapped to account for differences in dye labeling and detection efficiencies. Non-axenic experiments 1 and 2 each included six technical replicates, while the axenic experiment included four technical replicates.

### Microarray image analysis and normalization

Dual-channel arrays were scanned at 595 nm (Cy3) and 685 nm (Cy5) on ArrayWoRx scanners (Applied Precision, Inc., Issaquah, WA, USA). The scanning system converts signal from fluors to “pixel” values, which allows the data to be saved as tiff files. DigitalGenome software (MolecularWare, Cambridge, MA, USA) was then used to integrate annotated chip information with the tiff files and to visualize, edit and export the data for normalization. A loess algorithm was applied to the spot mean intensity values across replicate arrays within each biological experiment to correct for systematic biases using S+ArrayAnalyzer software (Insightful Corp., Seattle, WA, USA) [75,76]. Quality control included analyzing final intensity ratios for the control set of data after normalization. The normalized intensity data for each control spike that corresponded to time zero (T0) and time final (TF) experimental mRNAs in the labeling reactions were analyzed using linear regression analysis to verify that the mean integrated intensity across the control spots was equal (slope ≈ 1). The slope of the linear regression of T0 to TF control intensity values averaged across arrays approached 1 for all three biological experiments: Non-axenic experiment 1 = 0.95, R^2^ = 0.97; Non-axenic experiment 2 = 0.91, R^2^ = 0.95; Axenic experiment = 0.91, R^2^ = 0.98. Any negative values, outliers (defined as two standard deviations away from the mean for individual spots), and any spots that did not include data for at least three replicate arrays within each dataset, were removed from further analysis. These parameters resulted in the removal of data for <1.5% of the original 10,772 features printed on array (final datasets: Axenic experiment 1 = 10,723; Axenic experiment 2 = 10,614; Non-axenic experiment = 10,630).

### Microarray statistics

Significance analysis of gene expression was performed using a t-test algorithm modified for multiple tests: Significance Analysis of Microarrays (SAM) [77] [http://www-stat.stanford.edu/~tibs/SAM/]. SAM reports those genes with statistically significant differences between treatments based on an overall false discovery rate (FDR). A score d(i) is assigned to each gene based on changes in gene expression relative to the standard deviation of repeated measurements. The FDR is an estimate of the percentage of genes identified by chance that would have an observed relative difference d(i) greater than the expected relative difference dE(i) set by an adjustable threshold, delta. An FDR of 1% estimates that for every 100 genes called significant, less than one would be identified incorrectly. The FDR may be adjusted by changing the delta and fold-change thresholds. While SAM does not report individual p-values, each gene is assigned its own “local FDR” (LFDR), which is the comparable statistical measurement to identify individual genes with changes in expression. LFDR can be used to review the data beyond the defined set of differentially expressed genes based on FDR. Hong *et al.*[78] demonstrated an LFDR of 10% as a reliable cut-off to successfully identify changes in expression for specific genes. While the FDR is considered the most reliable measure of the statistically accurate gene list within an experiment, the LFDR offers a second method for reviewing the statistical likelihood of changes in expression for a particular gene. In our study, we used the overall FDR to define the initial set of differentially expressed genes. We used the LFDR to confirm the overall change in gene expression for those transcripts that had multiple cDNAs printed on the microarray.

Initially, each dataset in our study was analyzed independently. Non-axenic experiments 1 and 2 were analyzed for statistical significance using a relatively stringent fold-change cut-off of 2.5 to target genes that were substantially up- or down-regulated during the transition to stationary phase, when toxin was produced. A delta value of 0.275 resulted in overall FDRs that were <1% in both of these experiments. Expression levels were consistently lower in the axenic experiment as compared to the non-axenic experiments. For example, the cDNAs with positive fold-change differences averaged 4.07 ± 0.97 in Non-axenic experiment 1, 3.85 ± 1.17 Non-axenic experiment 2, and 1.92 ± 0.54 in the Axenic growth experiment. Therefore, a lower fold-change cut-off of 1.5, and a delta value of 0.275, resulted in a comparable FDR that was <2.5% for the Axenic experiment. Only those transcripts that were determined to be significantly up- or down-regulated in all three biological experiments were further analyzed.

Two layers of replicates (replicate cDNAs on the array and replicate hybridizations) were accounted for by first analyzing the replicates spots as uncollapsed, independent data points using the normalization and statistical analysis described above. Then, replicate spots were averaged and collapsed, accordingly, depending on whether or not they fell into a greater contig. For singletons, both replicate spots were required to be statistically significant in all three biological experiments to be further considered. In this case, replicate spots were averaged for a final expression ratio and standard deviation. For cDNA features that fell into a larger contig, 90% of the cDNAs that fell within the contig were required to be significantly differentially expressed (based on initial SAM analysis or LFDR) to further consider the overall contig as up- or down-regulated. In this case, the replicates were collapsed by averaging the mean ratios of all cDNAs for a final fold-change ratio and standard deviation for the overall contig. The individual cDNAs within contigs served as additional replicates for these transcripts, and the results confirmed the consistent change in gene expression (see Additional file 1). The final fold-change values for those transcripts that were statistically higher or lower in stationary (toxin-producing) as compared to late-exponential (low-toxin-producing) growth phase are presented in Tables 3 and 4. Additional data files complying with MIAME format [79] were deposited at the GEO [74] data repository, accession number GSE46845.

### Primer design and validation for RT-qPCR

Primers were designed manually to be within a length of 18–26 nucleotides with a GC content between 50-65%. These values resulted in high sequence specificity and melting temperatures (Tm) that worked well under our assay conditions using an annealing temperature of 60°C. JmjC forward, ATPase reverse and SLC6 Ex-Ex forward had lower GC contents than the original specifications, but still worked efficiently under these assay conditions. All primer sets were designed for PCR amplicons of 50–200 bp in length. Primers were synthesized by Integrated DNA Technologies, Inc. (Coralville, IA, USA) and purified by standard desalting. Efficiencies of amplification were initially determined for each primer set by running standard curves with 5-fold serial dilutions of *Ps-n* cDNA derived from stationary phase cultures, as well as genomic DNA. PCR conditions are described, below. Primer sequences and information can be found in Tables 2 and 5. Reported efficiencies in the tables correspond with the initial cDNA standard curve analyses. Standard curves were also run using 2-fold serial dilutions of pooled cDNA from the experimental samples combined in equal amounts. The primer sets used in the Si-limitation experimental analyses showed efficiencies >95%, with R^2^ values >0.99. Primers that span an exon-exon junction were designed for one reference and two of the target genes; these primer sets did not yield a product using gDNA as a template.

### RNA isolation, cDNA synthesis and RT-qPCRs

Total RNA was extracted daily from each flask beginning on day three of growth. Cells were collected from 250 mL of culture by filtering onto a 5.0-μm, 47-mm membrane filter (MF-Millipore mixed cellulose ester). Filters were transferred to 50 mL conical tubes and 3 mL of TRIzol were added. Cells were washed quickly and gently from the filter and homogenized at full speed with a Polytron homogenizer (Kinematica, Inc, Bohemia, NY) for 90 s. Samples were incubated at room temperature for 5 min following homogenization and centrifuged at 3000 *g* for 10 min at 4°C in order to pellet cellular debris. 200 μL of chloroform was added for every 1 mL of homogenate and samples were incubated for 3 min with periodic shaking at room temperature. Samples were centrifuged at 12,000 *g* for 20 min at 4°C in order to separate the aqueous and organic phases. 75-80% of the aqueous phase was transferred to fresh tubes and an equal volume of 70% EtOH was added. The RNA-EtOH mixture was cleaned using RNeasy mini columns with on-column RNAase-free DNAase digestion (Qiagen). Clean RNA samples were eluted in 100 μL of DEPC-treated water, and stored at -80°C until analyzed. RNA concentrations were analyzed using a Nanodrop 2000 spectrophotometer (Wilmington, DE, USA), and RNA quality was verified by gel electrophoresis using Lonza 1.2% RNA cassettes (Walkersville, MD, USA). RNA samples were diluted to 20 ng μL^-1^. 600 ng of total RNA from each sample was reverse transcribed using the iscript cDNA synthesis kit in 50 μL volume reactions, using both poly A and random hexamer primers (Bio-Rad Laboratories, Inc., Hercules, CA, USA). The reverse transcription reaction was carried out by incubating at 25°C for 5 min, followed by 42°C for 30 min. The enzyme was deactivated by heating to 85°C for 5 min and samples were held at 4°C until retrieved and stored at -20°C.

RT-qPCR reactions were set up as follows: 10 μL of SYBR Green PCR mix (Bio-Rad Laboratories, Inc.), 0.75 μL of cDNA, 0.2 μM of forward primer, 0.2 μM of reverse primer, and nuclease-free water to a final volume of 20 μL. An exception was that the β-tubulin Ex-Ex efficiency was within the 95-105% range using 0.4 μM for both forward and reverse primers. Each experimental cDNA was amplified in triplicate for each primer set using the following cycling parameters: 1) 95°C for 3 min; 2) 95°C for 10 s; 3) 60°C for 15 s; 4) 72°C for 30 s (plate read); 5) repeat 39 more cycles of steps 2–4; 6) 72°C for 10 min; 8) melting curve analysis from 65-95°C in 0.5°C increments every 5 s; and 9) hold at 4°C. Cq values were determined for each reaction at 150 relative fluorescent units.

### Evaluation of DNA contamination in RT-qPCRs

qPCR reactions were run on 1 μL of each RNA from the Si-limitation growth experiment to test for amplification due to contaminating DNA. The absence of DNA contamination was further confirmed by using both standard and exon-exon spanning primer sets in parallel for one control and two target genes in the RT-qPCR reactions (Figures 3 and 4).

### Analysis of candidate reference gene expression stability

Cq values were inputted into the GeNorm plus algorithm [20,80] and a stability value (M-value) was calculated for each gene (Figure 2A, 2B). Genes with the lowest M-values are considered the most stable; M-values <0.5 are ideal for use in normalization of qPCR data. The optimum number of reference genes to use for normalization is determined by calculating the geometric average of the two, three, four, and five most stable genes. The pairwise-variation between subsequent normalization factors is calculated and when the variation is below 0.15, the amount of change caused by the addition of the new control gene is considered negligible, and therefore unnecessary to include in subsequent normalization calculations.

### Normalization, quantification, and statistics of RT-qPCR analysis

The arithmetic mean of triplicate technical replicates was calculated and used for subsequent calculations. The ΔCq values were calculated by the difference between each sample and the average Cq of the chosen reference point, which was the first time point (T3). Relative quantities (RQ) were calculated by exponentiation of the ΔCqs (2^ΔCq^). RQ values of the target genes, for each sample, were divided by the geometric average of the chosen reference genes’ RQ values, resulting in a normalized relative quantity (NRQ) [19]. The arithmetic mean and standard deviation of biological replicate NRQs were then calculated and plotted (Figures 3 and 4). NRQ values were log transformed (Log_2_ NRQ) into Cq’ values for statistical analysis [81]. Statistically significant changes in gene expression were determined using a general linear model analysis of variance (ANOVA) with Bonferroni *post-hoc* test. Statistical analyses were performed with 95% confidence intervals (p < 0.05) using Minitab16 statistical software (State College, PA, USA). Additional files 6 and 7 contain the complete set of RT-qPCR data and statistical results. RT-qPCR data analyses and reporting were in accordance with MIQE guidelines [82,83].

### Availability of supporting data

The microarray data supporting the results discussed in this paper are included within the paper, and in Additional files 1 and 2. The microarray data are also available in the NCBI’s GEO repository, and are accessible through the GEO Series accession number GSE46845. The sequence data and annotations are presented in Additional files 2, 3, 4 and 5. The ESTs are also available through the NCBI dbEST database [GenBank accession numbers FD476666-FD480212]. The RT-qPCR data and statistical results are available in Additional files 6 and 7.

## Abbreviations

ASP: Amnesic shellfish poisoning; cDNA: Complementary DNA; DA: Domoic acid; EF-1α: Elongation factor 1-alpha, eIF-2, Elongation initiation factor 2; ESTs: Expressed sequence tags; FCP: Fucoxanthin-chlorophyll a-c binding protein; FDR: False discovery rate; FMOC: Fluorenylmethoxycarbonyl; GABA: γ-aminobutyric; GAPDH: Glyceraldehyde-3-phosphate dehydrogenase; GDH: Glutamate dehydrogenase; HPLC: High performance liquid chromatography; LFDR: Local false discovery rate; MIAME: Minimum information about a microarray experiment; PCR: Polymerase chain reaction; PEPCK: Phosphoenolpyruvate carboxykinase; PGK: Phosphoglycerate kinase; PFK: Phosphofructokinase; Ps-n: *Pseudo-nitzschia multiseries*; RT-qPCR: Reverse-transcription quantitative PCR; SAM: Significance analysis of microarrays; SLC6: Solute carrier family 6.

## Competing interests

The authors declare that they have no competing interests.

## Authors’ contributions

KRB contributed to the design, completion, and analysis of the RT-qPCR and microarray studies, and wrote the manuscript. BMH contributed to the design, completion, and analysis of the RT-qPCR experiments. KRB and JP constructed the cDNA library and sequencing was completed in JP’s lab at McGill University (or ACGT, Inc., for a small subset of samples). KRB and SM completed the microarray construction and data analysis. DLR participated in data review and in revising the manuscript. SSB participated in the design of the array studies, data review, and in drafting the manuscript. HPLC assays were completed in SSB’s lab in Moncton, NB (Fisheries and Oceans Canada). DAH contributed to the design of the RT-qPCR studies, ELISA domoic acid analyses, data review and in revising the manuscript. DEH contributed to the design of the RT-qPCR and microarray studies, data review and analyses, and drafting the manuscript. Microarray studies were completed in DEH’s lab at MIT; RT-qPCR studies were completed in KRB’s lab at PSU. All authors read and approved the final manuscript.

## Supplementary Material

Additional file 1 **Fold-change data and statistics for cDNA replicates on the *Ps-n *** **microarray for each of the transcripts discussed in this paper.** Click here for file

Additional file 2 **Excel file with the annotation and array data for the entire set of cDNA clones printed on the *Ps-n *** **microarray.** Click here for file

Additional file 3 **Fasta file with all of the *Ps-n *** **ESTs from this study.** Click here for file

Additional file 4 Fasta file with all of the assembled sequences from this study. Click here for file

Additional file 5 **Contig alignments in .ace format.** A number of the alignments (46A5, 53B6, 73D12, 135H6, 165G9, 177F1, PSN0011, PSN0014, PSN0016, PSN0019, PSN0032, PSN0042, PSN0060, PSN0072, PSN0080, PSN0100, PSN0332, PSN0547, PSN0918, and PSN1327) include sequences from the JGI *Pseudo-nitzschia* genome project [22] for comparison; these sequences are designated within the contig by the JGI modeled gene name or genome location. Note that contigs corresponding with three transcripts discussed in the manuscript, PSN0014, PSN0016, and PSN0052, showed splice variants. Expression of the individual cDNAs within these contigs were not significantly different in the microarray analysis (see Additional file 1). Click here for file

Additional file 6 RT-qPCR data. Click here for file

Additional file 7 RT-qPCR statistical results. Click here for file
