# Supplementary material for: Gene expression studies for the analysis of domoic acid production in the marine diatom Pseudo-nitzschia multiseries
Source: BMC Mol Biol. 2013 Nov 1;14:25. doi: 10.1186/1471-2199-14-25 (PMC3832940; doi:10.1186/1471-2199-14-25)
Supplement: Additional file 1 — Fold-change data and statistics for cDNA replicates on the Ps-n microarray for each of the transcripts discussed in this paper. [file 1471-2199-14-25-S1.pdf]

**Additional file 1:** Fold-change data and statistics for cDNA replicates on the *Ps-n* microarray for each of the transcripts discussed in Boissonneault et al. (2013). The entire dataset, including genes not specifically discussed in the manuscript, is also available in Additional file 2 and the corresponding GEO File (Accession #GSE46845). Transcripts that were identified as differentially expressed based on the overall FDR are listed as 'significant'. Local FDR (LFDR) percentages are also provided to further review the data beyond the originally defined set based on FDR. (See Methods).

| **UP-REGULATED TRANSCRIPTS** | **Clone** | **Spot I.D.** | **Stationary versus exponential Growth Phase** | | | | | | | | |
|---|---|---|---|---|---|---|---|---|---|---|---|
| | | | **Non-axenic Expt. 1, Fold-change** | **Overall FDR** | **LFDR (%)** | **Non-axenic Expt. 2, Fold-change** | **Overall FDR** | **LFDR (%)** | **Axenic Expt., Fold-change** | **Overall FDR** | **LFDR (%)** |
| **PSN0011** | 45 G9 | 10139 | **3.93** | Significant | 0.00 | **3.08** | Significant | 0.00 | **2.29** | Significant | 0.00 |
| **Cycloisomerase** | 45 G9 | 10329 | **4.15** | Significant | 0.00 | **3.08** | Significant | 0.00 | **2.45** | Significant | 0.00 |
| | 51 F10 | 8106 | **4.48** | Significant | 0.10 | **3.24** | Significant | 0.00 | **2.14** | Significant | 0.00 |
| | 51 F10 | 8296 | **4.53** | Significant | 0.08 | **3.35** | Significant | 0.00 | **2.16** | Significant | 0.00 |
| | 51 F9 | 10994 | **5.03** | Significant | 0.10 | **3.46** | Significant | 0.00 | **2.51** | Significant | 0.00 |
| | 51 F9 | 11184 | **5.18** | Significant | 0.09 | **3.53** | Significant | 0.00 | **2.44** | Significant | 0.00 |
| | 52 B1 | 9432 | **3.92** | NS | 0.00 | **2.25** | NS | 0.00 | **1.83** | Significant | 1.19 |
| | 52 B1 | 9622 | **3.86** | NS | 0.00 | **2.40** | NS | 0.00 | **1.87** | Significant | 0.00 |
| | 52 E12 | 56 | **4.18** | Significant | 0.00 | **3.33** | Significant | 0.00 | **2.23** | Significant | 0.00 |
| | 52 E12 | 246 | **3.65** | Significant | 0.00 | **3.68** | Significant | 0.00 | **2.49** | Significant | 0.00 |
| | 54.1 B7 | 4129 | **3.60** | Significant | 0.00 | **2.72** | Significant | 0.00 | **1.76** | Significant | 0.00 |
| 54 B7 included four replicates, so 54.1 averaged | 54.1 B7 | 4319 | **3.68** | Significant | 0.00 | **2.65** | Significant | 0.00 | **1.80** | Significant | 0.00 |
| 54.1 B7 average used in overall contig average | 54.1 AVG | | **3.64** | Significant | | **2.68** | Significant | | **1.78** | Significant | |
| | 54.2 B7 | 5118 | **2.84** | Significant | 0.00 | **2.91** | Significant | 0.00 | **1.80** | Significant | 0.00 |
| 54 B7 included four replicates, so 54.2 averaged | 54.2 B7 | 5308 | **3.02** | Significant | 0.00 | **2.68** | Significant | 0.00 | **1.83** | Significant | 0.00 |
| 54.2 B7 average used in overall contig average | 54.2 AVG | | **2.93** | Significant | | **2.79** | Significant | | **1.81** | Significant | |
| | 55 C4 | 1878 | **3.45** | Significant | 0.00 | **3.37** | Significant | 0.00 | **2.22** | Significant | 0.00 |
| | 55 C4 | 2068 | **3.42** | Significant | 0.00 | **3.65** | Significant | 0.03 | **2.21** | Significant | 0.00 |
| | 57 F7 | 5106 | **4.64** | Significant | 0.00 | **3.04** | Significant | 0.00 | **2.44** | Significant | 0.00 |
| | 57 F7 | 5296 | **4.30** | Significant | 0.01 | **3.10** | Significant | 0.00 | **2.41** | Significant | 0.00 |
| | 51 A4 | 1598 | **4.34** | Significant | 0.00 | **3.31** | Significant | 0.00 | **2.70** | Significant | 0.00 |
| | 51 A4 | 1788 | **4.57** | Significant | 0.00 | **3.46** | Significant | 0.00 | **1.95** | Significant | 23.68 |
| | 6 F2 | 6520 | **4.30** | Significant | 0.00 | **3.28** | Significant | 0.00 | **2.36** | Significant | 0.00 |
| | 6 F2 | 6710 | **4.34** | Significant | 0.00 | **3.30** | Significant | 0.00 | **2.38** | Significant | 0.00 |
| | 71 H1 | 10865 | **5.24** | Significant | 0.11 | **3.55** | Significant | 0.00 | **2.74** | Significant | 0.00 |
| | 71 H1 | 11055 | **4.91** | Significant | 0.10 | **3.57** | Significant | 0.00 | **2.65** | Significant | 0.00 |
| | 71 H3 | 5089 | **4.21** | Significant | 0.00 | **2.65** | Significant | 0.00 | **1.97** | Significant | 0.00 |
| | 71 H3 | 5279 | **4.27** | Significant | 0.00 | **3.05** | Significant | 0.00 | **2.08** | Significant | 0.00 |
| | 76 D7 | 5426 | **3.74** | NS | 0.00 | **2.26** | NS | 0.00 | **1.68** | Significant | 10.97 |
| | 76 D7 | 5616 | **3.83** | NS | 0.00 | **2.33** | NS | 0.00 | **1.65** | Significant | 11.44 |
| | 135 E8 | 1939 | **3.76** | Significant | 0.00 | **6.49** | Significant | 0.00 | **2.66** | Significant | 0.00 |
| | 135 E8 | 2129 | **4.32** | Significant | 0.05 | **14.33** | Significant | 0.10 | **2.49** | Significant | 0.00 |
| | 136 D7 | 4090 | **4.16** | Significant | 0.05 | **3.22** | Significant | 0.00 | **2.26** | Significant | 0.00 |
| | 136 D7 | 4280 | **4.57** | Significant | 0.06 | **3.28** | Significant | 0.00 | **2.29** | Significant | 0.00 |
| | 163 D4 | 823 | **4.47** | Significant | 0.11 | **3.78** | Significant | 0.00 | **2.35** | Significant | 0.00 |
| | 163 D4 | 1013 | **4.58** | Significant | 0.10 | **3.83** | Significant | 0.03 | **2.42** | Significant | 0.00 |
| | 169 H10 | 8093 | **5.51** | Significant | 0.11 | **3.87** | Significant | 0.00 | **2.80** | Significant | 0.00 |
| | 169 H10 | 8283 | **5.37** | Significant | 0.09 | **3.94** | Significant | 0.00 | **2.77** | Significant | 0.00 |
| | 180 E5 | 10535 | **4.22** | Significant | 0.02 | **4.34** | Significant | 0.00 | **2.49** | Significant | 0.00 |
| | 180 E5 | 10725 | **5.54** | Significant | 0.10 | **3.84** | Significant | 0.00 | **2.46** | Significant | 0.00 |
| | 182 D11 | 5444 | **3.10** | NS | 0.00 | **2.30** | NS | 0.00 | **1.66** | Significant | 9.38 |
| | 182 D11 | 5634 | **3.24** | Significant | 0.00 | **2.50** | Significant | 0.00 | **1.65** | Significant | 10.74 |
| | 183 G5 | 10616 | **2.32** | NS | 0.00 | **2.77** | NS | 0.00 | **1.58** | Significant | 10.44 |
| | 183 G5 | 10806 | **2.40** | NS | 0.00 | **5.17** | NS | 5.61 | **1.49** | NS | 11.80 |
| | 180 D8 | 2592 | **1.23** | NS | 43.12 | **0.99** | NS | 75.87 | **1.89** | Significant | 0.00 |
| | 180 D8 | 2782 | **1.21** | NS | 43.39 | **1.11** | NS | 71.43 | **1.86** | Significant | 0.00 |
| | **Average** | | **4.02** | | | **3.49** | | | **2.20** | | |
| | **St. Dev.** | | **0.97** | | | **1.94** | | | **0.36** | | |
| **PSN0072** | 30 H7 | 3651 | **3.72** | Significant | 0.00 | **3.87** | Significant | 0.00 | **2.12** | Significant | 0.00 |
| **SLC6 Transporter** | 30 H7 | 3841 | **3.83** | Significant | 0.00 | **3.67** | Significant | 0.00 | **2.17** | Significant | 0.00 |
| | 189 C3 | 4794 | **3.27** | Significant | 0.00 | **3.24** | Significant | 0.00 | **1.90** | Significant | 0.00 |
| | 189 C3 | 4984 | **3.52** | Significant | 0.00 | **3.39** | Significant | 0.00 | **2.13** | Significant | 0.00 |
| | 54.1 A7 | 3407 | **2.95** | Significant | 0.00 | **3.45** | Significant | 0.00 | **1.60** | Significant | 7.16 |
| 54 A7 included four replicates, so 54.1 averaged | 54.1 A7 | 3597 | **2.60** | Significant | 0.00 | **2.91** | Significant | 0.00 | **1.56** | Significant | 11.78 |
| 54.1 A7 average used in overall contig average | 54.1 AVG | | **2.78** | Significant | | **3.18** | Significant | | **1.58** | Significant | |
| | 54.2 A7 | 4396 | **3.28** | Significant | 0.00 | **3.05** | Significant | 0.00 | **2.19** | Significant | 5.77 |
| 54 A7 included four replicates, so 54.2 averaged | 54.2 A7 | 4586 | **3.31** | Significant | 0.00 | **2.93** | Significant | 0.00 | **2.14** | Significant | 5.14 |
| 54.2 A7 average used in overall contig average | 54.2 AVG | | **3.29** | Significant | | **2.99** | Significant | | **2.16** | Significant | |

| | | | | | | | | | | | |
|---|---|---|---|---|---|---|---|---|---|---|---|
| | **Average** | | **3.40** | | | **3.39** | | | **2.01** | | |
| | **St. Dev.** | | **0.38** | | | **0.32** | | | **0.24** | | |
| **PSN0014** | 135 B6 | 8473 | **5.99** | Significant | 0.09 | **5.09** | Significant | 0.05 | **2.52** | Significant | 0.00 |
| **Acyl-CoA synthetase** | 135 B6 | 8663 | **5.96** | Significant | 0.10 | **4.90** | Significant | 0.03 | **2.54** | Significant | 0.00 |
| | 173 H1 | 9916 | **4.82** | Significant | 0.11 | **3.59** | Significant | 0.00 | **2.31** | Significant | 0.00 |
| | 173 H1 | 10106 | **4.94** | Significant | 0.10 | **4.13** | Significant | 0.00 | **2.10** | Significant | 0.00 |
| | 176 H5 | 9840 | **5.46** | Significant | 0.11 | **3.98** | Significant | 0.00 | **2.27** | Significant | 0.00 |
| | 176 H5 | 10030 | **5.38** | Significant | 0.11 | **4.11** | Significant | 0.00 | **2.32** | Significant | 0.00 |
| | 178 G1 | 8727 | **5.04** | Significant | 0.11 | **4.17** | Significant | 0.00 | **2.14** | Significant | 0.00 |
| | 178 G1 | 8917 | **4.53** | Significant | 0.08 | **3.50** | Significant | 0.00 | **2.15** | Significant | 0.00 |
| | 180 G9 | 10540 | **4.41** | Significant | 0.04 | **5.15** | Significant | 0.04 | **2.05** | Significant | 0.00 |
| | 180 G9 | 10730 | **4.21** | Significant | 0.00 | **5.96** | Significant | 0.04 | **2.11** | Significant | 0.00 |
| | 30 C7 | 2927 | **4.37** | Significant | 0.00 | **3.95** | Significant | 0.00 | **1.88** | Significant | 21.10 |
| | 30 C7 | 3117 | **5.52** | Significant | 0.06 | **4.82** | Significant | 0.00 | **2.50** | Significant | 0.00 |
| | 45 F3 | 5076 | **5.26** | Significant | 0.11 | **5.40** | Significant | 0.10 | **2.27** | Significant | 0.00 |
| | 45 F3 | 5266 | **5.23** | Significant | 0.11 | **5.19** | Significant | 0.11 | **2.34** | Significant | 0.00 |
| | 47 E10 | 7330 | **4.16** | Significant | 0.08 | **4.30** | Significant | 0.00 | **1.83** | Significant | 1.50 |
| | 47 E10 | 7520 | **4.10** | Significant | 0.08 | **3.66** | Significant | 0.02 | **1.86** | Significant | 0.18 |
| | 57 A3 | 4378 | **4.55** | Significant | 0.02 | **3.71** | Significant | 0.00 | **2.26** | Significant | 0.00 |
| | 57 A3 | 4568 | **4.65** | Significant | 0.08 | **3.29** | Significant | 0.00 | **2.17** | Significant | 0.00 |
| | 57 E5 | 10160 | **2.63** | Significant | 0.00 | **2.72** | Significant | 0.00 | **1.71** | Significant | 25.12 |
| | 57 E5 | 10350 | **3.29** | Significant | 0.00 | **2.62** | Significant | 0.00 | **1.82** | Significant | 7.38 |
| | 72 E5 | 10507 | **4.95** | Significant | 0.09 | **4.41** | Significant | 0.10 | **2.27** | Significant | 0.00 |
| | 72 E5 | 10697 | **5.17** | Significant | 0.10 | **4.31** | Significant | 0.10 | **2.26** | Significant | 0.00 |
| | 76 B6 | 8313 | **5.83** | Significant | 0.10 | **4.72** | Significant | 0.02 | **2.51** | Significant | 0.00 |
| | 76 B6 | 8503 | **5.47** | Significant | 0.11 | **4.87** | Significant | 0.05 | **2.44** | Significant | 0.00 |
| | 182 H3 | 5438 | **3.57** | Significant | 0.00 | **2.75** | Significant | 0.00 | **1.98** | Significant | 0.00 |
| | 182 H3 | 5628 | **3.55** | Significant | 0.00 | **3.02** | Significant | 0.00 | **2.11** | Significant | 0.00 |
| | 183 H5 | 11338 | **3.23** | Significant | 0.00 | **2.92** | Significant | 0.00 | **1.62** | Significant | 9.77 |
| | 183 H5 | 11528 | **3.56** | Significant | 0.00 | **3.50** | Significant | 0.00 | **1.59** | Significant | 12.71 |
| | **Average** | | **4.64** | | | **4.10** | | | **2.14** | | |
| | **St. Dev.** | | **0.89** | | | **0.89** | | | **0.27** | | |
| **PSN0016** | 160 E2 | 6211 | **3.95** | Significant | 0.02 | **3.49** | Significant | 0.01 | **3.70** | Significant | 0.09 |
| **Phosphoenolpyruvate carboxykinase** | 160 E2 | 6401 | **3.75** | Significant | 0.02 | **3.54** | Significant | 0.04 | **3.61** | Significant | 0.07 |
| | 165 A3 | 4805 | **3.42** | Significant | 0.00 | **3.26** | Significant | 0.00 | **3.51** | Significant | 0.03 |
| | 165 A3 | 4995 | **3.43** | Significant | 0.00 | **3.14** | Significant | 0.00 | **3.57** | Significant | 0.05 |
| | 166 F6 | 6590 | **4.10** | Significant | 0.02 | **2.71** | Significant | 0.00 | **2.65** | Significant | 0.00 |
| | 166 F6 | 6780 | **4.19** | Significant | 0.02 | **2.77** | Significant | 0.00 | **2.66** | Significant | 0.00 |
| | 171 B10 | 8397 | **3.90** | Significant | 0.00 | **2.73** | Significant | 0.00 | **2.72** | Significant | 0.00 |
| | 171 B10 | 8587 | **3.82** | Significant | 0.00 | **2.96** | Significant | 0.00 | **2.68** | Significant | 0.00 |
| | 174 F8 | 868 | **3.63** | Significant | 0.00 | **2.91** | Significant | 0.00 | **2.91** | Significant | 0.00 |
| | 174 F8 | 1058 | **3.73** | Significant | 0.00 | **2.83** | Significant | 0.00 | **3.07** | Significant | 0.00 |
| | 184 H1 | 9796 | **3.68** | Significant | 0.00 | **2.69** | Significant | 0.00 | **2.82** | Significant | 0.00 |
| | 184 H1 | 9986 | **3.71** | Significant | 0.00 | **2.64** | Significant | 0.00 | **2.69** | Significant | 0.00 |
| | 186 G8 | 508 | **3.76** | Significant | 0.00 | **3.39** | Significant | 0.00 | **3.23** | Significant | 0.00 |
| | 186 G8 | 698 | **3.84** | Significant | 0.00 | **3.38** | Significant | 0.00 | **3.34** | Significant | 0.06 |
| | 187 D6 | 8033 | **3.85** | Significant | 0.00 | **2.71** | Significant | 0.00 | **3.25** | Significant | 0.00 |
| | 187 D6 | 8223 | **3.92** | Significant | 0.00 | **2.67** | Significant | 0.00 | **2.92** | Significant | 0.00 |
| | 25 D11 | 4054 | **4.36** | Significant | 0.00 | **3.03** | Significant | 0.00 | **3.13** | Significant | 0.03 |
| | 25 D11 | 4244 | **4.45** | Significant | 0.00 | **2.99** | Significant | 0.00 | **3.19** | Significant | 0.00 |
| | 50 C2 | 7333 | **3.64** | Significant | 0.00 | **3.06** | Significant | 0.00 | **3.16** | Significant | 0.00 |
| | 50 C2 | 7523 | **3.58** | Significant | 0.00 | **3.00** | Significant | 0.00 | **3.17** | Significant | 0.00 |
| | 51 D11 | 5217 | **3.57** | Significant | 0.00 | **2.62** | Significant | 0.00 | **2.23** | Significant | 0.00 |
| | 51 D11 | 5407 | **3.55** | Significant | 0.00 | **2.54** | Significant | 0.00 | **2.12** | Significant | 0.00 |
| | 51 H7 | 5215 | **3.76** | Significant | 0.00 | **3.10** | Significant | 0.00 | **3.59** | Significant | 0.06 |
| | 51 H7 | 5405 | **3.73** | Significant | 0.00 | **3.10** | Significant | 0.00 | **3.52** | Significant | 0.04 |
| | 75 E8 | 1456 | **3.22** | Significant | 0.00 | **3.17** | Significant | 0.00 | **4.02** | Significant | 0.00 |
| | 75 E8 | 1646 | **3.47** | Significant | 0.00 | **3.23** | Significant | 0.00 | **4.00** | Significant | 0.00 |
| | 78 C4 | 368 | **3.78** | Significant | 0.00 | **3.15** | Significant | 0.00 | **2.18** | Significant | 0.03 |
| | 78 C4 | 558 | **3.95** | Significant | 0.00 | **3.41** | Significant | 0.00 | **3.35** | Significant | 0.00 |
| | **Average** | | **3.78** | | | **3.01** | | | **3.11** | | |
| | **St. Dev.** | | **0.27** | | | **0.29** | | | **0.50** | | |
| **PSN0025** | 160 G1 | 9100 | **7.38** | Significant | 0.09 | **7.74** | Significant | 0.04 | **4.42** | Significant | 0.10 |
| **Heat shock protein alpha-crystallin domain** | 160 G1 | 9290 | **7.29** | Significant | 0.11 | **9.27** | Significant | 0.00 | **4.33** | Significant | 0.10 |
| | 167 D8 | 893 | **7.25** | Significant | 0.02 | **7.13** | Significant | 0.02 | **3.83** | Significant | 0.00 |
| | 167 D8 | 1083 | **7.30** | Significant | 0.01 | **7.38** | Significant | 0.01 | **3.92** | Significant | 0.00 |
| | 173 B6 | 7029 | **7.87** | Significant | 0.04 | **7.73** | Significant | 0.08 | **4.74** | Significant | 0.10 |
| | 173 B6 | 7219 | **7.51** | Significant | 0.01 | **7.43** | Significant | 0.11 | **4.81** | Significant | 0.10 |
| | 178 D3 | 3671 | **7.33** | Significant | 0.00 | **8.56** | Significant | 0.00 | **4.52** | Significant | 0.10 |

| | | | | | | | | | | |
|---|---|---|---|---|---|---|---|---|---|---|
| | 178 D3 3861 | **7.10** | Significant | 0.06 | **7.53** | Significant | 0.01 | **4.65** | Significant | 0.10 |
| short insert (~250bp) | 45 H6 7969 | **3.64** | Significant | 0.00 | **3.86** | Significant | 0.04 | **3.22** | Significant | 0.00 |
| short insert (~250bp) | 45 H6 8159 | **3.80** | Significant | 0.00 | **4.08** | Significant | 0.00 | **3.36** | Significant | 0.00 |
| | **Average** | **6.65** | | | **7.07** | | | **4.18** | | |
| | **St. Dev.** | **1.56** | | | **1.75** | | | **0.57** | | |
| **PSN0052** | 51 G5 6211 | **4.42** | Significant | 0.05 | **2.64** | Significant | 0.00 | **1.58** | Significant | 8.47 |
| **Mitochondrial carrier protein** | 51 G5 6401 | **4.75** | Significant | 0.10 | **2.79** | Significant | 0.00 | **1.60** | Significant | 5.17 |
| | 46 G6 4805 | **4.20** | Significant | 0.00 | **2.92** | Significant | 0.00 | **1.56** | Significant | 12.42 |
| | 46 G6 4995 | **4.15** | Significant | 0.00 | **2.68** | Significant | 0.00 | **1.53** | Significant | 7.73 |
| | 163 H9 6590 | **3.70** | Significant | 0.00 | **2.64** | Significant | 0.00 | **1.56** | Significant | 4.38 |
| | 163 H9 6780 | **3.71** | Significant | 0.00 | **2.60** | Significant | 0.00 | **1.57** | Significant | 3.98 |
| | 166 C4 8397 | **3.14** | Significant | 0.00 | **2.28** | NS | 0.00 | **1.56** | Significant | 17.59 |
| | 166 C4 8587 | **3.08** | Significant | 0.00 | **2.33** | NS | 0.00 | **1.59** | Significant | 12.65 |
| | **Average** | **3.89** | | | **2.61** | | | **1.57** | | |
| | **St. Dev.** | **0.59** | | | **0.21** | | | **0.02** | | |
| **PSN0015** | 76 E5 10481 | **3.18** | Significant | 0.00 | **3.13** | Significant | 0.00 | **1.80** | Significant | 0.00 |
| **Aldo /keto reductase** | 76 E5 10671 | **3.50** | Significant | 0.00 | **3.72** | Significant | 0.00 | **1.81** | Significant | 0.00 |
| | 183 G3 4836 | **2.55** | Significant | 0.00 | **2.85** | Significant | 0.00 | **1.61** | Significant | 13.21 |
| | 183 G3 5026 | **2.53** | Significant | 0.00 | **2.91** | Significant | 0.00 | **1.84** | Significant | 0.00 |
| | 178 F12 792 | **3.20** | Significant | 0.00 | **3.07** | Significant | 0.00 | **1.80** | Significant | 1.71 |
| | 178 F12 982 | **3.13** | Significant | 0.00 | **2.87** | Significant | 0.00 | **2.02** | Significant | 0.79 |
| | 175 H10 8081 | **3.58** | Significant | 0.00 | **3.24** | Significant | 0.00 | **2.03** | Significant | 0.00 |
| | 175 H10 8271 | **3.38** | Significant | 0.00 | **3.39** | Significant | 0.00 | **2.06** | Significant | 0.00 |
| | 171 B2 8389 | **4.00** | Significant | 0.00 | **3.92** | Significant | 0.00 | **2.09** | Significant | 0.00 |
| | 171 B2 8579 | **4.39** | Significant | 0.00 | **3.91** | Significant | 0.00 | **2.06** | Significant | 0.00 |
| | 163 C5 8769 | **3.25** | Significant | 0.00 | **2.95** | Significant | 0.00 | **1.82** | Significant | 0.00 |
| | 163 C5 8959 | **2.95** | Significant | 0.00 | **2.79** | Significant | 0.00 | **1.88** | Significant | 0.00 |
| | 161 D12 805 | **2.38** | NS | 0.00 | **2.71** | Significant | 0.00 | **1.53** | Significant | 8.33 |
| | 161 D12 995 | **2.61** | Significant | 0.00 | **2.59** | Significant | 0.00 | **1.72** | Significant | 1.24 |
| | **Average** | **3.19** | | | **3.15** | | | **1.86** | | |
| | **St. Dev.** | **0.57** | | | **0.43** | | | **0.17** | | |
| **PSN0042** | 78 H3 3980 | **6.78** | Significant | 0.06 | **6.95** | Significant | 0.07 | **3.81** | Significant | 0.10 |
| **Predicted protein with signal peptide** | 78 H3 4170 | **6.31** | Significant | 0.05 | **6.70** | Significant | 0.11 | **3.89** | Significant | 0.10 |
| | 74 C12 364 | **5.11** | Significant | 0.00 | **8.37** | Significant | 0.11 | **2.87** | Significant | 0.00 |
| | 74 C12 554 | **4.97** | Significant | 0.06 | **7.99** | Significant | 0.11 | **3.63** | Significant | 0.00 |
| | 46 E8 132 | **5.80** | Significant | 0.07 | **6.67** | Significant | 0.07 | **3.36** | Significant | 0.00 |
| | 46 E8 322 | **5.27** | Significant | 0.11 | **11.20** | Significant | 0.06 | **3.85** | Significant | 0.05 |
| | 173 C3 3416 | **4.80** | Significant | 0.09 | **6.09** | Significant | 0.07 | **2.73** | Significant | 0.00 |
| | 173 C3 3606 | **4.73** | Significant | 0.07 | **6.22** | Significant | 0.07 | **2.79** | Significant | 0.00 |
| | **Average** | **5.47** | | | **7.52** | | | **3.37** | | |
| | **St. Dev.** | **0.75** | | | **1.69** | | | **0.50** | | |
| **6 H1** | 6 H1 9409 | **5.06** | Significant | 0.02 | **3.76** | Significant | 0.00 | **2.60** | Significant | 0.00 |
| **Predicted protein with signal or transit peptide** | 6 H1 9599 | **5.84** | Significant | 0.06 | **3.75** | Significant | 0.00 | **2.54** | Significant | 0.00 |
| | **Average** | **5.45** | | | **3.76** | | | **2.57** | | |
| | **St. Dev.** | **0.55** | | | **0.00** | | | **0.04** | | |
| **73 D12** | 73 D12 725 | **3.37** | Significant | 0.00 | **4.03** | Significant | 0.00 | **1.68** | Significant | 1.26 |
| **Ps-n specific, no hits in NR or Swissprot** | 73 D12 915 | **3.53** | Significant | 0.00 | **3.99** | Significant | 0.00 | **1.58** | Significant | 5.20 |
| | **Average** | **3.45** | | | **4.01** | | | **1.63** | | |
| | **St. Dev.** | **0.11** | | | **0.03** | | | **0.07** | | |
| **46 A5** | 46 A5 8794 | **4.36** | Significant | 0.04 | **4.53** | Significant | 0.11 | **1.71** | Significant | 0.07 |
| **Ps-n specific, no hits in NR or Swissprot** | 46 A5 8984 | **4.36** | Significant | 0.04 | **4.68** | Significant | 0.11 | **1.74** | Significant | 0.10 |
| | **Average** | **4.36** | | | **4.61** | | | **1.73** | | |
| | **St. Dev.** | **0.00** | | | **0.11** | | | **0.02** | | |
| **17 F11** | 17 F11 5471 | **5.30** | Significant | 0.11 | **5.42** | Significant | 0.09 | **2.03** | Significant | 0.00 |
| **Predicted protein** | 17 F11 5661 | **5.55** | Significant | 0.11 | **6.62** | Significant | 0.11 | **2.11** | Significant | 0.00 |
| | **Average** | **5.42** | | | **6.02** | | | **2.07** | | |
| | **St. Dev.** | **0.18** | | | **0.85** | | | **0.06** | | |
| **PSN1428** | 7 G2 6160 | **2.38** | NS | 0.00 | **2.04** | NS | 0.00 | **2.10** | Significant | 0.00 |
| **NAD-specific glutamate dehydrogenase** | 7 G2 6350 | **2.46** | NS | 0.00 | **2.02** | NS | 0.00 | **2.16** | Significant | 0.00 |
| | 55 G10 7626 | **1.87** | NS | 0.00 | **1.67** | NS | 0.00 | **1.57** | Significant | 16.50 |
| | 55 G10 7816 | **2.18** | NS | 0.00 | **1.69** | NS | 0.00 | **1.43** | NS | 23.01 |
| | **Average** | **2.22** | | | **1.86** | | | **1.81** | | |
| | **St. Dev.** | **0.26** | | | **0.20** | | | **0.37** | | |

| DOWN-REGULATED TRANSCRIPTS | Clone | Spot I.D. | Stationary versus exponential Growth Phase | | | | | | | | |
|---|---|---|---|---|---|---|---|---|---|---|---|
| | | | Non-axenic Expt. 1, Fold-change | Overall FDR | LFDR (%) | Non-axenic Expt. 2, Fold-change | Overall FDR | LFDR (%) | Axenic Expt., Fold-change | Overall FDR | LFDR (%) |
| **PSN0100** | 179 E10 | 7344 | **0.35** | Significant | 0.02 | **0.37** | Significant | 0.00 | **0.38** | Significant | 0.00 |
| **Pyrophosphate-dependent phosphofructokinase** | 179 E10 | 7534 | **0.35** | Significant | 0.03 | **0.37** | Significant | 0.00 | **0.38** | Significant | 0.00 |
| | 74 B1 | 9779 | **0.34** | Significant | 0.06 | **0.30** | Significant | 0.00 | **0.39** | Significant | 0.00 |
| | 74 B1 | 9969 | **0.34** | Significant | 0.05 | **0.28** | Significant | 0.00 | **0.39** | Significant | 0.00 |
| | **Average** | | **0.34** | | | **0.33** | | | **0.39** | | |
| | **St. Dev.** | | **0.01** | | | **0.05** | | | **0.01** | | |
| **PSN0060** | 17 E1 | 10517 | **0.20** | Significant | 0.00 | **0.17** | Significant | 0.11 | **0.45** | Significant | 0.00 |
| **Predicted protein with signal or transit peptide** | 17 E1 | 10707 | **0.21** | Significant | 0.00 | **0.16** | Significant | 0.10 | **0.46** | Significant | 0.00 |
| | 183 D10 | 8452 | **0.19** | Significant | 0.00 | **0.15** | Significant | 0.10 | **0.38** | Significant | 0.00 |
| | 183 D10 | 8642 | **0.18** | Significant | 0.00 | **0.12** | Significant | 0.05 | **0.38** | Significant | 0.00 |
| | 185 D2 | 6613 | **0.18** | Significant | 0.00 | **0.15** | Significant | 0.10 | **0.47** | Significant | 0.00 |
| | 185 D2 | 6803 | **0.18** | Significant | 0.00 | **0.15** | Significant | 0.10 | **0.48** | Significant | 0.00 |
| | 25 B4 | 1195 | **0.18** | Significant | 0.03 | **0.15** | Significant | 0.08 | **0.49** | Significant | 0.00 |
| | 25 B4 | 1385 | **0.18** | Significant | 0.01 | **0.11** | Significant | 0.06 | **0.45** | Significant | 0.00 |
| | 46 C2 | 5903 | **0.24** | Significant | 0.06 | **0.20** | Significant | 0.11 | **0.44** | Significant | 0.00 |
| | 46 C2 | 6093 | **0.23** | Significant | 0.06 | **0.21** | Significant | 0.11 | **0.45** | Significant | 0.00 |
| | **Average** | | **0.20** | | | **0.16** | | | **0.44** | | |
| | **St. Dev.** | | **0.02** | | | **0.03** | | | **0.04** | | |
| **PSN0048** | 137 D5 | 10869 | **0.30** | Significant | 0.10 | **0.21** | Significant | 0.11 | **0.52** | Significant | 0.00 |
| **Predicted protein with signal or transit peptide** | 137 D5 | 11059 | **0.32** | Significant | 0.00 | **0.21** | Significant | 0.11 | **0.54** | Significant | 1.86 |
| | 166 G9 | 8723 | **0.53** | NS | 0.00 | **0.34** | Significant | 0.00 | **0.60** | Significant | 7.53 |
| | 166 G9 | 8913 | **0.49** | NS | 0.00 | **0.36** | Significant | 0.00 | **0.60** | Significant | 8.28 |
| | 180 A9 | 10537 | **0.31** | Significant | 0.06 | **0.27** | Significant | 0.00 | **0.51** | Significant | 0.00 |
| | 180 A9 | 10727 | **0.32** | Significant | 0.07 | **0.24** | Significant | 0.00 | **0.51** | Significant | 0.00 |
| | 57 F9 | 10886 | **0.30** | Significant | 0.11 | **0.19** | Significant | 0.11 | **0.45** | Significant | 0.00 |
| | 57 F9 | 11076 | **0.30** | Significant | 0.11 | **0.22** | Significant | 0.00 | **0.45** | Significant | 0.00 |
| | 76 F12 | 2543 | **0.35** | Significant | 0.00 | **0.22** | Significant | 0.10 | **0.59** | Significant | 5.28 |
| | 76 F12 | 2733 | **0.34** | Significant | 0.00 | **0.23** | Significant | 0.06 | **0.59** | Significant | 6.44 |
| | **Average** | | **0.36** | | | **0.25** | | | **0.54** | | |
| | **St. Dev.** | | **0.08** | | | **0.06** | | | **0.06** | | |
| **PSN0080** | 176 G1 | 9114 | **0.32** | Significant | 0.07 | **0.34** | Significant | 0.00 | **0.65** | Significant | 14.15 |
| **Predicted protein with mitochondrial transit peptide** | 176 G1 | 9304 | **0.33** | Significant | 0.08 | **0.33** | Significant | 0.00 | **0.67** | Significant | 17.08 |
| | 179 C5 | 10227 | **0.34** | Significant | 0.02 | **0.30** | Significant | 0.00 | **0.51** | Significant | 0.00 |
| | 179 C5 | 10417 | **0.33** | Significant | 0.04 | **0.27** | Significant | 0.00 | **0.52** | Significant | 0.00 |
| | 51 G8 | 1605 | **0.30** | Significant | 0.08 | **0.30** | Significant | 0.00 | **0.54** | Significant | 0.87 |
| | 51 G8 | 1795 | **0.31** | Significant | 0.00 | **0.31** | Significant | 0.00 | **0.53** | Significant | 0.00 |
| | **Average** | | **0.32** | | | **0.31** | | | **0.57** | | |
| | **St. Dev.** | | **0.02** | | | **0.03** | | | **0.07** | | |
| **135 E4** | 135 E4 | 1973 | **0.17** | Significant | 0.02 | **0.20** | Significant | 0.07 | **0.46** | Significant | 0.00 |
| **Predicted protein with signal peptide** | 135 E4 | 2163 | **0.17** | Significant | 0.01 | **0.17** | Significant | 0.11 | **0.43** | Significant | 0.00 |
| | **Average** | | **0.17** | | | **0.19** | | | **0.45** | | |
| | **St. Dev.** | | **0.00** | | | **0.02** | | | **0.02** | | |
| **165 G9** | 165 G9 | 10554 | **0.39** | Significant | 0.00 | **0.36** | Significant | 0.00 | **0.60** | Significant | 5.92 |
| **Tetratricopeptide repeat protein** | 165 G9 | 10744 | **0.35** | Significant | 0.00 | **0.39** | Significant | 0.00 | **0.65** | Significant | 16.21 |
| | **Average** | | **0.37** | | | **0.37** | | | **0.62** | | |
| | **St. Dev.** | | **0.02** | | | **0.02** | | | **0.04** | | |
| **135H6** | 135 H6 | 8438 | **0.41** | NS | 0.00 | **0.23** | Significant | 0.10 | **0.59** | Significant | 6.65 |
| **Fucoxanthin-chlorophyll a-c binding protein, chloroplastic** | 135 H6 | 8628 | **0.41** | NS | 0.00 | **0.20** | Significant | 0.09 | **0.59** | Significant | 5.54 |
| | **Average** | | **0.41** | | | **0.22** | | | **0.59** | | |
| | **St. Dev.** | | **0.00** | | | **0.02** | | | **0.00** | | |

| REFERENCE GENES | Clone | Spot I.D. | Stationary versus exponential Growth Phase | | | | | | | | |
|---|---|---|---|---|---|---|---|---|---|---|---|
| | | | Non-axenic Expt. 1, Fold-change | Overall FDR | LFDR (%) | Non-axenic Expt. 2, Fold-change | Overall FDR | LFDR (%) | Axenic Expt., Fold-change | Overall FDR | LFDR (%) |
| **53B6** | 53 B6 | 6656 | **1.05** | NS | 76.16 | **1.01** | NS | 76.06 | **1.06** | NS | 79.69 |
| **JmjC** | 53 B6 | 6846 | **1.06** | NS | 74.67 | **1.08** | NS | 68.51 | **1.03** | NS | 80.89 |
| | **Average** | | **1.05** | | | **1.04** | | | **1.05** | | |
| | **St. Dev.** | | **0.01** | | | **0.05** | | | **0.03** | | |
| **45E3** | 45 E3 | 4354 | **1.11** | NS | 70.51 | **1.29** | NS | 42.45 | **1.32** | NS | 73.39 |
| **Dynein, heavy chain, cytosolic** | 45 E3 | 4544 | **1.08** | NS | 77.05 | **1.19** | NS | 54.29 | **1.40** | NS | 65.86 |
| | **Average** | | **1.10** | | | **1.24** | | | **1.36** | | |

| | | | | | | | | | | | |
|---|---|---|---|---|---|---|---|---|---|---|---|
| | **St. Dev.** | | **0.02** | | | **0.07** | | | **0.05** | | |
| **177F1** | 177 F1 | 11307 | **1.03** | NS | 77.52 | **0.91** | NS | 66.08 | **0.84** | NS | 56.50 |
| **Histone H3** | 177 F1 | 11497 | **1.06** | NS | 75.86 | **0.94** | NS | 72.10 | **0.78** | NS | 45.82 |
| | **Average** | | **1.05** | | | **0.93** | | | **0.81** | | |
| | **St. Dev.** | | **0.02** | | | **0.02** | | | **0.04** | | |
| **PSN0918** | 51 C2 | 7375 | **1.31** | NS | 20.90 | **1.32** | NS | 32.30 | **0.99** | NS | 80.85 |
| **Cylophilin** | 51 C2 | 7565 | **1.30** | NS | 24.42 | **1.26** | NS | 43.31 | **0.98** | NS | 80.54 |
| | **Average** | | **1.30** | | | **1.29** | | | **0.99** | | |
| | **St. Dev.** | | **0.00** | | | **0.04** | | | **0.01** | | |
| **PSN0001** | 136 A8 | 479 | **1.28** | NS | 28.42 | **1.21** | NS | 52.52 | **1.18** | NS | 67.76 |
| **EF-1α** | 136 A8 | 669 | **1.31** | NS | 24.05 | **1.19** | NS | 58.06 | **1.13** | NS | 74.36 |
| | 136 H5 | 9868 | **1.41** | NS | 5.75 | **1.12** | NS | 61.63 | **0.88** | NS | 65.59 |
| | 136 H5 | 10058 | **1.43** | NS | 3.42 | **1.09** | NS | 66.55 | **0.85** | NS | 58.52 |
| | 165 C8 | 1884 | **1.27** | NS | 28.59 | **1.23** | NS | 42.07 | **1.13** | NS | 76.96 |
| | 165 C8 | 2074 | **1.29** | NS | 30.06 | **1.21** | NS | 47.31 | **1.10** | NS | 78.81 |
| | 165 E1 | 10583 | **1.44** | NS | 6.68 | **1.19** | NS | 44.02 | **1.30** | NS | 47.79 |
| | 165 E1 | 10773 | **1.44** | NS | 12.83 | **1.23** | NS | 38.15 | **1.33** | NS | 44.16 |
| | 165 G7 | 4774 | **1.31** | NS | 16.67 | **1.22** | NS | 41.39 | **1.11** | NS | 76.26 |
| | 165 G7 | 4964 | **1.31** | NS | 15.28 | **1.23** | NS | 37.54 | **1.08** | NS | 78.70 |
| | 166 B10 | 6592 | **1.33** | NS | 15.57 | **1.06** | NS | 70.26 | **0.94** | NS | 77.05 |
| | 166 B10 | 6782 | **1.33** | NS | 14.96 | **1.08** | NS | 65.90 | **0.94** | NS | 77.01 |
| | 166 E11 | 2946 | **1.39** | NS | 18.25 | **1.18** | NS | 59.75 | **1.15** | NS | 78.49 |
| | 166 E11 | 3136 | **1.39** | NS | 18.41 | **1.11** | NS | 65.45 | **1.19** | NS | 74.98 |
| | 167 A6 | 5946 | **1.22** | NS | 31.36 | **1.21** | NS | 39.50 | **0.90** | NS | 71.96 |
| | 167 A6 | 6136 | **1.20** | NS | 39.84 | **1.22** | NS | 36.34 | **0.89** | NS | 69.47 |
| | 167 F10 | 6636 | **1.32** | NS | 18.07 | **1.20** | NS | 44.60 | **0.93** | NS | 75.16 |
| | 167 F10 | 6826 | **1.28** | NS | 22.55 | **1.19** | NS | 46.46 | **0.96** | NS | 79.00 |
| | 169 C3 | 4473 | **1.39** | NS | 7.83 | **1.24** | NS | 40.75 | **0.95** | NS | 78.19 |
| | 169 C3 | 4663 | **1.37** | NS | 7.97 | **1.18** | NS | 48.85 | **0.94** | NS | 77.65 |
| | 169 F1 | 10972 | **1.49** | NS | 1.94 | **1.14** | NS | 56.75 | **1.08** | NS | 76.93 |
| | 169 F1 | 11162 | **1.45** | NS | 4.25 | **1.21** | NS | 48.53 | **1.10** | NS | 77.62 |
| | 169 F6 | 8088 | **1.33** | NS | 13.38 | **1.15** | NS | 54.71 | **0.91** | NS | 70.53 |
| | 169 F6 | 8278 | **1.36** | NS | 8.36 | **1.17** | NS | 49.73 | **0.92** | NS | 72.71 |
| | 169 G5 | 10255 | **1.29** | NS | 20.74 | **1.15** | NS | 54.84 | **0.93** | NS | 77.97 |
| | 169 G5 | 10445 | **1.34** | NS | 10.42 | **1.20** | NS | 43.90 | **0.95** | NS | 79.66 |
| | 171 B5 | 11281 | **1.23** | NS | 38.21 | **0.90** | NS | 69.29 | **0.81** | NS | 53.94 |
| | 171 B5 | 11471 | **1.23** | NS | 35.38 | **0.92** | NS | 72.74 | **0.80** | NS | 50.41 |
| | 171 C11 | 4788 | **1.30** | NS | 17.56 | **1.21** | NS | 40.67 | **1.17** | NS | 64.70 |
| | 171 C11 | 4978 | **1.30** | NS | 18.40 | **1.23** | NS | 36.12 | **1.15** | NS | 70.55 |
| | 171 H1 | 11280 | **1.49** | NS | 2.56 | **1.14** | NS | 60.50 | **1.00** | NS | 80.79 |
| | 171 H1 | 11470 | **1.48** | NS | 2.07 | **1.16** | NS | 58.43 | **0.95** | NS | 78.61 |
| | 174 H1 | 9529 | **1.44** | NS | 3.10 | **1.11** | NS | 62.47 | **1.05** | NS | 79.74 |
| | 174 H1 | 9719 | **1.46** | NS | 2.96 | **1.13** | NS | 58.56 | **1.06** | NS | 79.41 |
| | 174 H11 | 3761 | **1.41** | NS | 10.76 | **1.13** | NS | 63.02 | **1.02** | NS | 80.93 |
| | 174 H11 | 3951 | **1.54** | NS | 4.12 | **1.12** | NS | 61.81 | **1.03** | NS | 80.66 |
| | 174 H7 | 3757 | **1.33** | NS | 22.89 | **1.22** | NS | 45.18 | **0.97** | NS | 78.64 |
| | 174 H7 | 3947 | **1.39** | NS | 13.49 | **1.25** | NS | 33.14 | **0.98** | NS | 79.87 |
| | 177 C12 | 1928 | **1.25** | NS | 37.97 | **1.19** | NS | 53.22 | **1.16** | NS | 65.71 |
| | 177 C12 | 2118 | **1.28** | NS | 31.26 | **1.18** | NS | 54.53 | **1.09** | NS | 76.57 |
| | 177 E7 | 4813 | **1.29** | NS | 21.37 | **1.21** | NS | 42.11 | **0.92** | NS | 75.82 |
| | 177 E7 | 5003 | **1.29** | NS | 17.22 | **1.23** | NS | 35.25 | **0.96** | NS | 78.56 |
| | 177 H12 | 2652 | **1.36** | NS | 10.30 | **1.10** | NS | 65.94 | **1.00** | NS | 80.39 |
| | 177 H12 | 2842 | **1.38** | NS | 9.30 | **1.16** | NS | 56.00 | **0.96** | NS | 78.41 |
| | 179 F4 | 2282 | **1.27** | NS | 31.26 | **1.22** | NS | 48.23 | **1.11** | NS | 73.63 |
| | 179 F4 | 2472 | **1.31** | NS | 26.14 | **1.21** | NS | 46.86 | **1.09** | NS | 75.62 |
| | 183 B10 | 8451 | **1.44** | NS | 5.38 | **1.15** | NS | 56.52 | **0.97** | NS | 79.54 |
| | 183 B10 | 8641 | **1.46** | NS | 2.87 | **1.16** | NS | 54.74 | **0.97** | NS | 78.87 |
| | 183 C12 | 1954 | **1.32** | NS | 30.84 | **1.30** | NS | 34.30 | **1.30** | NS | 40.31 |
| | 183 C12 | 2144 | **1.36** | NS | 25.35 | **1.27** | NS | 35.70 | **1.27** | NS | 47.92 |
| | 185 G6 | 5897 | **1.38** | NS | 7.54 | **1.15** | NS | 56.83 | **0.99** | NS | 80.77 |
| | 185 G6 | 6087 | **1.37** | NS | 10.09 | **1.20** | NS | 43.94 | **0.92** | NS | 76.97 |
| | 25 F8 | 1163 | **1.44** | NS | 8.67 | **1.17** | NS | 55.90 | **1.24** | NS | 57.69 |
| | 25 F8 | 1353 | **1.40** | NS | 9.02 | **1.14** | NS | 60.88 | **1.13** | NS | 74.07 |
| | 30 B3 | 3682 | **1.43** | NS | 8.61 | **1.17** | NS | 56.48 | **1.07** | NS | 78.08 |
| | 30 B3 | 3872 | **1.54** | NS | 5.21 | **1.13** | NS | 63.39 | **1.09** | NS | 75.66 |
| | 30 C3 | 2961 | **1.34** | NS | 21.15 | **1.18** | NS | 55.78 | **1.17** | NS | 71.89 |
| | 30 C3 | 3151 | **1.36** | NS | 19.26 | **1.12** | NS | 64.35 | **1.12** | NS | 76.57 |
| | 30 D5 | 9425 | **1.37** | NS | 10.55 | **1.23** | NS | 40.02 | **1.10** | NS | 75.65 |
| | 30 D5 | 9615 | **1.38** | NS | 10.14 | **1.22** | NS | 36.52 | **1.11** | NS | 75.96 |

| | | | | | | | | | | | |
|---|---|---|---|---|---|---|---|---|---|---|---|
| | 30 G10 | 5821 | **1.44** | NS | 5.36 | **1.14** | NS | 60.40 | **1.05** | NS | 80.62 |
| | 30 G10 | 6011 | **1.43** | NS | 5.96 | **1.11** | NS | 65.06 | **0.99** | NS | 80.68 |
| | 30 H4 | 797 | **1.34** | NS | 20.66 | **1.09** | NS | 70.74 | **1.20** | NS | 63.08 |
| | 30 H4 | 987 | **1.30** | NS | 22.00 | **1.08** | NS | 70.96 | **1.09** | NS | 78.36 |
| | 45 A2 | 7240 | **1.59** | NS | 2.34 | **1.18** | NS | 51.41 | **1.12** | NS | 76.81 |
| | 45 A2 | 7430 | **1.59** | NS | 2.59 | **1.10** | NS | 66.01 | **1.10** | NS | 78.90 |
| | 45 A9 | 10136 | **1.32** | NS | 26.69 | **1.21** | NS | 45.18 | **1.20** | NS | 70.31 |
| | 45 A9 | 10326 | **1.37** | NS | 13.08 | **1.17** | NS | 48.36 | **1.34** | NS | 50.51 |
| | 45 B5 | 10854 | **1.47** | NS | 3.27 | **1.13** | NS | 58.43 | **1.18** | NS | 68.37 |
| | 45 B5 | 11044 | **1.47** | NS | 4.17 | **1.13** | NS | 56.60 | **1.21** | NS | 64.82 |
| | 45 G1 | 10131 | **1.26** | NS | 32.55 | **1.17** | NS | 48.15 | **0.92** | NS | 76.23 |
| | 45 G1 | 10321 | **1.30** | NS | 17.43 | **1.13** | NS | 58.80 | **1.01** | NS | 80.98 |
| | 47 H2 | 8045 | **1.48** | NS | 6.48 | **1.16** | NS | 52.71 | **1.10** | NS | 76.49 |
| | 47 H2 | 8235 | **1.43** | NS | 4.42 | **1.15** | NS | 54.91 | **1.06** | NS | 79.02 |
| | 50 A11 | 4414 | **1.46** | NS | 5.82 | **1.31** | NS | 35.40 | **1.51** | NS | 43.61 |
| | 50 A11 | 4604 | **1.50** | NS | 2.23 | **1.25** | NS | 51.08 | **1.53** | NS | 50.09 |
| | 50 A8 | 1522 | **1.75** | NS | 7.03 | **1.13** | NS | 66.34 | **1.55** | NS | 31.45 |
| | 50 A8 | 1712 | **1.43** | NS | 15.96 | **1.19** | NS | 61.30 | **1.57** | Significant | 26.28 |
| | 50 C7 | 4411 | **1.35** | NS | 14.00 | **1.30** | NS | 23.16 | **1.60** | NS | 36.11 |
| | 50 C7 | 4601 | **1.34** | NS | 15.90 | **1.32** | NS | 27.77 | **1.55** | NS | 43.77 |
| | 50 G7 | 4413 | **1.44** | NS | 4.39 | **1.23** | NS | 45.72 | **1.22** | NS | 70.28 |
| | 50 G7 | 4603 | **1.47** | NS | 2.04 | **1.19** | NS | 57.37 | **1.24** | NS | 69.28 |
| | 51 H6 | 8103 | **1.39** | NS | 5.87 | **1.15** | NS | 54.98 | **0.96** | NS | 78.57 |
| | 51 H6 | 8293 | **1.40** | NS | 5.49 | **1.19** | NS | 47.69 | **0.98** | NS | 79.82 |
| | 52 B3 | 3656 | **1.40** | NS | 14.87 | **1.19** | NS | 55.42 | **1.07** | NS | 78.46 |
| | 52 B3 | 3846 | **1.40** | NS | 14.51 | **1.18** | NS | 57.17 | **1.10** | NS | 75.03 |
| | 52 D2 | 6545 | **1.56** | NS | 1.59 | **1.28** | NS | 31.07 | **1.48** | NS | 23.42 |
| | 52 D2 | 6735 | **1.53** | NS | 6.95 | **1.26** | NS | 32.04 | **1.41** | NS | 28.33 |
| | 53 A9 | 8826 | **1.36** | NS | 10.86 | **1.29** | NS | 26.25 | **1.12** | NS | 74.91 |
| | 53 A9 | 9016 | **1.29** | NS | 26.94 | **1.28** | NS | 29.55 | **1.04** | NS | 80.13 |
| | 53 B2 | 6652 | **1.40** | NS | 9.03 | **1.23** | NS | 41.70 | **1.10** | NS | 75.13 |
| | 53 B2 | 6842 | **1.42** | NS | 10.78 | **1.24** | NS | 43.53 | **1.12** | NS | 72.47 |
| | 53 D3 | 3765 | **1.29** | NS | 27.08 | **1.22** | NS | 47.42 | **1.04** | NS | 80.23 |
| | 53 D3 | 3955 | **1.26** | NS | 28.01 | **1.21** | NS | 48.64 | **1.01** | NS | 80.98 |
| | 54.1 D10 | 7022 | **1.39** | NS | 4.83 | **1.13** | NS | 58.00 | **0.92** | NS | 71.89 |
| | 54.1 D10 | 7212 | **1.42** | NS | 3.63 | **1.09** | NS | 66.15 | **0.92** | NS | 71.56 |
| | 54.1 G1 | 9182 | **1.28** | NS | 15.46 | **1.34** | NS | 15.57 | **1.02** | NS | 80.89 |
| | 54.1 G1 | 9372 | **1.25** | NS | 20.69 | **1.31** | NS | 19.22 | **1.02** | NS | 80.88 |
| | 54.2 D10 | 8011 | **1.45** | NS | 4.72 | **1.11** | NS | 63.24 | **0.98** | NS | 80.46 |
| | 54.2 D10 | 8201 | **1.46** | NS | 3.00 | **1.11** | NS | 60.79 | **1.01** | NS | 80.95 |
| | 54.2 G1 | 10171 | **1.10** | NS | 75.19 | **1.12** | NS | 57.46 | **1.03** | NS | 80.65 |
| | 54.2 G1 | 10361 | **0.96** | NS | 76.99 | **1.12** | NS | 59.51 | **0.93** | NS | 75.99 |
| | 55 D12 | 2570 | **1.45** | NS | 7.19 | **1.23** | NS | 42.55 | **1.23** | NS | 50.07 |
| | 55 D12 | 2760 | **1.44** | NS | 4.36 | **1.21** | NS | 45.57 | **1.22** | NS | 59.85 |
| | 55 E9 | 10513 | **1.26** | NS | 26.36 | **1.25** | NS | 32.48 | **1.04** | NS | 80.34 |
| | 55 E9 | 10703 | **1.26** | NS | 22.84 | **1.26** | NS | 29.20 | **1.07** | NS | 79.05 |
| | 55 G11 | 4738 | **1.31** | NS | 17.62 | **1.22** | NS | 39.09 | **1.09** | NS | 77.78 |
| | 55 G11 | 4928 | **1.32** | NS | 15.98 | **1.21** | NS | 40.13 | **1.09** | NS | 76.50 |
| | 55 G12 | 1850 | **1.32** | NS | 24.33 | **1.24** | NS | 40.62 | **1.38** | NS | 31.26 |
| | 55 G12 | 2040 | **1.29** | NS | 27.88 | **1.24** | NS | 40.71 | **1.49** | NS | 16.48 |
| | 55 H4 | 2602 | **1.43** | NS | 7.59 | **1.16** | NS | 51.45 | **1.20** | NS | 67.96 |
| | 55 H4 | 2792 | **1.39** | NS | 8.98 | **1.21** | NS | 38.01 | **1.17** | NS | 72.84 |
| | 57 C5 | 10159 | **1.34** | NS | 16.37 | **1.17** | NS | 50.50 | **1.04** | NS | 80.80 |
| | 57 C5 | 10349 | **1.39** | NS | 3.79 | **1.19** | NS | 48.39 | **1.08** | NS | 78.57 |
| | 7 A10 | 6165 | **1.28** | NS | 26.33 | **1.25** | NS | 32.85 | **1.02** | NS | 80.87 |
| | 7 A10 | 6355 | **1.29** | NS | 28.98 | **1.31** | NS | 29.30 | **1.01** | NS | 80.96 |
| | 7 E4 | 383 | **1.27** | NS | 32.77 | **1.23** | NS | 47.83 | **1.17** | NS | 69.04 |
| | 7 E4 | 573 | **1.25** | NS | 39.19 | **1.25** | NS | 42.49 | **1.13** | NS | 76.36 |
| | 7 H12 | 1114 | **1.29** | NS | 26.48 | **1.28** | NS | 33.45 | **1.03** | NS | 80.97 |
| | 7 H12 | 1304 | **1.30** | NS | 19.83 | **1.25** | NS | 37.19 | **0.93** | NS | 74.71 |
| | **Average** | | **1.37** | | | **1.19** | | | **1.10** | | |
| | **St. Dev.** | | **0.10** | | | **0.07** | | | **0.17** | | |
| **PSN0547** | 183 D11 | 5564 | **0.95** | NS | 75.80 | **0.86** | NS | 54.96 | **1.23** | NS | 56.80 |
| **Phosphoglycerate kinase** | 183 D11 | 5754 | **0.99** | NS | 78.30 | **0.79** | NS | 35.08 | **1.23** | NS | 53.85 |
| | 30 E2 | 5850 | **0.87** | NS | 58.32 | **0.74** | NS | 17.66 | **1.29** | NS | 55.07 |
| | 30 E2 | 6040 | **0.80** | NS | 38.09 | **0.75** | NS | 28.33 | **1.28** | NS | 55.25 |
| | **Average** | | **0.90** | | | **0.78** | | | **1.26** | | |
| | **St. Dev.** | | **0.08** | | | **0.05** | | | **0.03** | | |
| **PSN1327** | 167 D4 | 889 | **0.98** | NS | 77.68 | **1.07** | NS | 75.09 | **1.27** | NS | 69.84 |

| | | | | | | | | | | | |
|---|---|---|---|---|---|---|---|---|---|---|---|
| **eIF-2** | 167 D4 | 1079 | 1.02 | NS | 78.13 | 1.06 | NS | 75.86 | 1.22 | NS | 71.08 |
| | **Average** | | 1.00 | | | 1.07 | | | 1.24 | | |
| | **St. Dev.** | | 0.03 | | | 0.00 | | | 0.03 | | |
| **PSN0332** | 177 C10 | 7704 | 1.14 | NS | 59.34 | 1.32 | NS | 39.14 | 1.03 | NS | 80.81 |
| **ATPase with AAA domain** | 177 C10 | 7894 | 1.07 | NS | 71.21 | 1.29 | NS | 41.61 | 1.05 | NS | 80.22 |
| | 52 G4 | 49 | 1.12 | NS | 76.30 | 1.25 | NS | 46.44 | 1.33 | NS | 72.93 |
| | 52 G4 | 239 | 1.19 | NS | 74.64 | 1.31 | NS | 39.72 | 1.35 | NS | 73.90 |
| | **Average** | | 1.13 | | | 1.29 | | | 1.19 | | |
| | **St. Dev.** | | 0.05 | | | 0.03 | | | 0.17 | | |
| **PSN0032** | 177 B8 | 2645 | 0.72 | NS | 8.13 | 0.62 | NS | 1.90 | 0.47 | Significant | 0.00 |
| **Ubiquitin** | 177 B8 | 2835 | 0.71 | NS | 6.52 | 0.62 | NS | 0.97 | 0.47 | Significant | 0.00 |
| | 183 B2 | 8443 | 0.81 | NS | 35.75 | 0.74 | NS | 31.61 | 0.45 | Significant | 0.00 |
| | 183 B2 | 8633 | 0.83 | NS | 39.10 | 0.73 | NS | 25.54 | 0.44 | Significant | 0.00 |
| | 47 H6 | 8049 | 0.75 | NS | 16.82 | 0.68 | NS | 9.46 | 0.43 | Significant | 0.00 |
| | 47 H6 | 8239 | 0.75 | NS | 16.86 | 0.67 | NS | 7.98 | 0.44 | Significant | 0.00 |
| | 55 F4 | 2601 | 0.70 | NS | 6.24 | 0.62 | NS | 1.21 | 0.42 | Significant | 0.00 |
| | 55 F4 | 2791 | 0.70 | NS | 5.09 | 0.66 | NS | 5.87 | 0.42 | Significant | 0.00 |
| | 57 F2 | 7990 | 0.73 | NS | 10.41 | 0.62 | NS | 1.55 | 0.49 | Significant | 0.00 |
| | 57 F2 | 8180 | 0.75 | NS | 17.50 | 0.64 | NS | 3.91 | 0.49 | Significant | 0.00 |
| | 7 E6 | 6163 | 0.75 | NS | 16.28 | 0.80 | NS | 39.36 | 0.43 | Significant | 0.00 |
| | 7 E6 | 6353 | 0.76 | NS | 19.72 | 0.80 | NS | 42.29 | 0.42 | Significant | 0.00 |
| | 75 G9 | 10125 | 0.68 | NS | 3.89 | 0.68 | NS | 11.29 | 0.41 | Significant | 0.00 |
| | 75 G9 | 10315 | 0.70 | NS | 8.13 | 0.67 | NS | 6.47 | 0.42 | Significant | 0.00 |
| | **Average** | | 0.74 | | | 0.68 | | | 0.44 | | |
| | **St. Dev.** | | 0.04 | | | 0.06 | | | 0.03 | | |
| **PSN1138** | 136 B6 | 6977 | 0.82 | NS | 45.44 | 0.83 | NS | 51.80 | 1.95 | Significant | 0.00 |
| **Glyceraldehyde 3-phosphate dehydrogenase** | 136 B6 | 7167 | 0.86 | NS | 55.31 | 0.87 | NS | 60.25 | 1.96 | Significant | 0.00 |
| | 165 C6 | 7660 | 1.05 | NS | 75.91 | 1.10 | NS | 69.04 | 1.27 | NS | 46.29 |
| | 165 C6 | 7850 | 1.03 | NS | 77.77 | 1.09 | NS | 68.76 | 1.28 | NS | 45.28 |
| | 174 D10 | 6647 | 0.89 | NS | 61.44 | 0.89 | NS | 65.12 | 1.91 | Significant | 0.00 |
| | 174 D10 | 6837 | 0.87 | NS | 57.22 | 0.88 | NS | 61.91 | 1.86 | Significant | 0.00 |
| | 174 G10 | 5927 | 0.82 | NS | 39.07 | 0.96 | NS | 73.28 | 1.77 | Significant | 0.21 |
| | 174 G10 | 6117 | 0.81 | NS | 35.28 | 0.94 | NS | 71.14 | 1.85 | Significant | 0.00 |
| | 185 C8 | 119 | 0.82 | NS | 46.80 | 0.90 | NS | 65.13 | 1.64 | Significant | 10.06 |
| | 185 C8 | 309 | 0.85 | NS | 51.44 | 0.85 | NS | 56.26 | 1.77 | Significant | 0.00 |
| | 45 H1 | 10853 | 0.83 | NS | 39.39 | 0.90 | NS | 68.24 | 1.76 | Significant | 0.70 |
| | 45 H1 | 11043 | 0.83 | NS | 37.98 | 0.85 | NS | 56.69 | 1.77 | Significant | 0.08 |
| | **Average** | | 0.87 | | | 0.92 | | | 1.73 | | |
| | **St. Dev.** | | 0.08 | | | 0.09 | | | 0.23 | | |
| **PSN0019** | 30 H2 | 6573 | 0.42 | NS | 0.00 | 0.43 | NS | 0.00 | 0.58 | Significant | 2.55 |
| **Actin** | 30 H2 | 6763 | 0.43 | NS | 0.00 | 0.43 | NS | 0.00 | 0.59 | Significant | 3.64 |
| | 161 E1 | 8778 | 0.38 | Significant | 0.00 | 0.48 | NS | 0.00 | 0.59 | Significant | 3.78 |
| | 161 E1 | 8968 | 0.38 | Significant | 0.00 | 0.50 | NS | 0.00 | 0.60 | Significant | 6.22 |
| | 136 F3 | 4087 | 0.44 | NS | 0.00 | 0.43 | NS | 0.00 | 0.45 | Significant | 0.00 |
| | 136 F3 | 4277 | 0.45 | NS | 0.00 | 0.43 | NS | 0.00 | 0.45 | Significant | 0.00 |
| | 169 B11 | 5202 | 0.46 | NS | 0.00 | 0.46 | NS | 0.00 | 0.46 | Significant | 0.00 |
| | 169 B11 | 5392 | 0.46 | NS | 0.00 | 0.46 | NS | 0.00 | 0.44 | Significant | 0.00 |
| | 170 H5 | 9814 | 0.44 | NS | 0.00 | 0.44 | NS | 0.00 | 0.47 | Significant | 0.00 |
| | 170 H5 | 10004 | 0.43 | NS | 0.00 | 0.44 | NS | 0.00 | 0.46 | Significant | 0.00 |
| | 183 D2 | 8444 | 0.45 | NS | 0.00 | 0.44 | NS | 0.00 | 0.50 | Significant | 0.00 |
| | 183 D2 | 8634 | 0.44 | NS | 0.00 | 0.44 | NS | 0.00 | 0.51 | Significant | 0.00 |
| | 186 A12 | 509 | 0.43 | NS | 0.00 | 0.52 | NS | 0.00 | 0.59 | Significant | 3.87 |
| | 186 A12 | 699 | 0.43 | NS | 0.00 | 0.52 | NS | 0.00 | 0.57 | Significant | 2.03 |
| | 186 B12 | 1231 | 1.77 | NS | 0.00 | 1.49 | NS | 8.57 | 1.48 | NS | 40.18 |
| | 186 B12 | 1421 | 1.88 | NS | 0.00 | 1.46 | NS | 9.00 | 1.56 | Significant | 17.43 |
| | 57 H7 | 5107 | 0.59 | NS | 0.00 | 0.58 | NS | 0.00 | 0.66 | Significant | 13.25 |
| | 57 H7 | 5297 | 0.59 | NS | 0.00 | 0.57 | NS | 0.00 | 0.66 | Significant | 12.27 |
| | 186 F1 | 9889 | 0.43 | NS | 0.00 | 0.41 | NS | 0.00 | 0.50 | Significant | 0.00 |
| | 186 F1 | 10079 | 0.43 | NS | 0.00 | 0.42 | NS | 0.00 | 0.48 | Significant | 0.00 |
| | 45 F4 | 2188 | 0.42 | NS | 0.00 | 0.44 | NS | 0.00 | 0.62 | Significant | 5.16 |
| | 45 F4 | 2378 | 0.40 | NS | 0.00 | 0.49 | NS | 0.00 | 0.62 | Significant | 5.06 |
| | 51 A8 | 1602 | 0.40 | NS | 0.00 | 0.47 | NS | 0.00 | 0.57 | Significant | 2.37 |
| | 51 A8 | 1792 | 0.41 | NS | 0.00 | 0.48 | NS | 0.00 | 0.58 | Significant | 3.82 |
| | 174 G12 | 151 | 0.39 | Significant | 0.00 | 0.46 | NS | 0.00 | 0.60 | Significant | 7.05 |
| | 174 G12 | 341 | 0.39 | Significant | 0.00 | 0.47 | NS | 0.00 | 0.58 | Significant | 4.08 |
| | **Average** | | 0.54 | | | 0.54 | | | 0.62 | | |
| | **St. Dev.** | | 0.38 | | | 0.28 | | | 0.27 | | |
