# Supplementary material for: Gene expression studies for the analysis of domoic acid production in the marine diatom Pseudo-nitzschia multiseries
Source: BMC Mol Biol. 2013 Nov 1;14:25. doi: 10.1186/1471-2199-14-25 (PMC3832940; doi:10.1186/1471-2199-14-25)
Supplement: Additional file 7 — RT-qPCR statistical results. [file 1471-2199-14-25-S7.docx]

135H6, T3 to T10:

**General Linear Model: Cq Prime versus Biounit, Time Point**

Factor Type Levels Values

Biounit random 3 1, 2, 3

Time Point fixed 8 3, 4, 5, 6, 7, 8, 9, 10

Analysis of Variance for Cq Prime, using Adjusted SS for Tests

Source DF Seq SS Adj SS Adj MS F P

Biounit 2 1.3704 0.5588 0.2794 1.20 0.334

Time Point 7 13.5313 13.5313 1.9330 8.27 0.001

Error 13 3.0386 3.0386 0.2337

Total 22 17.9403

S = 0.483467 R-Sq = 83.06% R-Sq(adj) = 71.34%

Term Coef SE Coef T P

Constant -1.6834 0.1022 -16.48 0.000

Biounit

1 -0.1629 0.1492 -1.09 0.295

2 0.2138 0.1420 1.51 0.156

Time Point

3 1.6019 0.3198 5.01 0.000

4 1.1171 0.2624 4.26 0.001

5 -0.6759 0.2624 -2.58 0.023

6 -0.8177 0.2624 -3.12 0.008

7 -0.6003 0.2624 -2.29 0.040

8 -0.3996 0.2624 -1.52 0.152

9 -0.0744 0.2624 -0.28 0.781

Unusual Observations for Cq Prime

Obs Cq Prime Fit SE Fit Residual St Resid

3 -1.67444 -0.72915 0.31650 -0.94529 -2.59 R

R denotes an observation with a large standardized residual.

Expected Mean Squares, using Adjusted SS

Expected Mean Square

Source for Each Term

1 Biounit (3) + 7.5000 (1)

2 Time Point (3) + Q[2]

3 Error (3)

Error Terms for Tests, using Adjusted SS

Synthesis

Source Error DF Error MS of Error MS

1 Biounit 13.00 0.2337 (3)

2 Time Point 13.00 0.2337 (3)

Variance Components, using Adjusted SS

Estimated

Source Value

Biounit 0.00609

Error 0.23374

Grouping Information Using Bonferroni Method and 95.0% Confidence

Time

Point N Mean Grouping

3 2 -0.081 A

4 3 -0.566 A B

9 3 -1.758 A B C

10 3 -1.834 B C

8 3 -2.083 B C

7 3 -2.284 C

5 3 -2.359 C

6 3 -2.501 C

Means that do not share a letter are significantly different.

Bonferroni 95.0% Simultaneous Confidence Intervals

Response Variable Cq Prime

All Pairwise Comparisons among Levels of Time Point

Time Point = 3 subtracted from:

Time

Point Lower Center Upper -+---------+---------+---------+-----

4 -2.236 -0.485 1.2661 (--------*-------)

5 -4.029 -2.278 -0.5269 (--------*-------)

6 -4.171 -2.420 -0.6687 (--------*--------)

7 -3.953 -2.202 -0.4513 (--------*--------)

8 -3.752 -2.002 -0.2506 (--------*--------)

9 -3.427 -1.676 0.0746 (--------*-------)

10 -3.504 -1.753 -0.0021 (--------*--------)

-+---------+---------+---------+-----

-4.0 -2.0 0.0 2.0

Time Point = 4 subtracted from:

Time

Point Lower Center Upper -+---------+---------+---------+-----

5 -3.337 -1.793 -0.2488 (-------*-------)

6 -3.479 -1.935 -0.3906 (------*-------)

7 -3.262 -1.717 -0.1732 (------*-------)

8 -3.061 -1.517 0.0275 (------*-------)

9 -2.736 -1.191 0.3527 (-------*-------)

10 -2.812 -1.268 0.2761 (-------*------)

-+---------+---------+---------+-----

-4.0 -2.0 0.0 2.0

Time Point = 5 subtracted from:

Time

Point Lower Center Upper -+---------+---------+---------+-----

6 -1.686 -0.1419 1.402 (------*-------)

7 -1.469 0.0756 1.620 (------*-------)

8 -1.268 0.2763 1.820 (------*-------)

9 -0.943 0.6015 2.146 (-------*-------)

10 -1.019 0.5248 2.069 (-------*------)

-+---------+---------+---------+-----

-4.0 -2.0 0.0 2.0

Time Point = 6 subtracted from:

Time

Point Lower Center Upper -+---------+---------+---------+-----

7 -1.327 0.2174 1.762 (-------*-------)

8 -1.126 0.4181 1.962 (-------*-------)

9 -0.801 0.7433 2.288 (-------*------)

10 -0.878 0.6667 2.211 (------*-------)

-+---------+---------+---------+-----

-4.0 -2.0 0.0 2.0

Time Point = 7 subtracted from:

Time

Point Lower Center Upper -+---------+---------+---------+-----

8 -1.343 0.2007 1.745 (-------*-------)

9 -1.018 0.5259 2.070 (-------*------)

10 -1.095 0.4493 1.993 (------*-------)

-+---------+---------+---------+-----

-4.0 -2.0 0.0 2.0

Time Point = 8 subtracted from:

Time

Point Lower Center Upper -+---------+---------+---------+-----

9 -1.219 0.3252 1.869 (-------*------)

10 -1.296 0.2485 1.793 (------*-------)

-+---------+---------+---------+-----

-4.0 -2.0 0.0 2.0

Time Point = 9 subtracted from:

Time

Point Lower Center Upper -+---------+---------+---------+-----

10 -1.621 -0.07667 1.468 (-------*------)

-+---------+---------+---------+-----

-4.0 -2.0 0.0 2.0

Bonferroni Simultaneous Tests

Response Variable Cq Prime

All Pairwise Comparisons among Levels of Time Point

Time Point = 3 subtracted from:

Time Difference SE of Adjusted

Point of Means Difference T-Value P-Value

4 -0.485 0.4476 -1.083 1.0000

5 -2.278 0.4476 -5.089 0.0058

6 -2.420 0.4476 -5.406 0.0034

7 -2.202 0.4476 -4.920 0.0078

8 -2.002 0.4476 -4.472 0.0176

9 -1.676 0.4476 -3.745 0.0686

10 -1.753 0.4476 -3.916 0.0496

Time Point = 4 subtracted from:

Time Difference SE of Adjusted

Point of Means Difference T-Value P-Value

5 -1.793 0.3947 -4.542 0.0155

6 -1.935 0.3947 -4.901 0.0081

7 -1.717 0.3947 -4.351 0.0220

8 -1.517 0.3947 -3.842 0.0571

9 -1.191 0.3947 -3.018 0.2768

10 -1.268 0.3947 -3.213 0.1904

Time Point = 5 subtracted from:

Time Difference SE of Adjusted

Point of Means Difference T-Value P-Value

6 -0.1419 0.3947 -0.3593 1.000

7 0.0756 0.3947 0.1914 1.000

8 0.2763 0.3947 0.6999 1.000

9 0.6015 0.3947 1.5237 1.000

10 0.5248 0.3947 1.3295 1.000

Time Point = 6 subtracted from:

Time Difference SE of Adjusted

Point of Means Difference T-Value P-Value

7 0.2174 0.3947 0.5507 1.000

8 0.4181 0.3947 1.0593 1.000

9 0.7433 0.3947 1.8831 1.000

10 0.6667 0.3947 1.6888 1.000

Time Point = 7 subtracted from:

Time Difference SE of Adjusted

Point of Means Difference T-Value P-Value

8 0.2007 0.3947 0.5085 1.000

9 0.5259 0.3947 1.3323 1.000

10 0.4493 0.3947 1.1381 1.000

Time Point = 8 subtracted from:

Time Difference SE of Adjusted

Point of Means Difference T-Value P-Value

9 0.3252 0.3947 0.8238 1.000

10 0.2485 0.3947 0.6296 1.000

Time Point = 9 subtracted from:

Time Difference SE of Adjusted

Point of Means Difference T-Value P-Value

10 -0.07667 0.3947 -0.1942 1.000

Grouping Information Using Sidak Method and 95.0% Confidence

Time

Point N Mean Grouping

3 2 -0.081 A

4 3 -0.566 A B

9 3 -1.758 A B C

10 3 -1.834 B C

8 3 -2.083 B C

7 3 -2.284 C

5 3 -2.359 C

6 3 -2.501 C

Means that do not share a letter are significantly different.

Sidak 95.0% Simultaneous Confidence Intervals

Response Variable Cq Prime

All Pairwise Comparisons among Levels of Time Point

Time Point = 3 subtracted from:

Time

Point Lower Center Upper -+---------+---------+---------+-----

4 -2.230 -0.485 1.2603 (--------*-------)

5 -4.023 -2.278 -0.5327 (--------*-------)

6 -4.165 -2.420 -0.6746 (--------*--------)

7 -3.947 -2.202 -0.4571 (--------*--------)

8 -3.747 -2.002 -0.2564 (--------*--------)

9 -3.421 -1.676 0.0688 (--------*-------)

10 -3.498 -1.753 -0.0079 (-------*--------)

-+---------+---------+---------+-----

-4.0 -2.0 0.0 2.0

Time Point = 4 subtracted from:

Time

Point Lower Center Upper -+---------+---------+---------+-----

5 -3.332 -1.793 -0.2539 (-------*-------)

6 -3.474 -1.935 -0.3958 (------*-------)

7 -3.256 -1.717 -0.1783 (------*-------)

8 -3.056 -1.517 0.0224 (------*-------)

9 -2.731 -1.191 0.3476 (-------*-------)

10 -2.807 -1.268 0.2709 (-------*------)

-+---------+---------+---------+-----

-4.0 -2.0 0.0 2.0

Time Point = 5 subtracted from:

Time

Point Lower Center Upper -+---------+---------+---------+-----

6 -1.681 -0.1419 1.397 (------*-------)

7 -1.464 0.0756 1.615 (------*-------)

8 -1.263 0.2763 1.815 (------*-------)

9 -0.938 0.6015 2.141 (-------*-------)

10 -1.014 0.5248 2.064 (-------*------)

-+---------+---------+---------+-----

-4.0 -2.0 0.0 2.0

Time Point = 6 subtracted from:

Time

Point Lower Center Upper -+---------+---------+---------+-----

7 -1.322 0.2174 1.756 (-------*-------)

8 -1.121 0.4181 1.957 (-------*-------)

9 -0.796 0.7433 2.282 (-------*------)

10 -0.872 0.6667 2.206 (------*-------)

-+---------+---------+---------+-----

-4.0 -2.0 0.0 2.0

Time Point = 7 subtracted from:

Time

Point Lower Center Upper -+---------+---------+---------+-----

8 -1.338 0.2007 1.740 (-------*-------)

9 -1.013 0.5259 2.065 (-------*------)

10 -1.090 0.4493 1.988 (------*-------)

-+---------+---------+---------+-----

-4.0 -2.0 0.0 2.0

Time Point = 8 subtracted from:

Time

Point Lower Center Upper -+---------+---------+---------+-----

9 -1.214 0.3252 1.864 (-------*------)

10 -1.291 0.2485 1.788 (------*-------)

-+---------+---------+---------+-----

-4.0 -2.0 0.0 2.0

Time Point = 9 subtracted from:

Time

Point Lower Center Upper -+---------+---------+---------+-----

10 -1.616 -0.07667 1.462 (-------*------)

-+---------+---------+---------+-----

-4.0 -2.0 0.0 2.0

Sidak Simultaneous Tests

Response Variable Cq Prime

All Pairwise Comparisons among Levels of Time Point

Time Point = 3 subtracted from:

Time Difference SE of Adjusted

Point of Means Difference T-Value P-Value

4 -0.485 0.4476 -1.083 1.0000

5 -2.278 0.4476 -5.089 0.0058

6 -2.420 0.4476 -5.406 0.0034

7 -2.202 0.4476 -4.920 0.0078

8 -2.002 0.4476 -4.472 0.0175

9 -1.676 0.4476 -3.745 0.0664

10 -1.753 0.4476 -3.916 0.0484

Time Point = 4 subtracted from:

Time Difference SE of Adjusted

Point of Means Difference T-Value P-Value

5 -1.793 0.3947 -4.542 0.0154

6 -1.935 0.3947 -4.901 0.0081

7 -1.717 0.3947 -4.351 0.0218

8 -1.517 0.3947 -3.842 0.0555

9 -1.191 0.3947 -3.018 0.2428

10 -1.268 0.3947 -3.213 0.1739

Time Point = 5 subtracted from:

Time Difference SE of Adjusted

Point of Means Difference T-Value P-Value

6 -0.1419 0.3947 -0.3593 1.0000

7 0.0756 0.3947 0.1914 1.0000

8 0.2763 0.3947 0.6999 1.0000

9 0.6015 0.3947 1.5237 0.9900

10 0.5248 0.3947 1.3295 0.9985

Time Point = 6 subtracted from:

Time Difference SE of Adjusted

Point of Means Difference T-Value P-Value

7 0.2174 0.3947 0.5507 1.0000

8 0.4181 0.3947 1.0593 1.0000

9 0.7433 0.3947 1.8831 0.9096

10 0.6667 0.3947 1.6888 0.9674

Time Point = 7 subtracted from:

Time Difference SE of Adjusted

Point of Means Difference T-Value P-Value

8 0.2007 0.3947 0.5085 1.0000

9 0.5259 0.3947 1.3323 0.9984

10 0.4493 0.3947 1.1381 0.9999

Time Point = 8 subtracted from:

Time Difference SE of Adjusted

Point of Means Difference T-Value P-Value

9 0.3252 0.3947 0.8238 1.000

10 0.2485 0.3947 0.6296 1.000

Time Point = 9 subtracted from:

Time Difference SE of Adjusted

Point of Means Difference T-Value P-Value

10 -0.07667 0.3947 -0.1942 1.000

Grouping Information Using Tukey Method and 95.0% Confidence

Time

Point N Mean Grouping

3 2 -0.081 A

4 3 -0.566 A B

9 3 -1.758 B C

10 3 -1.834 B C

8 3 -2.083 C

7 3 -2.284 C

5 3 -2.359 C

6 3 -2.501 C

Means that do not share a letter are significantly different.

Tukey 95.0% Simultaneous Confidence Intervals

Response Variable Cq Prime

All Pairwise Comparisons among Levels of Time Point

Time Point = 3 subtracted from:

Time

Point Lower Center Upper +---------+---------+---------+------

4 -2.083 -0.485 1.1135 (-------*-------)

5 -3.876 -2.278 -0.6795 (-------*-------)

6 -4.018 -2.420 -0.8213 (-------*-------)

7 -3.801 -2.202 -0.6039 (-------*-------)

8 -3.600 -2.002 -0.4032 (-------*-------)

9 -3.275 -1.676 -0.0780 (-------*-------)

10 -3.351 -1.753 -0.1547 (-------*-------)

+---------+---------+---------+------

-4.0 -2.0 0.0 2.0

Time Point = 4 subtracted from:

Time

Point Lower Center Upper +---------+---------+---------+------

5 -3.203 -1.793 -0.3834 (------*------)

6 -3.344 -1.935 -0.5252 (------*------)

7 -3.127 -1.717 -0.3078 (------*------)

8 -2.926 -1.517 -0.1071 (------*------)

9 -2.601 -1.191 0.2181 (------*------)

10 -2.678 -1.268 0.1415 (------*------)

+---------+---------+---------+------

-4.0 -2.0 0.0 2.0

Time Point = 5 subtracted from:

Time

Point Lower Center Upper +---------+---------+---------+------

6 -1.551 -0.1419 1.268 (------*------)

7 -1.334 0.0756 1.485 (------*------)

8 -1.133 0.2763 1.686 (------*------)

9 -0.808 0.6015 2.011 (------*------)

10 -0.885 0.5248 1.934 (------*------)

+---------+---------+---------+------

-4.0 -2.0 0.0 2.0

Time Point = 6 subtracted from:

Time

Point Lower Center Upper +---------+---------+---------+------

7 -1.192 0.2174 1.627 (------*------)

8 -0.991 0.4181 1.828 (------*------)

9 -0.666 0.7433 2.153 (------*------)

10 -0.743 0.6667 2.076 (------*------)

+---------+---------+---------+------

-4.0 -2.0 0.0 2.0

Time Point = 7 subtracted from:

Time

Point Lower Center Upper +---------+---------+---------+------

8 -1.209 0.2007 1.610 (------*------)

9 -0.884 0.5259 1.936 (------*------)

10 -0.960 0.4493 1.859 (------*------)

+---------+---------+---------+------

-4.0 -2.0 0.0 2.0

Time Point = 8 subtracted from:

Time

Point Lower Center Upper +---------+---------+---------+------

9 -1.084 0.3252 1.735 (------*------)

10 -1.161 0.2485 1.658 (------*------)

+---------+---------+---------+------

-4.0 -2.0 0.0 2.0

Time Point = 9 subtracted from:

Time

Point Lower Center Upper +---------+---------+---------+------

10 -1.486 -0.07667 1.333 (------*------)

+---------+---------+---------+------

-4.0 -2.0 0.0 2.0

Tukey Simultaneous Tests

Response Variable Cq Prime

All Pairwise Comparisons among Levels of Time Point

Time Point = 3 subtracted from:

Time Difference SE of Adjusted

Point of Means Difference T-Value P-Value

4 -0.485 0.4476 -1.083 0.9496

5 -2.278 0.4476 -5.089 0.0037

6 -2.420 0.4476 -5.406 0.0022

7 -2.202 0.4476 -4.920 0.0049

8 -2.002 0.4476 -4.472 0.0106

9 -1.676 0.4476 -3.745 0.0371

10 -1.753 0.4476 -3.916 0.0276

Time Point = 4 subtracted from:

Time Difference SE of Adjusted

Point of Means Difference T-Value P-Value

5 -1.793 0.3947 -4.542 0.0094

6 -1.935 0.3947 -4.901 0.0051

7 -1.717 0.3947 -4.351 0.0131

8 -1.517 0.3947 -3.842 0.0314

9 -1.191 0.3947 -3.018 0.1252

10 -1.268 0.3947 -3.213 0.0912

Time Point = 5 subtracted from:

Time Difference SE of Adjusted

Point of Means Difference T-Value P-Value

6 -0.1419 0.3947 -0.3593 0.9999

7 0.0756 0.3947 0.1914 1.0000

8 0.2763 0.3947 0.6999 0.9955

9 0.6015 0.3947 1.5237 0.7832

10 0.5248 0.3947 1.3295 0.8721

Time Point = 6 subtracted from:

Time Difference SE of Adjusted

Point of Means Difference T-Value P-Value

7 0.2174 0.3947 0.5507 0.9990

8 0.4181 0.3947 1.0593 0.9549

9 0.7433 0.3947 1.8831 0.5827

10 0.6667 0.3947 1.6888 0.6941

Time Point = 7 subtracted from:

Time Difference SE of Adjusted

Point of Means Difference T-Value P-Value

8 0.2007 0.3947 0.5085 0.9994

9 0.5259 0.3947 1.3323 0.8710

10 0.4493 0.3947 1.1381 0.9361

Time Point = 8 subtracted from:

Time Difference SE of Adjusted

Point of Means Difference T-Value P-Value

9 0.3252 0.3947 0.8238 0.9883

10 0.2485 0.3947 0.6296 0.9976

Time Point = 9 subtracted from:

Time Difference SE of Adjusted

Point of Means Difference T-Value P-Value

10 -0.07667 0.3947 -0.1942 1.000

**Residual Plots for Cq Prime**

**Main Effects Plot for Cq Prime**

135H6, T3 to T8:

**General Linear Model: Cq Prime versus Biounit, Time Point**

Factor Type Levels Values

Biounit random 3 1, 2, 3

Time Point fixed 6 3, 4, 5, 6, 7, 8

Analysis of Variance for Cq Prime, using Adjusted SS for Tests

Source DF Seq SS Adj SS Adj MS F P

Biounit 2 1.8174 0.6479 0.3239 1.14 0.362

Time Point 5 13.1440 13.1440 2.6288 9.24 0.002

Error 9 2.5600 2.5600 0.2844

Total 16 17.5215

S = 0.533330 R-Sq = 85.39% R-Sq(adj) = 74.03%

Term Coef SE Coef T P

Constant -1.6531 0.1318 -12.54 0.000

Biounit

1 -0.2511 0.1947 -1.29 0.229

2 0.2463 0.1822 1.35 0.209

Time Point

3 1.5276 0.3443 4.44 0.002

4 1.0869 0.2839 3.83 0.004

5 -0.7061 0.2839 -2.49 0.035

6 -0.8480 0.2839 -2.99 0.015

7 -0.6306 0.2839 -2.22 0.053

Unusual Observations for Cq Prime

Obs Cq Prime Fit SE Fit Residual St Resid

3 -1.67444 -0.81741 0.36433 -0.85704 -2.20 R

R denotes an observation with a large standardized residual.

Expected Mean Squares, using Adjusted SS

Expected Mean Square

Source for Each Term

1 Biounit (3) + 5.5000 (1)

2 Time Point (3) + Q[2]

3 Error (3)

Error Terms for Tests, using Adjusted SS

Synthesis

Source Error DF Error MS of Error MS

1 Biounit 9.00 0.2844 (3)

2 Time Point 9.00 0.2844 (3)

Variance Components, using Adjusted SS

Estimated

Source Value

Biounit 0.00718

Error 0.28444

Grouping Information Using Bonferroni Method and 95.0% Confidence

Time

Point N Mean Grouping

3 2 -0.126 A

4 3 -0.566 A B

8 3 -2.083 A B C

7 3 -2.284 B C

5 3 -2.359 C

6 3 -2.501 C

Means that do not share a letter are significantly different.

Bonferroni 95.0% Simultaneous Confidence Intervals

Response Variable Cq Prime

All Pairwise Comparisons among Levels of Time Point

Time Point = 3 subtracted from:

Time

Point Lower Center Upper --+---------+---------+---------+----

4 -2.404 -0.441 1.5225 (---------*---------)

5 -4.197 -2.234 -0.2704 (---------*---------)

6 -4.339 -2.376 -0.4123 (---------*---------)

7 -4.121 -2.158 -0.1949 (---------*---------)

8 -3.921 -1.957 0.0059 (---------*---------)

--+---------+---------+---------+----

-4.0 -2.0 0.0 2.0

Time Point = 4 subtracted from:

Time

Point Lower Center Upper --+---------+---------+---------+----

5 -3.515 -1.793 -0.0710 (--------*--------)

6 -3.657 -1.935 -0.2129 (-------*--------)

7 -3.439 -1.717 0.0045 (-------*--------)

8 -3.239 -1.517 0.2052 (-------*--------)

--+---------+---------+---------+----

-4.0 -2.0 0.0 2.0

Time Point = 5 subtracted from:

Time

Point Lower Center Upper --+---------+---------+---------+----

6 -1.864 -0.1419 1.580 (-------*--------)

7 -1.646 0.0756 1.797 (-------*--------)

8 -1.446 0.2763 1.998 (-------*--------)

--+---------+---------+---------+----

-4.0 -2.0 0.0 2.0

Time Point = 6 subtracted from:

Time

Point Lower Center Upper --+---------+---------+---------+----

7 -1.505 0.2174 1.939 (--------*--------)

8 -1.304 0.4181 2.140 (--------*--------)

--+---------+---------+---------+----

-4.0 -2.0 0.0 2.0

Time Point = 7 subtracted from:

Time

Point Lower Center Upper --+---------+---------+---------+----

8 -1.521 0.2007 1.923 (--------*--------)

--+---------+---------+---------+----

-4.0 -2.0 0.0 2.0

Bonferroni Simultaneous Tests

Response Variable Cq Prime

All Pairwise Comparisons among Levels of Time Point

Time Point = 3 subtracted from:

Time Difference SE of Adjusted

Point of Means Difference T-Value P-Value

4 -0.441 0.4965 -0.888 1.0000

5 -2.234 0.4965 -4.499 0.0224

6 -2.376 0.4965 -4.785 0.0149

7 -2.158 0.4965 -4.347 0.0279

8 -1.957 0.4965 -3.942 0.0509

Time Point = 4 subtracted from:

Time Difference SE of Adjusted

Point of Means Difference T-Value P-Value

5 -1.793 0.4355 -4.117 0.0391

6 -1.935 0.4355 -4.443 0.0242

7 -1.717 0.4355 -3.944 0.0508

8 -1.517 0.4355 -3.483 0.1036

Time Point = 5 subtracted from:

Time Difference SE of Adjusted

Point of Means Difference T-Value P-Value

6 -0.1419 0.4355 -0.3257 1.000

7 0.0756 0.4355 0.1735 1.000

8 0.2763 0.4355 0.6345 1.000

Time Point = 6 subtracted from:

Time Difference SE of Adjusted

Point of Means Difference T-Value P-Value

7 0.2174 0.4355 0.4993 1.000

8 0.4181 0.4355 0.9602 1.000

Time Point = 7 subtracted from:

Time Difference SE of Adjusted

Point of Means Difference T-Value P-Value

8 0.2007 0.4355 0.4610 1.000

Grouping Information Using Sidak Method and 95.0% Confidence

Time

Point N Mean Grouping

3 2 -0.126 A

4 3 -0.566 A B

8 3 -2.083 B C

7 3 -2.284 C

5 3 -2.359 C

6 3 -2.501 C

Means that do not share a letter are significantly different.

Sidak 95.0% Simultaneous Confidence Intervals

Response Variable Cq Prime

All Pairwise Comparisons among Levels of Time Point

Time Point = 3 subtracted from:

Time

Point Lower Center Upper --+---------+---------+---------+----

4 -2.396 -0.441 1.5147 (---------*---------)

5 -4.189 -2.234 -0.2782 (---------*---------)

6 -4.331 -2.376 -0.4201 (---------*---------)

7 -4.114 -2.158 -0.2027 (---------*---------)

8 -3.913 -1.957 -0.0019 (---------*---------)

--+---------+---------+---------+----

-4.0 -2.0 0.0 2.0

Time Point = 4 subtracted from:

Time

Point Lower Center Upper --+---------+---------+---------+----

5 -3.508 -1.793 -0.0779 (--------*--------)

6 -3.650 -1.935 -0.2197 (-------*--------)

7 -3.432 -1.717 -0.0023 (-------*--------)

8 -3.232 -1.517 0.1984 (-------*--------)

--+---------+---------+---------+----

-4.0 -2.0 0.0 2.0

Time Point = 5 subtracted from:

Time

Point Lower Center Upper --+---------+---------+---------+----

6 -1.857 -0.1419 1.573 (-------*--------)

7 -1.640 0.0756 1.791 (-------*--------)

8 -1.439 0.2763 1.991 (-------*--------)

--+---------+---------+---------+----

-4.0 -2.0 0.0 2.0

Time Point = 6 subtracted from:

Time

Point Lower Center Upper --+---------+---------+---------+----

7 -1.498 0.2174 1.932 (-------*--------)

8 -1.297 0.4181 2.133 (-------*--------)

--+---------+---------+---------+----

-4.0 -2.0 0.0 2.0

Time Point = 7 subtracted from:

Time

Point Lower Center Upper --+---------+---------+---------+----

8 -1.514 0.2007 1.916 (--------*--------)

--+---------+---------+---------+----

-4.0 -2.0 0.0 2.0

Sidak Simultaneous Tests

Response Variable Cq Prime

All Pairwise Comparisons among Levels of Time Point

Time Point = 3 subtracted from:

Time Difference SE of Adjusted

Point of Means Difference T-Value P-Value

4 -0.441 0.4965 -0.888 0.9995

5 -2.234 0.4965 -4.499 0.0221

6 -2.376 0.4965 -4.785 0.0148

7 -2.158 0.4965 -4.347 0.0275

8 -1.957 0.4965 -3.942 0.0497

Time Point = 4 subtracted from:

Time Difference SE of Adjusted

Point of Means Difference T-Value P-Value

5 -1.793 0.4355 -4.117 0.0384

6 -1.935 0.4355 -4.443 0.0240

7 -1.717 0.4355 -3.944 0.0496

8 -1.517 0.4355 -3.483 0.0987

Time Point = 5 subtracted from:

Time Difference SE of Adjusted

Point of Means Difference T-Value P-Value

6 -0.1419 0.4355 -0.3257 1.000

7 0.0756 0.4355 0.1735 1.000

8 0.2763 0.4355 0.6345 1.000

Time Point = 6 subtracted from:

Time Difference SE of Adjusted

Point of Means Difference T-Value P-Value

7 0.2174 0.4355 0.4993 1.0000

8 0.4181 0.4355 0.9602 0.9988

Time Point = 7 subtracted from:

Time Difference SE of Adjusted

Point of Means Difference T-Value P-Value

8 0.2007 0.4355 0.4610 1.000

Grouping Information Using Tukey Method and 95.0% Confidence

Time

Point N Mean Grouping

3 2 -0.126 A

4 3 -0.566 A B

8 3 -2.083 B C

7 3 -2.284 C

5 3 -2.359 C

6 3 -2.501 C

Means that do not share a letter are significantly different.

Tukey 95.0% Simultaneous Confidence Intervals

Response Variable Cq Prime

All Pairwise Comparisons among Levels of Time Point

Time Point = 3 subtracted from:

Time

Point Lower Center Upper -+---------+---------+---------+-----

4 -2.203 -0.441 1.3217 (--------*--------)

5 -3.996 -2.234 -0.4713 (--------*--------)

6 -4.138 -2.376 -0.6131 (--------*--------)

7 -3.921 -2.158 -0.3957 (--------*--------)

8 -3.720 -1.957 -0.1950 (--------*--------)

-+---------+---------+---------+-----

-4.0 -2.0 0.0 2.0

Time Point = 4 subtracted from:

Time

Point Lower Center Upper -+---------+---------+---------+-----

5 -3.339 -1.793 -0.2472 (-------*-------)

6 -3.481 -1.935 -0.3891 (------*-------)

7 -3.263 -1.717 -0.1717 (------*-------)

8 -3.062 -1.517 0.0291 (------*-------)

-+---------+---------+---------+-----

-4.0 -2.0 0.0 2.0

Time Point = 5 subtracted from:

Time

Point Lower Center Upper -+---------+---------+---------+-----

6 -1.688 -0.1419 1.404 (------*-------)

7 -1.470 0.0756 1.621 (------*-------)

8 -1.269 0.2763 1.822 (------*-------)

-+---------+---------+---------+-----

-4.0 -2.0 0.0 2.0

Time Point = 6 subtracted from:

Time

Point Lower Center Upper -+---------+---------+---------+-----

7 -1.328 0.2174 1.763 (-------*-------)

8 -1.128 0.4181 1.964 (-------*-------)

-+---------+---------+---------+-----

-4.0 -2.0 0.0 2.0

Time Point = 7 subtracted from:

Time

Point Lower Center Upper -+---------+---------+---------+-----

8 -1.345 0.2007 1.746 (-------*-------)

-+---------+---------+---------+-----

-4.0 -2.0 0.0 2.0

Tukey Simultaneous Tests

Response Variable Cq Prime

All Pairwise Comparisons among Levels of Time Point

Time Point = 3 subtracted from:

Time Difference SE of Adjusted

Point of Means Difference T-Value P-Value

4 -0.441 0.4965 -0.888 0.9403

5 -2.234 0.4965 -4.499 0.0133

6 -2.376 0.4965 -4.785 0.0090

7 -2.158 0.4965 -4.347 0.0163

8 -1.957 0.4965 -3.942 0.0287

Time Point = 4 subtracted from:

Time Difference SE of Adjusted

Point of Means Difference T-Value P-Value

5 -1.793 0.4355 -4.117 0.0225

6 -1.935 0.4355 -4.443 0.0143

7 -1.717 0.4355 -3.944 0.0287

8 -1.517 0.4355 -3.483 0.0552

Time Point = 5 subtracted from:

Time Difference SE of Adjusted

Point of Means Difference T-Value P-Value

6 -0.1419 0.4355 -0.3257 0.9993

7 0.0756 0.4355 0.1735 1.0000

8 0.2763 0.4355 0.6345 0.9851

Time Point = 6 subtracted from:

Time Difference SE of Adjusted

Point of Means Difference T-Value P-Value

7 0.2174 0.4355 0.4993 0.9949

8 0.4181 0.4355 0.9602 0.9197

Time Point = 7 subtracted from:

Time Difference SE of Adjusted

Point of Means Difference T-Value P-Value

8 0.2007 0.4355 0.4610 0.9965

**Residual Plots for Cq Prime**

**Main Effects Plot for Cq Prime**

**General Linear Model: Cq Prime versus Biounit, Time Point**

Factor Type Levels Values

Biounit random 3 1, 2, 3

Time Point fixed 4 3, 4, 5, 6

Analysis of Variance for Cq Prime, using Adjusted SS for Tests

Source DF Seq SS Adj SS Adj MS F P

Biounit 2 1.5894 0.3963 0.1981 0.48 0.647

Time Point 3 11.1395 11.1395 3.7132 8.93 0.019

Error 5 2.0792 2.0792 0.4158

Total 10 14.8081

S = 0.644861 R-Sq = 85.96% R-Sq(adj) = 71.92%

Term Coef SE Coef T P

Constant -1.3925 0.2011 -6.93 0.001

Biounit

1 -0.2867 0.3040 -0.94 0.389

2 0.2008 0.2740 0.73 0.496

Time Point

3 1.2492 0.3949 3.16 0.025

4 0.8262 0.3313 2.49 0.055

5 -0.9668 0.3313 -2.92 0.033

Expected Mean Squares, using Adjusted SS

Expected Mean Square

Source for Each Term

1 Biounit (3) + 3.5000 (1)

2 Time Point (3) + Q[2]

3 Error (3)

Error Terms for Tests, using Adjusted SS

Synthesis

Source Error DF Error MS of Error MS

1 Biounit 5.00 0.4158 (3)

2 Time Point 5.00 0.4158 (3)

Variance Components, using Adjusted SS

Estimated

Source Value

Biounit -0.06221

Error 0.41585

Grouping Information Using Bonferroni Method and 95.0% Confidence

Time

Point N Mean Grouping

3 2 -0.143 A

4 3 -0.566 A

5 3 -2.359 A

6 3 -2.501 A

Means that do not share a letter are significantly different.

Bonferroni 95.0% Simultaneous Confidence Intervals

Response Variable Cq Prime

All Pairwise Comparisons among Levels of Time Point

Time Point = 3 subtracted from:

Time

Point Lower Center Upper -----+---------+---------+---------+-

4 -2.988 -0.423 2.1423 (------------*------------)

5 -4.781 -2.216 0.3493 (------------*------------)

6 -4.923 -2.358 0.2075 (------------*------------)

-----+---------+---------+---------+-

-4.0 -2.0 0.0 2.0

Time Point = 4 subtracted from:

Time

Point Lower Center Upper -----+---------+---------+---------+-

5 -4.015 -1.793 0.4286 (----------*----------)

6 -4.156 -1.935 0.2868 (----------*----------)

-----+---------+---------+---------+-

-4.0 -2.0 0.0 2.0

Time Point = 5 subtracted from:

Time

Point Lower Center Upper -----+---------+---------+---------+-

6 -2.363 -0.1419 2.080 (----------*----------)

-----+---------+---------+---------+-

-4.0 -2.0 0.0 2.0

Bonferroni Simultaneous Tests

Response Variable Cq Prime

All Pairwise Comparisons among Levels of Time Point

Time Point = 3 subtracted from:

Time Difference SE of Adjusted

Point of Means Difference T-Value P-Value

4 -0.423 0.6080 -0.696 1.0000

5 -2.216 0.6080 -3.645 0.0890

6 -2.358 0.6080 -3.878 0.0700

Time Point = 4 subtracted from:

Time Difference SE of Adjusted

Point of Means Difference T-Value P-Value

5 -1.793 0.5265 -3.405 0.1148

6 -1.935 0.5265 -3.675 0.0862

Time Point = 5 subtracted from:

Time Difference SE of Adjusted

Point of Means Difference T-Value P-Value

6 -0.1419 0.5265 -0.2694 1.000

Grouping Information Using Sidak Method and 95.0% Confidence

Time

Point N Mean Grouping

3 2 -0.143 A

4 3 -0.566 A

5 3 -2.359 A

6 3 -2.501 A

Means that do not share a letter are significantly different.

Sidak 95.0% Simultaneous Confidence Intervals

Response Variable Cq Prime

All Pairwise Comparisons among Levels of Time Point

Time Point = 3 subtracted from:

Time

Point Lower Center Upper -----+---------+---------+---------+-

4 -2.975 -0.423 2.1289 (------------*------------)

5 -4.768 -2.216 0.3359 (------------*------------)

6 -4.910 -2.358 0.1940 (------------*------------)

-----+---------+---------+---------+-

-4.0 -2.0 0.0 2.0

Time Point = 4 subtracted from:

Time

Point Lower Center Upper -----+---------+---------+---------+-

5 -4.003 -1.793 0.4170 (----------*----------)

6 -4.145 -1.935 0.2751 (----------*----------)

-----+---------+---------+---------+-

-4.0 -2.0 0.0 2.0

Time Point = 5 subtracted from:

Time

Point Lower Center Upper -----+---------+---------+---------+-

6 -2.352 -0.1419 2.068 (----------*----------)

-----+---------+---------+---------+-

-4.0 -2.0 0.0 2.0

Sidak Simultaneous Tests

Response Variable Cq Prime

All Pairwise Comparisons among Levels of Time Point

Time Point = 3 subtracted from:

Time Difference SE of Adjusted

Point of Means Difference T-Value P-Value

4 -0.423 0.6080 -0.696 0.9874

5 -2.216 0.6080 -3.645 0.0857

6 -2.358 0.6080 -3.878 0.0680

Time Point = 4 subtracted from:

Time Difference SE of Adjusted

Point of Means Difference T-Value P-Value

5 -1.793 0.5265 -3.405 0.1095

6 -1.935 0.5265 -3.675 0.0832

Time Point = 5 subtracted from:

Time Difference SE of Adjusted

Point of Means Difference T-Value P-Value

6 -0.1419 0.5265 -0.2694 0.9999

Grouping Information Using Tukey Method and 95.0% Confidence

Time

Point N Mean Grouping

3 2 -0.143 A

4 3 -0.566 A B

5 3 -2.359 A B

6 3 -2.501 B

Means that do not share a letter are significantly different.

Tukey 95.0% Simultaneous Confidence Intervals

Response Variable Cq Prime

All Pairwise Comparisons among Levels of Time Point

Time Point = 3 subtracted from:

Time

Point Lower Center Upper ---+---------+---------+---------+---

4 -2.667 -0.423 1.8212 (----------*----------)

5 -4.460 -2.216 0.0282 (----------*----------)

6 -4.602 -2.358 -0.1137 (----------*----------)

---+---------+---------+---------+---

-4.0 -2.0 0.0 2.0

Time Point = 4 subtracted from:

Time

Point Lower Center Upper ---+---------+---------+---------+---

5 -3.736 -1.793 0.150499 (---------*---------)

6 -3.878 -1.935 0.008647 (--------*---------)

---+---------+---------+---------+---

-4.0 -2.0 0.0 2.0

Time Point = 5 subtracted from:

Time

Point Lower Center Upper ---+---------+---------+---------+---

6 -2.085 -0.1419 1.802 (--------*---------)

---+---------+---------+---------+---

-4.0 -2.0 0.0 2.0

Tukey Simultaneous Tests

Response Variable Cq Prime

All Pairwise Comparisons among Levels of Time Point

Time Point = 3 subtracted from:

Time Difference SE of Adjusted

Point of Means Difference T-Value P-Value

4 -0.423 0.6080 -0.696 0.8946

5 -2.216 0.6080 -3.645 0.0523

6 -2.358 0.6080 -3.878 0.0416

Time Point = 4 subtracted from:

Time Difference SE of Adjusted

Point of Means Difference T-Value P-Value

5 -1.793 0.5265 -3.405 0.0666

6 -1.935 0.5265 -3.675 0.0508

Time Point = 5 subtracted from:

Time Difference SE of Adjusted

Point of Means Difference T-Value P-Value

6 -0.1419 0.5265 -0.2694 0.9923

**Residual Plots for Cq Prime**

**Main Effects Plot for Cq Prime**

177F1:

**General Linear Model: Cq Prime versus Biounit, Time Point**

Factor Type Levels Values

Biounit random 3 1, 2, 3

Time Point fixed 8 3, 4, 5, 6, 7, 8, 9, 10

Analysis of Variance for Cq Prime, using Adjusted SS for Tests

Source DF Seq SS Adj SS Adj MS F P

Biounit 2 0.05778 0.07476 0.03738 1.99 0.177

Time Point 7 0.08592 0.08592 0.01227 0.65 0.707

Error 13 0.24450 0.24450 0.01881

Total 22 0.38821

S = 0.137141 R-Sq = 37.02% R-Sq(adj) = 0.00%

Term Coef SE Coef T P

Constant 0.08910 0.02898 3.07 0.009

Biounit

1 -0.08000 0.04232 -1.89 0.081

2 0.06174 0.04029 1.53 0.149

Time Point

3 -0.12910 0.09071 -1.42 0.178

4 -0.02484 0.07444 -0.33 0.744

5 -0.05669 0.07444 -0.76 0.460

6 0.02257 0.07444 0.30 0.767

7 0.04442 0.07444 0.60 0.561

8 0.04405 0.07444 0.59 0.564

9 -0.00076 0.07444 -0.01 0.992

Unusual Observations for Cq Prime

Obs Cq Prime Fit SE Fit Residual St Resid

7 0.332778 0.094144 0.088840 0.238634 2.28 R

R denotes an observation with a large standardized residual.

Expected Mean Squares, using Adjusted SS

Expected Mean Square

Source for Each Term

1 Biounit (3) + 7.5000 (1)

2 Time Point (3) + Q[2]

3 Error (3)

Error Terms for Tests, using Adjusted SS

Synthesis

Source Error DF Error MS of Error MS

1 Biounit 13.00 0.01881 (3)

2 Time Point 13.00 0.01881 (3)

Variance Components, using Adjusted SS

Estimated

Source Value

Biounit 0.00248

Error 0.01881

Grouping Information Using Bonferroni Method and 95.0% Confidence

Time

Point N Mean Grouping

10 3 0.18944 A

7 3 0.13352 A

8 3 0.13315 A

6 3 0.11167 A

9 3 0.08833 A

4 3 0.06426 A

5 3 0.03241 A

3 2 -0.04000 A

Means that do not share a letter are significantly different.

Bonferroni 95.0% Simultaneous Confidence Intervals

Response Variable Cq Prime

All Pairwise Comparisons among Levels of Time Point

Time Point = 3 subtracted from:

Time

Point Lower Center Upper ----+---------+---------+---------+--

4 -0.3924 0.10426 0.6009 (-------------*-------------)

5 -0.4243 0.07241 0.5691 (-------------*-------------)

6 -0.3450 0.15167 0.6483 (-------------*--------------)

7 -0.3232 0.17352 0.6702 (-------------*-------------)

8 -0.3235 0.17315 0.6698 (-------------*-------------)

9 -0.3683 0.12833 0.6250 (--------------*-------------)

10 -0.2672 0.22944 0.7261 (--------------*-------------)

----+---------+---------+---------+--

-0.35 0.00 0.35 0.70

Time Point = 4 subtracted from:

Time

Point Lower Center Upper ----+---------+---------+---------+--

5 -0.4699 -0.03185 0.4062 (-----------*------------)

6 -0.3906 0.04741 0.4854 (-----------*------------)

7 -0.3688 0.06926 0.5073 (------------*-----------)

8 -0.3691 0.06889 0.5069 (------------*-----------)

9 -0.4140 0.02407 0.4621 (------------*-----------)

10 -0.3128 0.12519 0.5632 (------------*-----------)

----+---------+---------+---------+--

-0.35 0.00 0.35 0.70

Time Point = 5 subtracted from:

Time

Point Lower Center Upper ----+---------+---------+---------+--

6 -0.3588 0.07926 0.5173 (-----------*------------)

7 -0.3369 0.10111 0.5391 (------------*-----------)

8 -0.3373 0.10074 0.5388 (------------*-----------)

9 -0.3821 0.05593 0.4940 (------------*-----------)

10 -0.2810 0.15704 0.5951 (-----------*------------)

----+---------+---------+---------+--

-0.35 0.00 0.35 0.70

Time Point = 6 subtracted from:

Time

Point Lower Center Upper ----+---------+---------+---------+--

7 -0.4162 0.02185 0.4599 (------------*-----------)

8 -0.4165 0.02148 0.4595 (------------*-----------)

9 -0.4614 -0.02333 0.4147 (-----------*------------)

10 -0.3603 0.07778 0.5158 (-----------*------------)

----+---------+---------+---------+--

-0.35 0.00 0.35 0.70

Time Point = 7 subtracted from:

Time

Point Lower Center Upper ----+---------+---------+---------+--

8 -0.4384 -0.00037 0.4377 (------------*------------)

9 -0.4832 -0.04519 0.3928 (------------*-----------)

10 -0.3821 0.05593 0.4940 (------------*-----------)

----+---------+---------+---------+--

-0.35 0.00 0.35 0.70

Time Point = 8 subtracted from:

Time

Point Lower Center Upper ----+---------+---------+---------+--

9 -0.4828 -0.04481 0.3932 (------------*-----------)

10 -0.3817 0.05630 0.4943 (------------*-----------)

----+---------+---------+---------+--

-0.35 0.00 0.35 0.70

Time Point = 9 subtracted from:

Time

Point Lower Center Upper ----+---------+---------+---------+--

10 -0.3369 0.1011 0.5391 (------------*-----------)

----+---------+---------+---------+--

-0.35 0.00 0.35 0.70

Bonferroni Simultaneous Tests

Response Variable Cq Prime

All Pairwise Comparisons among Levels of Time Point

Time Point = 3 subtracted from:

Time Difference SE of Adjusted

Point of Means Difference T-Value P-Value

4 0.10426 0.1270 0.8211 1.000

5 0.07241 0.1270 0.5703 1.000

6 0.15167 0.1270 1.1945 1.000

7 0.17352 0.1270 1.3666 1.000

8 0.17315 0.1270 1.3637 1.000

9 0.12833 0.1270 1.0108 1.000

10 0.22944 0.1270 1.8071 1.000

Time Point = 4 subtracted from:

Time Difference SE of Adjusted

Point of Means Difference T-Value P-Value

5 -0.03185 0.1120 -0.2845 1.000

6 0.04741 0.1120 0.4234 1.000

7 0.06926 0.1120 0.6185 1.000

8 0.06889 0.1120 0.6152 1.000

9 0.02407 0.1120 0.2150 1.000

10 0.12519 0.1120 1.1180 1.000

Time Point = 5 subtracted from:

Time Difference SE of Adjusted

Point of Means Difference T-Value P-Value

6 0.07926 0.1120 0.7078 1.000

7 0.10111 0.1120 0.9030 1.000

8 0.10074 0.1120 0.8997 1.000

9 0.05593 0.1120 0.4995 1.000

10 0.15704 0.1120 1.4024 1.000

Time Point = 6 subtracted from:

Time Difference SE of Adjusted

Point of Means Difference T-Value P-Value

7 0.02185 0.1120 0.1951 1.000

8 0.02148 0.1120 0.1918 1.000

9 -0.02333 0.1120 -0.2084 1.000

10 0.07778 0.1120 0.6946 1.000

Time Point = 7 subtracted from:

Time Difference SE of Adjusted

Point of Means Difference T-Value P-Value

8 -0.00037 0.1120 -0.0033 1.000

9 -0.04519 0.1120 -0.4035 1.000

10 0.05593 0.1120 0.4995 1.000

Time Point = 8 subtracted from:

Time Difference SE of Adjusted

Point of Means Difference T-Value P-Value

9 -0.04481 0.1120 -0.4002 1.000

10 0.05630 0.1120 0.5028 1.000

Time Point = 9 subtracted from:

Time Difference SE of Adjusted

Point of Means Difference T-Value P-Value

10 0.1011 0.1120 0.9030 1.000

Grouping Information Using Sidak Method and 95.0% Confidence

Time

Point N Mean Grouping

10 3 0.18944 A

7 3 0.13352 A

8 3 0.13315 A

6 3 0.11167 A

9 3 0.08833 A

4 3 0.06426 A

5 3 0.03241 A

3 2 -0.04000 A

Means that do not share a letter are significantly different.

Sidak 95.0% Simultaneous Confidence Intervals

Response Variable Cq Prime

All Pairwise Comparisons among Levels of Time Point

Time Point = 3 subtracted from:

Time

Point Lower Center Upper ----+---------+---------+---------+--

4 -0.3908 0.10426 0.5993 (-------------*-------------)

5 -0.4226 0.07241 0.5674 (-------------*-------------)

6 -0.3434 0.15167 0.6467 (-------------*-------------)

7 -0.3215 0.17352 0.6685 (-------------*-------------)

8 -0.3219 0.17315 0.6682 (-------------*-------------)

9 -0.3667 0.12833 0.6234 (-------------*-------------)

10 -0.2656 0.22944 0.7245 (--------------*-------------)

----+---------+---------+---------+--

-0.35 0.00 0.35 0.70

Time Point = 4 subtracted from:

Time

Point Lower Center Upper ----+---------+---------+---------+--

5 -0.4684 -0.03185 0.4047 (-----------*------------)

6 -0.3892 0.04741 0.4840 (-----------*------------)

7 -0.3673 0.06926 0.5058 (-----------*-----------)

8 -0.3677 0.06889 0.5055 (------------*-----------)

9 -0.4125 0.02407 0.4606 (------------*-----------)

10 -0.3114 0.12519 0.5618 (------------*-----------)

----+---------+---------+---------+--

-0.35 0.00 0.35 0.70

Time Point = 5 subtracted from:

Time

Point Lower Center Upper ----+---------+---------+---------+--

6 -0.3573 0.07926 0.5158 (-----------*------------)

7 -0.3355 0.10111 0.5377 (------------*-----------)

8 -0.3358 0.10074 0.5373 (------------*-----------)

9 -0.3806 0.05593 0.4925 (------------*-----------)

10 -0.2795 0.15704 0.5936 (-----------*------------)

----+---------+---------+---------+--

-0.35 0.00 0.35 0.70

Time Point = 6 subtracted from:

Time

Point Lower Center Upper ----+---------+---------+---------+--

7 -0.4147 0.02185 0.4584 (------------*-----------)

8 -0.4151 0.02148 0.4581 (------------*-----------)

9 -0.4599 -0.02333 0.4132 (-----------*------------)

10 -0.3588 0.07778 0.5143 (-----------*------------)

----+---------+---------+---------+--

-0.35 0.00 0.35 0.70

Time Point = 7 subtracted from:

Time

Point Lower Center Upper ----+---------+---------+---------+--

8 -0.4369 -0.00037 0.4362 (-----------*-----------)

9 -0.4818 -0.04519 0.3914 (------------*-----------)

10 -0.3806 0.05593 0.4925 (------------*-----------)

----+---------+---------+---------+--

-0.35 0.00 0.35 0.70

Time Point = 8 subtracted from:

Time

Point Lower Center Upper ----+---------+---------+---------+--

9 -0.4814 -0.04481 0.3918 (------------*-----------)

10 -0.3803 0.05630 0.4929 (------------*-----------)

----+---------+---------+---------+--

-0.35 0.00 0.35 0.70

Time Point = 9 subtracted from:

Time

Point Lower Center Upper ----+---------+---------+---------+--

10 -0.3355 0.1011 0.5377 (------------*-----------)

----+---------+---------+---------+--

-0.35 0.00 0.35 0.70

Sidak Simultaneous Tests

Response Variable Cq Prime

All Pairwise Comparisons among Levels of Time Point

Time Point = 3 subtracted from:

Time Difference SE of Adjusted

Point of Means Difference T-Value P-Value

4 0.10426 0.1270 0.8211 1.0000

5 0.07241 0.1270 0.5703 1.0000

6 0.15167 0.1270 1.1945 0.9997

7 0.17352 0.1270 1.3666 0.9977

8 0.17315 0.1270 1.3637 0.9978

9 0.12833 0.1270 1.0108 1.0000

10 0.22944 0.1270 1.8071 0.9368

Time Point = 4 subtracted from:

Time Difference SE of Adjusted

Point of Means Difference T-Value P-Value

5 -0.03185 0.1120 -0.2845 1.0000

6 0.04741 0.1120 0.4234 1.0000

7 0.06926 0.1120 0.6185 1.0000

8 0.06889 0.1120 0.6152 1.0000

9 0.02407 0.1120 0.2150 1.0000

10 0.12519 0.1120 1.1180 0.9999

Time Point = 5 subtracted from:

Time Difference SE of Adjusted

Point of Means Difference T-Value P-Value

6 0.07926 0.1120 0.7078 1.0000

7 0.10111 0.1120 0.9030 1.0000

8 0.10074 0.1120 0.8997 1.0000

9 0.05593 0.1120 0.4995 1.0000

10 0.15704 0.1120 1.4024 0.9967

Time Point = 6 subtracted from:

Time Difference SE of Adjusted

Point of Means Difference T-Value P-Value

7 0.02185 0.1120 0.1951 1.000

8 0.02148 0.1120 0.1918 1.000

9 -0.02333 0.1120 -0.2084 1.000

10 0.07778 0.1120 0.6946 1.000

Time Point = 7 subtracted from:

Time Difference SE of Adjusted

Point of Means Difference T-Value P-Value

8 -0.00037 0.1120 -0.0033 1.000

9 -0.04519 0.1120 -0.4035 1.000

10 0.05593 0.1120 0.4995 1.000

Time Point = 8 subtracted from:

Time Difference SE of Adjusted

Point of Means Difference T-Value P-Value

9 -0.04481 0.1120 -0.4002 1.000

10 0.05630 0.1120 0.5028 1.000

Time Point = 9 subtracted from:

Time Difference SE of Adjusted

Point of Means Difference T-Value P-Value

10 0.1011 0.1120 0.9030 1.000

Grouping Information Using Tukey Method and 95.0% Confidence

Time

Point N Mean Grouping

10 3 0.18944 A

7 3 0.13352 A

8 3 0.13315 A

6 3 0.11167 A

9 3 0.08833 A

4 3 0.06426 A

5 3 0.03241 A

3 2 -0.04000 A

Means that do not share a letter are significantly different.

Tukey 95.0% Simultaneous Confidence Intervals

Response Variable Cq Prime

All Pairwise Comparisons among Levels of Time Point

Time Point = 3 subtracted from:

Time

Point Lower Center Upper ---+---------+---------+---------+---

4 -0.3491 0.10426 0.5576 (------------*------------)

5 -0.3810 0.07241 0.5258 (------------*------------)

6 -0.3017 0.15167 0.6051 (------------*------------)

7 -0.2799 0.17352 0.6269 (------------*------------)

8 -0.2802 0.17315 0.6265 (------------*------------)

9 -0.3251 0.12833 0.5817 (------------*------------)

10 -0.2239 0.22944 0.6828 (------------*------------)

---+---------+---------+---------+---

-0.35 0.00 0.35 0.70

Time Point = 4 subtracted from:

Time

Point Lower Center Upper ---+---------+---------+---------+---

5 -0.4317 -0.03185 0.3680 (----------*-----------)

6 -0.3524 0.04741 0.4473 (----------*-----------)

7 -0.3306 0.06926 0.4691 (----------*----------)

8 -0.3310 0.06889 0.4687 (----------*----------)

9 -0.3758 0.02407 0.4239 (-----------*----------)

10 -0.2747 0.12519 0.5250 (-----------*----------)

---+---------+---------+---------+---

-0.35 0.00 0.35 0.70

Time Point = 5 subtracted from:

Time

Point Lower Center Upper ---+---------+---------+---------+---

6 -0.3206 0.07926 0.4791 (----------*-----------)

7 -0.2987 0.10111 0.5010 (-----------*----------)

8 -0.2991 0.10074 0.5006 (-----------*----------)

9 -0.3439 0.05593 0.4558 (-----------*----------)

10 -0.2428 0.15704 0.5569 (----------*-----------)

---+---------+---------+---------+---

-0.35 0.00 0.35 0.70

Time Point = 6 subtracted from:

Time

Point Lower Center Upper ---+---------+---------+---------+---

7 -0.3780 0.02185 0.4217 (-----------*----------)

8 -0.3784 0.02148 0.4213 (-----------*----------)

9 -0.4232 -0.02333 0.3765 (----------*-----------)

10 -0.3221 0.07778 0.4776 (----------*-----------)

---+---------+---------+---------+---

-0.35 0.00 0.35 0.70

Time Point = 7 subtracted from:

Time

Point Lower Center Upper ---+---------+---------+---------+---

8 -0.4002 -0.00037 0.3995 (----------*----------)

9 -0.4450 -0.04519 0.3547 (-----------*----------)

10 -0.3439 0.05593 0.4558 (-----------*----------)

---+---------+---------+---------+---

-0.35 0.00 0.35 0.70

Time Point = 8 subtracted from:

Time

Point Lower Center Upper ---+---------+---------+---------+---

9 -0.4447 -0.04481 0.3550 (-----------*----------)

10 -0.3436 0.05630 0.4561 (-----------*----------)

---+---------+---------+---------+---

-0.35 0.00 0.35 0.70

Time Point = 9 subtracted from:

Time

Point Lower Center Upper ---+---------+---------+---------+---

10 -0.2987 0.1011 0.5010 (-----------*----------)

---+---------+---------+---------+---

-0.35 0.00 0.35 0.70

Tukey Simultaneous Tests

Response Variable Cq Prime

All Pairwise Comparisons among Levels of Time Point

Time Point = 3 subtracted from:

Time Difference SE of Adjusted

Point of Means Difference T-Value P-Value

4 0.10426 0.1270 0.8211 0.9885

5 0.07241 0.1270 0.5703 0.9987

6 0.15167 0.1270 1.1945 0.9199

7 0.17352 0.1270 1.3666 0.8568

8 0.17315 0.1270 1.3637 0.8581

9 0.12833 0.1270 1.0108 0.9644

10 0.22944 0.1270 1.8071 0.6265

Time Point = 4 subtracted from:

Time Difference SE of Adjusted

Point of Means Difference T-Value P-Value

5 -0.03185 0.1120 -0.2845 1.0000

6 0.04741 0.1120 0.4234 0.9998

7 0.06926 0.1120 0.6185 0.9979

8 0.06889 0.1120 0.6152 0.9979

9 0.02407 0.1120 0.2150 1.0000

10 0.12519 0.1120 1.1180 0.9413

Time Point = 5 subtracted from:

Time Difference SE of Adjusted

Point of Means Difference T-Value P-Value

6 0.07926 0.1120 0.7078 0.9952

7 0.10111 0.1120 0.9030 0.9804

8 0.10074 0.1120 0.8997 0.9808

9 0.05593 0.1120 0.4995 0.9995

10 0.15704 0.1120 1.4024 0.8413

Time Point = 6 subtracted from:

Time Difference SE of Adjusted

Point of Means Difference T-Value P-Value

7 0.02185 0.1120 0.1951 1.0000

8 0.02148 0.1120 0.1918 1.0000

9 -0.02333 0.1120 -0.2084 1.0000

10 0.07778 0.1120 0.6946 0.9957

Time Point = 7 subtracted from:

Time Difference SE of Adjusted

Point of Means Difference T-Value P-Value

8 -0.00037 0.1120 -0.0033 1.0000

9 -0.04519 0.1120 -0.4035 0.9999

10 0.05593 0.1120 0.4995 0.9995

Time Point = 8 subtracted from:

Time Difference SE of Adjusted

Point of Means Difference T-Value P-Value

9 -0.04481 0.1120 -0.4002 0.9999

10 0.05630 0.1120 0.5028 0.9994

Time Point = 9 subtracted from:

Time Difference SE of Adjusted

Point of Means Difference T-Value P-Value

10 0.1011 0.1120 0.9030 0.9804

**Residual Plots for Cq Prime**

**Main Effects Plot for Cq Prime**

45E3:

**————— 9/11/2012 7:56:24 PM ————————————————————**

**General Linear Model: Cq Prime versus Biounit, Time Point**

Factor Type Levels Values

Biounit random 3 1, 2, 3

Time Point fixed 8 3, 4, 5, 6, 7, 8, 9, 10

Analysis of Variance for Cq Prime, using Adjusted SS for Tests

Source DF Seq SS Adj SS Adj MS F P

Biounit 2 0.02654 0.01375 0.00688 0.36 0.702

Time Point 7 0.09475 0.09475 0.01354 0.72 0.661

Error 13 0.24590 0.24590 0.01892

Total 22 0.36719

S = 0.137533 R-Sq = 33.03% R-Sq(adj) = 0.00%

Term Coef SE Coef T P

Constant -0.16366 0.02906 -5.63 0.000

Biounit

1 -0.03302 0.04244 -0.78 0.451

2 0.00449 0.04041 0.11 0.913

Time Point

3 0.14715 0.09097 1.62 0.130

4 0.07459 0.07465 1.00 0.336

5 -0.05171 0.07465 -0.69 0.501

6 -0.04912 0.07465 -0.66 0.522

7 -0.00171 0.07465 -0.02 0.982

8 -0.02875 0.07465 -0.39 0.706

9 -0.00912 0.07465 -0.12 0.905

Unusual Observations for Cq Prime

Obs Cq Prime Fit SE Fit Residual St Resid

7 -0.473889 -0.210876 0.089094 -0.263013 -2.51 R

R denotes an observation with a large standardized residual.

Expected Mean Squares, using Adjusted SS

Expected Mean Square

Source for Each Term

1 Biounit (3) + 7.5000 (1)

2 Time Point (3) + Q[2]

3 Error (3)

Error Terms for Tests, using Adjusted SS

Synthesis

Source Error DF Error MS of Error MS

1 Biounit 13.00 0.01892 (3)

2 Time Point 13.00 0.01892 (3)

Variance Components, using Adjusted SS

Estimated

Source Value

Biounit -0.00161

Error 0.01892

Grouping Information Using Bonferroni Method and 95.0% Confidence

Time

Point N Mean Grouping

3 2 -0.0165 A

4 3 -0.0891 A

7 3 -0.1654 A

9 3 -0.1728 A

8 3 -0.1924 A

6 3 -0.2128 A

5 3 -0.2154 A

10 3 -0.2450 A

Means that do not share a letter are significantly different.

Bonferroni 95.0% Simultaneous Confidence Intervals

Response Variable Cq Prime

All Pairwise Comparisons among Levels of Time Point

Time Point = 3 subtracted from:

Time

Point Lower Center Upper -+---------+---------+---------+-----

4 -0.5707 -0.0726 0.4255 (-------------*-------------)

5 -0.6970 -0.1989 0.2992 (-------------*--------------)

6 -0.6944 -0.1963 0.3018 (-------------*--------------)

7 -0.6470 -0.1489 0.3492 (-------------*-------------)

8 -0.6740 -0.1759 0.3222 (-------------*-------------)

9 -0.6544 -0.1563 0.3418 (--------------*-------------)

10 -0.7266 -0.2285 0.2696 (-------------*--------------)

-+---------+---------+---------+-----

-0.70 -0.35 0.00 0.35

Time Point = 4 subtracted from:

Time

Point Lower Center Upper -+---------+---------+---------+-----

5 -0.5656 -0.1263 0.3130 (-----------*------------)

6 -0.5630 -0.1237 0.3156 (-----------*------------)

7 -0.5156 -0.0763 0.3630 (------------*-----------)

8 -0.5426 -0.1033 0.3359 (------------*------------)

9 -0.5230 -0.0837 0.3556 (------------*-----------)

10 -0.5952 -0.1559 0.2834 (------------*-----------)

-+---------+---------+---------+-----

-0.70 -0.35 0.00 0.35

Time Point = 5 subtracted from:

Time

Point Lower Center Upper -+---------+---------+---------+-----

6 -0.4367 0.00259 0.4419 (-----------*------------)

7 -0.3893 0.05000 0.4893 (-----------*------------)

8 -0.4163 0.02296 0.4622 (------------*-----------)

9 -0.3967 0.04259 0.4819 (-----------*------------)

10 -0.4689 -0.02963 0.4097 (-----------*------------)

-+---------+---------+---------+-----

-0.70 -0.35 0.00 0.35

Time Point = 6 subtracted from:

Time

Point Lower Center Upper -+---------+---------+---------+-----

7 -0.3919 0.04741 0.4867 (-----------*------------)

8 -0.4189 0.02037 0.4597 (------------*-----------)

9 -0.3993 0.04000 0.4793 (-----------*------------)

10 -0.4715 -0.03222 0.4071 (-----------*------------)

-+---------+---------+---------+-----

-0.70 -0.35 0.00 0.35

Time Point = 7 subtracted from:

Time

Point Lower Center Upper -+---------+---------+---------+-----

8 -0.4663 -0.02704 0.4122 (-----------*------------)

9 -0.4467 -0.00741 0.4319 (------------*-----------)

10 -0.5189 -0.07963 0.3597 (------------*-----------)

-+---------+---------+---------+-----

-0.70 -0.35 0.00 0.35

Time Point = 8 subtracted from:

Time

Point Lower Center Upper -+---------+---------+---------+-----

9 -0.4197 0.01963 0.4589 (------------*-----------)

10 -0.4919 -0.05259 0.3867 (-----------*------------)

-+---------+---------+---------+-----

-0.70 -0.35 0.00 0.35

Time Point = 9 subtracted from:

Time

Point Lower Center Upper -+---------+---------+---------+-----

10 -0.5115 -0.07222 0.3671 (------------*-----------)

-+---------+---------+---------+-----

-0.70 -0.35 0.00 0.35

Bonferroni Simultaneous Tests

Response Variable Cq Prime

All Pairwise Comparisons among Levels of Time Point

Time Point = 3 subtracted from:

Time Difference SE of Adjusted

Point of Means Difference T-Value P-Value

4 -0.0726 0.1273 -0.570 1.000

5 -0.1989 0.1273 -1.562 1.000

6 -0.1963 0.1273 -1.541 1.000

7 -0.1489 0.1273 -1.169 1.000

8 -0.1759 0.1273 -1.381 1.000

9 -0.1563 0.1273 -1.227 1.000

10 -0.2285 0.1273 -1.794 1.000

Time Point = 4 subtracted from:

Time Difference SE of Adjusted

Point of Means Difference T-Value P-Value

5 -0.1263 0.1123 -1.125 1.000

6 -0.1237 0.1123 -1.102 1.000

7 -0.0763 0.1123 -0.679 1.000

8 -0.1033 0.1123 -0.920 1.000

9 -0.0837 0.1123 -0.745 1.000

10 -0.1559 0.1123 -1.389 1.000

Time Point = 5 subtracted from:

Time Difference SE of Adjusted

Point of Means Difference T-Value P-Value

6 0.00259 0.1123 0.0231 1.000

7 0.05000 0.1123 0.4453 1.000

8 0.02296 0.1123 0.2045 1.000

9 0.04259 0.1123 0.3793 1.000

10 -0.02963 0.1123 -0.2639 1.000

Time Point = 6 subtracted from:

Time Difference SE of Adjusted

Point of Means Difference T-Value P-Value

7 0.04741 0.1123 0.4222 1.000

8 0.02037 0.1123 0.1814 1.000

9 0.04000 0.1123 0.3562 1.000

10 -0.03222 0.1123 -0.2869 1.000

Time Point = 7 subtracted from:

Time Difference SE of Adjusted

Point of Means Difference T-Value P-Value

8 -0.02704 0.1123 -0.2408 1.000

9 -0.00741 0.1123 -0.0660 1.000

10 -0.07963 0.1123 -0.7091 1.000

Time Point = 8 subtracted from:

Time Difference SE of Adjusted

Point of Means Difference T-Value P-Value

9 0.01963 0.1123 0.1748 1.000

10 -0.05259 0.1123 -0.4683 1.000

Time Point = 9 subtracted from:

Time Difference SE of Adjusted

Point of Means Difference T-Value P-Value

10 -0.07222 0.1123 -0.6431 1.000

Grouping Information Using Sidak Method and 95.0% Confidence

Time

Point N Mean Grouping

3 2 -0.0165 A

4 3 -0.0891 A

7 3 -0.1654 A

9 3 -0.1728 A

8 3 -0.1924 A

6 3 -0.2128 A

5 3 -0.2154 A

10 3 -0.2450 A

Means that do not share a letter are significantly different.

Sidak 95.0% Simultaneous Confidence Intervals

Response Variable Cq Prime

All Pairwise Comparisons among Levels of Time Point

Time Point = 3 subtracted from:

Time

Point Lower Center Upper -+---------+---------+---------+-----

4 -0.5690 -0.0726 0.4239 (-------------*-------------)

5 -0.6953 -0.1989 0.2976 (-------------*--------------)

6 -0.6927 -0.1963 0.3002 (-------------*--------------)

7 -0.6453 -0.1489 0.3476 (-------------*-------------)

8 -0.6723 -0.1759 0.3205 (-------------*-------------)

9 -0.6527 -0.1563 0.3402 (--------------*-------------)

10 -0.7249 -0.2285 0.2680 (-------------*--------------)

-+---------+---------+---------+-----

-0.70 -0.35 0.00 0.35

Time Point = 4 subtracted from:

Time

Point Lower Center Upper -+---------+---------+---------+-----

5 -0.5641 -0.1263 0.3115 (-----------*------------)

6 -0.5615 -0.1237 0.3141 (-----------*------------)

7 -0.5141 -0.0763 0.3615 (------------*-----------)

8 -0.5412 -0.1033 0.3345 (-----------*------------)

9 -0.5215 -0.0837 0.3541 (------------*-----------)

10 -0.5937 -0.1559 0.2819 (------------*-----------)

-+---------+---------+---------+-----

-0.70 -0.35 0.00 0.35

Time Point = 5 subtracted from:

Time

Point Lower Center Upper -+---------+---------+---------+-----

6 -0.4352 0.00259 0.4404 (-----------*------------)

7 -0.3878 0.05000 0.4878 (-----------*------------)

8 -0.4149 0.02296 0.4608 (------------*-----------)

9 -0.3952 0.04259 0.4804 (-----------*------------)

10 -0.4675 -0.02963 0.4082 (-----------*------------)

-+---------+---------+---------+-----

-0.70 -0.35 0.00 0.35

Time Point = 6 subtracted from:

Time

Point Lower Center Upper -+---------+---------+---------+-----

7 -0.3904 0.04741 0.4852 (-----------*------------)

8 -0.4175 0.02037 0.4582 (------------*-----------)

9 -0.3978 0.04000 0.4778 (-----------*------------)

10 -0.4700 -0.03222 0.4056 (-----------*------------)

-+---------+---------+---------+-----

-0.70 -0.35 0.00 0.35

Time Point = 7 subtracted from:

Time

Point Lower Center Upper -+---------+---------+---------+-----

8 -0.4649 -0.02704 0.4108 (-----------*------------)

9 -0.4452 -0.00741 0.4304 (------------*-----------)

10 -0.5175 -0.07963 0.3582 (------------*-----------)

-+---------+---------+---------+-----

-0.70 -0.35 0.00 0.35

Time Point = 8 subtracted from:

Time

Point Lower Center Upper -+---------+---------+---------+-----

9 -0.4182 0.01963 0.4575 (------------*-----------)

10 -0.4904 -0.05259 0.3852 (-----------*------------)

-+---------+---------+---------+-----

-0.70 -0.35 0.00 0.35

Time Point = 9 subtracted from:

Time

Point Lower Center Upper -+---------+---------+---------+-----

10 -0.5100 -0.07222 0.3656 (------------*-----------)

-+---------+---------+---------+-----

-0.70 -0.35 0.00 0.35

Sidak Simultaneous Tests

Response Variable Cq Prime

All Pairwise Comparisons among Levels of Time Point

Time Point = 3 subtracted from:

Time Difference SE of Adjusted

Point of Means Difference T-Value P-Value

4 -0.0726 0.1273 -0.570 1.0000

5 -0.1989 0.1273 -1.562 0.9864

6 -0.1963 0.1273 -1.541 0.9884

7 -0.1489 0.1273 -1.169 0.9998

8 -0.1759 0.1273 -1.381 0.9973

9 -0.1563 0.1273 -1.227 0.9996

10 -0.2285 0.1273 -1.794 0.9408

Time Point = 4 subtracted from:

Time Difference SE of Adjusted

Point of Means Difference T-Value P-Value

5 -0.1263 0.1123 -1.125 0.9999

6 -0.1237 0.1123 -1.102 0.9999

7 -0.0763 0.1123 -0.679 1.0000

8 -0.1033 0.1123 -0.920 1.0000

9 -0.0837 0.1123 -0.745 1.0000

10 -0.1559 0.1123 -1.389 0.9971

Time Point = 5 subtracted from:

Time Difference SE of Adjusted

Point of Means Difference T-Value P-Value

6 0.00259 0.1123 0.0231 1.000

7 0.05000 0.1123 0.4453 1.000

8 0.02296 0.1123 0.2045 1.000

9 0.04259 0.1123 0.3793 1.000

10 -0.02963 0.1123 -0.2639 1.000

Time Point = 6 subtracted from:

Time Difference SE of Adjusted

Point of Means Difference T-Value P-Value

7 0.04741 0.1123 0.4222 1.000

8 0.02037 0.1123 0.1814 1.000

9 0.04000 0.1123 0.3562 1.000

10 -0.03222 0.1123 -0.2869 1.000

Time Point = 7 subtracted from:

Time Difference SE of Adjusted

Point of Means Difference T-Value P-Value

8 -0.02704 0.1123 -0.2408 1.000

9 -0.00741 0.1123 -0.0660 1.000

10 -0.07963 0.1123 -0.7091 1.000

Time Point = 8 subtracted from:

Time Difference SE of Adjusted

Point of Means Difference T-Value P-Value

9 0.01963 0.1123 0.1748 1.000

10 -0.05259 0.1123 -0.4683 1.000

Time Point = 9 subtracted from:

Time Difference SE of Adjusted

Point of Means Difference T-Value P-Value

10 -0.07222 0.1123 -0.6431 1.000

Grouping Information Using Tukey Method and 95.0% Confidence

Time

Point N Mean Grouping

3 2 -0.0165 A

4 3 -0.0891 A

7 3 -0.1654 A

9 3 -0.1728 A

8 3 -0.1924 A

6 3 -0.2128 A

5 3 -0.2154 A

10 3 -0.2450 A

Means that do not share a letter are significantly different.

Tukey 95.0% Simultaneous Confidence Intervals

Response Variable Cq Prime

All Pairwise Comparisons among Levels of Time Point

Time Point = 3 subtracted from:

Time

Point Lower Center Upper +---------+---------+---------+------

4 -0.5273 -0.0726 0.3821 (------------*------------)

5 -0.6535 -0.1989 0.2558 (------------*------------)

6 -0.6510 -0.1963 0.2584 (------------*------------)

7 -0.6035 -0.1489 0.3058 (------------*------------)

8 -0.6306 -0.1759 0.2788 (------------*------------)

9 -0.6110 -0.1563 0.2984 (------------*------------)

10 -0.6832 -0.2285 0.2262 (------------*------------)

+---------+---------+---------+------

-0.70 -0.35 0.00 0.35

Time Point = 4 subtracted from:

Time

Point Lower Center Upper +---------+---------+---------+------

5 -0.5273 -0.1263 0.2747 (----------*-----------)

6 -0.5247 -0.1237 0.2773 (----------*-----------)

7 -0.4773 -0.0763 0.3247 (-----------*----------)

8 -0.5043 -0.1033 0.2977 (----------*-----------)

9 -0.4847 -0.0837 0.3173 (-----------*----------)

10 -0.5569 -0.1559 0.2451 (-----------*----------)

+---------+---------+---------+------

-0.70 -0.35 0.00 0.35

Time Point = 5 subtracted from:

Time

Point Lower Center Upper +---------+---------+---------+------

6 -0.3984 0.00259 0.4036 (----------*-----------)

7 -0.3510 0.05000 0.4510 (----------*-----------)

8 -0.3780 0.02296 0.4240 (-----------*----------)

9 -0.3584 0.04259 0.4436 (----------*-----------)

10 -0.4306 -0.02963 0.3714 (----------*-----------)

+---------+---------+---------+------

-0.70 -0.35 0.00 0.35

Time Point = 6 subtracted from:

Time

Point Lower Center Upper +---------+---------+---------+------

7 -0.3536 0.04741 0.4484 (----------*-----------)

8 -0.3806 0.02037 0.4214 (-----------*----------)

9 -0.3610 0.04000 0.4410 (----------*-----------)

10 -0.4332 -0.03222 0.3688 (----------*-----------)

+---------+---------+---------+------

-0.70 -0.35 0.00 0.35

Time Point = 7 subtracted from:

Time

Point Lower Center Upper +---------+---------+---------+------

8 -0.4280 -0.02704 0.3740 (----------*-----------)

9 -0.4084 -0.00741 0.3936 (-----------*----------)

10 -0.4806 -0.07963 0.3214 (-----------*----------)

+---------+---------+---------+------

-0.70 -0.35 0.00 0.35

Time Point = 8 subtracted from:

Time

Point Lower Center Upper +---------+---------+---------+------

9 -0.3814 0.01963 0.4206 (-----------*----------)

10 -0.4536 -0.05259 0.3484 (----------*-----------)

+---------+---------+---------+------

-0.70 -0.35 0.00 0.35

Time Point = 9 subtracted from:

Time

Point Lower Center Upper +---------+---------+---------+------

10 -0.4732 -0.07222 0.3288 (-----------*----------)

+---------+---------+---------+------

-0.70 -0.35 0.00 0.35

Tukey Simultaneous Tests

Response Variable Cq Prime

All Pairwise Comparisons among Levels of Time Point

Time Point = 3 subtracted from:

Time Difference SE of Adjusted

Point of Means Difference T-Value P-Value

4 -0.0726 0.1273 -0.570 0.9987

5 -0.1989 0.1273 -1.562 0.7635

6 -0.1963 0.1273 -1.541 0.7741

7 -0.1489 0.1273 -1.169 0.9275

8 -0.1759 0.1273 -1.381 0.8505

9 -0.1563 0.1273 -1.227 0.9095

10 -0.2285 0.1273 -1.794 0.6338

Time Point = 4 subtracted from:

Time Difference SE of Adjusted

Point of Means Difference T-Value P-Value

5 -0.1263 0.1123 -1.125 0.9396

6 -0.1237 0.1123 -1.102 0.9453

7 -0.0763 0.1123 -0.679 0.9962

8 -0.1033 0.1123 -0.920 0.9783

9 -0.0837 0.1123 -0.745 0.9934

10 -0.1559 0.1123 -1.389 0.8474

Time Point = 5 subtracted from:

Time Difference SE of Adjusted

Point of Means Difference T-Value P-Value

6 0.00259 0.1123 0.0231 1.0000

7 0.05000 0.1123 0.4453 0.9997

8 0.02296 0.1123 0.2045 1.0000

9 0.04259 0.1123 0.3793 0.9999

10 -0.02963 0.1123 -0.2639 1.0000

Time Point = 6 subtracted from:

Time Difference SE of Adjusted

Point of Means Difference T-Value P-Value

7 0.04741 0.1123 0.4222 0.9998

8 0.02037 0.1123 0.1814 1.0000

9 0.04000 0.1123 0.3562 0.9999

10 -0.03222 0.1123 -0.2869 1.0000

Time Point = 7 subtracted from:

Time Difference SE of Adjusted

Point of Means Difference T-Value P-Value

8 -0.02704 0.1123 -0.2408 1.0000

9 -0.00741 0.1123 -0.0660 1.0000

10 -0.07963 0.1123 -0.7091 0.9951

Time Point = 8 subtracted from:

Time Difference SE of Adjusted

Point of Means Difference T-Value P-Value

9 0.01963 0.1123 0.1748 1.0000

10 -0.05259 0.1123 -0.4683 0.9996

Time Point = 9 subtracted from:

Time Difference SE of Adjusted

Point of Means Difference T-Value P-Value

10 -0.07222 0.1123 -0.6431 0.9973

**Residual Plots for Cq Prime**

**Main Effects Plot for Cq Prime**

53B6:

**General Linear Model: Cq Prime versus Biounit, Time Point**

Factor Type Levels Values

Biounit random 3 1, 2, 3

Time Point fixed 8 3, 4, 5, 6, 7, 8, 9, 10

Analysis of Variance for Cq Prime, using Adjusted SS for Tests

Source DF Seq SS Adj SS Adj MS F P

Biounit 2 0.14575 0.13562 0.06781 4.11 0.041

Time Point 7 0.05295 0.05295 0.00756 0.46 0.848

Error 13 0.21451 0.21451 0.01650

Total 22 0.41321

S = 0.128457 R-Sq = 48.09% R-Sq(adj) = 12.15%

Term Coef SE Coef T P

Constant 0.07456 0.02714 2.75 0.017

Biounit

1 0.11302 0.03964 2.85 0.014

2 -0.06623 0.03774 -1.75 0.103

Time Point

3 -0.01806 0.08497 -0.21 0.835

4 -0.04975 0.06973 -0.71 0.488

5 0.10840 0.06973 1.55 0.144

6 0.02655 0.06973 0.38 0.710

7 -0.04271 0.06973 -0.61 0.551

8 -0.01530 0.06973 -0.22 0.830

9 0.00988 0.06973 0.14 0.889

Unusual Observations for Cq Prime

Obs Cq Prime Fit SE Fit Residual St Resid

13 0.185556 -0.034378 0.083214 0.219934 2.25 R

14 -0.261111 -0.014934 0.083214 -0.246177 -2.52 R

R denotes an observation with a large standardized residual.

Expected Mean Squares, using Adjusted SS

Expected Mean Square

Source for Each Term

1 Biounit (3) + 7.5000 (1)

2 Time Point (3) + Q[2]

3 Error (3)

Error Terms for Tests, using Adjusted SS

Synthesis

Source Error DF Error MS of Error MS

1 Biounit 13.00 0.01650 (3)

2 Time Point 13.00 0.01650 (3)

Variance Components, using Adjusted SS

Estimated

Source Value

Biounit 0.00684

Error 0.01650

Grouping Information Using Bonferroni Method and 95.0% Confidence

Time

Point N Mean Grouping

5 3 0.18296 A

6 3 0.10111 A

9 3 0.08444 A

8 3 0.05926 A

3 2 0.05651 A

10 3 0.05556 A

7 3 0.03185 A

4 3 0.02481 A

Means that do not share a letter are significantly different.

Bonferroni 95.0% Simultaneous Confidence Intervals

Response Variable Cq Prime

All Pairwise Comparisons among Levels of Time Point

Time Point = 3 subtracted from:

Time

Point Lower Center Upper ------+---------+---------+---------+

4 -0.4969 -0.03169 0.4335 (------------*------------)

5 -0.3388 0.12646 0.5917 (-------------*------------)

6 -0.4206 0.04460 0.5098 (------------*-------------)

7 -0.4899 -0.02466 0.4406 (------------*-------------)

8 -0.4625 0.00275 0.4680 (------------*------------)

9 -0.4373 0.02794 0.4932 (------------*------------)

10 -0.4662 -0.00095 0.4643 (------------*------------)

------+---------+---------+---------+

-0.35 0.00 0.35 0.70

Time Point = 4 subtracted from:

Time

Point Lower Center Upper ------+---------+---------+---------+

5 -0.2521 0.158148 0.5684 (-----------*----------)

6 -0.3340 0.076296 0.4866 (-----------*-----------)

7 -0.4033 0.007037 0.4173 (-----------*-----------)

8 -0.3758 0.034444 0.4447 (-----------*-----------)

9 -0.3507 0.059630 0.4699 (-----------*----------)

10 -0.3796 0.030741 0.4410 (-----------*-----------)

------+---------+---------+---------+

-0.35 0.00 0.35 0.70

Time Point = 5 subtracted from:

Time

Point Lower Center Upper ------+---------+---------+---------+

6 -0.4921 -0.0819 0.3284 (-----------*----------)

7 -0.5614 -0.1511 0.2592 (-----------*----------)

8 -0.5340 -0.1237 0.2866 (----------*-----------)

9 -0.5088 -0.0985 0.3118 (-----------*-----------)

10 -0.5377 -0.1274 0.2829 (----------*-----------)

------+---------+---------+---------+

-0.35 0.00 0.35 0.70

Time Point = 6 subtracted from:

Time

Point Lower Center Upper ------+---------+---------+---------+

7 -0.4796 -0.06926 0.3410 (-----------*-----------)

8 -0.4521 -0.04185 0.3684 (-----------*-----------)

9 -0.4270 -0.01667 0.3936 (-----------*----------)

10 -0.4558 -0.04556 0.3647 (-----------*----------)

------+---------+---------+---------+

-0.35 0.00 0.35 0.70

Time Point = 7 subtracted from:

Time

Point Lower Center Upper ------+---------+---------+---------+

8 -0.3829 0.02741 0.4377 (-----------*-----------)

9 -0.3577 0.05259 0.4629 (-----------*----------)

10 -0.3866 0.02370 0.4340 (-----------*----------)

------+---------+---------+---------+

-0.35 0.00 0.35 0.70

Time Point = 8 subtracted from:

Time

Point Lower Center Upper ------+---------+---------+---------+

9 -0.3851 0.025185 0.4355 (-----------*----------)

10 -0.4140 -0.003704 0.4066 (-----------*-----------)

------+---------+---------+---------+

-0.35 0.00 0.35 0.70

Time Point = 9 subtracted from:

Time

Point Lower Center Upper ------+---------+---------+---------+

10 -0.4392 -0.02889 0.3814 (-----------*-----------)

------+---------+---------+---------+

-0.35 0.00 0.35 0.70

Bonferroni Simultaneous Tests

Response Variable Cq Prime

All Pairwise Comparisons among Levels of Time Point

Time Point = 3 subtracted from:

Time Difference SE of Adjusted

Point of Means Difference T-Value P-Value

4 -0.03169 0.1189 -0.2665 1.000

5 0.12646 0.1189 1.0633 1.000

6 0.04460 0.1189 0.3750 1.000

7 -0.02466 0.1189 -0.2073 1.000

8 0.00275 0.1189 0.0231 1.000

9 0.02794 0.1189 0.2349 1.000

10 -0.00095 0.1189 -0.0080 1.000

Time Point = 4 subtracted from:

Time Difference SE of Adjusted

Point of Means Difference T-Value P-Value

5 0.158148 0.1049 1.50783 1.000

6 0.076296 0.1049 0.72743 1.000

7 0.007037 0.1049 0.06709 1.000

8 0.034444 0.1049 0.32840 1.000

9 0.059630 0.1049 0.56853 1.000

10 0.030741 0.1049 0.29309 1.000

Time Point = 5 subtracted from:

Time Difference SE of Adjusted

Point of Means Difference T-Value P-Value

6 -0.0819 0.1049 -0.780 1.000

7 -0.1511 0.1049 -1.441 1.000

8 -0.1237 0.1049 -1.179 1.000

9 -0.0985 0.1049 -0.939 1.000

10 -0.1274 0.1049 -1.215 1.000

Time Point = 6 subtracted from:

Time Difference SE of Adjusted

Point of Means Difference T-Value P-Value

7 -0.06926 0.1049 -0.6603 1.000

8 -0.04185 0.1049 -0.3990 1.000

9 -0.01667 0.1049 -0.1589 1.000

10 -0.04556 0.1049 -0.4343 1.000

Time Point = 7 subtracted from:

Time Difference SE of Adjusted

Point of Means Difference T-Value P-Value

8 0.02741 0.1049 0.2613 1.000

9 0.05259 0.1049 0.5014 1.000

10 0.02370 0.1049 0.2260 1.000

Time Point = 8 subtracted from:

Time Difference SE of Adjusted

Point of Means Difference T-Value P-Value

9 0.025185 0.1049 0.24012 1.000

10 -0.003704 0.1049 -0.03531 1.000

Time Point = 9 subtracted from:

Time Difference SE of Adjusted

Point of Means Difference T-Value P-Value

10 -0.02889 0.1049 -0.2754 1.000

Grouping Information Using Sidak Method and 95.0% Confidence

Time

Point N Mean Grouping

5 3 0.18296 A

6 3 0.10111 A

9 3 0.08444 A

8 3 0.05926 A

3 2 0.05651 A

10 3 0.05556 A

7 3 0.03185 A

4 3 0.02481 A

Means that do not share a letter are significantly different.

Sidak 95.0% Simultaneous Confidence Intervals

Response Variable Cq Prime

All Pairwise Comparisons among Levels of Time Point

Time Point = 3 subtracted from:

Time

Point Lower Center Upper ------+---------+---------+---------+

4 -0.4954 -0.03169 0.4320 (------------*------------)

5 -0.3372 0.12646 0.5901 (-------------*------------)

6 -0.4191 0.04460 0.5083 (------------*-------------)

7 -0.4883 -0.02466 0.4390 (------------*-------------)

8 -0.4609 0.00275 0.4664 (------------*------------)

9 -0.4357 0.02794 0.4916 (------------*------------)

10 -0.4646 -0.00095 0.4627 (------------*------------)

------+---------+---------+---------+

-0.35 0.00 0.35 0.70

Time Point = 4 subtracted from:

Time

Point Lower Center Upper ------+---------+---------+---------+

5 -0.2508 0.158148 0.5671 (-----------*----------)

6 -0.3326 0.076296 0.4852 (-----------*-----------)

7 -0.4019 0.007037 0.4160 (----------*-----------)

8 -0.3745 0.034444 0.4434 (-----------*-----------)

9 -0.3493 0.059630 0.4686 (-----------*----------)

10 -0.3782 0.030741 0.4397 (-----------*-----------)

------+---------+---------+---------+

-0.35 0.00 0.35 0.70

Time Point = 5 subtracted from:

Time

Point Lower Center Upper ------+---------+---------+---------+

6 -0.4908 -0.0819 0.3271 (-----------*----------)

7 -0.5600 -0.1511 0.2578 (-----------*----------)

8 -0.5326 -0.1237 0.2852 (----------*-----------)

9 -0.5074 -0.0985 0.3104 (----------*-----------)

10 -0.5363 -0.1274 0.2815 (----------*-----------)

------+---------+---------+---------+

-0.35 0.00 0.35 0.70

Time Point = 6 subtracted from:

Time

Point Lower Center Upper ------+---------+---------+---------+

7 -0.4782 -0.06926 0.3397 (-----------*-----------)

8 -0.4508 -0.04185 0.3671 (-----------*----------)

9 -0.4256 -0.01667 0.3923 (-----------*----------)

10 -0.4545 -0.04556 0.3634 (-----------*----------)

------+---------+---------+---------+

-0.35 0.00 0.35 0.70

Time Point = 7 subtracted from:

Time

Point Lower Center Upper ------+---------+---------+---------+

8 -0.3815 0.02741 0.4363 (-----------*----------)

9 -0.3563 0.05259 0.4615 (-----------*----------)

10 -0.3852 0.02370 0.4326 (-----------*----------)

------+---------+---------+---------+

-0.35 0.00 0.35 0.70

Time Point = 8 subtracted from:

Time

Point Lower Center Upper ------+---------+---------+---------+

9 -0.3837 0.025185 0.4341 (-----------*----------)

10 -0.4126 -0.003704 0.4052 (-----------*-----------)

------+---------+---------+---------+

-0.35 0.00 0.35 0.70

Time Point = 9 subtracted from:

Time

Point Lower Center Upper ------+---------+---------+---------+

10 -0.4378 -0.02889 0.3800 (-----------*-----------)

------+---------+---------+---------+

-0.35 0.00 0.35 0.70

Sidak Simultaneous Tests

Response Variable Cq Prime

All Pairwise Comparisons among Levels of Time Point

Time Point = 3 subtracted from:

Time Difference SE of Adjusted

Point of Means Difference T-Value P-Value

4 -0.03169 0.1189 -0.2665 1.000

5 0.12646 0.1189 1.0633 1.000

6 0.04460 0.1189 0.3750 1.000

7 -0.02466 0.1189 -0.2073 1.000

8 0.00275 0.1189 0.0231 1.000

9 0.02794 0.1189 0.2349 1.000

10 -0.00095 0.1189 -0.0080 1.000

Time Point = 4 subtracted from:

Time Difference SE of Adjusted

Point of Means Difference T-Value P-Value

5 0.158148 0.1049 1.50783 0.9912

6 0.076296 0.1049 0.72743 1.0000

7 0.007037 0.1049 0.06709 1.0000

8 0.034444 0.1049 0.32840 1.0000

9 0.059630 0.1049 0.56853 1.0000

10 0.030741 0.1049 0.29309 1.0000

Time Point = 5 subtracted from:

Time Difference SE of Adjusted

Point of Means Difference T-Value P-Value

6 -0.0819 0.1049 -0.780 1.0000

7 -0.1511 0.1049 -1.441 0.9952

8 -0.1237 0.1049 -1.179 0.9998

9 -0.0985 0.1049 -0.939 1.0000

10 -0.1274 0.1049 -1.215 0.9996

Time Point = 6 subtracted from:

Time Difference SE of Adjusted

Point of Means Difference T-Value P-Value

7 -0.06926 0.1049 -0.6603 1.000

8 -0.04185 0.1049 -0.3990 1.000

9 -0.01667 0.1049 -0.1589 1.000

10 -0.04556 0.1049 -0.4343 1.000

Time Point = 7 subtracted from:

Time Difference SE of Adjusted

Point of Means Difference T-Value P-Value

8 0.02741 0.1049 0.2613 1.000

9 0.05259 0.1049 0.5014 1.000

10 0.02370 0.1049 0.2260 1.000

Time Point = 8 subtracted from:

Time Difference SE of Adjusted

Point of Means Difference T-Value P-Value

9 0.025185 0.1049 0.24012 1.000

10 -0.003704 0.1049 -0.03531 1.000

Time Point = 9 subtracted from:

Time Difference SE of Adjusted

Point of Means Difference T-Value P-Value

10 -0.02889 0.1049 -0.2754 1.000

Grouping Information Using Tukey Method and 95.0% Confidence

Time

Point N Mean Grouping

5 3 0.18296 A

6 3 0.10111 A

9 3 0.08444 A

8 3 0.05926 A

3 2 0.05651 A

10 3 0.05556 A

7 3 0.03185 A

4 3 0.02481 A

Means that do not share a letter are significantly different.

Tukey 95.0% Simultaneous Confidence Intervals

Response Variable Cq Prime

All Pairwise Comparisons among Levels of Time Point

Time Point = 3 subtracted from:

Time

Point Lower Center Upper --------+---------+---------+--------

4 -0.4564 -0.03169 0.3930 (-------------*-------------)

5 -0.2982 0.12646 0.5511 (-------------*-------------)

6 -0.3801 0.04460 0.4693 (-------------*--------------)

7 -0.4493 -0.02466 0.4000 (-------------*-------------)

8 -0.4219 0.00275 0.4274 (-------------*-------------)

9 -0.3967 0.02794 0.4526 (-------------*-------------)

10 -0.4256 -0.00095 0.4237 (-------------*-------------)

--------+---------+---------+--------

-0.30 0.00 0.30

Time Point = 4 subtracted from:

Time

Point Lower Center Upper --------+---------+---------+--------

5 -0.2164 0.158148 0.5327 (-----------*------------)

6 -0.2982 0.076296 0.4508 (------------*-----------)

7 -0.3675 0.007037 0.3816 (-----------*------------)

8 -0.3401 0.034444 0.4090 (-----------*------------)

9 -0.3149 0.059630 0.4342 (-----------*-----------)

10 -0.3438 0.030741 0.4053 (-----------*------------)

--------+---------+---------+--------

-0.30 0.00 0.30

Time Point = 5 subtracted from:

Time

Point Lower Center Upper --------+---------+---------+--------

6 -0.4564 -0.0819 0.2927 (-----------*------------)

7 -0.5256 -0.1511 0.2234 (------------*-----------)

8 -0.4982 -0.1237 0.2508 (------------*-----------)

9 -0.4730 -0.0985 0.2760 (------------*-----------)

10 -0.5019 -0.1274 0.2471 (------------*-----------)

--------+---------+---------+--------

-0.30 0.00 0.30

Time Point = 6 subtracted from:

Time

Point Lower Center Upper --------+---------+---------+--------

7 -0.4438 -0.06926 0.3053 (------------*-----------)

8 -0.4164 -0.04185 0.3327 (------------*-----------)

9 -0.3912 -0.01667 0.3579 (-----------*------------)

10 -0.4201 -0.04556 0.3290 (-----------*------------)

--------+---------+---------+--------

-0.30 0.00 0.30

Time Point = 7 subtracted from:

Time

Point Lower Center Upper --------+---------+---------+--------

8 -0.3471 0.02741 0.4019 (------------*-----------)

9 -0.3219 0.05259 0.4271 (------------*-----------)

10 -0.3508 0.02370 0.3982 (------------*-----------)

--------+---------+---------+--------

-0.30 0.00 0.30

Time Point = 8 subtracted from:

Time

Point Lower Center Upper --------+---------+---------+--------

9 -0.3493 0.025185 0.3997 (------------*-----------)

10 -0.3782 -0.003704 0.3708 (------------*-----------)

--------+---------+---------+--------

-0.30 0.00 0.30

Time Point = 9 subtracted from:

Time

Point Lower Center Upper --------+---------+---------+--------

10 -0.4034 -0.02889 0.3456 (-----------*------------)

--------+---------+---------+--------

-0.30 0.00 0.30

Tukey Simultaneous Tests

Response Variable Cq Prime

All Pairwise Comparisons among Levels of Time Point

Time Point = 3 subtracted from:

Time Difference SE of Adjusted

Point of Means Difference T-Value P-Value

4 -0.03169 0.1189 -0.2665 1.0000

5 0.12646 0.1189 1.0633 0.9541

6 0.04460 0.1189 0.3750 0.9999

7 -0.02466 0.1189 -0.2073 1.0000

8 0.00275 0.1189 0.0231 1.0000

9 0.02794 0.1189 0.2349 1.0000

10 -0.00095 0.1189 -0.0080 1.0000

Time Point = 4 subtracted from:

Time Difference SE of Adjusted

Point of Means Difference T-Value P-Value

5 0.158148 0.1049 1.50783 0.7912

6 0.076296 0.1049 0.72743 0.9943

7 0.007037 0.1049 0.06709 1.0000

8 0.034444 0.1049 0.32840 1.0000

9 0.059630 0.1049 0.56853 0.9987

10 0.030741 0.1049 0.29309 1.0000

Time Point = 5 subtracted from:

Time Difference SE of Adjusted

Point of Means Difference T-Value P-Value

6 -0.0819 0.1049 -0.780 0.9914

7 -0.1511 0.1049 -1.441 0.8238

8 -0.1237 0.1049 -1.179 0.9244

9 -0.0985 0.1049 -0.939 0.9758

10 -0.1274 0.1049 -1.215 0.9135

Time Point = 6 subtracted from:

Time Difference SE of Adjusted

Point of Means Difference T-Value P-Value

7 -0.06926 0.1049 -0.6603 0.9968

8 -0.04185 0.1049 -0.3990 0.9999

9 -0.01667 0.1049 -0.1589 1.0000

10 -0.04556 0.1049 -0.4343 0.9998

Time Point = 7 subtracted from:

Time Difference SE of Adjusted

Point of Means Difference T-Value P-Value

8 0.02741 0.1049 0.2613 1.0000

9 0.05259 0.1049 0.5014 0.9994

10 0.02370 0.1049 0.2260 1.0000

Time Point = 8 subtracted from:

Time Difference SE of Adjusted

Point of Means Difference T-Value P-Value

9 0.025185 0.1049 0.24012 1.000

10 -0.003704 0.1049 -0.03531 1.000

Time Point = 9 subtracted from:

Time Difference SE of Adjusted

Point of Means Difference T-Value P-Value

10 -0.02889 0.1049 -0.2754 1.000

**Residual Plots for Cq Prime**

**Main Effects Plot for Cq Prime**

PSN0001:

**————— 9/11/2012 7:56:24 PM ————————————————————**

**General Linear Model: Cq Prime versus Biounit, Time Point**

Factor Type Levels Values

Biounit random 3 1, 2, 3

Time Point fixed 8 3, 4, 5, 6, 7, 8, 9, 10

Analysis of Variance for Cq Prime, using Adjusted SS for Tests

Source DF Seq SS Adj SS Adj MS F P

Biounit 2 3.81040 4.09252 2.04626 35.32 0.000

Time Point 7 3.30180 3.30180 0.47169 8.14 0.001

Error 13 0.75316 0.75316 0.05794

Total 22 7.86536

S = 0.240698 R-Sq = 90.42% R-Sq(adj) = 83.80%

Term Coef SE Coef T P

Constant -0.05205 0.05086 -1.02 0.325

Biounit

1 0.62270 0.07428 8.38 0.000

2 -0.27503 0.07071 -3.89 0.002

Time Point

3 0.3634 0.1592 2.28 0.040

4 -0.9020 0.1307 -6.90 0.000

5 -0.1950 0.1307 -1.49 0.159

6 0.0276 0.1307 0.21 0.836

7 0.3439 0.1307 2.63 0.021

8 0.1024 0.1307 0.78 0.447

9 0.1743 0.1307 1.33 0.205

Unusual Observations for Cq Prime

Obs Cq Prime Fit SE Fit Residual St Resid

1 0.36000 0.03632 0.18052 0.32368 2.03 R

2 -0.36000 -0.03632 0.18052 -0.32368 -2.03 R

R denotes an observation with a large standardized residual.

Expected Mean Squares, using Adjusted SS

Expected Mean Square

Source for Each Term

1 Biounit (3) + 7.5000 (1)

2 Time Point (3) + Q[2]

3 Error (3)

Error Terms for Tests, using Adjusted SS

Synthesis

Source Error DF Error MS of Error MS

1 Biounit 13.00 0.05794 (3)

2 Time Point 13.00 0.05794 (3)

Variance Components, using Adjusted SS

Estimated

Source Value

Biounit 0.26511

Error 0.05794

Grouping Information Using Bonferroni Method and 95.0% Confidence

Time

Point N Mean Grouping

3 2 0.3113 A

7 3 0.2919 A

9 3 0.1222 A

8 3 0.0504 A

10 3 0.0333 A

6 3 -0.0244 A

5 3 -0.2470 A B

4 3 -0.9541 B

Means that do not share a letter are significantly different.

Bonferroni 95.0% Simultaneous Confidence Intervals

Response Variable Cq Prime

All Pairwise Comparisons among Levels of Time Point

Time Point = 3 subtracted from:

Time

Point Lower Center Upper --------+---------+---------+--------

4 -2.137 -1.265 -0.3937 (------*-------)

5 -1.430 -0.558 0.3133 (------*-------)

6 -1.208 -0.336 0.5359 (------*------)

7 -0.891 -0.019 0.8522 (------*------)

8 -1.133 -0.261 0.6107 (------*------)

9 -1.061 -0.189 0.6826 (------*-------)

10 -1.150 -0.278 0.5937 (-------*------)

--------+---------+---------+--------

-1.2 0.0 1.2

Time Point = 4 subtracted from:

Time

Point Lower Center Upper --------+---------+---------+--------

5 -0.06175 0.7070 1.476 (------*-----)

6 0.16084 0.9296 1.698 (------*-----)

7 0.47714 1.2459 2.015 (-----*------)

8 0.23565 1.0044 1.773 (-----*------)

9 0.30751 1.0763 1.845 (-----*-----)

10 0.21862 0.9874 1.756 (-----*------)

--------+---------+---------+--------

-1.2 0.0 1.2

Time Point = 5 subtracted from:

Time

Point Lower Center Upper --------+---------+---------+--------

6 -0.5462 0.2226 0.9914 (------*-----)

7 -0.2299 0.5389 1.3077 (-----*------)

8 -0.4714 0.2974 1.0662 (-----*------)

9 -0.3995 0.3693 1.1380 (-----*-----)

10 -0.4884 0.2804 1.0492 (-----*------)

--------+---------+---------+--------

-1.2 0.0 1.2

Time Point = 6 subtracted from:

Time

Point Lower Center Upper --------+---------+---------+--------

7 -0.4525 0.31630 1.0851 (------*-----)

8 -0.6940 0.07481 0.8436 (------*-----)

9 -0.6221 0.14667 0.9155 (-----*------)

10 -0.7110 0.05778 0.8266 (-----*------)

--------+---------+---------+--------

-1.2 0.0 1.2

Time Point = 7 subtracted from:

Time

Point Lower Center Upper --------+---------+---------+--------

8 -1.010 -0.2415 0.5273 (-----*-----)

9 -0.938 -0.1696 0.5992 (------*-----)

10 -1.027 -0.2585 0.5103 (------*-----)

--------+---------+---------+--------

-1.2 0.0 1.2

Time Point = 8 subtracted from:

Time

Point Lower Center Upper --------+---------+---------+--------

9 -0.6969 0.07185 0.8406 (------*-----)

10 -0.7858 -0.01704 0.7518 (------*-----)

--------+---------+---------+--------

-1.2 0.0 1.2

Time Point = 9 subtracted from:

Time

Point Lower Center Upper --------+---------+---------+--------

10 -0.8577 -0.08889 0.6799 (-----*------)

--------+---------+---------+--------

-1.2 0.0 1.2

Bonferroni Simultaneous Tests

Response Variable Cq Prime

All Pairwise Comparisons among Levels of Time Point

Time Point = 3 subtracted from:

Time Difference SE of Adjusted

Point of Means Difference T-Value P-Value

4 -1.265 0.2228 -5.679 0.0021

5 -0.558 0.2228 -2.506 0.7365

6 -0.336 0.2228 -1.507 1.0000

7 -0.019 0.2228 -0.087 1.0000

8 -0.261 0.2228 -1.171 1.0000

9 -0.189 0.2228 -0.849 1.0000

10 -0.278 0.2228 -1.248 1.0000

Time Point = 4 subtracted from:

Time Difference SE of Adjusted

Point of Means Difference T-Value P-Value

5 0.7070 0.1965 3.598 0.0909

6 0.9296 0.1965 4.730 0.0110

7 1.2459 0.1965 6.340 0.0007

8 1.0044 0.1965 5.111 0.0056

9 1.0763 0.1965 5.477 0.0030

10 0.9874 0.1965 5.024 0.0065

Time Point = 5 subtracted from:

Time Difference SE of Adjusted

Point of Means Difference T-Value P-Value

6 0.2226 0.1965 1.133 1.0000

7 0.5389 0.1965 2.742 0.4702

8 0.2974 0.1965 1.513 1.0000

9 0.3693 0.1965 1.879 1.0000

10 0.2804 0.1965 1.427 1.0000

Time Point = 6 subtracted from:

Time Difference SE of Adjusted

Point of Means Difference T-Value P-Value

7 0.31630 0.1965 1.6094 1.000

8 0.07481 0.1965 0.3807 1.000

9 0.14667 0.1965 0.7463 1.000

10 0.05778 0.1965 0.2940 1.000

Time Point = 7 subtracted from:

Time Difference SE of Adjusted

Point of Means Difference T-Value P-Value

8 -0.2415 0.1965 -1.229 1.000

9 -0.1696 0.1965 -0.863 1.000

10 -0.2585 0.1965 -1.315 1.000

Time Point = 8 subtracted from:

Time Difference SE of Adjusted

Point of Means Difference T-Value P-Value

9 0.07185 0.1965 0.36560 1.000

10 -0.01704 0.1965 -0.08669 1.000

Time Point = 9 subtracted from:

Time Difference SE of Adjusted

Point of Means Difference T-Value P-Value

10 -0.08889 0.1965 -0.4523 1.000

Grouping Information Using Sidak Method and 95.0% Confidence

Time

Point N Mean Grouping

3 2 0.3113 A

7 3 0.2919 A

9 3 0.1222 A

8 3 0.0504 A

10 3 0.0333 A

6 3 -0.0244 A

5 3 -0.2470 A B

4 3 -0.9541 B

Means that do not share a letter are significantly different.

Sidak 95.0% Simultaneous Confidence Intervals

Response Variable Cq Prime

All Pairwise Comparisons among Levels of Time Point

Time Point = 3 subtracted from:

Time

Point Lower Center Upper --------+---------+---------+--------

4 -2.134 -1.265 -0.3966 (------*-------)

5 -1.427 -0.558 0.3104 (------*-------)

6 -1.205 -0.336 0.5330 (------*------)

7 -0.888 -0.019 0.8493 (------*------)

8 -1.130 -0.261 0.6078 (------*------)

9 -1.058 -0.189 0.6797 (------*-------)

10 -1.147 -0.278 0.5908 (-------*------)

--------+---------+---------+--------

-1.2 0.0 1.2

Time Point = 4 subtracted from:

Time

Point Lower Center Upper --------+---------+---------+--------

5 -0.05919 0.7070 1.473 (-----*-----)

6 0.16340 0.9296 1.696 (------*-----)

7 0.47969 1.2459 2.012 (-----*------)

8 0.23821 1.0044 1.771 (-----*------)

9 0.31006 1.0763 1.843 (-----*-----)

10 0.22118 0.9874 1.754 (-----*------)

--------+---------+---------+--------

-1.2 0.0 1.2

Time Point = 5 subtracted from:

Time

Point Lower Center Upper --------+---------+---------+--------

6 -0.5436 0.2226 0.9888 (------*-----)

7 -0.2273 0.5389 1.3051 (-----*------)

8 -0.4688 0.2974 1.0636 (-----*------)

9 -0.3970 0.3693 1.1355 (-----*-----)

10 -0.4859 0.2804 1.0466 (-----*------)

--------+---------+---------+--------

-1.2 0.0 1.2

Time Point = 6 subtracted from:

Time

Point Lower Center Upper --------+---------+---------+--------

7 -0.4499 0.31630 1.0825 (------*-----)

8 -0.6914 0.07481 0.8410 (------*-----)

9 -0.6196 0.14667 0.9129 (-----*------)

10 -0.7085 0.05778 0.8240 (-----*------)

--------+---------+---------+--------

-1.2 0.0 1.2

Time Point = 7 subtracted from:

Time

Point Lower Center Upper --------+---------+---------+--------

8 -1.008 -0.2415 0.5248 (-----*-----)

9 -0.936 -0.1696 0.5966 (------*-----)

10 -1.025 -0.2585 0.5077 (------*-----)

--------+---------+---------+--------

-1.2 0.0 1.2

Time Point = 8 subtracted from:

Time

Point Lower Center Upper --------+---------+---------+--------

9 -0.6944 0.07185 0.8381 (------*-----)

10 -0.7833 -0.01704 0.7492 (------*-----)

--------+---------+---------+--------

-1.2 0.0 1.2

Time Point = 9 subtracted from:

Time

Point Lower Center Upper --------+---------+---------+--------

10 -0.8551 -0.08889 0.6773 (-----*------)

--------+---------+---------+--------

-1.2 0.0 1.2

Sidak Simultaneous Tests

Response Variable Cq Prime

All Pairwise Comparisons among Levels of Time Point

Time Point = 3 subtracted from:

Time Difference SE of Adjusted

Point of Means Difference T-Value P-Value

4 -1.265 0.2228 -5.679 0.0021

5 -0.558 0.2228 -2.506 0.5259

6 -0.336 0.2228 -1.507 0.9913

7 -0.019 0.2228 -0.087 1.0000

8 -0.261 0.2228 -1.171 0.9998

9 -0.189 0.2228 -0.849 1.0000

10 -0.278 0.2228 -1.248 0.9994

Time Point = 4 subtracted from:

Time Difference SE of Adjusted

Point of Means Difference T-Value P-Value

5 0.7070 0.1965 3.598 0.0870

6 0.9296 0.1965 4.730 0.0110

7 1.2459 0.1965 6.340 0.0007

8 1.0044 0.1965 5.111 0.0056

9 1.0763 0.1965 5.477 0.0030

10 0.9874 0.1965 5.024 0.0065

Time Point = 5 subtracted from:

Time Difference SE of Adjusted

Point of Means Difference T-Value P-Value

6 0.2226 0.1965 1.133 0.9999

7 0.5389 0.1965 2.742 0.3776

8 0.2974 0.1965 1.513 0.9908

9 0.3693 0.1965 1.879 0.9112

10 0.2804 0.1965 1.427 0.9958

Time Point = 6 subtracted from:

Time Difference SE of Adjusted

Point of Means Difference T-Value P-Value

7 0.31630 0.1965 1.6094 0.9807

8 0.07481 0.1965 0.3807 1.0000

9 0.14667 0.1965 0.7463 1.0000

10 0.05778 0.1965 0.2940 1.0000

Time Point = 7 subtracted from:

Time Difference SE of Adjusted

Point of Means Difference T-Value P-Value

8 -0.2415 0.1965 -1.229 0.9996

9 -0.1696 0.1965 -0.863 1.0000

10 -0.2585 0.1965 -1.315 0.9987

Time Point = 8 subtracted from:

Time Difference SE of Adjusted

Point of Means Difference T-Value P-Value

9 0.07185 0.1965 0.36560 1.000

10 -0.01704 0.1965 -0.08669 1.000

Time Point = 9 subtracted from:

Time Difference SE of Adjusted

Point of Means Difference T-Value P-Value

10 -0.08889 0.1965 -0.4523 1.000

Grouping Information Using Tukey Method and 95.0% Confidence

Time

Point N Mean Grouping

3 2 0.3113 A

7 3 0.2919 A

9 3 0.1222 A

8 3 0.0504 A

10 3 0.0333 A

6 3 -0.0244 A

5 3 -0.2470 A

4 3 -0.9541 B

Means that do not share a letter are significantly different.

Tukey 95.0% Simultaneous Confidence Intervals

Response Variable Cq Prime

All Pairwise Comparisons among Levels of Time Point

Time Point = 3 subtracted from:

Time

Point Lower Center Upper -------+---------+---------+---------

4 -2.061 -1.265 -0.4697 (-----*------)

5 -1.354 -0.558 0.2374 (-----*------)

6 -1.132 -0.336 0.4600 (-----*------)

7 -0.815 -0.019 0.7762 (------*-----)

8 -1.057 -0.261 0.5348 (------*-----)

9 -0.985 -0.189 0.6066 (-----*------)

10 -1.074 -0.278 0.5177 (------*-----)

-------+---------+---------+---------

-1.2 0.0 1.2

Time Point = 4 subtracted from:

Time

Point Lower Center Upper -------+---------+---------+---------

5 0.005255 0.7070 1.409 (-----*-----)

6 0.227847 0.9296 1.631 (-----*-----)

7 0.544143 1.2459 1.948 (----*-----)

8 0.302662 1.0044 1.706 (----*-----)

9 0.374514 1.0763 1.778 (-----*-----)

10 0.285625 0.9874 1.689 (-----*-----)

-------+---------+---------+---------

-1.2 0.0 1.2

Time Point = 5 subtracted from:

Time

Point Lower Center Upper -------+---------+---------+---------

6 -0.4792 0.2226 0.9244 (-----*-----)

7 -0.1629 0.5389 1.2407 (----*-----)

8 -0.4044 0.2974 0.9992 (----*-----)

9 -0.3325 0.3693 1.0710 (-----*-----)

10 -0.4214 0.2804 0.9822 (-----*-----)

-------+---------+---------+---------

-1.2 0.0 1.2

Time Point = 6 subtracted from:

Time

Point Lower Center Upper -------+---------+---------+---------

7 -0.3855 0.31630 1.0181 (-----*----)

8 -0.6270 0.07481 0.7766 (-----*----)

9 -0.5551 0.14667 0.8484 (-----*-----)

10 -0.6440 0.05778 0.7596 (----*-----)

-------+---------+---------+---------

-1.2 0.0 1.2

Time Point = 7 subtracted from:

Time

Point Lower Center Upper -------+---------+---------+---------

8 -0.9433 -0.2415 0.4603 (-----*-----)

9 -0.8714 -0.1696 0.5322 (-----*----)

10 -0.9603 -0.2585 0.4433 (-----*-----)

-------+---------+---------+---------

-1.2 0.0 1.2

Time Point = 8 subtracted from:

Time

Point Lower Center Upper -------+---------+---------+---------

9 -0.6299 0.07185 0.7736 (-----*----)

10 -0.7188 -0.01704 0.6847 (-----*-----)

-------+---------+---------+---------

-1.2 0.0 1.2

Time Point = 9 subtracted from:

Time

Point Lower Center Upper -------+---------+---------+---------

10 -0.7907 -0.08889 0.6129 (-----*-----)

-------+---------+---------+---------

-1.2 0.0 1.2

Tukey Simultaneous Tests

Response Variable Cq Prime

All Pairwise Comparisons among Levels of Time Point

Time Point = 3 subtracted from:

Time Difference SE of Adjusted

Point of Means Difference T-Value P-Value

4 -1.265 0.2228 -5.679 0.0014

5 -0.558 0.2228 -2.506 0.2728

6 -0.336 0.2228 -1.507 0.7917

7 -0.019 0.2228 -0.087 1.0000

8 -0.261 0.2228 -1.171 0.9269

9 -0.189 0.2228 -0.849 0.9861

10 -0.278 0.2228 -1.248 0.9026

Time Point = 4 subtracted from:

Time Difference SE of Adjusted

Point of Means Difference T-Value P-Value

5 0.7070 0.1965 3.598 0.0477

6 0.9296 0.1965 4.730 0.0068

7 1.2459 0.1965 6.340 0.0005

8 1.0044 0.1965 5.111 0.0036

9 1.0763 0.1965 5.477 0.0020

10 0.9874 0.1965 5.024 0.0042

Time Point = 5 subtracted from:

Time Difference SE of Adjusted

Point of Means Difference T-Value P-Value

6 0.2226 0.1965 1.133 0.9375

7 0.5389 0.1965 2.742 0.1929

8 0.2974 0.1965 1.513 0.7885

9 0.3693 0.1965 1.879 0.5851

10 0.2804 0.1965 1.427 0.8303

Time Point = 6 subtracted from:

Time Difference SE of Adjusted

Point of Means Difference T-Value P-Value

7 0.31630 0.1965 1.6094 0.7381

8 0.07481 0.1965 0.3807 0.9999

9 0.14667 0.1965 0.7463 0.9934

10 0.05778 0.1965 0.2940 1.0000

Time Point = 7 subtracted from:

Time Difference SE of Adjusted

Point of Means Difference T-Value P-Value

8 -0.2415 0.1965 -1.229 0.9090

9 -0.1696 0.1965 -0.863 0.9847

10 -0.2585 0.1965 -1.315 0.8777

Time Point = 8 subtracted from:

Time Difference SE of Adjusted

Point of Means Difference T-Value P-Value

9 0.07185 0.1965 0.36560 0.9999

10 -0.01704 0.1965 -0.08669 1.0000

Time Point = 9 subtracted from:

Time Difference SE of Adjusted

Point of Means Difference T-Value P-Value

10 -0.08889 0.1965 -0.4523 0.9997

**Residual Plots for Cq Prime**

**Main Effects Plot for Cq Prime**

PSN0011:

**General Linear Model: Cq Prime versus Biounit, Time Point**

Factor Type Levels Values

Biounit random 3 1, 2, 3

Time Point fixed 8 3, 4, 5, 6, 7, 8, 9, 10

Analysis of Variance for Cq Prime, using Adjusted SS for Tests

Source DF Seq SS Adj SS Adj MS F P

Biounit 2 7.5103 16.2975 8.1488 15.26 0.000

Time Point 7 64.6191 64.6191 9.2313 17.29 0.000

Error 13 6.9409 6.9409 0.5339

Total 22 79.0703

S = 0.730695 R-Sq = 91.22% R-Sq(adj) = 85.14%

Term Coef SE Coef T P

Constant 4.1608 0.1544 26.95 0.000

Biounit

1 -1.1765 0.2255 -5.22 0.000

2 0.2562 0.2147 1.19 0.254

Time Point

3 -4.7491 0.4833 -9.83 0.000

4 -1.1688 0.3966 -2.95 0.011

5 0.5893 0.3966 1.49 0.161

6 0.8897 0.3966 2.24 0.043

7 0.8671 0.3966 2.19 0.048

8 1.3579 0.3966 3.42 0.005

9 1.2219 0.3966 3.08 0.009

Unusual Observations for Cq Prime

Obs Cq Prime Fit SE Fit Residual St Resid

1 -1.55500 -0.33201 0.54802 -1.22299 -2.53 R

2 1.55500 0.33201 0.54802 1.22299 2.53 R

R denotes an observation with a large standardized residual.

Expected Mean Squares, using Adjusted SS

Expected Mean Square

Source for Each Term

1 Biounit (3) + 7.5000 (1)

2 Time Point (3) + Q[2]

3 Error (3)

Error Terms for Tests, using Adjusted SS

Synthesis

Source Error DF Error MS of Error MS

1 Biounit 13.00 0.5339 (3)

2 Time Point 13.00 0.5339 (3)

Variance Components, using Adjusted SS

Estimated

Source Value

Biounit 1.0153

Error 0.5339

Grouping Information Using Bonferroni Method and 95.0% Confidence

Time

Point N Mean Grouping

8 3 5.5187 A

9 3 5.3828 A

10 3 5.1528 A B

6 3 5.0506 A B

7 3 5.0280 A B

5 3 4.7502 A B

4 3 2.9920 B

3 2 -0.5883 C

Means that do not share a letter are significantly different.

Bonferroni 95.0% Simultaneous Confidence Intervals

Response Variable Cq Prime

All Pairwise Comparisons among Levels of Time Point

Time Point = 3 subtracted from:

Time

Point Lower Center Upper --------+---------+---------+--------

4 0.9340 3.580 6.227 (------*-------)

5 2.6921 5.338 7.985 (------*-------)

6 2.9925 5.639 8.285 (------*-------)

7 2.9699 5.616 8.263 (-------*-------)

8 3.4606 6.107 8.753 (------*-------)

9 3.3247 5.971 8.617 (-------*-------)

10 3.0947 5.741 8.387 (------*-------)

--------+---------+---------+--------

0.0 3.5 7.0

Time Point = 4 subtracted from:

Time

Point Lower Center Upper --------+---------+---------+--------

5 -0.5757 1.758 4.092 (------*------)

6 -0.2753 2.059 4.392 (------*------)

7 -0.2979 2.036 4.370 (------*-----)

8 0.1928 2.527 4.861 (-----*------)

9 0.0569 2.391 4.725 (------*-----)

10 -0.1731 2.161 4.495 (-----*------)

--------+---------+---------+--------

0.0 3.5 7.0

Time Point = 5 subtracted from:

Time

Point Lower Center Upper --------+---------+---------+--------

6 -2.033 0.3004 2.634 (------*------)

7 -2.056 0.2778 2.612 (------*-----)

8 -1.565 0.7685 3.102 (-----*------)

9 -1.701 0.6326 2.966 (------*-----)

10 -1.931 0.4026 2.736 (------*------)

--------+---------+---------+--------

0.0 3.5 7.0

Time Point = 6 subtracted from:

Time

Point Lower Center Upper --------+---------+---------+--------

7 -2.356 -0.02259 2.311 (------*------)

8 -1.866 0.46815 2.802 (-----*------)

9 -2.002 0.33222 2.666 (------*------)

10 -2.232 0.10222 2.436 (-----*------)

--------+---------+---------+--------

0.0 3.5 7.0

Time Point = 7 subtracted from:

Time

Point Lower Center Upper --------+---------+---------+--------

8 -1.843 0.4907 2.825 (-----*------)

9 -1.979 0.3548 2.689 (------*------)

10 -2.209 0.1248 2.459 (-----*------)

--------+---------+---------+--------

0.0 3.5 7.0

Time Point = 8 subtracted from:

Time

Point Lower Center Upper --------+---------+---------+--------

9 -2.470 -0.1359 2.198 (------*-----)

10 -2.700 -0.3659 1.968 (------*------)

--------+---------+---------+--------

0.0 3.5 7.0

Time Point = 9 subtracted from:

Time

Point Lower Center Upper --------+---------+---------+--------

10 -2.564 -0.2300 2.104 (-----*------)

--------+---------+---------+--------

0.0 3.5 7.0

Bonferroni Simultaneous Tests

Response Variable Cq Prime

All Pairwise Comparisons among Levels of Time Point

Time Point = 3 subtracted from:

Time Difference SE of Adjusted

Point of Means Difference T-Value P-Value

4 3.580 0.6765 5.292 0.0041

5 5.338 0.6765 7.891 0.0001

6 5.639 0.6765 8.335 0.0000

7 5.616 0.6765 8.302 0.0000

8 6.107 0.6765 9.027 0.0000

9 5.971 0.6765 8.826 0.0000

10 5.741 0.6765 8.486 0.0000

Time Point = 4 subtracted from:

Time Difference SE of Adjusted

Point of Means Difference T-Value P-Value

5 1.758 0.5966 2.947 0.3175

6 2.059 0.5966 3.450 0.1205

7 2.036 0.5966 3.412 0.1296

8 2.527 0.5966 4.235 0.0273

9 2.391 0.5966 4.007 0.0418

10 2.161 0.5966 3.622 0.0868

Time Point = 5 subtracted from:

Time Difference SE of Adjusted

Point of Means Difference T-Value P-Value

6 0.3004 0.5966 0.5035 1.000

7 0.2778 0.5966 0.4656 1.000

8 0.7685 0.5966 1.2881 1.000

9 0.6326 0.5966 1.0603 1.000

10 0.4026 0.5966 0.6748 1.000

Time Point = 6 subtracted from:

Time Difference SE of Adjusted

Point of Means Difference T-Value P-Value

7 -0.02259 0.5966 -0.03787 1.000

8 0.46815 0.5966 0.78468 1.000

9 0.33222 0.5966 0.55685 1.000

10 0.10222 0.5966 0.17134 1.000

Time Point = 7 subtracted from:

Time Difference SE of Adjusted

Point of Means Difference T-Value P-Value

8 0.4907 0.5966 0.8225 1.000

9 0.3548 0.5966 0.5947 1.000

10 0.1248 0.5966 0.2092 1.000

Time Point = 8 subtracted from:

Time Difference SE of Adjusted

Point of Means Difference T-Value P-Value

9 -0.1359 0.5966 -0.2278 1.000

10 -0.3659 0.5966 -0.6133 1.000

Time Point = 9 subtracted from:

Time Difference SE of Adjusted

Point of Means Difference T-Value P-Value

10 -0.2300 0.5966 -0.3855 1.000

Grouping Information Using Sidak Method and 95.0% Confidence

Time

Point N Mean Grouping

8 3 5.5187 A

9 3 5.3828 A

10 3 5.1528 A B

6 3 5.0506 A B

7 3 5.0280 A B

5 3 4.7502 A B

4 3 2.9920 B

3 2 -0.5883 C

Means that do not share a letter are significantly different.

Sidak 95.0% Simultaneous Confidence Intervals

Response Variable Cq Prime

All Pairwise Comparisons among Levels of Time Point

Time Point = 3 subtracted from:

Time

Point Lower Center Upper --------+---------+---------+--------

4 0.9428 3.580 6.218 (------*-------)

5 2.7009 5.338 7.976 (------*-------)

6 3.0013 5.639 8.276 (------*-------)

7 2.9787 5.616 8.254 (------*-------)

8 3.4694 6.107 8.744 (------*-------)

9 3.3335 5.971 8.609 (------*-------)

10 3.1035 5.741 8.379 (------*-------)

--------+---------+---------+--------

0.0 3.5 7.0

Time Point = 4 subtracted from:

Time

Point Lower Center Upper --------+---------+---------+--------

5 -0.5679 1.758 4.084 (------*------)

6 -0.2676 2.059 4.385 (------*------)

7 -0.2902 2.036 4.362 (------*-----)

8 0.2006 2.527 4.853 (-----*------)

9 0.0647 2.391 4.717 (------*-----)

10 -0.1653 2.161 4.487 (-----*------)

--------+---------+---------+--------

0.0 3.5 7.0

Time Point = 5 subtracted from:

Time

Point Lower Center Upper --------+---------+---------+--------

6 -2.026 0.3004 2.626 (------*------)

7 -2.048 0.2778 2.604 (------*-----)

8 -1.558 0.7685 3.095 (-----*------)

9 -1.693 0.6326 2.959 (------*-----)

10 -1.923 0.4026 2.729 (-----*------)

--------+---------+---------+--------

0.0 3.5 7.0

Time Point = 6 subtracted from:

Time

Point Lower Center Upper --------+---------+---------+--------

7 -2.349 -0.02259 2.303 (------*------)

8 -1.858 0.46815 2.794 (-----*------)

9 -1.994 0.33222 2.658 (------*------)

10 -2.224 0.10222 2.428 (-----*------)

--------+---------+---------+--------

0.0 3.5 7.0

Time Point = 7 subtracted from:

Time

Point Lower Center Upper --------+---------+---------+--------

8 -1.835 0.4907 2.817 (-----*------)

9 -1.971 0.3548 2.681 (------*------)

10 -2.201 0.1248 2.451 (-----*------)

--------+---------+---------+--------

0.0 3.5 7.0

Time Point = 8 subtracted from:

Time

Point Lower Center Upper --------+---------+---------+--------

9 -2.462 -0.1359 2.190 (------*-----)

10 -2.692 -0.3659 1.960 (------*------)

--------+---------+---------+--------

0.0 3.5 7.0

Time Point = 9 subtracted from:

Time

Point Lower Center Upper --------+---------+---------+--------

10 -2.556 -0.2300 2.096 (-----*------)

--------+---------+---------+--------

0.0 3.5 7.0

Sidak Simultaneous Tests

Response Variable Cq Prime

All Pairwise Comparisons among Levels of Time Point

Time Point = 3 subtracted from:

Time Difference SE of Adjusted

Point of Means Difference T-Value P-Value

4 3.580 0.6765 5.292 0.0041

5 5.338 0.6765 7.891 0.0001

6 5.639 0.6765 8.335 0.0000

7 5.616 0.6765 8.302 0.0000

8 6.107 0.6765 9.027 0.0000

9 5.971 0.6765 8.826 0.0000

10 5.741 0.6765 8.486 0.0000

Time Point = 4 subtracted from:

Time Difference SE of Adjusted

Point of Means Difference T-Value P-Value

5 1.758 0.5966 2.947 0.2734

6 2.059 0.5966 3.450 0.1138

7 2.036 0.5966 3.412 0.1218

8 2.527 0.5966 4.235 0.0269

9 2.391 0.5966 4.007 0.0409

10 2.161 0.5966 3.622 0.0833

Time Point = 5 subtracted from:

Time Difference SE of Adjusted

Point of Means Difference T-Value P-Value

6 0.3004 0.5966 0.5035 1.0000

7 0.2778 0.5966 0.4656 1.0000

8 0.7685 0.5966 1.2881 0.9991

9 0.6326 0.5966 1.0603 1.0000

10 0.4026 0.5966 0.6748 1.0000

Time Point = 6 subtracted from:

Time Difference SE of Adjusted

Point of Means Difference T-Value P-Value

7 -0.02259 0.5966 -0.03787 1.000

8 0.46815 0.5966 0.78468 1.000

9 0.33222 0.5966 0.55685 1.000

10 0.10222 0.5966 0.17134 1.000

Time Point = 7 subtracted from:

Time Difference SE of Adjusted

Point of Means Difference T-Value P-Value

8 0.4907 0.5966 0.8225 1.000

9 0.3548 0.5966 0.5947 1.000

10 0.1248 0.5966 0.2092 1.000

Time Point = 8 subtracted from:

Time Difference SE of Adjusted

Point of Means Difference T-Value P-Value

9 -0.1359 0.5966 -0.2278 1.000

10 -0.3659 0.5966 -0.6133 1.000

Time Point = 9 subtracted from:

Time Difference SE of Adjusted

Point of Means Difference T-Value P-Value

10 -0.2300 0.5966 -0.3855 1.000

Grouping Information Using Tukey Method and 95.0% Confidence

Time

Point N Mean Grouping

8 3 5.5187 A

9 3 5.3828 A

10 3 5.1528 A

6 3 5.0506 A B

7 3 5.0280 A B

5 3 4.7502 A B

4 3 2.9920 B

3 2 -0.5883 C

Means that do not share a letter are significantly different.

Tukey 95.0% Simultaneous Confidence Intervals

Response Variable Cq Prime

All Pairwise Comparisons among Levels of Time Point

Time Point = 3 subtracted from:

Time

Point Lower Center Upper --------+---------+---------+--------

4 1.165 3.580 5.996 (-------*-------)

5 2.923 5.338 7.754 (-------*-------)

6 3.223 5.639 8.054 (-------*-------)

7 3.201 5.616 8.032 (-------*-------)

8 3.691 6.107 8.523 (-------*-------)

9 3.555 5.971 8.387 (-------*-------)

10 3.325 5.741 8.157 (-------*-------)

--------+---------+---------+--------

0.0 3.0 6.0

Time Point = 4 subtracted from:

Time

Point Lower Center Upper --------+---------+---------+--------

5 -0.3723 1.758 3.889 (------*------)

6 -0.0719 2.059 4.189 (------*------)

7 -0.0945 2.036 4.166 (------*------)

8 0.3962 2.527 4.657 (------*-------)

9 0.2603 2.391 4.521 (------*------)

10 0.0303 2.161 4.291 (------*------)

--------+---------+---------+--------

0.0 3.0 6.0

Time Point = 5 subtracted from:

Time

Point Lower Center Upper --------+---------+---------+--------

6 -1.830 0.3004 2.431 (------*------)

7 -1.853 0.2778 2.408 (------*------)

8 -1.362 0.7685 2.899 (-------*------)

9 -1.498 0.6326 2.763 (------*------)

10 -1.728 0.4026 2.533 (------*------)

--------+---------+---------+--------

0.0 3.0 6.0

Time Point = 6 subtracted from:

Time

Point Lower Center Upper --------+---------+---------+--------

7 -2.153 -0.02259 2.108 (------*------)

8 -1.662 0.46815 2.599 (-------*------)

9 -1.798 0.33222 2.463 (------*------)

10 -2.028 0.10222 2.233 (------*------)

--------+---------+---------+--------

0.0 3.0 6.0

Time Point = 7 subtracted from:

Time

Point Lower Center Upper --------+---------+---------+--------

8 -1.640 0.4907 2.621 (------*------)

9 -1.776 0.3548 2.485 (------*------)

10 -2.006 0.1248 2.255 (------*-------)

--------+---------+---------+--------

0.0 3.0 6.0

Time Point = 8 subtracted from:

Time

Point Lower Center Upper --------+---------+---------+--------

9 -2.266 -0.1359 1.995 (-------*------)

10 -2.496 -0.3659 1.765 (------*------)

--------+---------+---------+--------

0.0 3.0 6.0

Time Point = 9 subtracted from:

Time

Point Lower Center Upper --------+---------+---------+--------

10 -2.360 -0.2300 1.900 (------*------)

--------+---------+---------+--------

0.0 3.0 6.0

Tukey Simultaneous Tests

Response Variable Cq Prime

All Pairwise Comparisons among Levels of Time Point

Time Point = 3 subtracted from:

Time Difference SE of Adjusted

Point of Means Difference T-Value P-Value

4 3.580 0.6765 5.292 0.0027

5 5.338 0.6765 7.891 0.0001

6 5.639 0.6765 8.335 0.0000

7 5.616 0.6765 8.302 0.0000

8 6.107 0.6765 9.027 0.0000

9 5.971 0.6765 8.826 0.0000

10 5.741 0.6765 8.486 0.0000

Time Point = 4 subtracted from:

Time Difference SE of Adjusted

Point of Means Difference T-Value P-Value

5 1.758 0.5966 2.947 0.1403

6 2.059 0.5966 3.450 0.0613

7 2.036 0.5966 3.412 0.0653

8 2.527 0.5966 4.235 0.0159

9 2.391 0.5966 4.007 0.0236

10 2.161 0.5966 3.622 0.0458

Time Point = 5 subtracted from:

Time Difference SE of Adjusted

Point of Means Difference T-Value P-Value

6 0.3004 0.5966 0.5035 0.9994

7 0.2778 0.5966 0.4656 0.9997

8 0.7685 0.5966 1.2881 0.8881

9 0.6326 0.5966 1.0603 0.9547

10 0.4026 0.5966 0.6748 0.9964

Time Point = 6 subtracted from:

Time Difference SE of Adjusted

Point of Means Difference T-Value P-Value

7 -0.02259 0.5966 -0.03787 1.0000

8 0.46815 0.5966 0.78468 0.9911

9 0.33222 0.5966 0.55685 0.9989

10 0.10222 0.5966 0.17134 1.0000

Time Point = 7 subtracted from:

Time Difference SE of Adjusted

Point of Means Difference T-Value P-Value

8 0.4907 0.5966 0.8225 0.9884

9 0.3548 0.5966 0.5947 0.9983

10 0.1248 0.5966 0.2092 1.0000

Time Point = 8 subtracted from:

Time Difference SE of Adjusted

Point of Means Difference T-Value P-Value

9 -0.1359 0.5966 -0.2278 1.0000

10 -0.3659 0.5966 -0.6133 0.9980

Time Point = 9 subtracted from:

Time Difference SE of Adjusted

Point of Means Difference T-Value P-Value

10 -0.2300 0.5966 -0.3855 0.9999

**Residual Plots for Cq Prime**

**Main Effects Plot for Cq Prime**

PSN0015:

**General Linear Model: Cq Prime versus Biounit, Time Point**

Factor Type Levels Values

Biounit random 3 1, 2, 3

Time Point fixed 8 3, 4, 5, 6, 7, 8, 9, 10

Analysis of Variance for Cq Prime, using Adjusted SS for Tests

Source DF Seq SS Adj SS Adj MS F P

Biounit 2 18.6811 8.6655 4.3328 5.90 0.015

Time Point 7 64.6800 64.6800 9.2400 12.57 0.000

Error 13 9.5528 9.5528 0.7348

Total 22 92.9140

S = 0.857224 R-Sq = 89.72% R-Sq(adj) = 82.60%

Term Coef SE Coef T P

Constant 4.9044 0.1811 27.08 0.000

Biounit

1 0.8211 0.2645 3.10 0.008

2 -0.0957 0.2518 -0.38 0.710

Time Point

3 -4.4939 0.5670 -7.93 0.000

4 -1.4313 0.4653 -3.08 0.009

5 0.2446 0.4653 0.53 0.608

6 0.5383 0.4653 1.16 0.268

7 1.0935 0.4653 2.35 0.035

8 1.3365 0.4653 2.87 0.013

9 1.3594 0.4653 2.92 0.012

Unusual Observations for Cq Prime

Obs Cq Prime Fit SE Fit Residual St Resid

5 1.36389 2.74773 0.55531 -1.38384 -2.12 R

R denotes an observation with a large standardized residual.

Expected Mean Squares, using Adjusted SS

Expected Mean Square

Source for Each Term

1 Biounit (3) + 7.5000 (1)

2 Time Point (3) + Q[2]

3 Error (3)

Error Terms for Tests, using Adjusted SS

Synthesis

Source Error DF Error MS of Error MS

1 Biounit 13.00 0.7348 (3)

2 Time Point 13.00 0.7348 (3)

Variance Components, using Adjusted SS

Estimated

Source Value

Biounit 0.4797

Error 0.7348

Grouping Information Using Bonferroni Method and 95.0% Confidence

Time

Point N Mean Grouping

9 3 6.2639 A

10 3 6.2572 A

8 3 6.2409 A

7 3 5.9980 A B

6 3 5.4428 A B

5 3 5.1491 A B

4 3 3.4731 B C

3 2 0.4106 C

Means that do not share a letter are significantly different.

Bonferroni 95.0% Simultaneous Confidence Intervals

Response Variable Cq Prime

All Pairwise Comparisons among Levels of Time Point

Time Point = 3 subtracted from:

Time

Point Lower Center Upper --------+---------+---------+--------

4 -0.04199 3.063 6.167 (--------*--------)

5 1.63394 4.739 7.843 (--------*-------)

6 1.92764 5.032 8.137 (-------*--------)

7 2.48283 5.587 8.692 (--------*--------)

8 2.72579 5.830 8.935 (--------*--------)

9 2.74876 5.853 8.958 (--------*--------)

10 2.74209 5.847 8.951 (--------*--------)

--------+---------+---------+--------

0.0 3.5 7.0

Time Point = 4 subtracted from:

Time

Point Lower Center Upper --------+---------+---------+--------

5 -1.062 1.676 4.414 (-------*-------)

6 -0.768 1.970 4.708 (-------*------)

7 -0.213 2.525 5.263 (-------*-------)

8 0.030 2.768 5.506 (-------*-------)

9 0.053 2.791 5.529 (-------*-------)

10 0.046 2.784 5.522 (-------*-------)

--------+---------+---------+--------

0.0 3.5 7.0

Time Point = 5 subtracted from:

Time

Point Lower Center Upper --------+---------+---------+--------

6 -2.444 0.2937 3.032 (-------*-------)

7 -1.889 0.8489 3.587 (------*-------)

8 -1.646 1.0919 3.830 (-------*-------)

9 -1.623 1.1148 3.853 (-------*-------)

10 -1.630 1.1081 3.846 (-------*-------)

--------+---------+---------+--------

0.0 3.5 7.0

Time Point = 6 subtracted from:

Time

Point Lower Center Upper --------+---------+---------+--------

7 -2.183 0.5552 3.293 (-------*------)

8 -1.940 0.7981 3.536 (-------*-------)

9 -1.917 0.8211 3.559 (------*-------)

10 -1.924 0.8144 3.552 (------*-------)

--------+---------+---------+--------

0.0 3.5 7.0

Time Point = 7 subtracted from:

Time

Point Lower Center Upper --------+---------+---------+--------

8 -2.495 0.2430 2.981 (-------*-------)

9 -2.472 0.2659 3.004 (-------*-------)

10 -2.479 0.2593 2.997 (-------*-------)

--------+---------+---------+--------

0.0 3.5 7.0

Time Point = 8 subtracted from:

Time

Point Lower Center Upper --------+---------+---------+--------

9 -2.715 0.02296 2.761 (-------*-------)

10 -2.722 0.01630 2.754 (-------*-------)

--------+---------+---------+--------

0.0 3.5 7.0

Time Point = 9 subtracted from:

Time

Point Lower Center Upper --------+---------+---------+--------

10 -2.745 -0.006667 2.731 (-------*-------)

--------+---------+---------+--------

0.0 3.5 7.0

Bonferroni Simultaneous Tests

Response Variable Cq Prime

All Pairwise Comparisons among Levels of Time Point

Time Point = 3 subtracted from:

Time Difference SE of Adjusted

Point of Means Difference T-Value P-Value

4 3.063 0.7936 3.859 0.0553

5 4.739 0.7936 5.971 0.0013

6 5.032 0.7936 6.341 0.0007

7 5.587 0.7936 7.040 0.0002

8 5.830 0.7936 7.346 0.0002

9 5.853 0.7936 7.375 0.0002

10 5.847 0.7936 7.367 0.0002

Time Point = 4 subtracted from:

Time Difference SE of Adjusted

Point of Means Difference T-Value P-Value

5 1.676 0.6999 2.394 0.9078

6 1.970 0.6999 2.814 0.4096

7 2.525 0.6999 3.607 0.0892

8 2.768 0.6999 3.954 0.0461

9 2.791 0.6999 3.987 0.0434

10 2.784 0.6999 3.978 0.0442

Time Point = 5 subtracted from:

Time Difference SE of Adjusted

Point of Means Difference T-Value P-Value

6 0.2937 0.6999 0.4196 1.000

7 0.8489 0.6999 1.2128 1.000

8 1.0919 0.6999 1.5600 1.000

9 1.1148 0.6999 1.5928 1.000

10 1.1081 0.6999 1.5832 1.000

Time Point = 6 subtracted from:

Time Difference SE of Adjusted

Point of Means Difference T-Value P-Value

7 0.5552 0.6999 0.7932 1.000

8 0.7981 0.6999 1.1403 1.000

9 0.8211 0.6999 1.1731 1.000

10 0.8144 0.6999 1.1636 1.000

Time Point = 7 subtracted from:

Time Difference SE of Adjusted

Point of Means Difference T-Value P-Value

8 0.2430 0.6999 0.3471 1.000

9 0.2659 0.6999 0.3799 1.000

10 0.2593 0.6999 0.3704 1.000

Time Point = 8 subtracted from:

Time Difference SE of Adjusted

Point of Means Difference T-Value P-Value

9 0.02296 0.6999 0.03281 1.000

10 0.01630 0.6999 0.02328 1.000

Time Point = 9 subtracted from:

Time Difference SE of Adjusted

Point of Means Difference T-Value P-Value

10 -0.006667 0.6999 -0.009525 1.000

Grouping Information Using Sidak Method and 95.0% Confidence

Time

Point N Mean Grouping

9 3 6.2639 A

10 3 6.2572 A

8 3 6.2409 A

7 3 5.9980 A B

6 3 5.4428 A B

5 3 5.1491 A B

4 3 3.4731 B C

3 2 0.4106 C

Means that do not share a letter are significantly different.

Sidak 95.0% Simultaneous Confidence Intervals

Response Variable Cq Prime

All Pairwise Comparisons among Levels of Time Point

Time Point = 3 subtracted from:

Time

Point Lower Center Upper --------+---------+---------+--------

4 -0.03165 3.063 6.157 (--------*--------)

5 1.64427 4.739 7.833 (--------*-------)

6 1.93798 5.032 8.126 (-------*--------)

7 2.49316 5.587 8.682 (--------*--------)

8 2.73612 5.830 8.925 (--------*-------)

9 2.75909 5.853 8.948 (--------*--------)

10 2.75242 5.847 8.941 (--------*--------)

--------+---------+---------+--------

0.0 3.5 7.0

Time Point = 4 subtracted from:

Time

Point Lower Center Upper --------+---------+---------+--------

5 -1.053 1.676 4.405 (-------*-------)

6 -0.759 1.970 4.698 (-------*------)

7 -0.204 2.525 5.254 (-------*-------)

8 0.039 2.768 5.497 (-------*-------)

9 0.062 2.791 5.520 (-------*-------)

10 0.055 2.784 5.513 (-------*-------)

--------+---------+---------+--------

0.0 3.5 7.0

Time Point = 5 subtracted from:

Time

Point Lower Center Upper --------+---------+---------+--------

6 -2.435 0.2937 3.023 (-------*-------)

7 -1.880 0.8489 3.578 (------*-------)

8 -1.637 1.0919 3.821 (-------*-------)

9 -1.614 1.1148 3.844 (-------*-------)

10 -1.621 1.1081 3.837 (-------*-------)

--------+---------+---------+--------

0.0 3.5 7.0

Time Point = 6 subtracted from:

Time

Point Lower Center Upper --------+---------+---------+--------

7 -2.174 0.5552 3.284 (-------*------)

8 -1.931 0.7981 3.527 (-------*-------)

9 -1.908 0.8211 3.550 (------*-------)

10 -1.914 0.8144 3.543 (------*-------)

--------+---------+---------+--------

0.0 3.5 7.0

Time Point = 7 subtracted from:

Time

Point Lower Center Upper --------+---------+---------+--------

8 -2.486 0.2430 2.972 (-------*------)

9 -2.463 0.2659 2.995 (-------*-------)

10 -2.470 0.2593 2.988 (-------*-------)

--------+---------+---------+--------

0.0 3.5 7.0

Time Point = 8 subtracted from:

Time

Point Lower Center Upper --------+---------+---------+--------

9 -2.706 0.02296 2.752 (-------*-------)

10 -2.713 0.01630 2.745 (-------*-------)

--------+---------+---------+--------

0.0 3.5 7.0

Time Point = 9 subtracted from:

Time

Point Lower Center Upper --------+---------+---------+--------

10 -2.736 -0.006667 2.722 (-------*-------)

--------+---------+---------+--------

0.0 3.5 7.0

Sidak Simultaneous Tests

Response Variable Cq Prime

All Pairwise Comparisons among Levels of Time Point

Time Point = 3 subtracted from:

Time Difference SE of Adjusted

Point of Means Difference T-Value P-Value

4 3.063 0.7936 3.859 0.0538

5 4.739 0.7936 5.971 0.0013

6 5.032 0.7936 6.341 0.0007

7 5.587 0.7936 7.040 0.0002

8 5.830 0.7936 7.346 0.0002

9 5.853 0.7936 7.375 0.0002

10 5.847 0.7936 7.367 0.0002

Time Point = 4 subtracted from:

Time Difference SE of Adjusted

Point of Means Difference T-Value P-Value

5 1.676 0.6999 2.394 0.6026

6 1.970 0.6999 2.814 0.3381

7 2.525 0.6999 3.607 0.0855

8 2.768 0.6999 3.954 0.0451

9 2.791 0.6999 3.987 0.0425

10 2.784 0.6999 3.978 0.0432

Time Point = 5 subtracted from:

Time Difference SE of Adjusted

Point of Means Difference T-Value P-Value

6 0.2937 0.6999 0.4196 1.0000

7 0.8489 0.6999 1.2128 0.9996

8 1.0919 0.6999 1.5600 0.9866

9 1.1148 0.6999 1.5928 0.9829

10 1.1081 0.6999 1.5832 0.9840

Time Point = 6 subtracted from:

Time Difference SE of Adjusted

Point of Means Difference T-Value P-Value

7 0.5552 0.6999 0.7932 1.0000

8 0.7981 0.6999 1.1403 0.9999

9 0.8211 0.6999 1.1731 0.9998

10 0.8144 0.6999 1.1636 0.9998

Time Point = 7 subtracted from:

Time Difference SE of Adjusted

Point of Means Difference T-Value P-Value

8 0.2430 0.6999 0.3471 1.000

9 0.2659 0.6999 0.3799 1.000

10 0.2593 0.6999 0.3704 1.000

Time Point = 8 subtracted from:

Time Difference SE of Adjusted

Point of Means Difference T-Value P-Value

9 0.02296 0.6999 0.03281 1.000

10 0.01630 0.6999 0.02328 1.000

Time Point = 9 subtracted from:

Time Difference SE of Adjusted

Point of Means Difference T-Value P-Value

10 -0.006667 0.6999 -0.009525 1.000

Grouping Information Using Tukey Method and 95.0% Confidence

Time

Point N Mean Grouping

9 3 6.2639 A

10 3 6.2572 A

8 3 6.2409 A

7 3 5.9980 A

6 3 5.4428 A B

5 3 5.1491 A B

4 3 3.4731 B

3 2 0.4106 C

Means that do not share a letter are significantly different.

Tukey 95.0% Simultaneous Confidence Intervals

Response Variable Cq Prime

All Pairwise Comparisons among Levels of Time Point

Time Point = 3 subtracted from:

Time

Point Lower Center Upper -------+---------+---------+---------

4 0.2286 3.063 5.897 (-------*-------)

5 1.9045 4.739 7.573 (--------*-------)

6 2.1982 5.032 7.866 (-------*-------)

7 2.7534 5.587 8.421 (-------*-------)

8 2.9964 5.830 8.664 (-------*-------)

9 3.0194 5.853 8.687 (-------*-------)

10 3.0127 5.847 8.681 (-------*-------)

-------+---------+---------+---------

0.0 3.5 7.0

Time Point = 4 subtracted from:

Time

Point Lower Center Upper -------+---------+---------+---------

5 -0.8234 1.676 4.175 (------*------)

6 -0.5297 1.970 4.469 (-------*------)

7 0.0255 2.525 5.024 (------*------)

8 0.2684 2.768 5.267 (------*------)

9 0.2914 2.791 5.290 (------*------)

10 0.2847 2.784 5.283 (------*------)

-------+---------+---------+---------

0.0 3.5 7.0

Time Point = 5 subtracted from:

Time

Point Lower Center Upper -------+---------+---------+---------

6 -2.206 0.2937 2.793 (------*------)

7 -1.650 0.8489 3.348 (------*-------)

8 -1.407 1.0919 3.591 (------*------)

9 -1.385 1.1148 3.614 (------*------)

10 -1.391 1.1081 3.607 (------*------)

-------+---------+---------+---------

0.0 3.5 7.0

Time Point = 6 subtracted from:

Time

Point Lower Center Upper -------+---------+---------+---------

7 -1.944 0.5552 3.055 (-------*------)

8 -1.701 0.7981 3.297 (------*------)

9 -1.678 0.8211 3.320 (------*------)

10 -1.685 0.8144 3.314 (------*------)

-------+---------+---------+---------

0.0 3.5 7.0

Time Point = 7 subtracted from:

Time

Point Lower Center Upper -------+---------+---------+---------

8 -2.256 0.2430 2.742 (------*------)

9 -2.233 0.2659 2.765 (------*------)

10 -2.240 0.2593 2.759 (------*------)

-------+---------+---------+---------

0.0 3.5 7.0

Time Point = 8 subtracted from:

Time

Point Lower Center Upper -------+---------+---------+---------

9 -2.476 0.02296 2.522 (------*------)

10 -2.483 0.01630 2.516 (------*------)

-------+---------+---------+---------

0.0 3.5 7.0

Time Point = 9 subtracted from:

Time

Point Lower Center Upper -------+---------+---------+---------

10 -2.506 -0.006667 2.493 (------*------)

-------+---------+---------+---------

0.0 3.5 7.0

Tukey Simultaneous Tests

Response Variable Cq Prime

All Pairwise Comparisons among Levels of Time Point

Time Point = 3 subtracted from:

Time Difference SE of Adjusted

Point of Means Difference T-Value P-Value

4 3.063 0.7936 3.859 0.0305

5 4.739 0.7936 5.971 0.0009

6 5.032 0.7936 6.341 0.0005

7 5.587 0.7936 7.040 0.0002

8 5.830 0.7936 7.346 0.0001

9 5.853 0.7936 7.375 0.0001

10 5.847 0.7936 7.367 0.0001

Time Point = 4 subtracted from:

Time Difference SE of Adjusted

Point of Means Difference T-Value P-Value

5 1.676 0.6999 2.394 0.3182

6 1.970 0.6999 2.814 0.1727

7 2.525 0.6999 3.607 0.0470

8 2.768 0.6999 3.954 0.0259

9 2.791 0.6999 3.987 0.0244

10 2.784 0.6999 3.978 0.0249

Time Point = 5 subtracted from:

Time Difference SE of Adjusted

Point of Means Difference T-Value P-Value

6 0.2937 0.6999 0.4196 0.9998

7 0.8489 0.6999 1.2128 0.9142

8 1.0919 0.6999 1.5600 0.7644

9 1.1148 0.6999 1.5928 0.7470

10 1.1081 0.6999 1.5832 0.7521

Time Point = 6 subtracted from:

Time Difference SE of Adjusted

Point of Means Difference T-Value P-Value

7 0.5552 0.6999 0.7932 0.9905

8 0.7981 0.6999 1.1403 0.9355

9 0.8211 0.6999 1.1731 0.9263

10 0.8144 0.6999 1.1636 0.9290

Time Point = 7 subtracted from:

Time Difference SE of Adjusted

Point of Means Difference T-Value P-Value

8 0.2430 0.6999 0.3471 1.0000

9 0.2659 0.6999 0.3799 0.9999

10 0.2593 0.6999 0.3704 0.9999

Time Point = 8 subtracted from:

Time Difference SE of Adjusted

Point of Means Difference T-Value P-Value

9 0.02296 0.6999 0.03281 1.000

10 0.01630 0.6999 0.02328 1.000

Time Point = 9 subtracted from:

Time Difference SE of Adjusted

Point of Means Difference T-Value P-Value

10 -0.006667 0.6999 -0.009525 1.000

**Residual Plots for Cq Prime**

**Main Effects Plot for Cq Prime**

PSN0016:

**General Linear Model: Cq Prime versus Biounit, Time Point**

Factor Type Levels Values

Biounit random 3 1, 2, 3

Time Point fixed 8 3, 4, 5, 6, 7, 8, 9, 10

Analysis of Variance for Cq Prime, using Adjusted SS for Tests

Source DF Seq SS Adj SS Adj MS F P

Biounit 2 0.9624 4.4660 2.2330 7.37 0.007

Time Point 7 63.7946 63.7946 9.1135 30.07 0.000

Error 13 3.9401 3.9401 0.3031

Total 22 68.6970

S = 0.550530 R-Sq = 94.26% R-Sq(adj) = 90.29%

Term Coef SE Coef T P

Constant 3.4288 0.1163 29.48 0.000

Biounit

1 -0.6506 0.1699 -3.83 0.002

2 0.3616 0.1617 2.24 0.044

Time Point

3 -3.7541 0.3641 -10.31 0.000

4 -2.2795 0.2988 -7.63 0.000

5 0.3920 0.2988 1.31 0.212

6 0.9723 0.2988 3.25 0.006

7 1.0520 0.2988 3.52 0.004

8 1.7771 0.2988 5.95 0.000

9 1.2990 0.2988 4.35 0.001

Unusual Observations for Cq Prime

Obs Cq Prime Fit SE Fit Residual St Resid

7 5.02444 4.18238 0.35663 0.84207 2.01 R

R denotes an observation with a large standardized residual.

Expected Mean Squares, using Adjusted SS

Expected Mean Square

Source for Each Term

1 Biounit (3) + 7.5000 (1)

2 Time Point (3) + Q[2]

3 Error (3)

Error Terms for Tests, using Adjusted SS

Synthesis

Source Error DF Error MS of Error MS

1 Biounit 13.00 0.3031 (3)

2 Time Point 13.00 0.3031 (3)

Variance Components, using Adjusted SS

Estimated

Source Value

Biounit 0.2573

Error 0.3031

Grouping Information Using Bonferroni Method and 95.0% Confidence

Time

Point N Mean Grouping

8 3 5.2059 A

9 3 4.7278 A

7 3 4.4807 A

6 3 4.4011 A

10 3 3.9700 A

5 3 3.8207 A

4 3 1.1493 B

3 2 -0.3253 B

Means that do not share a letter are significantly different.

Bonferroni 95.0% Simultaneous Confidence Intervals

Response Variable Cq Prime

All Pairwise Comparisons among Levels of Time Point

Time Point = 3 subtracted from:

Time

Point Lower Center Upper +---------+---------+---------+------

4 -0.5193 1.475 3.468 (------*------)

5 2.1522 4.146 6.140 (------*-----)

6 2.7326 4.726 6.720 (------*-----)

7 2.8122 4.806 6.800 (------*------)

8 3.5374 5.531 7.525 (-----*------)

9 3.0593 5.053 7.047 (------*-----)

10 2.3015 4.295 6.289 (-----*------)

+---------+---------+---------+------

-3.0 0.0 3.0 6.0

Time Point = 4 subtracted from:

Time

Point Lower Center Upper +---------+---------+---------+------

5 0.9131 2.671 4.430 (-----*-----)

6 1.4935 3.252 5.010 (-----*-----)

7 1.5731 3.331 5.090 (-----*-----)

8 2.2983 4.057 5.815 (-----*----)

9 1.8201 3.579 5.337 (-----*-----)

10 1.0623 2.821 4.579 (----*-----)

+---------+---------+---------+------

-3.0 0.0 3.0 6.0

Time Point = 5 subtracted from:

Time

Point Lower Center Upper +---------+---------+---------+------

6 -1.178 0.5804 2.339 (-----*-----)

7 -1.098 0.6600 2.418 (-----*-----)

8 -0.373 1.3852 3.144 (-----*----)

9 -0.851 0.9070 2.665 (-----*-----)

10 -1.609 0.1493 1.908 (----*-----)

+---------+---------+---------+------

-3.0 0.0 3.0 6.0

Time Point = 6 subtracted from:

Time

Point Lower Center Upper +---------+---------+---------+------

7 -1.679 0.0796 1.838 (-----*-----)

8 -0.954 0.8048 2.563 (-----*-----)

9 -1.432 0.3267 2.085 (-----*-----)

10 -2.190 -0.4311 1.327 (-----*----)

+---------+---------+---------+------

-3.0 0.0 3.0 6.0

Time Point = 7 subtracted from:

Time

Point Lower Center Upper +---------+---------+---------+------

8 -1.033 0.7252 2.484 (----*-----)

9 -1.511 0.2470 2.005 (-----*-----)

10 -2.269 -0.5107 1.248 (-----*-----)

+---------+---------+---------+------

-3.0 0.0 3.0 6.0

Time Point = 8 subtracted from:

Time

Point Lower Center Upper +---------+---------+---------+------

9 -2.237 -0.478 1.2803 (----*-----)

10 -2.994 -1.236 0.5225 (-----*-----)

+---------+---------+---------+------

-3.0 0.0 3.0 6.0

Time Point = 9 subtracted from:

Time

Point Lower Center Upper +---------+---------+---------+------

10 -2.516 -0.7578 1.001 (----*-----)

+---------+---------+---------+------

-3.0 0.0 3.0 6.0

Bonferroni Simultaneous Tests

Response Variable Cq Prime

All Pairwise Comparisons among Levels of Time Point

Time Point = 3 subtracted from:

Time Difference SE of Adjusted

Point of Means Difference T-Value P-Value

4 1.475 0.5097 2.893 0.3521

5 4.146 0.5097 8.134 0.0001

6 4.726 0.5097 9.273 0.0000

7 4.806 0.5097 9.429 0.0000

8 5.531 0.5097 10.852 0.0000

9 5.053 0.5097 9.914 0.0000

10 4.295 0.5097 8.427 0.0000

Time Point = 4 subtracted from:

Time Difference SE of Adjusted

Point of Means Difference T-Value P-Value

5 2.671 0.4495 5.943 0.0014

6 3.252 0.4495 7.234 0.0002

7 3.331 0.4495 7.411 0.0001

8 4.057 0.4495 9.025 0.0000

9 3.579 0.4495 7.961 0.0001

10 2.821 0.4495 6.275 0.0008

Time Point = 5 subtracted from:

Time Difference SE of Adjusted

Point of Means Difference T-Value P-Value

6 0.5804 0.4495 1.2911 1.0000

7 0.6600 0.4495 1.4683 1.0000

8 1.3852 0.4495 3.0816 0.2450

9 0.9070 0.4495 2.0179 1.0000

10 0.1493 0.4495 0.3321 1.0000

Time Point = 6 subtracted from:

Time Difference SE of Adjusted

Point of Means Difference T-Value P-Value

7 0.0796 0.4495 0.1771 1.000

8 0.8048 0.4495 1.7904 1.000

9 0.3267 0.4495 0.7267 1.000

10 -0.4311 0.4495 -0.9591 1.000

Time Point = 7 subtracted from:

Time Difference SE of Adjusted

Point of Means Difference T-Value P-Value

8 0.7252 0.4495 1.613 1.000

9 0.2470 0.4495 0.550 1.000

10 -0.5107 0.4495 -1.136 1.000

Time Point = 8 subtracted from:

Time Difference SE of Adjusted

Point of Means Difference T-Value P-Value

9 -0.478 0.4495 -1.064 1.0000

10 -1.236 0.4495 -2.750 0.4635

Time Point = 9 subtracted from:

Time Difference SE of Adjusted

Point of Means Difference T-Value P-Value

10 -0.7578 0.4495 -1.686 1.000

Grouping Information Using Sidak Method and 95.0% Confidence

Time

Point N Mean Grouping

8 3 5.2059 A

9 3 4.7278 A

7 3 4.4807 A

6 3 4.4011 A

10 3 3.9700 A

5 3 3.8207 A

4 3 1.1493 B

3 2 -0.3253 B

Means that do not share a letter are significantly different.

Sidak 95.0% Simultaneous Confidence Intervals

Response Variable Cq Prime

All Pairwise Comparisons among Levels of Time Point

Time Point = 3 subtracted from:

Time

Point Lower Center Upper +---------+---------+---------+------

4 -0.5126 1.475 3.462 (------*------)

5 2.1589 4.146 6.133 (------*-----)

6 2.7392 4.726 6.714 (------*-----)

7 2.8189 4.806 6.793 (------*------)

8 3.5440 5.531 7.518 (-----*------)

9 3.0659 5.053 7.040 (------*-----)

10 2.3081 4.295 6.283 (-----*------)

+---------+---------+---------+------

-3.0 0.0 3.0 6.0

Time Point = 4 subtracted from:

Time

Point Lower Center Upper +---------+---------+---------+------

5 0.9189 2.671 4.424 (-----*-----)

6 1.4993 3.252 5.004 (-----*-----)

7 1.5789 3.331 5.084 (-----*-----)

8 2.3041 4.057 5.809 (-----*----)

9 1.8260 3.579 5.331 (-----*-----)

10 1.0682 2.821 4.573 (----*-----)

+---------+---------+---------+------

-3.0 0.0 3.0 6.0

Time Point = 5 subtracted from:

Time

Point Lower Center Upper +---------+---------+---------+------

6 -1.172 0.5804 2.333 (-----*-----)

7 -1.093 0.6600 2.413 (-----*-----)

8 -0.367 1.3852 3.138 (-----*----)

9 -0.846 0.9070 2.660 (-----*-----)

10 -1.603 0.1493 1.902 (----*-----)

+---------+---------+---------+------

-3.0 0.0 3.0 6.0

Time Point = 6 subtracted from:

Time

Point Lower Center Upper +---------+---------+---------+------

7 -1.673 0.0796 1.832 (-----*-----)

8 -0.948 0.8048 2.557 (-----*-----)

9 -1.426 0.3267 2.079 (-----*-----)

10 -2.184 -0.4311 1.321 (-----*----)

+---------+---------+---------+------

-3.0 0.0 3.0 6.0

Time Point = 7 subtracted from:

Time

Point Lower Center Upper +---------+---------+---------+------

8 -1.027 0.7252 2.478 (----*-----)

9 -1.506 0.2470 2.000 (-----*-----)

10 -2.263 -0.5107 1.242 (-----*-----)

+---------+---------+---------+------

-3.0 0.0 3.0 6.0

Time Point = 8 subtracted from:

Time

Point Lower Center Upper +---------+---------+---------+------

9 -2.231 -0.478 1.2744 (----*-----)

10 -2.988 -1.236 0.5166 (-----*-----)

+---------+---------+---------+------

-3.0 0.0 3.0 6.0

Time Point = 9 subtracted from:

Time

Point Lower Center Upper +---------+---------+---------+------

10 -2.510 -0.7578 0.9948 (----*-----)

+---------+---------+---------+------

-3.0 0.0 3.0 6.0

Sidak Simultaneous Tests

Response Variable Cq Prime

All Pairwise Comparisons among Levels of Time Point

Time Point = 3 subtracted from:

Time Difference SE of Adjusted

Point of Means Difference T-Value P-Value

4 1.475 0.5097 2.893 0.2983

5 4.146 0.5097 8.134 0.0001

6 4.726 0.5097 9.273 0.0000

7 4.806 0.5097 9.429 0.0000

8 5.531 0.5097 10.852 0.0000

9 5.053 0.5097 9.914 0.0000

10 4.295 0.5097 8.427 0.0000

Time Point = 4 subtracted from:

Time Difference SE of Adjusted

Point of Means Difference T-Value P-Value

5 2.671 0.4495 5.943 0.0014

6 3.252 0.4495 7.234 0.0002

7 3.331 0.4495 7.411 0.0001

8 4.057 0.4495 9.025 0.0000

9 3.579 0.4495 7.961 0.0001

10 2.821 0.4495 6.275 0.0008

Time Point = 5 subtracted from:

Time Difference SE of Adjusted

Point of Means Difference T-Value P-Value

6 0.5804 0.4495 1.2911 0.9990

7 0.6600 0.4495 1.4683 0.9938

8 1.3852 0.4495 3.0816 0.2182

9 0.9070 0.4495 2.0179 0.8465

10 0.1493 0.4495 0.3321 1.0000

Time Point = 6 subtracted from:

Time Difference SE of Adjusted

Point of Means Difference T-Value P-Value

7 0.0796 0.4495 0.1771 1.0000

8 0.8048 0.4495 1.7904 0.9420

9 0.3267 0.4495 0.7267 1.0000

10 -0.4311 0.4495 -0.9591 1.0000

Time Point = 7 subtracted from:

Time Difference SE of Adjusted

Point of Means Difference T-Value P-Value

8 0.7252 0.4495 1.613 0.9802

9 0.2470 0.4495 0.550 1.0000

10 -0.5107 0.4495 -1.136 0.9999

Time Point = 8 subtracted from:

Time Difference SE of Adjusted

Point of Means Difference T-Value P-Value

9 -0.478 0.4495 -1.064 1.0000

10 -1.236 0.4495 -2.750 0.3733

Time Point = 9 subtracted from:

Time Difference SE of Adjusted

Point of Means Difference T-Value P-Value

10 -0.7578 0.4495 -1.686 0.9680

Grouping Information Using Tukey Method and 95.0% Confidence

Time

Point N Mean Grouping

8 3 5.2059 A

9 3 4.7278 A

7 3 4.4807 A

6 3 4.4011 A

10 3 3.9700 A

5 3 3.8207 A

4 3 1.1493 B

3 2 -0.3253 B

Means that do not share a letter are significantly different.

Tukey 95.0% Simultaneous Confidence Intervals

Response Variable Cq Prime

All Pairwise Comparisons among Levels of Time Point

Time Point = 3 subtracted from:

Time

Point Lower Center Upper ---------+---------+---------+-------

4 -0.3455 1.475 3.295 (-----*-----)

5 2.3260 4.146 5.966 (-----*-----)

6 2.9064 4.726 6.546 (-----*-----)

7 2.9860 4.806 6.626 (-----*-----)

8 3.7112 5.531 7.351 (-----*------)

9 3.2330 5.053 6.873 (-----*-----)

10 2.4753 4.295 6.115 (-----*-----)

---------+---------+---------+-------

0.0 3.0 6.0

Time Point = 4 subtracted from:

Time

Point Lower Center Upper ---------+---------+---------+-------

5 1.066 2.671 4.277 (----*----)

6 1.647 3.252 4.857 (-----*----)

7 1.726 3.331 4.937 (----*----)

8 2.452 4.057 5.662 (-----*----)

9 1.973 3.579 5.184 (----*----)

10 1.216 2.821 4.426 (----*-----)

---------+---------+---------+-------

0.0 3.0 6.0

Time Point = 5 subtracted from:

Time

Point Lower Center Upper ---------+---------+---------+-------

6 -1.025 0.5804 2.186 (----*----)

7 -0.945 0.6600 2.265 (----*-----)

8 -0.220 1.3852 2.990 (-----*----)

9 -0.698 0.9070 2.512 (----*----)

10 -1.456 0.1493 1.754 (----*-----)

---------+---------+---------+-------

0.0 3.0 6.0

Time Point = 6 subtracted from:

Time

Point Lower Center Upper ---------+---------+---------+-------

7 -1.526 0.0796 1.685 (----*-----)

8 -0.800 0.8048 2.410 (-----*----)

9 -1.278 0.3267 1.932 (----*----)

10 -2.036 -0.4311 1.174 (-----*----)

---------+---------+---------+-------

0.0 3.0 6.0

Time Point = 7 subtracted from:

Time

Point Lower Center Upper ---------+---------+---------+-------

8 -0.880 0.7252 2.330 (----*-----)

9 -1.358 0.2470 1.852 (-----*----)

10 -2.116 -0.5107 1.094 (----*-----)

---------+---------+---------+-------

0.0 3.0 6.0

Time Point = 8 subtracted from:

Time

Point Lower Center Upper ---------+---------+---------+-------

9 -2.083 -0.478 1.1270 (----*-----)

10 -2.841 -1.236 0.3692 (----*----)

---------+---------+---------+-------

0.0 3.0 6.0

Time Point = 9 subtracted from:

Time

Point Lower Center Upper ---------+---------+---------+-------

10 -2.363 -0.7578 0.8474 (----*-----)

---------+---------+---------+-------

0.0 3.0 6.0

Tukey Simultaneous Tests

Response Variable Cq Prime

All Pairwise Comparisons among Levels of Time Point

Time Point = 3 subtracted from:

Time Difference SE of Adjusted

Point of Means Difference T-Value P-Value

4 1.475 0.5097 2.893 0.1527

5 4.146 0.5097 8.134 0.0001

6 4.726 0.5097 9.273 0.0000

7 4.806 0.5097 9.429 0.0000

8 5.531 0.5097 10.852 0.0000

9 5.053 0.5097 9.914 0.0000

10 4.295 0.5097 8.427 0.0000

Time Point = 4 subtracted from:

Time Difference SE of Adjusted

Point of Means Difference T-Value P-Value

5 2.671 0.4495 5.943 0.0009

6 3.252 0.4495 7.234 0.0001

7 3.331 0.4495 7.411 0.0001

8 4.057 0.4495 9.025 0.0000

9 3.579 0.4495 7.961 0.0001

10 2.821 0.4495 6.275 0.0006

Time Point = 5 subtracted from:

Time Difference SE of Adjusted

Point of Means Difference T-Value P-Value

6 0.5804 0.4495 1.2911 0.8870

7 0.6600 0.4495 1.4683 0.8107

8 1.3852 0.4495 3.0816 0.1130

9 0.9070 0.4495 2.0179 0.5062

10 0.1493 0.4495 0.3321 1.0000

Time Point = 6 subtracted from:

Time Difference SE of Adjusted

Point of Means Difference T-Value P-Value

7 0.0796 0.4495 0.1771 1.0000

8 0.8048 0.4495 1.7904 0.6361

9 0.3267 0.4495 0.7267 0.9943

10 -0.4311 0.4495 -0.9591 0.9729

Time Point = 7 subtracted from:

Time Difference SE of Adjusted

Point of Means Difference T-Value P-Value

8 0.7252 0.4495 1.613 0.7360

9 0.2470 0.4495 0.550 0.9990

10 -0.5107 0.4495 -1.136 0.9366

Time Point = 8 subtracted from:

Time Difference SE of Adjusted

Point of Means Difference T-Value P-Value

9 -0.478 0.4495 -1.064 0.9540

10 -1.236 0.4495 -2.750 0.1907

Time Point = 9 subtracted from:

Time Difference SE of Adjusted

Point of Means Difference T-Value P-Value

10 -0.7578 0.4495 -1.686 0.6958

**Residual Plots for Cq Prime**

**Main Effects Plot for Cq Prime**

PSN0025:

**————— 9/11/2012 7:56:24 PM ————————————————————**

**General Linear Model: Cq Prime versus Biounit, Time Point**

Factor Type Levels Values

Biounit random 3 1, 2, 3

Time Point fixed 8 3, 4, 5, 6, 7, 8, 9, 10

Analysis of Variance for Cq Prime, using Adjusted SS for Tests

Source DF Seq SS Adj SS Adj MS F P

Biounit 2 6.524 4.334 2.167 1.84 0.198

Time Point 7 52.604 52.604 7.515 6.38 0.002

Error 13 15.319 15.319 1.178

Total 22 74.447

S = 1.08552 R-Sq = 79.42% R-Sq(adj) = 65.18%

Term Coef SE Coef T P

Constant 3.3625 0.2294 14.66 0.000

Biounit

1 0.1617 0.3350 0.48 0.637

2 -0.5846 0.3189 -1.83 0.090

Time Point

3 -3.2816 0.7180 -4.57 0.001

4 -1.4277 0.5892 -2.42 0.031

5 -0.1206 0.5892 -0.20 0.841

6 -0.4336 0.5892 -0.74 0.475

7 0.2227 0.5892 0.38 0.712

8 1.8512 0.5892 3.14 0.008

9 1.7742 0.5892 3.01 0.010

Unusual Observations for Cq Prime

Obs Cq Prime Fit SE Fit Residual St Resid

3 4.54556 2.09656 0.71064 2.44899 2.98 R

R denotes an observation with a large standardized residual.

Expected Mean Squares, using Adjusted SS

Expected Mean Square

Source for Each Term

1 Biounit (3) + 7.5000 (1)

2 Time Point (3) + Q[2]

3 Error (3)

Error Terms for Tests, using Adjusted SS

Synthesis

Source Error DF Error MS of Error MS

1 Biounit 13.00 1.178 (3)

2 Time Point 13.00 1.178 (3)

Variance Components, using Adjusted SS

Estimated

Source Value

Biounit 0.1318

Error 1.1784

Grouping Information Using Bonferroni Method and 95.0% Confidence

Time

Point N Mean Grouping

8 3 5.21370 A

9 3 5.13667 A

10 3 4.77778 A

7 3 3.58519 A B

5 3 3.24185 A B

6 3 2.92889 A B

4 3 1.93481 A B

3 2 0.08087 B

Means that do not share a letter are significantly different.

Bonferroni 95.0% Simultaneous Confidence Intervals

Response Variable Cq Prime

All Pairwise Comparisons among Levels of Time Point

Time Point = 3 subtracted from:

Time

Point Lower Center Upper +---------+---------+---------+------

4 -2.077 1.854 5.785 (---------*--------)

5 -0.770 3.161 7.092 (---------*---------)

6 -1.083 2.848 6.779 (---------*---------)

7 -0.427 3.504 7.436 (---------*---------)

8 1.201 5.133 9.064 (---------*---------)

9 1.124 5.056 8.987 (---------*--------)

10 0.766 4.697 8.628 (---------*---------)

+---------+---------+---------+------

-4.0 0.0 4.0 8.0

Time Point = 4 subtracted from:

Time

Point Lower Center Upper +---------+---------+---------+------

5 -2.160 1.3070 4.774 (-------*--------)

6 -2.473 0.9941 4.461 (-------*--------)

7 -1.817 1.6504 5.118 (--------*--------)

8 -0.188 3.2789 6.746 (-------*--------)

9 -0.265 3.2019 6.669 (--------*--------)

10 -0.624 2.8430 6.310 (--------*--------)

+---------+---------+---------+------

-4.0 0.0 4.0 8.0

Time Point = 5 subtracted from:

Time

Point Lower Center Upper +---------+---------+---------+------

6 -3.780 -0.3130 3.154 (-------*--------)

7 -3.124 0.3433 3.811 (--------*--------)

8 -1.495 1.9719 5.439 (--------*--------)

9 -1.572 1.8948 5.362 (--------*-------)

10 -1.931 1.5359 5.003 (--------*--------)

+---------+---------+---------+------

-4.0 0.0 4.0 8.0

Time Point = 6 subtracted from:

Time

Point Lower Center Upper +---------+---------+---------+------

7 -2.811 0.6563 4.123 (--------*-------)

8 -1.182 2.2848 5.752 (--------*-------)

9 -1.259 2.2078 5.675 (--------*-------)

10 -1.618 1.8489 5.316 (--------*-------)

+---------+---------+---------+------

-4.0 0.0 4.0 8.0

Time Point = 7 subtracted from:

Time

Point Lower Center Upper +---------+---------+---------+------

8 -1.839 1.629 5.096 (--------*--------)

9 -1.916 1.551 5.019 (--------*--------)

10 -2.275 1.193 4.660 (--------*--------)

+---------+---------+---------+------

-4.0 0.0 4.0 8.0

Time Point = 8 subtracted from:

Time

Point Lower Center Upper +---------+---------+---------+------

9 -3.544 -0.0770 3.390 (--------*-------)

10 -3.903 -0.4359 3.031 (--------*--------)

+---------+---------+---------+------

-4.0 0.0 4.0 8.0

Time Point = 9 subtracted from:

Time

Point Lower Center Upper +---------+---------+---------+------

10 -3.826 -0.3589 3.108 (--------*--------)

+---------+---------+---------+------

-4.0 0.0 4.0 8.0

Bonferroni Simultaneous Tests

Response Variable Cq Prime

All Pairwise Comparisons among Levels of Time Point

Time Point = 3 subtracted from:

Time Difference SE of Adjusted

Point of Means Difference T-Value P-Value

4 1.854 1.005 1.845 1.0000

5 3.161 1.005 3.145 0.2168

6 2.848 1.005 2.834 0.3944

7 3.504 1.005 3.487 0.1124

8 5.133 1.005 5.107 0.0056

9 5.056 1.005 5.031 0.0064

10 4.697 1.005 4.674 0.0122

Time Point = 4 subtracted from:

Time Difference SE of Adjusted

Point of Means Difference T-Value P-Value

5 1.3070 0.8863 1.475 1.0000

6 0.9941 0.8863 1.122 1.0000

7 1.6504 0.8863 1.862 1.0000

8 3.2789 0.8863 3.699 0.0748

9 3.2019 0.8863 3.612 0.0884

10 2.8430 0.8863 3.208 0.1923

Time Point = 5 subtracted from:

Time Difference SE of Adjusted

Point of Means Difference T-Value P-Value

6 -0.3130 0.8863 -0.3531 1.000

7 0.3433 0.8863 0.3874 1.000

8 1.9719 0.8863 2.2247 1.000

9 1.8948 0.8863 2.1378 1.000

10 1.5359 0.8863 1.7329 1.000

Time Point = 6 subtracted from:

Time Difference SE of Adjusted

Point of Means Difference T-Value P-Value

7 0.6563 0.8863 0.7405 1.0000

8 2.2848 0.8863 2.5778 0.6426

9 2.2078 0.8863 2.4909 0.7573

10 1.8489 0.8863 2.0860 1.0000

Time Point = 7 subtracted from:

Time Difference SE of Adjusted

Point of Means Difference T-Value P-Value

8 1.629 0.8863 1.837 1.000

9 1.551 0.8863 1.750 1.000

10 1.193 0.8863 1.346 1.000

Time Point = 8 subtracted from:

Time Difference SE of Adjusted

Point of Means Difference T-Value P-Value

9 -0.0770 0.8863 -0.0869 1.000

10 -0.4359 0.8863 -0.4918 1.000

Time Point = 9 subtracted from:

Time Difference SE of Adjusted

Point of Means Difference T-Value P-Value

10 -0.3589 0.8863 -0.4049 1.000

Grouping Information Using Sidak Method and 95.0% Confidence

Time

Point N Mean Grouping

8 3 5.21370 A

9 3 5.13667 A

10 3 4.77778 A

7 3 3.58519 A B

5 3 3.24185 A B

6 3 2.92889 A B

4 3 1.93481 A B

3 2 0.08087 B

Means that do not share a letter are significantly different.

Sidak 95.0% Simultaneous Confidence Intervals

Response Variable Cq Prime

All Pairwise Comparisons among Levels of Time Point

Time Point = 3 subtracted from:

Time

Point Lower Center Upper -+---------+---------+---------+-----

4 -2.064 1.854 5.772 (----------*----------)

5 -0.757 3.161 7.079 (----------*----------)

6 -1.070 2.848 6.766 (----------*----------)

7 -0.414 3.504 7.423 (----------*----------)

8 1.215 5.133 9.051 (-----------*----------)

9 1.137 5.056 8.974 (----------*-----------)

10 0.779 4.697 8.615 (----------*-----------)

-+---------+---------+---------+-----

-3.5 0.0 3.5 7.0

Time Point = 4 subtracted from:

Time

Point Lower Center Upper -+---------+---------+---------+-----

5 -2.149 1.3070 4.763 (---------*---------)

6 -2.462 0.9941 4.450 (---------*---------)

7 -1.805 1.6504 5.106 (---------*---------)

8 -0.177 3.2789 6.735 (---------*---------)

9 -0.254 3.2019 6.657 (---------*---------)

10 -0.613 2.8430 6.299 (---------*---------)

-+---------+---------+---------+-----

-3.5 0.0 3.5 7.0

Time Point = 5 subtracted from:

Time

Point Lower Center Upper -+---------+---------+---------+-----

6 -3.769 -0.3130 3.143 (---------*---------)

7 -3.112 0.3433 3.799 (---------*---------)

8 -1.484 1.9719 5.427 (---------*---------)

9 -1.561 1.8948 5.350 (--------*---------)

10 -1.920 1.5359 4.992 (--------*---------)

-+---------+---------+---------+-----

-3.5 0.0 3.5 7.0

Time Point = 6 subtracted from:

Time

Point Lower Center Upper -+---------+---------+---------+-----

7 -2.799 0.6563 4.112 (---------*---------)

8 -1.171 2.2848 5.740 (---------*--------)

9 -1.248 2.2078 5.663 (---------*---------)

10 -1.607 1.8489 5.305 (---------*---------)

-+---------+---------+---------+-----

-3.5 0.0 3.5 7.0

Time Point = 7 subtracted from:

Time

Point Lower Center Upper -+---------+---------+---------+-----

8 -1.827 1.629 5.084 (---------*---------)

9 -1.904 1.551 5.007 (--------*---------)

10 -2.263 1.193 4.648 (--------*---------)

-+---------+---------+---------+-----

-3.5 0.0 3.5 7.0

Time Point = 8 subtracted from:

Time

Point Lower Center Upper -+---------+---------+---------+-----

9 -3.533 -0.0770 3.379 (---------*---------)

10 -3.892 -0.4359 3.020 (---------*---------)

-+---------+---------+---------+-----

-3.5 0.0 3.5 7.0

Time Point = 9 subtracted from:

Time

Point Lower Center Upper -+---------+---------+---------+-----

10 -3.815 -0.3589 3.097 (---------*---------)

-+---------+---------+---------+-----

-3.5 0.0 3.5 7.0

Sidak Simultaneous Tests

Response Variable Cq Prime

All Pairwise Comparisons among Levels of Time Point

Time Point = 3 subtracted from:

Time Difference SE of Adjusted

Point of Means Difference T-Value P-Value

4 1.854 1.005 1.845 0.9241

5 3.161 1.005 3.145 0.1956

6 2.848 1.005 2.834 0.3278

7 3.504 1.005 3.487 0.1065

8 5.133 1.005 5.107 0.0056

9 5.056 1.005 5.031 0.0064

10 4.697 1.005 4.674 0.0121

Time Point = 4 subtracted from:

Time Difference SE of Adjusted

Point of Means Difference T-Value P-Value

5 1.3070 0.8863 1.475 0.9934

6 0.9941 0.8863 1.122 0.9999

7 1.6504 0.8863 1.862 0.9178

8 3.2789 0.8863 3.699 0.0722

9 3.2019 0.8863 3.612 0.0847

10 2.8430 0.8863 3.208 0.1755

Time Point = 5 subtracted from:

Time Difference SE of Adjusted

Point of Means Difference T-Value P-Value

6 -0.3130 0.8863 -0.3531 1.0000

7 0.3433 0.8863 0.3874 1.0000

8 1.9719 0.8863 2.2247 0.7199

9 1.8948 0.8863 2.1378 0.7765

10 1.5359 0.8863 1.7329 0.9576

Time Point = 6 subtracted from:

Time Difference SE of Adjusted

Point of Means Difference T-Value P-Value

7 0.6563 0.8863 0.7405 1.0000

8 2.2848 0.8863 2.5778 0.4780

9 2.2078 0.8863 2.4909 0.5359

10 1.8489 0.8863 2.0860 0.8081

Time Point = 7 subtracted from:

Time Difference SE of Adjusted

Point of Means Difference T-Value P-Value

8 1.629 0.8863 1.837 0.9267

9 1.551 0.8863 1.750 0.9532

10 1.193 0.8863 1.346 0.9982

Time Point = 8 subtracted from:

Time Difference SE of Adjusted

Point of Means Difference T-Value P-Value

9 -0.0770 0.8863 -0.0869 1.000

10 -0.4359 0.8863 -0.4918 1.000

Time Point = 9 subtracted from:

Time Difference SE of Adjusted

Point of Means Difference T-Value P-Value

10 -0.3589 0.8863 -0.4049 1.000

Grouping Information Using Tukey Method and 95.0% Confidence

Time

Point N Mean Grouping

8 3 5.21370 A

9 3 5.13667 A

10 3 4.77778 A B

7 3 3.58519 A B C

5 3 3.24185 A B C

6 3 2.92889 A B C

4 3 1.93481 B C

3 2 0.08087 C

Means that do not share a letter are significantly different.

Tukey 95.0% Simultaneous Confidence Intervals

Response Variable Cq Prime

All Pairwise Comparisons among Levels of Time Point

Time Point = 3 subtracted from:

Time

Point Lower Center Upper +---------+---------+---------+------

4 -1.735 1.854 5.443 (---------*----------)

5 -0.428 3.161 6.750 (---------*---------)

6 -0.741 2.848 6.437 (---------*---------)

7 -0.084 3.504 7.093 (---------*---------)

8 1.544 5.133 8.722 (----------*---------)

9 1.467 5.056 8.645 (---------*----------)

10 1.108 4.697 8.286 (---------*----------)

+---------+---------+---------+------

-3.5 0.0 3.5 7.0

Time Point = 4 subtracted from:

Time

Point Lower Center Upper +---------+---------+---------+------

5 -1.858 1.3070 4.472 (--------*--------)

6 -2.171 0.9941 4.159 (--------*--------)

7 -1.515 1.6504 4.815 (--------*--------)

8 0.114 3.2789 6.444 (--------*--------)

9 0.037 3.2019 6.367 (--------*--------)

10 -0.322 2.8430 6.008 (--------*--------)

+---------+---------+---------+------

-3.5 0.0 3.5 7.0

Time Point = 5 subtracted from:

Time

Point Lower Center Upper +---------+---------+---------+------

6 -3.478 -0.3130 2.852 (--------*--------)

7 -2.822 0.3433 3.508 (--------*--------)

8 -1.193 1.9719 5.137 (--------*--------)

9 -1.270 1.8948 5.060 (--------*--------)

10 -1.629 1.5359 4.701 (--------*--------)

+---------+---------+---------+------

-3.5 0.0 3.5 7.0

Time Point = 6 subtracted from:

Time

Point Lower Center Upper +---------+---------+---------+------

7 -2.509 0.6563 3.821 (--------*--------)

8 -0.880 2.2848 5.450 (---------*--------)

9 -0.957 2.2078 5.373 (--------*--------)

10 -1.316 1.8489 5.014 (--------*--------)

+---------+---------+---------+------

-3.5 0.0 3.5 7.0

Time Point = 7 subtracted from:

Time

Point Lower Center Upper +---------+---------+---------+------

8 -1.536 1.629 4.793 (--------*--------)

9 -1.613 1.551 4.716 (--------*--------)

10 -1.972 1.193 4.358 (--------*--------)

+---------+---------+---------+------

-3.5 0.0 3.5 7.0

Time Point = 8 subtracted from:

Time

Point Lower Center Upper +---------+---------+---------+------

9 -3.242 -0.0770 3.088 (--------*--------)

10 -3.601 -0.4359 2.729 (--------*--------)

+---------+---------+---------+------

-3.5 0.0 3.5 7.0

Time Point = 9 subtracted from:

Time

Point Lower Center Upper +---------+---------+---------+------

10 -3.524 -0.3589 2.806 (--------*--------)

+---------+---------+---------+------

-3.5 0.0 3.5 7.0

Tukey Simultaneous Tests

Response Variable Cq Prime

All Pairwise Comparisons among Levels of Time Point

Time Point = 3 subtracted from:

Time Difference SE of Adjusted

Point of Means Difference T-Value P-Value

4 1.854 1.005 1.845 0.6048

5 3.161 1.005 3.145 0.1019

6 2.848 1.005 2.834 0.1675

7 3.504 1.005 3.487 0.0576

8 5.133 1.005 5.107 0.0036

9 5.056 1.005 5.031 0.0041

10 4.697 1.005 4.674 0.0075

Time Point = 4 subtracted from:

Time Difference SE of Adjusted

Point of Means Difference T-Value P-Value

5 1.3070 0.8863 1.475 0.8076

6 0.9941 0.8863 1.122 0.9404

7 1.6504 0.8863 1.862 0.5948

8 3.2789 0.8863 3.699 0.0401

9 3.2019 0.8863 3.612 0.0465

10 2.8430 0.8863 3.208 0.0920

Time Point = 5 subtracted from:

Time Difference SE of Adjusted

Point of Means Difference T-Value P-Value

6 -0.3130 0.8863 -0.3531 0.9999

7 0.3433 0.8863 0.3874 0.9999

8 1.9719 0.8863 2.2247 0.3969

9 1.8948 0.8863 2.1378 0.4413

10 1.5359 0.8863 1.7329 0.6692

Time Point = 6 subtracted from:

Time Difference SE of Adjusted

Point of Means Difference T-Value P-Value

7 0.6563 0.8863 0.7405 0.9937

8 2.2848 0.8863 2.5778 0.2460

9 2.2078 0.8863 2.4909 0.2785

10 1.8489 0.8863 2.0860 0.4688

Time Point = 7 subtracted from:

Time Difference SE of Adjusted

Point of Means Difference T-Value P-Value

8 1.629 0.8863 1.837 0.6091

9 1.551 0.8863 1.750 0.6591

10 1.193 0.8863 1.346 0.8656

Time Point = 8 subtracted from:

Time Difference SE of Adjusted

Point of Means Difference T-Value P-Value

9 -0.0770 0.8863 -0.0869 1.0000

10 -0.4359 0.8863 -0.4918 0.9995

Time Point = 9 subtracted from:

Time Difference SE of Adjusted

Point of Means Difference T-Value P-Value

10 -0.3589 0.8863 -0.4049 0.9999

**Residual Plots for Cq Prime**

**Main Effects Plot for Cq Prime**

Psn0072:

**————— 9/11/2012 7:56:24 PM ————————————————————**

**General Linear Model: Cq Prime versus Biounit, Time Point**

Factor Type Levels Values

Biounit random 3 1, 2, 3

Time Point fixed 8 3, 4, 5, 6, 7, 8, 9, 10

Analysis of Variance for Cq Prime, using Adjusted SS for Tests

Source DF Seq SS Adj SS Adj MS F P

Biounit 2 0.191 1.793 0.897 1.34 0.295

Time Point 7 94.642 94.642 13.520 20.28 0.000

Error 13 8.667 8.667 0.667

Total 22 103.500

S = 0.816490 R-Sq = 91.63% R-Sq(adj) = 85.83%

Term Coef SE Coef T P

Constant 5.4693 0.1725 31.70 0.000

Biounit

1 -0.4121 0.2520 -1.64 0.126

2 0.1807 0.2399 0.75 0.465

Time Point

3 -5.6753 0.5401 -10.51 0.000

4 -1.4951 0.4432 -3.37 0.005

5 0.7698 0.4432 1.74 0.106

6 1.2812 0.4432 2.89 0.013

7 1.3153 0.4432 2.97 0.011

8 1.5483 0.4432 3.49 0.004

9 1.6112 0.4432 3.64 0.003

Unusual Observations for Cq Prime

Obs Cq Prime Fit SE Fit Residual St Resid

3 5.20722 3.56220 0.53452 1.64503 2.67 R

5 2.94056 4.20564 0.52892 -1.26508 -2.03 R

R denotes an observation with a large standardized residual.

Expected Mean Squares, using Adjusted SS

Expected Mean Square

Source for Each Term

1 Biounit (3) + 7.5000 (1)

2 Time Point (3) + Q[2]

3 Error (3)

Error Terms for Tests, using Adjusted SS

Synthesis

Source Error DF Error MS of Error MS

1 Biounit 13.00 0.667 (3)

2 Time Point 13.00 0.667 (3)

Variance Components, using Adjusted SS

Estimated

Source Value

Biounit 0.03066

Error 0.66666

Grouping Information Using Bonferroni Method and 95.0% Confidence

Time

Point N Mean Grouping

9 3 7.0806 A

8 3 7.0176 A

7 3 6.7846 A

6 3 6.7506 A

5 3 6.2391 A B

10 3 6.1139 A B

4 3 3.9743 B

3 2 -0.2060 C

Means that do not share a letter are significantly different.

Bonferroni 95.0% Simultaneous Confidence Intervals

Response Variable Cq Prime

All Pairwise Comparisons among Levels of Time Point

Time Point = 3 subtracted from:

Time

Point Lower Center Upper ---------+---------+---------+-------

4 1.223 4.180 7.137 (------*-------)

5 3.488 6.445 9.402 (------*-------)

6 4.000 6.957 9.914 (------*-------)

7 4.034 6.991 9.948 (------*-------)

8 4.267 7.224 10.181 (------*------)

9 4.330 7.287 10.244 (------*-------)

10 3.363 6.320 9.277 (-------*------)

---------+---------+---------+-------

0.0 4.0 8.0

Time Point = 4 subtracted from:

Time

Point Lower Center Upper ---------+---------+---------+-------

5 -0.3431 2.265 4.873 (------*-----)

6 0.1684 2.776 5.384 (------*-----)

7 0.2025 2.810 5.418 (-----*------)

8 0.4355 3.043 5.651 (------*-----)

9 0.4984 3.106 5.714 (------*-----)

10 -0.4682 2.140 4.748 (-----*------)

---------+---------+---------+-------

0.0 4.0 8.0

Time Point = 5 subtracted from:

Time

Point Lower Center Upper ---------+---------+---------+-------

6 -2.096 0.5115 3.119 (-----*------)

7 -2.062 0.5456 3.153 (-----*------)

8 -1.829 0.7785 3.386 (------*-----)

9 -1.766 0.8415 3.449 (-----*------)

10 -2.733 -0.1252 2.483 (------*-----)

---------+---------+---------+-------

0.0 4.0 8.0

Time Point = 6 subtracted from:

Time

Point Lower Center Upper ---------+---------+---------+-------

7 -2.574 0.0341 2.642 (-----*------)

8 -2.341 0.2670 2.875 (------*-----)

9 -2.278 0.3300 2.938 (------*-----)

10 -3.245 -0.6367 1.971 (-----*------)

---------+---------+---------+-------

0.0 4.0 8.0

Time Point = 7 subtracted from:

Time

Point Lower Center Upper ---------+---------+---------+-------

8 -2.375 0.2330 2.841 (------*-----)

9 -2.312 0.2959 2.904 (------*-----)

10 -3.279 -0.6707 1.937 (-----*------)

---------+---------+---------+-------

0.0 4.0 8.0

Time Point = 8 subtracted from:

Time

Point Lower Center Upper ---------+---------+---------+-------

9 -2.545 0.0630 2.671 (-----*------)

10 -3.512 -0.9037 1.704 (------*-----)

---------+---------+---------+-------

0.0 4.0 8.0

Time Point = 9 subtracted from:

Time

Point Lower Center Upper ---------+---------+---------+-------

10 -3.575 -0.9667 1.641 (------*-----)

---------+---------+---------+-------

0.0 4.0 8.0

Bonferroni Simultaneous Tests

Response Variable Cq Prime

All Pairwise Comparisons among Levels of Time Point

Time Point = 3 subtracted from:

Time Difference SE of Adjusted

Point of Means Difference T-Value P-Value

4 4.180 0.7559 5.530 0.0027

5 6.445 0.7559 8.526 0.0000

6 6.957 0.7559 9.203 0.0000

7 6.991 0.7559 9.248 0.0000

8 7.224 0.7559 9.556 0.0000

9 7.287 0.7559 9.639 0.0000

10 6.320 0.7559 8.361 0.0000

Time Point = 4 subtracted from:

Time Difference SE of Adjusted

Point of Means Difference T-Value P-Value

5 2.265 0.6667 3.397 0.1335

6 2.776 0.6667 4.164 0.0311

7 2.810 0.6667 4.216 0.0283

8 3.043 0.6667 4.565 0.0149

9 3.106 0.6667 4.659 0.0125

10 2.140 0.6667 3.209 0.1916

Time Point = 5 subtracted from:

Time Difference SE of Adjusted

Point of Means Difference T-Value P-Value

6 0.5115 0.6667 0.7672 1.000

7 0.5456 0.6667 0.8183 1.000

8 0.7785 0.6667 1.1678 1.000

9 0.8415 0.6667 1.2622 1.000

10 -0.1252 0.6667 -0.1878 1.000

Time Point = 6 subtracted from:

Time Difference SE of Adjusted

Point of Means Difference T-Value P-Value

7 0.0341 0.6667 0.0511 1.000

8 0.2670 0.6667 0.4006 1.000

9 0.3300 0.6667 0.4950 1.000

10 -0.6367 0.6667 -0.9550 1.000

Time Point = 7 subtracted from:

Time Difference SE of Adjusted

Point of Means Difference T-Value P-Value

8 0.2330 0.6667 0.349 1.000

9 0.2959 0.6667 0.444 1.000

10 -0.6707 0.6667 -1.006 1.000

Time Point = 8 subtracted from:

Time Difference SE of Adjusted

Point of Means Difference T-Value P-Value

9 0.0630 0.6667 0.094 1.000

10 -0.9037 0.6667 -1.356 1.000

Time Point = 9 subtracted from:

Time Difference SE of Adjusted

Point of Means Difference T-Value P-Value

10 -0.9667 0.6667 -1.450 1.000

Grouping Information Using Sidak Method and 95.0% Confidence

Time

Point N Mean Grouping

9 3 7.0806 A

8 3 7.0176 A

7 3 6.7846 A

6 3 6.7506 A

5 3 6.2391 A B

10 3 6.1139 A B

4 3 3.9743 B

3 2 -0.2060 C

Means that do not share a letter are significantly different.

Sidak 95.0% Simultaneous Confidence Intervals

Response Variable Cq Prime

All Pairwise Comparisons among Levels of Time Point

Time Point = 3 subtracted from:

Time

Point Lower Center Upper ---------+---------+---------+-------

4 1.233 4.180 7.128 (------*-------)

5 3.498 6.445 9.392 (------*------)

6 4.009 6.957 9.904 (------*-------)

7 4.043 6.991 9.938 (------*-------)

8 4.276 7.224 10.171 (------*------)

9 4.339 7.287 10.234 (------*-------)

10 3.373 6.320 9.267 (-------*------)

---------+---------+---------+-------

0.0 4.0 8.0

Time Point = 4 subtracted from:

Time

Point Lower Center Upper ---------+---------+---------+-------

5 -0.3344 2.265 4.864 (------*-----)

6 0.1771 2.776 5.375 (------*-----)

7 0.2112 2.810 5.410 (-----*------)

8 0.4441 3.043 5.643 (------*-----)

9 0.5071 3.106 5.705 (------*-----)

10 -0.4596 2.140 4.739 (-----*------)

---------+---------+---------+-------

0.0 4.0 8.0

Time Point = 5 subtracted from:

Time

Point Lower Center Upper ---------+---------+---------+-------

6 -2.088 0.5115 3.111 (-----*------)

7 -2.054 0.5456 3.145 (-----*------)

8 -1.821 0.7785 3.378 (------*-----)

9 -1.758 0.8415 3.441 (-----*------)

10 -2.724 -0.1252 2.474 (------*-----)

---------+---------+---------+-------

0.0 4.0 8.0

Time Point = 6 subtracted from:

Time

Point Lower Center Upper ---------+---------+---------+-------

7 -2.565 0.0341 2.633 (-----*------)

8 -2.332 0.2670 2.866 (------*-----)

9 -2.269 0.3300 2.929 (------*-----)

10 -3.236 -0.6367 1.963 (-----*------)

---------+---------+---------+-------

0.0 4.0 8.0

Time Point = 7 subtracted from:

Time

Point Lower Center Upper ---------+---------+---------+-------

8 -2.366 0.2330 2.832 (------*-----)

9 -2.303 0.2959 2.895 (------*-----)

10 -3.270 -0.6707 1.928 (-----*------)

---------+---------+---------+-------

0.0 4.0 8.0

Time Point = 8 subtracted from:

Time

Point Lower Center Upper ---------+---------+---------+-------

9 -2.536 0.0630 2.662 (-----*------)

10 -3.503 -0.9037 1.695 (------*-----)

---------+---------+---------+-------

0.0 4.0 8.0

Time Point = 9 subtracted from:

Time

Point Lower Center Upper ---------+---------+---------+-------

10 -3.566 -0.9667 1.633 (------*-----)

---------+---------+---------+-------

0.0 4.0 8.0

Sidak Simultaneous Tests

Response Variable Cq Prime

All Pairwise Comparisons among Levels of Time Point

Time Point = 3 subtracted from:

Time Difference SE of Adjusted

Point of Means Difference T-Value P-Value

4 4.180 0.7559 5.530 0.0027

5 6.445 0.7559 8.526 0.0000

6 6.957 0.7559 9.203 0.0000

7 6.991 0.7559 9.248 0.0000

8 7.224 0.7559 9.556 0.0000

9 7.287 0.7559 9.639 0.0000

10 6.320 0.7559 8.361 0.0000

Time Point = 4 subtracted from:

Time Difference SE of Adjusted

Point of Means Difference T-Value P-Value

5 2.265 0.6667 3.397 0.1252

6 2.776 0.6667 4.164 0.0306

7 2.810 0.6667 4.216 0.0279

8 3.043 0.6667 4.565 0.0147

9 3.106 0.6667 4.659 0.0124

10 2.140 0.6667 3.209 0.1749

Time Point = 5 subtracted from:

Time Difference SE of Adjusted

Point of Means Difference T-Value P-Value

6 0.5115 0.6667 0.7672 1.0000

7 0.5456 0.6667 0.8183 1.0000

8 0.7785 0.6667 1.1678 0.9998

9 0.8415 0.6667 1.2622 0.9993

10 -0.1252 0.6667 -0.1878 1.0000

Time Point = 6 subtracted from:

Time Difference SE of Adjusted

Point of Means Difference T-Value P-Value

7 0.0341 0.6667 0.0511 1.000

8 0.2670 0.6667 0.4006 1.000

9 0.3300 0.6667 0.4950 1.000

10 -0.6367 0.6667 -0.9550 1.000

Time Point = 7 subtracted from:

Time Difference SE of Adjusted

Point of Means Difference T-Value P-Value

8 0.2330 0.6667 0.349 1.000

9 0.2959 0.6667 0.444 1.000

10 -0.6707 0.6667 -1.006 1.000

Time Point = 8 subtracted from:

Time Difference SE of Adjusted

Point of Means Difference T-Value P-Value

9 0.0630 0.6667 0.094 1.0000

10 -0.9037 0.6667 -1.356 0.9979

Time Point = 9 subtracted from:

Time Difference SE of Adjusted

Point of Means Difference T-Value P-Value

10 -0.9667 0.6667 -1.450 0.9947

Grouping Information Using Tukey Method and 95.0% Confidence

Time

Point N Mean Grouping

9 3 7.0806 A

8 3 7.0176 A

7 3 6.7846 A

6 3 6.7506 A

5 3 6.2391 A B

10 3 6.1139 A B

4 3 3.9743 B

3 2 -0.2060 C

Means that do not share a letter are significantly different.

Tukey 95.0% Simultaneous Confidence Intervals

Response Variable Cq Prime

All Pairwise Comparisons among Levels of Time Point

Time Point = 3 subtracted from:

Time

Point Lower Center Upper --------+---------+---------+--------

4 1.481 4.180 6.880 (-----*------)

5 3.746 6.445 9.144 (------*------)

6 4.257 6.957 9.656 (-----*------)

7 4.291 6.991 9.690 (-----*------)

8 4.524 7.224 9.923 (------*------)

9 4.587 7.287 9.986 (------*------)

10 3.621 6.320 9.019 (------*------)

--------+---------+---------+--------

0.0 4.0 8.0

Time Point = 4 subtracted from:

Time

Point Lower Center Upper --------+---------+---------+--------

5 -0.1158 2.265 4.645 (-----*-----)

6 0.3957 2.776 5.157 (-----*-----)

7 0.4298 2.810 5.191 (-----*-----)

8 0.6628 3.043 5.424 (-----*-----)

9 0.7257 3.106 5.487 (-----*-----)

10 -0.2409 2.140 4.520 (-----*-----)

--------+---------+---------+--------

0.0 4.0 8.0

Time Point = 5 subtracted from:

Time

Point Lower Center Upper --------+---------+---------+--------

6 -1.869 0.5115 2.892 (-----*-----)

7 -1.835 0.5456 2.926 (-----*-----)

8 -1.602 0.7785 3.159 (-----*-----)

9 -1.539 0.8415 3.222 (-----*-----)

10 -2.506 -0.1252 2.255 (-----*-----)

--------+---------+---------+--------

0.0 4.0 8.0

Time Point = 6 subtracted from:

Time

Point Lower Center Upper --------+---------+---------+--------

7 -2.346 0.0341 2.415 (-----*-----)

8 -2.114 0.2670 2.648 (-----*-----)

9 -2.051 0.3300 2.711 (-----*-----)

10 -3.017 -0.6367 1.744 (-----*-----)

--------+---------+---------+--------

0.0 4.0 8.0

Time Point = 7 subtracted from:

Time

Point Lower Center Upper --------+---------+---------+--------

8 -2.148 0.2330 2.614 (-----*-----)

9 -2.085 0.2959 2.676 (-----*-----)

10 -3.051 -0.6707 1.710 (-----*-----)

--------+---------+---------+--------

0.0 4.0 8.0

Time Point = 8 subtracted from:

Time

Point Lower Center Upper --------+---------+---------+--------

9 -2.318 0.0630 2.444 (-----*-----)

10 -3.284 -0.9037 1.477 (-----*-----)

--------+---------+---------+--------

0.0 4.0 8.0

Time Point = 9 subtracted from:

Time

Point Lower Center Upper --------+---------+---------+--------

10 -3.347 -0.9667 1.414 (-----*-----)

--------+---------+---------+--------

0.0 4.0 8.0

Tukey Simultaneous Tests

Response Variable Cq Prime

All Pairwise Comparisons among Levels of Time Point

Time Point = 3 subtracted from:

Time Difference SE of Adjusted

Point of Means Difference T-Value P-Value

4 4.180 0.7559 5.530 0.0018

5 6.445 0.7559 8.526 0.0000

6 6.957 0.7559 9.203 0.0000

7 6.991 0.7559 9.248 0.0000

8 7.224 0.7559 9.556 0.0000

9 7.287 0.7559 9.639 0.0000

10 6.320 0.7559 8.361 0.0000

Time Point = 4 subtracted from:

Time Difference SE of Adjusted

Point of Means Difference T-Value P-Value

5 2.265 0.6667 3.397 0.0670

6 2.776 0.6667 4.164 0.0180

7 2.810 0.6667 4.216 0.0165

8 3.043 0.6667 4.565 0.0090

9 3.106 0.6667 4.659 0.0077

10 2.140 0.6667 3.209 0.0917

Time Point = 5 subtracted from:

Time Difference SE of Adjusted

Point of Means Difference T-Value P-Value

6 0.5115 0.6667 0.7672 0.9922

7 0.5456 0.6667 0.8183 0.9887

8 0.7785 0.6667 1.1678 0.9278

9 0.8415 0.6667 1.2622 0.8975

10 -0.1252 0.6667 -0.1878 1.0000

Time Point = 6 subtracted from:

Time Difference SE of Adjusted

Point of Means Difference T-Value P-Value

7 0.0341 0.6667 0.0511 1.0000

8 0.2670 0.6667 0.4006 0.9999

9 0.3300 0.6667 0.4950 0.9995

10 -0.6367 0.6667 -0.9550 0.9736

Time Point = 7 subtracted from:

Time Difference SE of Adjusted

Point of Means Difference T-Value P-Value

8 0.2330 0.6667 0.349 0.9999

9 0.2959 0.6667 0.444 0.9997

10 -0.6707 0.6667 -1.006 0.9653

Time Point = 8 subtracted from:

Time Difference SE of Adjusted

Point of Means Difference T-Value P-Value

9 0.0630 0.6667 0.094 1.0000

10 -0.9037 0.6667 -1.356 0.8615

Time Point = 9 subtracted from:

Time Difference SE of Adjusted

Point of Means Difference T-Value P-Value

10 -0.9667 0.6667 -1.450 0.8194

**Residual Plots for Cq Prime**

**Main Effects Plot for Cq Prime**

Psn0072 EX:

**————— 9/11/2012 7:56:24 PM ————————————————————**

**General Linear Model: Cq Prime versus Biounit, Time Point**

Factor Type Levels Values

Biounit random 3 1, 2, 3

Time Point fixed 8 3, 4, 5, 6, 7, 8, 9, 10

Analysis of Variance for Cq Prime, using Adjusted SS for Tests

Source DF Seq SS Adj SS Adj MS F P

Biounit 2 0.264 1.816 0.908 1.31 0.304

Time Point 7 100.483 100.483 14.355 20.65 0.000

Error 13 9.039 9.039 0.695

Total 22 109.786

S = 0.833842 R-Sq = 91.77% R-Sq(adj) = 86.07%

Term Coef SE Coef T P

Constant 5.6517 0.1762 32.08 0.000

Biounit

1 -0.4122 0.2573 -1.60 0.133

2 0.1616 0.2450 0.66 0.521

Time Point

3 -5.8578 0.5515 -10.62 0.000

4 -1.5258 0.4526 -3.37 0.005

5 0.8401 0.4526 1.86 0.086

6 1.3305 0.4526 2.94 0.012

7 1.3246 0.4526 2.93 0.012

8 1.5797 0.4526 3.49 0.004

9 1.6705 0.4526 3.69 0.003

Unusual Observations for Cq Prime

Obs Cq Prime Fit SE Fit Residual St Resid

3 5.41556 3.71370 0.54588 1.70185 2.70 R

5 3.08889 4.37655 0.54016 -1.28766 -2.03 R

R denotes an observation with a large standardized residual.

Expected Mean Squares, using Adjusted SS

Expected Mean Square

Source for Each Term

1 Biounit (3) + 7.5000 (1)

2 Time Point (3) + Q[2]

3 Error (3)

Error Terms for Tests, using Adjusted SS

Synthesis

Source Error DF Error MS of Error MS

1 Biounit 13.00 0.695 (3)

2 Time Point 13.00 0.695 (3)

Variance Components, using Adjusted SS

Estimated

Source Value

Biounit 0.02836

Error 0.69529

Grouping Information Using Bonferroni Method and 95.0% Confidence

Time

Point N Mean Grouping

9 3 7.3222 A

8 3 7.2315 A

6 3 6.9822 A

7 3 6.9763 A

5 3 6.4919 A B

10 3 6.2900 A B

4 3 4.1259 B

3 2 -0.2061 C

Means that do not share a letter are significantly different.

Bonferroni 95.0% Simultaneous Confidence Intervals

Response Variable Cq Prime

All Pairwise Comparisons among Levels of Time Point

Time Point = 3 subtracted from:

Time

Point Lower Center Upper ---------+---------+---------+-------

4 1.312 4.332 7.352 (-------*------)

5 3.678 6.698 9.718 (-------*------)

6 4.168 7.188 10.208 (-------*-------)

7 4.163 7.182 10.202 (-------*-------)

8 4.418 7.438 10.457 (-------*------)

9 4.508 7.528 10.548 (-------*------)

10 3.476 6.496 9.516 (------*-------)

---------+---------+---------+-------

0.0 4.0 8.0

Time Point = 4 subtracted from:

Time

Point Lower Center Upper ---------+---------+---------+-------

5 -0.2974 2.366 5.029 (------*------)

6 0.1930 2.856 5.520 (------*------)

7 0.1871 2.850 5.514 (------*------)

8 0.4423 3.106 5.769 (------*-----)

9 0.5330 3.196 5.860 (------*------)

10 -0.4992 2.164 4.827 (-----*------)

---------+---------+---------+-------

0.0 4.0 8.0

Time Point = 5 subtracted from:

Time

Point Lower Center Upper ---------+---------+---------+-------

6 -2.173 0.4904 3.154 (-----*------)

7 -2.179 0.4844 3.148 (-----*------)

8 -1.924 0.7396 3.403 (------*------)

9 -1.833 0.8304 3.494 (------*------)

10 -2.865 -0.2019 2.461 (-----*------)

---------+---------+---------+-------

0.0 4.0 8.0

Time Point = 6 subtracted from:

Time

Point Lower Center Upper ---------+---------+---------+-------

7 -2.669 -0.0059 2.657 (------*------)

8 -2.414 0.2493 2.913 (------*-----)

9 -2.323 0.3400 3.003 (------*------)

10 -3.356 -0.6922 1.971 (-----*------)

---------+---------+---------+-------

0.0 4.0 8.0

Time Point = 7 subtracted from:

Time

Point Lower Center Upper ---------+---------+---------+-------

8 -2.408 0.2552 2.918 (------*-----)

9 -2.317 0.3459 3.009 (------*------)

10 -3.350 -0.6863 1.977 (-----*------)

---------+---------+---------+-------

0.0 4.0 8.0

Time Point = 8 subtracted from:

Time

Point Lower Center Upper ---------+---------+---------+-------

9 -2.573 0.0907 2.754 (-----*------)

10 -3.605 -0.9415 1.722 (------*-----)

---------+---------+---------+-------

0.0 4.0 8.0

Time Point = 9 subtracted from:

Time

Point Lower Center Upper ---------+---------+---------+-------

10 -3.696 -1.032 1.631 (-----*------)

---------+---------+---------+-------

0.0 4.0 8.0

Bonferroni Simultaneous Tests

Response Variable Cq Prime

All Pairwise Comparisons among Levels of Time Point

Time Point = 3 subtracted from:

Time Difference SE of Adjusted

Point of Means Difference T-Value P-Value

4 4.332 0.7720 5.612 0.0024

5 6.698 0.7720 8.676 0.0000

6 7.188 0.7720 9.311 0.0000

7 7.182 0.7720 9.304 0.0000

8 7.438 0.7720 9.634 0.0000

9 7.528 0.7720 9.752 0.0000

10 6.496 0.7720 8.415 0.0000

Time Point = 4 subtracted from:

Time Difference SE of Adjusted

Point of Means Difference T-Value P-Value

5 2.366 0.6808 3.475 0.1150

6 2.856 0.6808 4.195 0.0294

7 2.850 0.6808 4.187 0.0298

8 3.106 0.6808 4.561 0.0149

9 3.196 0.6808 4.695 0.0117

10 2.164 0.6808 3.179 0.2033

Time Point = 5 subtracted from:

Time Difference SE of Adjusted

Point of Means Difference T-Value P-Value

6 0.4904 0.6808 0.7203 1.000

7 0.4844 0.6808 0.7116 1.000

8 0.7396 0.6808 1.0864 1.000

9 0.8304 0.6808 1.2196 1.000

10 -0.2019 0.6808 -0.2965 1.000

Time Point = 6 subtracted from:

Time Difference SE of Adjusted

Point of Means Difference T-Value P-Value

7 -0.0059 0.6808 -0.009 1.000

8 0.2493 0.6808 0.366 1.000

9 0.3400 0.6808 0.499 1.000

10 -0.6922 0.6808 -1.017 1.000

Time Point = 7 subtracted from:

Time Difference SE of Adjusted

Point of Means Difference T-Value P-Value

8 0.2552 0.6808 0.375 1.000

9 0.3459 0.6808 0.508 1.000

10 -0.6863 0.6808 -1.008 1.000

Time Point = 8 subtracted from:

Time Difference SE of Adjusted

Point of Means Difference T-Value P-Value

9 0.0907 0.6808 0.133 1.000

10 -0.9415 0.6808 -1.383 1.000

Time Point = 9 subtracted from:

Time Difference SE of Adjusted

Point of Means Difference T-Value P-Value

10 -1.032 0.6808 -1.516 1.000

Grouping Information Using Sidak Method and 95.0% Confidence

Time

Point N Mean Grouping

9 3 7.3222 A

8 3 7.2315 A

6 3 6.9822 A

7 3 6.9763 A

5 3 6.4919 A B

10 3 6.2900 A B

4 3 4.1259 B

3 2 -0.2061 C

Means that do not share a letter are significantly different.

Sidak 95.0% Simultaneous Confidence Intervals

Response Variable Cq Prime

All Pairwise Comparisons among Levels of Time Point

Time Point = 3 subtracted from:

Time

Point Lower Center Upper ---------+---------+---------+-------

4 1.322 4.332 7.342 (-------*------)

5 3.688 6.698 9.708 (-------*------)

6 4.178 7.188 10.198 (-------*------)

7 4.173 7.182 10.192 (-------*------)

8 4.428 7.438 10.447 (-------*------)

9 4.518 7.528 10.538 (-------*------)

10 3.486 6.496 9.506 (------*-------)

---------+---------+---------+-------

0.0 4.0 8.0

Time Point = 4 subtracted from:

Time

Point Lower Center Upper ---------+---------+---------+-------

5 -0.2885 2.366 5.020 (------*------)

6 0.2019 2.856 5.511 (-----*------)

7 0.1959 2.850 5.505 (------*------)

8 0.4511 3.106 5.760 (------*-----)

9 0.5419 3.196 5.851 (------*------)

10 -0.4904 2.164 4.819 (-----*------)

---------+---------+---------+-------

0.0 4.0 8.0

Time Point = 5 subtracted from:

Time

Point Lower Center Upper ---------+---------+---------+-------

6 -2.164 0.4904 3.145 (-----*------)

7 -2.170 0.4844 3.139 (-----*------)

8 -1.915 0.7396 3.394 (------*-----)

9 -1.824 0.8304 3.485 (------*------)

10 -2.856 -0.2019 2.453 (-----*------)

---------+---------+---------+-------

0.0 4.0 8.0

Time Point = 6 subtracted from:

Time

Point Lower Center Upper ---------+---------+---------+-------

7 -2.660 -0.0059 2.649 (------*------)

8 -2.405 0.2493 2.904 (------*-----)

9 -2.314 0.3400 2.994 (------*-----)

10 -3.347 -0.6922 1.962 (-----*------)

---------+---------+---------+-------

0.0 4.0 8.0

Time Point = 7 subtracted from:

Time

Point Lower Center Upper ---------+---------+---------+-------

8 -2.399 0.2552 2.910 (------*-----)

9 -2.309 0.3459 3.000 (------*------)

10 -3.341 -0.6863 1.968 (-----*------)

---------+---------+---------+-------

0.0 4.0 8.0

Time Point = 8 subtracted from:

Time

Point Lower Center Upper ---------+---------+---------+-------

9 -2.564 0.0907 2.745 (-----*------)

10 -3.596 -0.9415 1.713 (------*-----)

---------+---------+---------+-------

0.0 4.0 8.0

Time Point = 9 subtracted from:

Time

Point Lower Center Upper ---------+---------+---------+-------

10 -3.687 -1.032 1.622 (-----*------)

---------+---------+---------+-------

0.0 4.0 8.0

Sidak Simultaneous Tests

Response Variable Cq Prime

All Pairwise Comparisons among Levels of Time Point

Time Point = 3 subtracted from:

Time Difference SE of Adjusted

Point of Means Difference T-Value P-Value

4 4.332 0.7720 5.612 0.0024

5 6.698 0.7720 8.676 0.0000

6 7.188 0.7720 9.311 0.0000

7 7.182 0.7720 9.304 0.0000

8 7.438 0.7720 9.634 0.0000

9 7.528 0.7720 9.752 0.0000

10 6.496 0.7720 8.415 0.0000

Time Point = 4 subtracted from:

Time Difference SE of Adjusted

Point of Means Difference T-Value P-Value

5 2.366 0.6808 3.475 0.1088

6 2.856 0.6808 4.195 0.0290

7 2.850 0.6808 4.187 0.0294

8 3.106 0.6808 4.561 0.0148

9 3.196 0.6808 4.695 0.0117

10 2.164 0.6808 3.179 0.1846

Time Point = 5 subtracted from:

Time Difference SE of Adjusted

Point of Means Difference T-Value P-Value

6 0.4904 0.6808 0.7203 1.0000

7 0.4844 0.6808 0.7116 1.0000

8 0.7396 0.6808 1.0864 0.9999

9 0.8304 0.6808 1.2196 0.9996

10 -0.2019 0.6808 -0.2965 1.0000

Time Point = 6 subtracted from:

Time Difference SE of Adjusted

Point of Means Difference T-Value P-Value

7 -0.0059 0.6808 -0.009 1.000

8 0.2493 0.6808 0.366 1.000

9 0.3400 0.6808 0.499 1.000

10 -0.6922 0.6808 -1.017 1.000

Time Point = 7 subtracted from:

Time Difference SE of Adjusted

Point of Means Difference T-Value P-Value

8 0.2552 0.6808 0.375 1.000

9 0.3459 0.6808 0.508 1.000

10 -0.6863 0.6808 -1.008 1.000

Time Point = 8 subtracted from:

Time Difference SE of Adjusted

Point of Means Difference T-Value P-Value

9 0.0907 0.6808 0.133 1.0000

10 -0.9415 0.6808 -1.383 0.9973

Time Point = 9 subtracted from:

Time Difference SE of Adjusted

Point of Means Difference T-Value P-Value

10 -1.032 0.6808 -1.516 0.9906

Grouping Information Using Tukey Method and 95.0% Confidence

Time

Point N Mean Grouping

9 3 7.3222 A

8 3 7.2315 A

6 3 6.9822 A

7 3 6.9763 A

5 3 6.4919 A B

10 3 6.2900 A B

4 3 4.1259 B

3 2 -0.2061 C

Means that do not share a letter are significantly different.

Tukey 95.0% Simultaneous Confidence Intervals

Response Variable Cq Prime

All Pairwise Comparisons among Levels of Time Point

Time Point = 3 subtracted from:

Time

Point Lower Center Upper ---------+---------+---------+-------

4 1.575 4.332 7.089 (------*------)

5 3.941 6.698 9.455 (------*------)

6 4.432 7.188 9.945 (------*------)

7 4.426 7.182 9.939 (------*------)

8 4.681 7.438 10.194 (------*-----)

9 4.772 7.528 10.285 (------*------)

10 3.739 6.496 9.253 (------*------)

---------+---------+---------+-------

0.0 4.0 8.0

Time Point = 4 subtracted from:

Time

Point Lower Center Upper ---------+---------+---------+-------

5 -0.0652 2.366 4.797 (-----*-----)

6 0.4251 2.856 5.287 (-----*-----)

7 0.4192 2.850 5.282 (-----*-----)

8 0.6744 3.106 5.537 (-----*-----)

9 0.7651 3.196 5.627 (-----*-----)

10 -0.2671 2.164 4.595 (-----*-----)

---------+---------+---------+-------

0.0 4.0 8.0

Time Point = 5 subtracted from:

Time

Point Lower Center Upper ---------+---------+---------+-------

6 -1.941 0.4904 2.922 (-----*-----)

7 -1.947 0.4844 2.916 (-----*-----)

8 -1.692 0.7396 3.171 (-----*-----)

9 -1.601 0.8304 3.262 (-----*-----)

10 -2.633 -0.2019 2.229 (-----*------)

---------+---------+---------+-------

0.0 4.0 8.0

Time Point = 6 subtracted from:

Time

Point Lower Center Upper ---------+---------+---------+-------

7 -2.437 -0.0059 2.425 (-----*-----)

8 -2.182 0.2493 2.680 (-----*-----)

9 -2.091 0.3400 2.771 (-----*-----)

10 -3.123 -0.6922 1.739 (-----*-----)

---------+---------+---------+-------

0.0 4.0 8.0

Time Point = 7 subtracted from:

Time

Point Lower Center Upper ---------+---------+---------+-------

8 -2.176 0.2552 2.686 (-----*-----)

9 -2.085 0.3459 2.777 (-----*-----)

10 -3.117 -0.6863 1.745 (-----*-----)

---------+---------+---------+-------

0.0 4.0 8.0

Time Point = 8 subtracted from:

Time

Point Lower Center Upper ---------+---------+---------+-------

9 -2.340 0.0907 2.522 (-----*-----)

10 -3.373 -0.9415 1.490 (-----*-----)

---------+---------+---------+-------

0.0 4.0 8.0

Time Point = 9 subtracted from:

Time

Point Lower Center Upper ---------+---------+---------+-------

10 -3.463 -1.032 1.399 (-----*-----)

---------+---------+---------+-------

0.0 4.0 8.0

Tukey Simultaneous Tests

Response Variable Cq Prime

All Pairwise Comparisons among Levels of Time Point

Time Point = 3 subtracted from:

Time Difference SE of Adjusted

Point of Means Difference T-Value P-Value

4 4.332 0.7720 5.612 0.0016

5 6.698 0.7720 8.676 0.0000

6 7.188 0.7720 9.311 0.0000

7 7.182 0.7720 9.304 0.0000

8 7.438 0.7720 9.634 0.0000

9 7.528 0.7720 9.752 0.0000

10 6.496 0.7720 8.415 0.0000

Time Point = 4 subtracted from:

Time Difference SE of Adjusted

Point of Means Difference T-Value P-Value

5 2.366 0.6808 3.475 0.0588

6 2.856 0.6808 4.195 0.0171

7 2.850 0.6808 4.187 0.0173

8 3.106 0.6808 4.561 0.0091

9 3.196 0.6808 4.695 0.0072

10 2.164 0.6808 3.179 0.0965

Time Point = 5 subtracted from:

Time Difference SE of Adjusted

Point of Means Difference T-Value P-Value

6 0.4904 0.6808 0.7203 0.9946

7 0.4844 0.6808 0.7116 0.9950

8 0.7396 0.6808 1.0864 0.9489

9 0.8304 0.6808 1.2196 0.9120

10 -0.2019 0.6808 -0.2965 1.0000

Time Point = 6 subtracted from:

Time Difference SE of Adjusted

Point of Means Difference T-Value P-Value

7 -0.0059 0.6808 -0.009 1.0000

8 0.2493 0.6808 0.366 0.9999

9 0.3400 0.6808 0.499 0.9995

10 -0.6922 0.6808 -1.017 0.9633

Time Point = 7 subtracted from:

Time Difference SE of Adjusted

Point of Means Difference T-Value P-Value

8 0.2552 0.6808 0.375 0.9999

9 0.3459 0.6808 0.508 0.9994

10 -0.6863 0.6808 -1.008 0.9649

Time Point = 8 subtracted from:

Time Difference SE of Adjusted

Point of Means Difference T-Value P-Value

9 0.0907 0.6808 0.133 1.0000

10 -0.9415 0.6808 -1.383 0.8499

Time Point = 9 subtracted from:

Time Difference SE of Adjusted

Point of Means Difference T-Value P-Value

10 -1.032 0.6808 -1.516 0.7870

**Residual Plots for Cq Prime**

**Main Effects Plot for Cq Prime**

Psn0100:

**————— 9/11/2012 7:56:24 PM ————————————————————**

**General Linear Model: Cq Prime versus Biounit, Time Point**

Factor Type Levels Values

Biounit random 3 1, 2, 3

Time Point fixed 8 3, 4, 5, 6, 7, 8, 9, 10

Analysis of Variance for Cq Prime, using Adjusted SS for Tests

Source DF Seq SS Adj SS Adj MS F P

Biounit 2 10.3192 9.3303 4.6652 10.46 0.002

Time Point 7 7.9705 7.9705 1.1386 2.55 0.069

Error 13 5.7971 5.7971 0.4459

Total 22 24.0869

S = 0.667782 R-Sq = 75.93% R-Sq(adj) = 59.27%

Term Coef SE Coef T P

Constant -0.7687 0.1411 -5.45 0.000

Biounit

1 -0.9206 0.2061 -4.47 0.001

2 0.2962 0.1962 1.51 0.155

Time Point

3 0.3084 0.4417 0.70 0.497

4 1.0308 0.3625 2.84 0.014

5 -0.0600 0.3625 -0.17 0.871

6 -0.8285 0.3625 -2.29 0.040

7 -0.8811 0.3625 -2.43 0.030

8 0.0785 0.3625 0.22 0.832

9 0.1526 0.3625 0.42 0.681

Unusual Observations for Cq Prime

Obs Cq Prime Fit SE Fit Residual St Resid

13 -0.22944 -1.35359 0.43259 1.12415 2.21 R

R denotes an observation with a large standardized residual.

Expected Mean Squares, using Adjusted SS

Expected Mean Square

Source for Each Term

1 Biounit (3) + 7.5000 (1)

2 Time Point (3) + Q[2]

3 Error (3)

Error Terms for Tests, using Adjusted SS

Synthesis

Source Error DF Error MS of Error MS

1 Biounit 13.00 0.4459 (3)

2 Time Point 13.00 0.4459 (3)

Variance Components, using Adjusted SS

Estimated

Source Value

Biounit 0.5626

Error 0.4459

Grouping Information Using Bonferroni Method and 95.0% Confidence

Time

Point N Mean Grouping

4 3 0.262 A

3 2 -0.460 A

10 3 -0.569 A

9 3 -0.616 A

8 3 -0.690 A

5 3 -0.829 A

6 3 -1.597 A

7 3 -1.650 A

Means that do not share a letter are significantly different.

Bonferroni 95.0% Simultaneous Confidence Intervals

Response Variable Cq Prime

All Pairwise Comparisons among Levels of Time Point

Time Point = 3 subtracted from:

Time

Point Lower Center Upper +---------+---------+---------+------

4 -1.696 0.722 3.141 (-----------*-----------)

5 -2.787 -0.368 2.050 (-----------*-----------)

6 -3.555 -1.137 1.282 (-----------*-----------)

7 -3.608 -1.189 1.229 (-----------*-----------)

8 -2.648 -0.230 2.189 (-----------*-----------)

9 -2.574 -0.156 2.263 (-----------*-----------)

10 -2.528 -0.109 2.309 (-----------*------------)

+---------+---------+---------+------

-4.0 -2.0 0.0 2.0

Time Point = 4 subtracted from:

Time

Point Lower Center Upper +---------+---------+---------+------

5 -3.224 -1.091 1.0422 (----------*---------)

6 -3.992 -1.859 0.2736 (----------*---------)

7 -4.045 -1.912 0.2211 (---------*----------)

8 -3.085 -0.952 1.1807 (---------*----------)

9 -3.011 -0.878 1.2548 (----------*---------)

10 -2.964 -0.831 1.3014 (----------*----------)

+---------+---------+---------+------

-4.0 -2.0 0.0 2.0

Time Point = 5 subtracted from:

Time

Point Lower Center Upper +---------+---------+---------+------

6 -2.901 -0.7685 1.364 (----------*----------)

7 -2.954 -0.8211 1.312 (----------*----------)

8 -1.994 0.1385 2.271 (----------*---------)

9 -1.920 0.2126 2.345 (----------*----------)

10 -1.874 0.2593 2.392 (---------*----------)

+---------+---------+---------+------

-4.0 -2.0 0.0 2.0

Time Point = 6 subtracted from:

Time

Point Lower Center Upper +---------+---------+---------+------

7 -2.185 -0.05259 2.080 (----------*---------)

8 -1.226 0.90704 3.040 (----------*---------)

9 -1.152 0.98111 3.114 (----------*----------)

10 -1.105 1.02778 3.161 (----------*----------)

+---------+---------+---------+------

-4.0 -2.0 0.0 2.0

Time Point = 7 subtracted from:

Time

Point Lower Center Upper +---------+---------+---------+------

8 -1.173 0.9596 3.093 (----------*---------)

9 -1.099 1.0337 3.167 (---------*----------)

10 -1.053 1.0804 3.213 (---------*----------)

+---------+---------+---------+------

-4.0 -2.0 0.0 2.0

Time Point = 8 subtracted from:

Time

Point Lower Center Upper +---------+---------+---------+------

9 -2.059 0.07407 2.207 (---------*----------)

10 -2.012 0.12074 2.254 (----------*---------)

+---------+---------+---------+------

-4.0 -2.0 0.0 2.0

Time Point = 9 subtracted from:

Time

Point Lower Center Upper +---------+---------+---------+------

10 -2.086 0.04667 2.180 (---------*----------)

+---------+---------+---------+------

-4.0 -2.0 0.0 2.0

Bonferroni Simultaneous Tests

Response Variable Cq Prime

All Pairwise Comparisons among Levels of Time Point

Time Point = 3 subtracted from:

Time Difference SE of Adjusted

Point of Means Difference T-Value P-Value

4 0.722 0.6182 1.168 1.000

5 -0.368 0.6182 -0.596 1.000

6 -1.137 0.6182 -1.839 1.000

7 -1.189 0.6182 -1.924 1.000

8 -0.230 0.6182 -0.372 1.000

9 -0.156 0.6182 -0.252 1.000

10 -0.109 0.6182 -0.177 1.000

Time Point = 4 subtracted from:

Time Difference SE of Adjusted

Point of Means Difference T-Value P-Value

5 -1.091 0.5452 -2.000 1.0000

6 -1.859 0.5452 -3.410 0.1303

7 -1.912 0.5452 -3.506 0.1083

8 -0.952 0.5452 -1.746 1.0000

9 -0.878 0.5452 -1.611 1.0000

10 -0.831 0.5452 -1.525 1.0000

Time Point = 5 subtracted from:

Time Difference SE of Adjusted

Point of Means Difference T-Value P-Value

6 -0.7685 0.5452 -1.410 1.000

7 -0.8211 0.5452 -1.506 1.000

8 0.1385 0.5452 0.254 1.000

9 0.2126 0.5452 0.390 1.000

10 0.2593 0.5452 0.475 1.000

Time Point = 6 subtracted from:

Time Difference SE of Adjusted

Point of Means Difference T-Value P-Value

7 -0.05259 0.5452 -0.09646 1.000

8 0.90704 0.5452 1.66355 1.000

9 0.98111 0.5452 1.79941 1.000

10 1.02778 0.5452 1.88499 1.000

Time Point = 7 subtracted from:

Time Difference SE of Adjusted

Point of Means Difference T-Value P-Value

8 0.9596 0.5452 1.760 1.000

9 1.0337 0.5452 1.896 1.000

10 1.0804 0.5452 1.981 1.000

Time Point = 8 subtracted from:

Time Difference SE of Adjusted

Point of Means Difference T-Value P-Value

9 0.07407 0.5452 0.1359 1.000

10 0.12074 0.5452 0.2214 1.000

Time Point = 9 subtracted from:

Time Difference SE of Adjusted

Point of Means Difference T-Value P-Value

10 0.04667 0.5452 0.08559 1.000

Grouping Information Using Sidak Method and 95.0% Confidence

Time

Point N Mean Grouping

4 3 0.262 A

3 2 -0.460 A

10 3 -0.569 A

9 3 -0.616 A

8 3 -0.690 A

5 3 -0.829 A

6 3 -1.597 A

7 3 -1.650 A

Means that do not share a letter are significantly different.

Sidak 95.0% Simultaneous Confidence Intervals

Response Variable Cq Prime

All Pairwise Comparisons among Levels of Time Point

Time Point = 3 subtracted from:

Time

Point Lower Center Upper +---------+---------+---------+------

4 -1.688 0.722 3.133 (-----------*-----------)

5 -2.779 -0.368 2.042 (-----------*-----------)

6 -3.547 -1.137 1.274 (-----------*-----------)

7 -3.600 -1.189 1.221 (-----------*-----------)

8 -2.640 -0.230 2.181 (-----------*-----------)

9 -2.566 -0.156 2.255 (-----------*-----------)

10 -2.520 -0.109 2.301 (-----------*------------)

+---------+---------+---------+------

-4.0 -2.0 0.0 2.0

Time Point = 4 subtracted from:

Time

Point Lower Center Upper +---------+---------+---------+------

5 -3.217 -1.091 1.0351 (----------*---------)

6 -3.985 -1.859 0.2665 (----------*---------)

7 -4.038 -1.912 0.2140 (---------*----------)

8 -3.078 -0.952 1.1736 (---------*----------)

9 -3.004 -0.878 1.2477 (----------*---------)

10 -2.957 -0.831 1.2943 (----------*---------)

+---------+---------+---------+------

-4.0 -2.0 0.0 2.0

Time Point = 5 subtracted from:

Time

Point Lower Center Upper +---------+---------+---------+------

6 -2.894 -0.7685 1.357 (---------*----------)

7 -2.947 -0.8211 1.305 (----------*----------)

8 -1.987 0.1385 2.264 (----------*---------)

9 -1.913 0.2126 2.338 (----------*----------)

10 -1.867 0.2593 2.385 (---------*----------)

+---------+---------+---------+------

-4.0 -2.0 0.0 2.0

Time Point = 6 subtracted from:

Time

Point Lower Center Upper +---------+---------+---------+------

7 -2.178 -0.05259 2.073 (----------*---------)

8 -1.219 0.90704 3.033 (----------*---------)

9 -1.145 0.98111 3.107 (----------*----------)

10 -1.098 1.02778 3.154 (---------*----------)

+---------+---------+---------+------

-4.0 -2.0 0.0 2.0

Time Point = 7 subtracted from:

Time

Point Lower Center Upper +---------+---------+---------+------

8 -1.166 0.9596 3.085 (----------*---------)

9 -1.092 1.0337 3.160 (---------*----------)

10 -1.045 1.0804 3.206 (---------*----------)

+---------+---------+---------+------

-4.0 -2.0 0.0 2.0

Time Point = 8 subtracted from:

Time

Point Lower Center Upper +---------+---------+---------+------

9 -2.052 0.07407 2.200 (---------*----------)

10 -2.005 0.12074 2.247 (----------*---------)

+---------+---------+---------+------

-4.0 -2.0 0.0 2.0

Time Point = 9 subtracted from:

Time

Point Lower Center Upper +---------+---------+---------+------

10 -2.079 0.04667 2.172 (---------*----------)

+---------+---------+---------+------

-4.0 -2.0 0.0 2.0

Sidak Simultaneous Tests

Response Variable Cq Prime

All Pairwise Comparisons among Levels of Time Point

Time Point = 3 subtracted from:

Time Difference SE of Adjusted

Point of Means Difference T-Value P-Value

4 0.722 0.6182 1.168 0.9998

5 -0.368 0.6182 -0.596 1.0000

6 -1.137 0.6182 -1.839 0.9262

7 -1.189 0.6182 -1.924 0.8924

8 -0.230 0.6182 -0.372 1.0000

9 -0.156 0.6182 -0.252 1.0000

10 -0.109 0.6182 -0.177 1.0000

Time Point = 4 subtracted from:

Time Difference SE of Adjusted

Point of Means Difference T-Value P-Value

5 -1.091 0.5452 -2.000 0.8556

6 -1.859 0.5452 -3.410 0.1224

7 -1.912 0.5452 -3.506 0.1028

8 -0.952 0.5452 -1.746 0.9542

9 -0.878 0.5452 -1.611 0.9806

10 -0.831 0.5452 -1.525 0.9899

Time Point = 5 subtracted from:

Time Difference SE of Adjusted

Point of Means Difference T-Value P-Value

6 -0.7685 0.5452 -1.410 0.9964

7 -0.8211 0.5452 -1.506 0.9913

8 0.1385 0.5452 0.254 1.0000

9 0.2126 0.5452 0.390 1.0000

10 0.2593 0.5452 0.475 1.0000

Time Point = 6 subtracted from:

Time Difference SE of Adjusted

Point of Means Difference T-Value P-Value

7 -0.05259 0.5452 -0.09646 1.0000

8 0.90704 0.5452 1.66355 0.9722

9 0.98111 0.5452 1.79941 0.9393

10 1.02778 0.5452 1.88499 0.9088

Time Point = 7 subtracted from:

Time Difference SE of Adjusted

Point of Means Difference T-Value P-Value

8 0.9596 0.5452 1.760 0.9507

9 1.0337 0.5452 1.896 0.9044

10 1.0804 0.5452 1.981 0.8653

Time Point = 8 subtracted from:

Time Difference SE of Adjusted

Point of Means Difference T-Value P-Value

9 0.07407 0.5452 0.1359 1.000

10 0.12074 0.5452 0.2214 1.000

Time Point = 9 subtracted from:

Time Difference SE of Adjusted

Point of Means Difference T-Value P-Value

10 0.04667 0.5452 0.08559 1.000

Grouping Information Using Tukey Method and 95.0% Confidence

Time

Point N Mean Grouping

4 3 0.262 A

3 2 -0.460 A

10 3 -0.569 A

9 3 -0.616 A

8 3 -0.690 A

5 3 -0.829 A

6 3 -1.597 A

7 3 -1.650 A

Means that do not share a letter are significantly different.

Tukey 95.0% Simultaneous Confidence Intervals

Response Variable Cq Prime

All Pairwise Comparisons among Levels of Time Point

Time Point = 3 subtracted from:

Time

Point Lower Center Upper ---------+---------+---------+-------

4 -1.485 0.722 2.930 (----------*----------)

5 -2.576 -0.368 1.839 (----------*----------)

6 -3.345 -1.137 1.071 (----------*----------)

7 -3.397 -1.189 1.018 (----------*----------)

8 -2.438 -0.230 1.978 (----------*----------)

9 -2.363 -0.156 2.052 (----------*----------)

10 -2.317 -0.109 2.099 (----------*----------)

---------+---------+---------+-------

-2.0 0.0 2.0

Time Point = 4 subtracted from:

Time

Point Lower Center Upper ---------+---------+---------+-------

5 -3.038 -1.091 0.85626 (---------*--------)

6 -3.806 -1.859 0.08774 (---------*--------)

7 -3.859 -1.912 0.03515 (--------*---------)

8 -2.899 -0.952 0.99478 (--------*---------)

9 -2.825 -0.878 1.06885 (---------*--------)

10 -2.778 -0.831 1.11552 (---------*---------)

---------+---------+---------+-------

-2.0 0.0 2.0

Time Point = 5 subtracted from:

Time

Point Lower Center Upper ---------+---------+---------+-------

6 -2.716 -0.7685 1.178 (---------*---------)

7 -2.768 -0.8211 1.126 (---------*---------)

8 -1.808 0.1385 2.086 (---------*--------)

9 -1.734 0.2126 2.160 (---------*---------)

10 -1.688 0.2593 2.206 (--------*---------)

---------+---------+---------+-------

-2.0 0.0 2.0

Time Point = 6 subtracted from:

Time

Point Lower Center Upper ---------+---------+---------+-------

7 -2.000 -0.05259 1.894 (---------*--------)

8 -1.040 0.90704 2.854 (---------*--------)

9 -0.966 0.98111 2.928 (---------*---------)

10 -0.919 1.02778 2.975 (---------*---------)

---------+---------+---------+-------

-2.0 0.0 2.0

Time Point = 7 subtracted from:

Time

Point Lower Center Upper ---------+---------+---------+-------

8 -0.9874 0.9596 2.907 (---------*---------)

9 -0.9133 1.0337 2.981 (---------*---------)

10 -0.8666 1.0804 3.027 (--------*---------)

---------+---------+---------+-------

-2.0 0.0 2.0

Time Point = 8 subtracted from:

Time

Point Lower Center Upper ---------+---------+---------+-------

9 -1.873 0.07407 2.021 (--------*---------)

10 -1.826 0.12074 2.068 (---------*--------)

---------+---------+---------+-------

-2.0 0.0 2.0

Time Point = 9 subtracted from:

Time

Point Lower Center Upper ---------+---------+---------+-------

10 -1.900 0.04667 1.994 (---------*---------)

---------+---------+---------+-------

-2.0 0.0 2.0

Tukey Simultaneous Tests

Response Variable Cq Prime

All Pairwise Comparisons among Levels of Time Point

Time Point = 3 subtracted from:

Time Difference SE of Adjusted

Point of Means Difference T-Value P-Value

4 0.722 0.6182 1.168 0.9277

5 -0.368 0.6182 -0.596 0.9983

6 -1.137 0.6182 -1.839 0.6082

7 -1.189 0.6182 -1.924 0.5592

8 -0.230 0.6182 -0.372 0.9999

9 -0.156 0.6182 -0.252 1.0000

10 -0.109 0.6182 -0.177 1.0000

Time Point = 4 subtracted from:

Time Difference SE of Adjusted

Point of Means Difference T-Value P-Value

5 -1.091 0.5452 -2.000 0.5159

6 -1.859 0.5452 -3.410 0.0656

7 -1.912 0.5452 -3.506 0.0557

8 -0.952 0.5452 -1.746 0.6614

9 -0.878 0.5452 -1.611 0.7374

10 -0.831 0.5452 -1.525 0.7825

Time Point = 5 subtracted from:

Time Difference SE of Adjusted

Point of Means Difference T-Value P-Value

6 -0.7685 0.5452 -1.410 0.8381

7 -0.8211 0.5452 -1.506 0.7921

8 0.1385 0.5452 0.254 1.0000

9 0.2126 0.5452 0.390 0.9999

10 0.2593 0.5452 0.475 0.9996

Time Point = 6 subtracted from:

Time Difference SE of Adjusted

Point of Means Difference T-Value P-Value

7 -0.05259 0.5452 -0.09646 1.0000

8 0.90704 0.5452 1.66355 0.7083

9 0.98111 0.5452 1.79941 0.6310

10 1.02778 0.5452 1.88499 0.5816

Time Point = 7 subtracted from:

Time Difference SE of Adjusted

Point of Means Difference T-Value P-Value

8 0.9596 0.5452 1.760 0.6536

9 1.0337 0.5452 1.896 0.5754

10 1.0804 0.5452 1.981 0.5266

Time Point = 8 subtracted from:

Time Difference SE of Adjusted

Point of Means Difference T-Value P-Value

9 0.07407 0.5452 0.1359 1.000

10 0.12074 0.5452 0.2214 1.000

Time Point = 9 subtracted from:

Time Difference SE of Adjusted

Point of Means Difference T-Value P-Value

10 0.04667 0.5452 0.08559 1.000

**Residual Plots for Cq Prime**

**Main Effects Plot for Cq Prime**

PSN0100 EX:

**————— 9/11/2012 7:56:24 PM ————————————————————**

**General Linear Model: Cq Prime versus Biounit, Time Point**

Factor Type Levels Values

Biounit random 3 1, 2, 3

Time Point fixed 8 3, 4, 5, 6, 7, 8, 9, 10

Analysis of Variance for Cq Prime, using Adjusted SS for Tests

Source DF Seq SS Adj SS Adj MS F P

Biounit 2 11.4523 10.2790 5.1395 9.49 0.003

Time Point 7 9.9033 9.9033 1.4148 2.61 0.064

Error 13 7.0402 7.0402 0.5416

Total 22 28.3958

S = 0.735903 R-Sq = 75.21% R-Sq(adj) = 58.04%

Term Coef SE Coef T P

Constant -0.8462 0.1555 -5.44 0.000

Biounit

1 -0.9694 0.2271 -4.27 0.001

2 0.3241 0.2162 1.50 0.158

Time Point

3 0.3615 0.4868 0.74 0.471

4 1.1538 0.3995 2.89 0.013

5 -0.0903 0.3995 -0.23 0.825

6 -0.8010 0.3995 -2.01 0.066

7 -1.0814 0.3995 -2.71 0.018

8 0.1460 0.3995 0.37 0.721

9 0.1545 0.3995 0.39 0.705

Unusual Observations for Cq Prime

Obs Cq Prime Fit SE Fit Residual St Resid

13 -0.35278 -1.60347 0.47672 1.25069 2.23 R

R denotes an observation with a large standardized residual.

Expected Mean Squares, using Adjusted SS

Expected Mean Square

Source for Each Term

1 Biounit (3) + 7.5000 (1)

2 Time Point (3) + Q[2]

3 Error (3)

Error Terms for Tests, using Adjusted SS

Synthesis

Source Error DF Error MS of Error MS

1 Biounit 13.00 0.5416 (3)

2 Time Point 13.00 0.5416 (3)

Variance Components, using Adjusted SS

Estimated

Source Value

Biounit 0.6131

Error 0.5416

Grouping Information Using Bonferroni Method and 95.0% Confidence

Time

Point N Mean Grouping

4 3 0.308 A

3 2 -0.485 A

10 3 -0.689 A

9 3 -0.692 A

8 3 -0.700 A

5 3 -0.936 A

6 3 -1.647 A

7 3 -1.928 A

Means that do not share a letter are significantly different.

Bonferroni 95.0% Simultaneous Confidence Intervals

Response Variable Cq Prime

All Pairwise Comparisons among Levels of Time Point

Time Point = 3 subtracted from:

Time

Point Lower Center Upper --------+---------+---------+--------

4 -1.873 0.792 3.457 (---------*----------)

5 -3.117 -0.452 2.213 (---------*----------)

6 -3.828 -1.163 1.503 (---------*----------)

7 -4.108 -1.443 1.222 (---------*----------)

8 -2.881 -0.216 2.450 (----------*----------)

9 -2.872 -0.207 2.458 (---------*----------)

10 -2.870 -0.205 2.460 (---------*----------)

--------+---------+---------+--------

-2.5 0.0 2.5

Time Point = 4 subtracted from:

Time

Point Lower Center Upper --------+---------+---------+--------

5 -3.595 -1.244 1.1064 (--------*--------)

6 -4.305 -1.955 0.3957 (--------*---------)

7 -4.586 -2.235 0.1153 (--------*--------)

8 -3.358 -1.008 1.3427 (--------*--------)

9 -3.350 -0.999 1.3512 (--------*--------)

10 -3.348 -0.997 1.3534 (--------*--------)

--------+---------+---------+--------

-2.5 0.0 2.5

Time Point = 5 subtracted from:

Time

Point Lower Center Upper --------+---------+---------+--------

6 -3.061 -0.7107 1.640 (--------*---------)

7 -3.342 -0.9911 1.359 (--------*--------)

8 -2.114 0.2363 2.587 (--------*--------)

9 -2.106 0.2448 2.595 (--------*--------)

10 -2.103 0.2470 2.598 (--------*--------)

--------+---------+---------+--------

-2.5 0.0 2.5

Time Point = 6 subtracted from:

Time

Point Lower Center Upper --------+---------+---------+--------

7 -2.631 -0.2804 2.070 (---------*--------)

8 -1.403 0.9470 3.298 (---------*--------)

9 -1.395 0.9556 3.306 (---------*--------)

10 -1.393 0.9578 3.308 (---------*--------)

--------+---------+---------+--------

-2.5 0.0 2.5

Time Point = 7 subtracted from:

Time

Point Lower Center Upper --------+---------+---------+--------

8 -1.123 1.227 3.578 (--------*--------)

9 -1.115 1.236 3.586 (--------*--------)

10 -1.112 1.238 3.589 (--------*--------)

--------+---------+---------+--------

-2.5 0.0 2.5

Time Point = 8 subtracted from:

Time

Point Lower Center Upper --------+---------+---------+--------

9 -2.342 0.008519 2.359 (--------*--------)

10 -2.340 0.010741 2.361 (--------*--------)

--------+---------+---------+--------

-2.5 0.0 2.5

Time Point = 9 subtracted from:

Time

Point Lower Center Upper --------+---------+---------+--------

10 -2.348 0.002222 2.353 (--------*--------)

--------+---------+---------+--------

-2.5 0.0 2.5

Bonferroni Simultaneous Tests

Response Variable Cq Prime

All Pairwise Comparisons among Levels of Time Point

Time Point = 3 subtracted from:

Time Difference SE of Adjusted

Point of Means Difference T-Value P-Value

4 0.792 0.6813 1.163 1.000

5 -0.452 0.6813 -0.663 1.000

6 -1.163 0.6813 -1.706 1.000

7 -1.443 0.6813 -2.118 1.000

8 -0.216 0.6813 -0.316 1.000

9 -0.207 0.6813 -0.304 1.000

10 -0.205 0.6813 -0.301 1.000

Time Point = 4 subtracted from:

Time Difference SE of Adjusted

Point of Means Difference T-Value P-Value

5 -1.244 0.6009 -2.070 1.0000

6 -1.955 0.6009 -3.253 0.1761

7 -2.235 0.6009 -3.720 0.0720

8 -1.008 0.6009 -1.677 1.0000

9 -0.999 0.6009 -1.663 1.0000

10 -0.997 0.6009 -1.659 1.0000

Time Point = 5 subtracted from:

Time Difference SE of Adjusted

Point of Means Difference T-Value P-Value

6 -0.7107 0.6009 -1.183 1.000

7 -0.9911 0.6009 -1.649 1.000

8 0.2363 0.6009 0.393 1.000

9 0.2448 0.6009 0.407 1.000

10 0.2470 0.6009 0.411 1.000

Time Point = 6 subtracted from:

Time Difference SE of Adjusted

Point of Means Difference T-Value P-Value

7 -0.2804 0.6009 -0.4666 1.000

8 0.9470 0.6009 1.5761 1.000

9 0.9556 0.6009 1.5903 1.000

10 0.9578 0.6009 1.5940 1.000

Time Point = 7 subtracted from:

Time Difference SE of Adjusted

Point of Means Difference T-Value P-Value

8 1.227 0.6009 2.043 1.000

9 1.236 0.6009 2.057 1.000

10 1.238 0.6009 2.061 1.000

Time Point = 8 subtracted from:

Time Difference SE of Adjusted

Point of Means Difference T-Value P-Value

9 0.008519 0.6009 0.01418 1.000

10 0.010741 0.6009 0.01788 1.000

Time Point = 9 subtracted from:

Time Difference SE of Adjusted

Point of Means Difference T-Value P-Value

10 0.002222 0.6009 0.003698 1.000

Grouping Information Using Sidak Method and 95.0% Confidence

Time

Point N Mean Grouping

4 3 0.308 A

3 2 -0.485 A

10 3 -0.689 A

9 3 -0.692 A

8 3 -0.700 A

5 3 -0.936 A

6 3 -1.647 A

7 3 -1.928 A

Means that do not share a letter are significantly different.

Sidak 95.0% Simultaneous Confidence Intervals

Response Variable Cq Prime

All Pairwise Comparisons among Levels of Time Point

Time Point = 3 subtracted from:

Time

Point Lower Center Upper --------+---------+---------+--------

4 -1.864 0.792 3.449 (---------*----------)

5 -3.108 -0.452 2.205 (---------*----------)

6 -3.819 -1.163 1.494 (---------*----------)

7 -4.099 -1.443 1.213 (---------*----------)

8 -2.872 -0.216 2.441 (---------*----------)

9 -2.863 -0.207 2.449 (---------*----------)

10 -2.861 -0.205 2.452 (---------*----------)

--------+---------+---------+--------

-2.5 0.0 2.5

Time Point = 4 subtracted from:

Time

Point Lower Center Upper --------+---------+---------+--------

5 -3.587 -1.244 1.0986 (--------*--------)

6 -4.297 -1.955 0.3878 (--------*---------)

7 -4.578 -2.235 0.1075 (--------*--------)

8 -3.350 -1.008 1.3349 (--------*--------)

9 -3.342 -0.999 1.3434 (--------*--------)

10 -3.340 -0.997 1.3456 (--------*--------)

--------+---------+---------+--------

-2.5 0.0 2.5

Time Point = 5 subtracted from:

Time

Point Lower Center Upper --------+---------+---------+--------

6 -3.053 -0.7107 1.632 (--------*---------)

7 -3.334 -0.9911 1.352 (--------*--------)

8 -2.106 0.2363 2.579 (--------*--------)

9 -2.098 0.2448 2.587 (--------*--------)

10 -2.096 0.2470 2.590 (--------*--------)

--------+---------+---------+--------

-2.5 0.0 2.5

Time Point = 6 subtracted from:

Time

Point Lower Center Upper --------+---------+---------+--------

7 -2.623 -0.2804 2.062 (--------*--------)

8 -1.396 0.9470 3.290 (---------*--------)

9 -1.387 0.9556 3.298 (---------*--------)

10 -1.385 0.9578 3.300 (---------*--------)

--------+---------+---------+--------

-2.5 0.0 2.5

Time Point = 7 subtracted from:

Time

Point Lower Center Upper --------+---------+---------+--------

8 -1.115 1.227 3.570 (--------*--------)

9 -1.107 1.236 3.579 (--------*--------)

10 -1.105 1.238 3.581 (--------*--------)

--------+---------+---------+--------

-2.5 0.0 2.5

Time Point = 8 subtracted from:

Time

Point Lower Center Upper --------+---------+---------+--------

9 -2.334 0.008519 2.351 (--------*--------)

10 -2.332 0.010741 2.353 (--------*--------)

--------+---------+---------+--------

-2.5 0.0 2.5

Time Point = 9 subtracted from:

Time

Point Lower Center Upper --------+---------+---------+--------

10 -2.340 0.002222 2.345 (--------*--------)

--------+---------+---------+--------

-2.5 0.0 2.5

Sidak Simultaneous Tests

Response Variable Cq Prime

All Pairwise Comparisons among Levels of Time Point

Time Point = 3 subtracted from:

Time Difference SE of Adjusted

Point of Means Difference T-Value P-Value

4 0.792 0.6813 1.163 0.9998

5 -0.452 0.6813 -0.663 1.0000

6 -1.163 0.6813 -1.706 0.9637

7 -1.443 0.6813 -2.118 0.7889

8 -0.216 0.6813 -0.316 1.0000

9 -0.207 0.6813 -0.304 1.0000

10 -0.205 0.6813 -0.301 1.0000

Time Point = 4 subtracted from:

Time Difference SE of Adjusted

Point of Means Difference T-Value P-Value

5 -1.244 0.6009 -2.070 0.8172

6 -1.955 0.6009 -3.253 0.1619

7 -2.235 0.6009 -3.720 0.0695

8 -1.008 0.6009 -1.677 0.9697

9 -0.999 0.6009 -1.663 0.9723

10 -0.997 0.6009 -1.659 0.9729

Time Point = 5 subtracted from:

Time Difference SE of Adjusted

Point of Means Difference T-Value P-Value

6 -0.7107 0.6009 -1.183 0.9998

7 -0.9911 0.6009 -1.649 0.9746

8 0.2363 0.6009 0.393 1.0000

9 0.2448 0.6009 0.407 1.0000

10 0.2470 0.6009 0.411 1.0000

Time Point = 6 subtracted from:

Time Difference SE of Adjusted

Point of Means Difference T-Value P-Value

7 -0.2804 0.6009 -0.4666 1.0000

8 0.9470 0.6009 1.5761 0.9849

9 0.9556 0.6009 1.5903 0.9832

10 0.9578 0.6009 1.5940 0.9827

Time Point = 7 subtracted from:

Time Difference SE of Adjusted

Point of Means Difference T-Value P-Value

8 1.227 0.6009 2.043 0.8329

9 1.236 0.6009 2.057 0.8249

10 1.238 0.6009 2.061 0.8228

Time Point = 8 subtracted from:

Time Difference SE of Adjusted

Point of Means Difference T-Value P-Value

9 0.008519 0.6009 0.01418 1.000

10 0.010741 0.6009 0.01788 1.000

Time Point = 9 subtracted from:

Time Difference SE of Adjusted

Point of Means Difference T-Value P-Value

10 0.002222 0.6009 0.003698 1.000

Grouping Information Using Tukey Method and 95.0% Confidence

Time

Point N Mean Grouping

4 3 0.308 A

3 2 -0.485 A B

10 3 -0.689 A B

9 3 -0.692 A B

8 3 -0.700 A B

5 3 -0.936 A B

6 3 -1.647 A B

7 3 -1.928 B

Means that do not share a letter are significantly different.

Tukey 95.0% Simultaneous Confidence Intervals

Response Variable Cq Prime

All Pairwise Comparisons among Levels of Time Point

Time Point = 3 subtracted from:

Time

Point Lower Center Upper --------+---------+---------+--------

4 -1.641 0.792 3.2252 (---------*---------)

5 -2.885 -0.452 1.9811 (---------*---------)

6 -3.595 -1.163 1.2704 (--------*---------)

7 -3.876 -1.443 0.9900 (---------*---------)

8 -2.648 -0.216 2.2174 (---------*---------)

9 -2.640 -0.207 2.2259 (---------*---------)

10 -2.638 -0.205 2.2281 (---------*---------)

--------+---------+---------+--------

-2.5 0.0 2.5

Time Point = 4 subtracted from:

Time

Point Lower Center Upper --------+---------+---------+--------

5 -3.390 -1.244 0.90154 (--------*--------)

6 -4.100 -1.955 0.19080 (-------*--------)

7 -4.381 -2.235 -0.08957 (--------*--------)

8 -3.153 -1.008 1.13783 (--------*--------)

9 -3.145 -0.999 1.14635 (--------*--------)

10 -3.143 -0.997 1.14858 (--------*--------)

--------+---------+---------+--------

-2.5 0.0 2.5

Time Point = 5 subtracted from:

Time

Point Lower Center Upper --------+---------+---------+--------

6 -2.856 -0.7107 1.435 (-------*--------)

7 -3.137 -0.9911 1.155 (--------*--------)

8 -1.909 0.2363 2.382 (--------*--------)

9 -1.901 0.2448 2.390 (--------*--------)

10 -1.899 0.2470 2.393 (--------*--------)

--------+---------+---------+--------

-2.5 0.0 2.5

Time Point = 6 subtracted from:

Time

Point Lower Center Upper --------+---------+---------+--------

7 -2.426 -0.2804 1.865 (--------*-------)

8 -1.199 0.9470 3.093 (--------*-------)

9 -1.190 0.9556 3.101 (--------*-------)

10 -1.188 0.9578 3.103 (--------*-------)

--------+---------+---------+--------

-2.5 0.0 2.5

Time Point = 7 subtracted from:

Time

Point Lower Center Upper --------+---------+---------+--------

8 -0.9182 1.227 3.373 (--------*-------)

9 -0.9097 1.236 3.382 (--------*--------)

10 -0.9075 1.238 3.384 (--------*--------)

--------+---------+---------+--------

-2.5 0.0 2.5

Time Point = 8 subtracted from:

Time

Point Lower Center Upper --------+---------+---------+--------

9 -2.137 0.008519 2.154 (--------*--------)

10 -2.135 0.010741 2.156 (--------*--------)

--------+---------+---------+--------

-2.5 0.0 2.5

Time Point = 9 subtracted from:

Time

Point Lower Center Upper --------+---------+---------+--------

10 -2.143 0.002222 2.148 (--------*--------)

--------+---------+---------+--------

-2.5 0.0 2.5

Tukey Simultaneous Tests

Response Variable Cq Prime

All Pairwise Comparisons among Levels of Time Point

Time Point = 3 subtracted from:

Time Difference SE of Adjusted

Point of Means Difference T-Value P-Value

4 0.792 0.6813 1.163 0.9292

5 -0.452 0.6813 -0.663 0.9967

6 -1.163 0.6813 -1.706 0.6843

7 -1.443 0.6813 -2.118 0.4518

8 -0.216 0.6813 -0.316 1.0000

9 -0.207 0.6813 -0.304 1.0000

10 -0.205 0.6813 -0.301 1.0000

Time Point = 4 subtracted from:

Time Difference SE of Adjusted

Point of Means Difference T-Value P-Value

5 -1.244 0.6009 -2.070 0.4773

6 -1.955 0.6009 -3.253 0.0853

7 -2.235 0.6009 -3.720 0.0387

8 -1.008 0.6009 -1.677 0.7007

9 -0.999 0.6009 -1.663 0.7086

10 -0.997 0.6009 -1.659 0.7106

Time Point = 5 subtracted from:

Time Difference SE of Adjusted

Point of Means Difference T-Value P-Value

6 -0.7107 0.6009 -1.183 0.9234

7 -0.9911 0.6009 -1.649 0.7161

8 0.2363 0.6009 0.393 0.9999

9 0.2448 0.6009 0.407 0.9999

10 0.2470 0.6009 0.411 0.9998

Time Point = 6 subtracted from:

Time Difference SE of Adjusted

Point of Means Difference T-Value P-Value

7 -0.2804 0.6009 -0.4666 0.9996

8 0.9470 0.6009 1.5761 0.7559

9 0.9556 0.6009 1.5903 0.7484

10 0.9578 0.6009 1.5940 0.7464

Time Point = 7 subtracted from:

Time Difference SE of Adjusted

Point of Means Difference T-Value P-Value

8 1.227 0.6009 2.043 0.4924

9 1.236 0.6009 2.057 0.4847

10 1.238 0.6009 2.061 0.4826

Time Point = 8 subtracted from:

Time Difference SE of Adjusted

Point of Means Difference T-Value P-Value

9 0.008519 0.6009 0.01418 1.000

10 0.010741 0.6009 0.01788 1.000

Time Point = 9 subtracted from:

Time Difference SE of Adjusted

Point of Means Difference T-Value P-Value

10 0.002222 0.6009 0.003698 1.000

**Residual Plots for Cq Prime**

**Main Effects Plot for Cq Prime**

Psn0918:

**————— 9/11/2012 7:56:24 PM ————————————————————**

**General Linear Model: Cq Prime versus Biounit, Time Point**

Factor Type Levels Values

Biounit random 3 1, 2, 3

Time Point fixed 8 3, 4, 5, 6, 7, 8, 9, 10

Analysis of Variance for Cq Prime, using Adjusted SS for Tests

Source DF Seq SS Adj SS Adj MS F P

Biounit 2 0.0432 0.0281 0.0140 0.12 0.891

Time Point 7 1.4983 1.4983 0.2140 1.78 0.175

Error 13 1.5615 1.5615 0.1201

Total 22 3.1030

S = 0.346572 R-Sq = 49.68% R-Sq(adj) = 14.84%

Term Coef SE Coef T P

Constant 0.48606 0.07323 6.64 0.000

Biounit

1 -0.0086 0.1070 -0.08 0.937

2 0.0456 0.1018 0.45 0.662

Time Point

3 -0.4903 0.2292 -2.14 0.052

4 0.5404 0.1881 2.87 0.013

5 0.1419 0.1881 0.75 0.464

6 -0.1200 0.1881 -0.64 0.535

7 -0.0637 0.1881 -0.34 0.740

8 -0.0340 0.1881 -0.18 0.859

9 -0.0833 0.1881 -0.44 0.665

Unusual Observations for Cq Prime

Obs Cq Prime Fit SE Fit Residual St Resid

3 0.32389 1.01791 0.22688 -0.69402 -2.65 R

4 1.73167 1.07209 0.22451 0.65958 2.50 R

R denotes an observation with a large standardized residual.

Expected Mean Squares, using Adjusted SS

Expected Mean Square

Source for Each Term

1 Biounit (3) + 7.5000 (1)

2 Time Point (3) + Q[2]

3 Error (3)

Error Terms for Tests, using Adjusted SS

Synthesis

Source Error DF Error MS of Error MS

1 Biounit 13.00 0.1201 (3)

2 Time Point 13.00 0.1201 (3)

Variance Components, using Adjusted SS

Estimated

Source Value

Biounit -0.01414

Error 0.12011

Grouping Information Using Bonferroni Method and 95.0% Confidence

Time

Point N Mean Grouping

4 3 1.02648 A

5 3 0.62796 A

10 3 0.59500 A

8 3 0.45204 A

7 3 0.42241 A

9 3 0.40278 A

6 3 0.36611 A

3 2 -0.00429 A

Means that do not share a letter are significantly different.

Bonferroni 95.0% Simultaneous Confidence Intervals

Response Variable Cq Prime

All Pairwise Comparisons among Levels of Time Point

Time Point = 3 subtracted from:

Time

Point Lower Center Upper -----+---------+---------+---------+-

4 -0.2244 1.0308 2.286 (----------*---------)

5 -0.6229 0.6322 1.887 (---------*----------)

6 -0.8848 0.3704 1.626 (---------*----------)

7 -0.8285 0.4267 1.682 (----------*---------)

8 -0.7988 0.4563 1.711 (----------*---------)

9 -0.8481 0.4071 1.662 (---------*----------)

10 -0.6559 0.5993 1.854 (---------*---------)

-----+---------+---------+---------+-

-1.2 0.0 1.2 2.4

Time Point = 4 subtracted from:

Time

Point Lower Center Upper -----+---------+---------+---------+-

5 -1.505 -0.3985 0.7084 (---------*--------)

6 -1.767 -0.6604 0.4466 (--------*---------)

7 -1.711 -0.6041 0.5029 (--------*--------)

8 -1.681 -0.5744 0.5325 (--------*--------)

9 -1.731 -0.6237 0.4833 (--------*--------)

10 -1.538 -0.4315 0.6755 (--------*---------)

-----+---------+---------+---------+-

-1.2 0.0 1.2 2.4

Time Point = 5 subtracted from:

Time

Point Lower Center Upper -----+---------+---------+---------+-

6 -1.369 -0.2619 0.8451 (--------*--------)

7 -1.313 -0.2056 0.9014 (--------*---------)

8 -1.283 -0.1759 0.9310 (---------*--------)

9 -1.332 -0.2252 0.8818 (--------*--------)

10 -1.140 -0.0330 1.0740 (--------*--------)

-----+---------+---------+---------+-

-1.2 0.0 1.2 2.4

Time Point = 6 subtracted from:

Time

Point Lower Center Upper -----+---------+---------+---------+-

7 -1.051 0.05630 1.163 (--------*---------)

8 -1.021 0.08593 1.193 (---------*--------)

9 -1.070 0.03667 1.144 (--------*---------)

10 -0.878 0.22889 1.336 (--------*--------)

-----+---------+---------+---------+-

-1.2 0.0 1.2 2.4

Time Point = 7 subtracted from:

Time

Point Lower Center Upper -----+---------+---------+---------+-

8 -1.077 0.02963 1.137 (--------*--------)

9 -1.127 -0.01963 1.087 (--------*--------)

10 -0.934 0.17259 1.280 (--------*---------)

-----+---------+---------+---------+-

-1.2 0.0 1.2 2.4

Time Point = 8 subtracted from:

Time

Point Lower Center Upper -----+---------+---------+---------+-

9 -1.156 -0.04926 1.058 (---------*--------)

10 -0.964 0.14296 1.250 (--------*--------)

-----+---------+---------+---------+-

-1.2 0.0 1.2 2.4

Time Point = 9 subtracted from:

Time

Point Lower Center Upper -----+---------+---------+---------+-

10 -0.9147 0.1922 1.299 (---------*--------)

-----+---------+---------+---------+-

-1.2 0.0 1.2 2.4

Bonferroni Simultaneous Tests

Response Variable Cq Prime

All Pairwise Comparisons among Levels of Time Point

Time Point = 3 subtracted from:

Time Difference SE of Adjusted

Point of Means Difference T-Value P-Value

4 1.0308 0.3209 3.212 0.1905

5 0.6322 0.3209 1.970 1.0000

6 0.3704 0.3209 1.154 1.0000

7 0.4267 0.3209 1.330 1.0000

8 0.4563 0.3209 1.422 1.0000

9 0.4071 0.3209 1.269 1.0000

10 0.5993 0.3209 1.868 1.0000

Time Point = 4 subtracted from:

Time Difference SE of Adjusted

Point of Means Difference T-Value P-Value

5 -0.3985 0.2830 -1.408 1.000

6 -0.6604 0.2830 -2.334 1.000

7 -0.6041 0.2830 -2.135 1.000

8 -0.5744 0.2830 -2.030 1.000

9 -0.6237 0.2830 -2.204 1.000

10 -0.4315 0.2830 -1.525 1.000

Time Point = 5 subtracted from:

Time Difference SE of Adjusted

Point of Means Difference T-Value P-Value

6 -0.2619 0.2830 -0.9254 1.000

7 -0.2056 0.2830 -0.7264 1.000

8 -0.1759 0.2830 -0.6217 1.000

9 -0.2252 0.2830 -0.7958 1.000

10 -0.0330 0.2830 -0.1165 1.000

Time Point = 6 subtracted from:

Time Difference SE of Adjusted

Point of Means Difference T-Value P-Value

7 0.05630 0.2830 0.1989 1.000

8 0.08593 0.2830 0.3037 1.000

9 0.03667 0.2830 0.1296 1.000

10 0.22889 0.2830 0.8089 1.000

Time Point = 7 subtracted from:

Time Difference SE of Adjusted

Point of Means Difference T-Value P-Value

8 0.02963 0.2830 0.10471 1.000

9 -0.01963 0.2830 -0.06937 1.000

10 0.17259 0.2830 0.60992 1.000

Time Point = 8 subtracted from:

Time Difference SE of Adjusted

Point of Means Difference T-Value P-Value

9 -0.04926 0.2830 -0.1741 1.000

10 0.14296 0.2830 0.5052 1.000

Time Point = 9 subtracted from:

Time Difference SE of Adjusted

Point of Means Difference T-Value P-Value

10 0.1922 0.2830 0.6793 1.000

Grouping Information Using Sidak Method and 95.0% Confidence

Time

Point N Mean Grouping

4 3 1.02648 A

5 3 0.62796 A

10 3 0.59500 A

8 3 0.45204 A

7 3 0.42241 A

9 3 0.40278 A

6 3 0.36611 A

3 2 -0.00429 A

Means that do not share a letter are significantly different.

Sidak 95.0% Simultaneous Confidence Intervals

Response Variable Cq Prime

All Pairwise Comparisons among Levels of Time Point

Time Point = 3 subtracted from:

Time

Point Lower Center Upper -----+---------+---------+---------+-

4 -0.2202 1.0308 2.282 (----------*---------)

5 -0.6187 0.6322 1.883 (---------*----------)

6 -0.8806 0.3704 1.621 (---------*----------)

7 -0.8243 0.4267 1.678 (----------*---------)

8 -0.7947 0.4563 1.707 (----------*---------)

9 -0.8439 0.4071 1.658 (---------*----------)

10 -0.6517 0.5993 1.850 (---------*---------)

-----+---------+---------+---------+-

-1.2 0.0 1.2 2.4

Time Point = 4 subtracted from:

Time

Point Lower Center Upper -----+---------+---------+---------+-

5 -1.502 -0.3985 0.7048 (---------*--------)

6 -1.764 -0.6604 0.4429 (--------*---------)

7 -1.707 -0.6041 0.4992 (--------*--------)

8 -1.678 -0.5744 0.5288 (--------*--------)

9 -1.727 -0.6237 0.4796 (--------*--------)

10 -1.535 -0.4315 0.6718 (--------*---------)

-----+---------+---------+---------+-

-1.2 0.0 1.2 2.4

Time Point = 5 subtracted from:

Time

Point Lower Center Upper -----+---------+---------+---------+-

6 -1.365 -0.2619 0.8414 (--------*--------)

7 -1.309 -0.2056 0.8977 (--------*--------)

8 -1.279 -0.1759 0.9273 (---------*--------)

9 -1.328 -0.2252 0.8781 (--------*--------)

10 -1.136 -0.0330 1.0703 (--------*--------)

-----+---------+---------+---------+-

-1.2 0.0 1.2 2.4

Time Point = 6 subtracted from:

Time

Point Lower Center Upper -----+---------+---------+---------+-

7 -1.047 0.05630 1.160 (--------*---------)

8 -1.017 0.08593 1.189 (--------*--------)

9 -1.067 0.03667 1.140 (--------*--------)

10 -0.874 0.22889 1.332 (--------*--------)

-----+---------+---------+---------+-

-1.2 0.0 1.2 2.4

Time Point = 7 subtracted from:

Time

Point Lower Center Upper -----+---------+---------+---------+-

8 -1.074 0.02963 1.133 (--------*--------)

9 -1.123 -0.01963 1.084 (--------*--------)

10 -0.931 0.17259 1.276 (--------*---------)

-----+---------+---------+---------+-

-1.2 0.0 1.2 2.4

Time Point = 8 subtracted from:

Time

Point Lower Center Upper -----+---------+---------+---------+-

9 -1.153 -0.04926 1.054 (---------*--------)

10 -0.960 0.14296 1.246 (--------*--------)

-----+---------+---------+---------+-

-1.2 0.0 1.2 2.4

Time Point = 9 subtracted from:

Time

Point Lower Center Upper -----+---------+---------+---------+-

10 -0.9110 0.1922 1.295 (---------*--------)

-----+---------+---------+---------+-

-1.2 0.0 1.2 2.4

Sidak Simultaneous Tests

Response Variable Cq Prime

All Pairwise Comparisons among Levels of Time Point

Time Point = 3 subtracted from:

Time Difference SE of Adjusted

Point of Means Difference T-Value P-Value

4 1.0308 0.3209 3.212 0.1740

5 0.6322 0.3209 1.970 0.8707

6 0.3704 0.3209 1.154 0.9998

7 0.4267 0.3209 1.330 0.9985

8 0.4563 0.3209 1.422 0.9959

9 0.4071 0.3209 1.269 0.9993

10 0.5993 0.3209 1.868 0.9156

Time Point = 4 subtracted from:

Time Difference SE of Adjusted

Point of Means Difference T-Value P-Value

5 -0.3985 0.2830 -1.408 0.9965

6 -0.6604 0.2830 -2.334 0.6450

7 -0.6041 0.2830 -2.135 0.7784

8 -0.5744 0.2830 -2.030 0.8399

9 -0.6237 0.2830 -2.204 0.7336

10 -0.4315 0.2830 -1.525 0.9899

Time Point = 5 subtracted from:

Time Difference SE of Adjusted

Point of Means Difference T-Value P-Value

6 -0.2619 0.2830 -0.9254 1.000

7 -0.2056 0.2830 -0.7264 1.000

8 -0.1759 0.2830 -0.6217 1.000

9 -0.2252 0.2830 -0.7958 1.000

10 -0.0330 0.2830 -0.1165 1.000

Time Point = 6 subtracted from:

Time Difference SE of Adjusted

Point of Means Difference T-Value P-Value

7 0.05630 0.2830 0.1989 1.000

8 0.08593 0.2830 0.3037 1.000

9 0.03667 0.2830 0.1296 1.000

10 0.22889 0.2830 0.8089 1.000

Time Point = 7 subtracted from:

Time Difference SE of Adjusted

Point of Means Difference T-Value P-Value

8 0.02963 0.2830 0.10471 1.000

9 -0.01963 0.2830 -0.06937 1.000

10 0.17259 0.2830 0.60992 1.000

Time Point = 8 subtracted from:

Time Difference SE of Adjusted

Point of Means Difference T-Value P-Value

9 -0.04926 0.2830 -0.1741 1.000

10 0.14296 0.2830 0.5052 1.000

Time Point = 9 subtracted from:

Time Difference SE of Adjusted

Point of Means Difference T-Value P-Value

10 0.1922 0.2830 0.6793 1.000

Grouping Information Using Tukey Method and 95.0% Confidence

Time

Point N Mean Grouping

4 3 1.02648 A

5 3 0.62796 A

10 3 0.59500 A

8 3 0.45204 A

7 3 0.42241 A

9 3 0.40278 A

6 3 0.36611 A

3 2 -0.00429 A

Means that do not share a letter are significantly different.

Tukey 95.0% Simultaneous Confidence Intervals

Response Variable Cq Prime

All Pairwise Comparisons among Levels of Time Point

Time Point = 3 subtracted from:

Time

Point Lower Center Upper ----+---------+---------+---------+--

4 -0.1150 1.0308 2.177 (---------*--------)

5 -0.5135 0.6322 1.778 (--------*---------)

6 -0.7754 0.3704 1.516 (--------*---------)

7 -0.7191 0.4267 1.572 (---------*--------)

8 -0.6894 0.4563 1.602 (---------*--------)

9 -0.7387 0.4071 1.553 (--------*---------)

10 -0.5465 0.5993 1.745 (---------*---------)

----+---------+---------+---------+--

-1.2 0.0 1.2 2.4

Time Point = 4 subtracted from:

Time

Point Lower Center Upper ----+---------+---------+---------+--

5 -1.409 -0.3985 0.6120 (--------*-------)

6 -1.671 -0.6604 0.3501 (-------*--------)

7 -1.615 -0.6041 0.4064 (-------*-------)

8 -1.585 -0.5744 0.4360 (-------*--------)

9 -1.634 -0.6237 0.3868 (--------*-------)

10 -1.442 -0.4315 0.5790 (-------*--------)

----+---------+---------+---------+--

-1.2 0.0 1.2 2.4

Time Point = 5 subtracted from:

Time

Point Lower Center Upper ----+---------+---------+---------+--

6 -1.272 -0.2619 0.7486 (--------*-------)

7 -1.216 -0.2056 0.8049 (-------*--------)

8 -1.186 -0.1759 0.8345 (--------*-------)

9 -1.236 -0.2252 0.7853 (-------*--------)

10 -1.043 -0.0330 0.9775 (--------*-------)

----+---------+---------+---------+--

-1.2 0.0 1.2 2.4

Time Point = 6 subtracted from:

Time

Point Lower Center Upper ----+---------+---------+---------+--

7 -0.9542 0.05630 1.067 (-------*--------)

8 -0.9245 0.08593 1.096 (--------*-------)

9 -0.9738 0.03667 1.047 (-------*--------)

10 -0.7816 0.22889 1.239 (--------*-------)

----+---------+---------+---------+--

-1.2 0.0 1.2 2.4

Time Point = 7 subtracted from:

Time

Point Lower Center Upper ----+---------+---------+---------+--

8 -0.981 0.02963 1.0401 (-------*--------)

9 -1.030 -0.01963 0.9908 (--------*-------)

10 -0.838 0.17259 1.1831 (-------*--------)

----+---------+---------+---------+--

-1.2 0.0 1.2 2.4

Time Point = 8 subtracted from:

Time

Point Lower Center Upper ----+---------+---------+---------+--

9 -1.060 -0.04926 0.9612 (--------*-------)

10 -0.868 0.14296 1.1534 (-------*--------)

----+---------+---------+---------+--

-1.2 0.0 1.2 2.4

Time Point = 9 subtracted from:

Time

Point Lower Center Upper ----+---------+---------+---------+--

10 -0.8182 0.1922 1.203 (--------*-------)

----+---------+---------+---------+--

-1.2 0.0 1.2 2.4

Tukey Simultaneous Tests

Response Variable Cq Prime

All Pairwise Comparisons among Levels of Time Point

Time Point = 3 subtracted from:

Time Difference SE of Adjusted

Point of Means Difference T-Value P-Value

4 1.0308 0.3209 3.212 0.0912

5 0.6322 0.3209 1.970 0.5328

6 0.3704 0.3209 1.154 0.9316

7 0.4267 0.3209 1.330 0.8720

8 0.4563 0.3209 1.422 0.8323

9 0.4071 0.3209 1.269 0.8952

10 0.5993 0.3209 1.868 0.5916

Time Point = 4 subtracted from:

Time Difference SE of Adjusted

Point of Means Difference T-Value P-Value

5 -0.3985 0.2830 -1.408 0.8386

6 -0.6604 0.2830 -2.334 0.3451

7 -0.6041 0.2830 -2.135 0.4429

8 -0.5744 0.2830 -2.030 0.4995

9 -0.6237 0.2830 -2.204 0.4072

10 -0.4315 0.2830 -1.525 0.7826

Time Point = 5 subtracted from:

Time Difference SE of Adjusted

Point of Means Difference T-Value P-Value

6 -0.2619 0.2830 -0.9254 0.9777

7 -0.2056 0.2830 -0.7264 0.9944

8 -0.1759 0.2830 -0.6217 0.9978

9 -0.2252 0.2830 -0.7958 0.9904

10 -0.0330 0.2830 -0.1165 1.0000

Time Point = 6 subtracted from:

Time Difference SE of Adjusted

Point of Means Difference T-Value P-Value

7 0.05630 0.2830 0.1989 1.0000

8 0.08593 0.2830 0.3037 1.0000

9 0.03667 0.2830 0.1296 1.0000

10 0.22889 0.2830 0.8089 0.9894

Time Point = 7 subtracted from:

Time Difference SE of Adjusted

Point of Means Difference T-Value P-Value

8 0.02963 0.2830 0.10471 1.0000

9 -0.01963 0.2830 -0.06937 1.0000

10 0.17259 0.2830 0.60992 0.9981

Time Point = 8 subtracted from:

Time Difference SE of Adjusted

Point of Means Difference T-Value P-Value

9 -0.04926 0.2830 -0.1741 1.0000

10 0.14296 0.2830 0.5052 0.9994

Time Point = 9 subtracted from:

Time Difference SE of Adjusted

Point of Means Difference T-Value P-Value

10 0.1922 0.2830 0.6793 0.9962

**Residual Plots for Cq Prime**

**Main Effects Plot for Cq Prime**

Psn0918 EX:

**————— 9/11/2012 7:56:24 PM ————————————————————**

**General Linear Model: Cq Prime versus Biounit, Time Point**

Factor Type Levels Values

Biounit random 3 1, 2, 3

Time Point fixed 8 3, 4, 5, 6, 7, 8, 9, 10

Analysis of Variance for Cq Prime, using Adjusted SS for Tests

Source DF Seq SS Adj SS Adj MS F P

Biounit 2 0.5260 0.3479 0.1739 1.15 0.347

Time Point 7 1.6972 1.6972 0.2425 1.60 0.220

Error 13 1.9673 1.9673 0.1513

Total 22 4.1905

S = 0.389010 R-Sq = 53.05% R-Sq(adj) = 20.55%

Term Coef SE Coef T P

Constant 0.63975 0.08219 7.78 0.000

Biounit

1 0.1138 0.1201 0.95 0.360

2 0.0582 0.1143 0.51 0.619

Time Point

3 -0.5828 0.2573 -2.27 0.041

4 0.3940 0.2112 1.87 0.085

5 0.0110 0.2112 0.05 0.959

6 -0.3242 0.2112 -1.54 0.149

7 0.1221 0.2112 0.58 0.573

8 0.0606 0.2112 0.29 0.779

9 0.0469 0.2112 0.22 0.828

Unusual Observations for Cq Prime

Obs Cq Prime Fit SE Fit Residual St Resid

3 0.44222 1.14751 0.25467 -0.70529 -2.40 R

4 1.78667 1.09187 0.25200 0.69480 2.34 R

R denotes an observation with a large standardized residual.

Expected Mean Squares, using Adjusted SS

Expected Mean Square

Source for Each Term

1 Biounit (3) + 7.5000 (1)

2 Time Point (3) + Q[2]

3 Error (3)

Error Terms for Tests, using Adjusted SS

Synthesis

Source Error DF Error MS of Error MS

1 Biounit 13.00 0.1513 (3)

2 Time Point 13.00 0.1513 (3)

Variance Components, using Adjusted SS

Estimated

Source Value

Biounit 0.00301

Error 0.15133

Grouping Information Using Bonferroni Method and 95.0% Confidence

Time

Point N Mean Grouping

4 3 1.03370 A

10 3 0.91222 A

7 3 0.76185 A

8 3 0.70037 A

9 3 0.68667 A

5 3 0.65074 A

6 3 0.31556 A

3 2 0.05690 A

Means that do not share a letter are significantly different.

Bonferroni 95.0% Simultaneous Confidence Intervals

Response Variable Cq Prime

All Pairwise Comparisons among Levels of Time Point

Time Point = 3 subtracted from:

Time

Point Lower Center Upper ------+---------+---------+---------+

4 -0.432 0.9768 2.386 (-----------*-----------)

5 -0.815 0.5938 2.003 (-----------*-----------)

6 -1.150 0.2587 1.668 (-----------*-----------)

7 -0.704 0.7049 2.114 (-----------*-----------)

8 -0.765 0.6435 2.052 (----------*-----------)

9 -0.779 0.6298 2.039 (----------*-----------)

10 -0.554 0.8553 2.264 (-----------*-----------)

------+---------+---------+---------+

-1.2 0.0 1.2 2.4

Time Point = 4 subtracted from:

Time

Point Lower Center Upper ------+---------+---------+---------+

5 -1.625 -0.3830 0.8595 (----------*---------)

6 -1.961 -0.7181 0.5244 (---------*---------)

7 -1.514 -0.2719 0.9707 (----------*---------)

8 -1.576 -0.3333 0.9092 (---------*----------)

9 -1.590 -0.3470 0.8955 (---------*---------)

10 -1.364 -0.1215 1.1210 (---------*---------)

------+---------+---------+---------+

-1.2 0.0 1.2 2.4

Time Point = 5 subtracted from:

Time

Point Lower Center Upper ------+---------+---------+---------+

6 -1.578 -0.3352 0.9073 (---------*----------)

7 -1.131 0.1111 1.3536 (---------*---------)

8 -1.193 0.0496 1.2921 (---------*----------)

9 -1.207 0.0359 1.2784 (---------*----------)

10 -0.981 0.2615 1.5040 (---------*----------)

------+---------+---------+---------+

-1.2 0.0 1.2 2.4

Time Point = 6 subtracted from:

Time

Point Lower Center Upper ------+---------+---------+---------+

7 -0.7962 0.4463 1.689 (----------*---------)

8 -0.8577 0.3848 1.627 (---------*----------)

9 -0.8714 0.3711 1.614 (---------*---------)

10 -0.6458 0.5967 1.839 (---------*---------)

------+---------+---------+---------+

-1.2 0.0 1.2 2.4

Time Point = 7 subtracted from:

Time

Point Lower Center Upper ------+---------+---------+---------+

8 -1.304 -0.06148 1.181 (---------*----------)

9 -1.318 -0.07519 1.167 (---------*----------)

10 -1.092 0.15037 1.393 (---------*----------)

------+---------+---------+---------+

-1.2 0.0 1.2 2.4

Time Point = 8 subtracted from:

Time

Point Lower Center Upper ------+---------+---------+---------+

9 -1.256 -0.01370 1.229 (---------*---------)

10 -1.031 0.21185 1.454 (----------*---------)

------+---------+---------+---------+

-1.2 0.0 1.2 2.4

Time Point = 9 subtracted from:

Time

Point Lower Center Upper ------+---------+---------+---------+

10 -1.017 0.2256 1.468 (---------*---------)

------+---------+---------+---------+

-1.2 0.0 1.2 2.4

Bonferroni Simultaneous Tests

Response Variable Cq Prime

All Pairwise Comparisons among Levels of Time Point

Time Point = 3 subtracted from:

Time Difference SE of Adjusted

Point of Means Difference T-Value P-Value

4 0.9768 0.3602 2.7122 0.4977

5 0.5938 0.3602 1.6488 1.0000

6 0.2587 0.3602 0.7182 1.0000

7 0.7049 0.3602 1.9574 1.0000

8 0.6435 0.3602 1.7866 1.0000

9 0.6298 0.3602 1.7486 1.0000

10 0.8553 0.3602 2.3749 0.9416

Time Point = 4 subtracted from:

Time Difference SE of Adjusted

Point of Means Difference T-Value P-Value

5 -0.3830 0.3176 -1.206 1.000

6 -0.7181 0.3176 -2.261 1.000

7 -0.2719 0.3176 -0.856 1.000

8 -0.3333 0.3176 -1.049 1.000

9 -0.3470 0.3176 -1.093 1.000

10 -0.1215 0.3176 -0.382 1.000

Time Point = 5 subtracted from:

Time Difference SE of Adjusted

Point of Means Difference T-Value P-Value

6 -0.3352 0.3176 -1.055 1.000

7 0.1111 0.3176 0.350 1.000

8 0.0496 0.3176 0.156 1.000

9 0.0359 0.3176 0.113 1.000

10 0.2615 0.3176 0.823 1.000

Time Point = 6 subtracted from:

Time Difference SE of Adjusted

Point of Means Difference T-Value P-Value

7 0.4463 0.3176 1.405 1.000

8 0.3848 0.3176 1.212 1.000

9 0.3711 0.3176 1.168 1.000

10 0.5967 0.3176 1.879 1.000

Time Point = 7 subtracted from:

Time Difference SE of Adjusted

Point of Means Difference T-Value P-Value

8 -0.06148 0.3176 -0.1936 1.000

9 -0.07519 0.3176 -0.2367 1.000

10 0.15037 0.3176 0.4734 1.000

Time Point = 8 subtracted from:

Time Difference SE of Adjusted

Point of Means Difference T-Value P-Value

9 -0.01370 0.3176 -0.04314 1.000

10 0.21185 0.3176 0.66699 1.000

Time Point = 9 subtracted from:

Time Difference SE of Adjusted

Point of Means Difference T-Value P-Value

10 0.2256 0.3176 0.7101 1.000

Grouping Information Using Sidak Method and 95.0% Confidence

Time

Point N Mean Grouping

4 3 1.03370 A

10 3 0.91222 A

7 3 0.76185 A

8 3 0.70037 A

9 3 0.68667 A

5 3 0.65074 A

6 3 0.31556 A

3 2 0.05690 A

Means that do not share a letter are significantly different.

Sidak 95.0% Simultaneous Confidence Intervals

Response Variable Cq Prime

All Pairwise Comparisons among Levels of Time Point

Time Point = 3 subtracted from:

Time

Point Lower Center Upper ------+---------+---------+---------+

4 -0.427 0.9768 2.381 (-----------*-----------)

5 -0.810 0.5938 1.998 (-----------*-----------)

6 -1.146 0.2587 1.663 (-----------*-----------)

7 -0.699 0.7049 2.109 (-----------*-----------)

8 -0.761 0.6435 2.048 (----------*-----------)

9 -0.774 0.6298 2.034 (----------*-----------)

10 -0.549 0.8553 2.259 (-----------*-----------)

------+---------+---------+---------+

-1.2 0.0 1.2 2.4

Time Point = 4 subtracted from:

Time

Point Lower Center Upper ------+---------+---------+---------+

5 -1.621 -0.3830 0.8554 (----------*---------)

6 -1.957 -0.7181 0.5202 (---------*---------)

7 -1.510 -0.2719 0.9665 (----------*---------)

8 -1.572 -0.3333 0.9050 (---------*----------)

9 -1.585 -0.3470 0.8913 (---------*---------)

10 -1.360 -0.1215 1.1169 (---------*---------)

------+---------+---------+---------+

-1.2 0.0 1.2 2.4

Time Point = 5 subtracted from:

Time

Point Lower Center Upper ------+---------+---------+---------+

6 -1.574 -0.3352 0.9032 (---------*----------)

7 -1.127 0.1111 1.3495 (---------*---------)

8 -1.189 0.0496 1.2880 (---------*----------)

9 -1.202 0.0359 1.2743 (---------*----------)

10 -0.977 0.2615 1.4998 (---------*---------)

------+---------+---------+---------+

-1.2 0.0 1.2 2.4

Time Point = 6 subtracted from:

Time

Point Lower Center Upper ------+---------+---------+---------+

7 -0.7921 0.4463 1.685 (----------*---------)

8 -0.8536 0.3848 1.623 (---------*----------)

9 -0.8673 0.3711 1.609 (---------*---------)

10 -0.6417 0.5967 1.835 (---------*---------)

------+---------+---------+---------+

-1.2 0.0 1.2 2.4

Time Point = 7 subtracted from:

Time

Point Lower Center Upper ------+---------+---------+---------+

8 -1.300 -0.06148 1.177 (---------*----------)

9 -1.314 -0.07519 1.163 (---------*----------)

10 -1.088 0.15037 1.389 (---------*----------)

------+---------+---------+---------+

-1.2 0.0 1.2 2.4

Time Point = 8 subtracted from:

Time

Point Lower Center Upper ------+---------+---------+---------+

9 -1.252 -0.01370 1.225 (---------*---------)

10 -1.027 0.21185 1.450 (----------*---------)

------+---------+---------+---------+

-1.2 0.0 1.2 2.4

Time Point = 9 subtracted from:

Time

Point Lower Center Upper ------+---------+---------+---------+

10 -1.013 0.2256 1.464 (---------*---------)

------+---------+---------+---------+

-1.2 0.0 1.2 2.4

Sidak Simultaneous Tests

Response Variable Cq Prime

All Pairwise Comparisons among Levels of Time Point

Time Point = 3 subtracted from:

Time Difference SE of Adjusted

Point of Means Difference T-Value P-Value

4 0.9768 0.3602 2.7122 0.3948

5 0.5938 0.3602 1.6488 0.9747

6 0.2587 0.3602 0.7182 1.0000

7 0.7049 0.3602 1.9574 0.8771

8 0.6435 0.3602 1.7866 0.9431

9 0.6298 0.3602 1.7486 0.9537

10 0.8553 0.3602 2.3749 0.6163

Time Point = 4 subtracted from:

Time Difference SE of Adjusted

Point of Means Difference T-Value P-Value

5 -0.3830 0.3176 -1.206 0.9997

6 -0.7181 0.3176 -2.261 0.6953

7 -0.2719 0.3176 -0.856 1.0000

8 -0.3333 0.3176 -1.049 1.0000

9 -0.3470 0.3176 -1.093 0.9999

10 -0.1215 0.3176 -0.382 1.0000

Time Point = 5 subtracted from:

Time Difference SE of Adjusted

Point of Means Difference T-Value P-Value

6 -0.3352 0.3176 -1.055 1.000

7 0.1111 0.3176 0.350 1.000

8 0.0496 0.3176 0.156 1.000

9 0.0359 0.3176 0.113 1.000

10 0.2615 0.3176 0.823 1.000

Time Point = 6 subtracted from:

Time Difference SE of Adjusted

Point of Means Difference T-Value P-Value

7 0.4463 0.3176 1.405 0.9966

8 0.3848 0.3176 1.212 0.9996

9 0.3711 0.3176 1.168 0.9998

10 0.5967 0.3176 1.879 0.9114

Time Point = 7 subtracted from:

Time Difference SE of Adjusted

Point of Means Difference T-Value P-Value

8 -0.06148 0.3176 -0.1936 1.000

9 -0.07519 0.3176 -0.2367 1.000

10 0.15037 0.3176 0.4734 1.000

Time Point = 8 subtracted from:

Time Difference SE of Adjusted

Point of Means Difference T-Value P-Value

9 -0.01370 0.3176 -0.04314 1.000

10 0.21185 0.3176 0.66699 1.000

Time Point = 9 subtracted from:

Time Difference SE of Adjusted

Point of Means Difference T-Value P-Value

10 0.2256 0.3176 0.7101 1.000

Grouping Information Using Tukey Method and 95.0% Confidence

Time

Point N Mean Grouping

4 3 1.03370 A

10 3 0.91222 A

7 3 0.76185 A

8 3 0.70037 A

9 3 0.68667 A

5 3 0.65074 A

6 3 0.31556 A

3 2 0.05690 A

Means that do not share a letter are significantly different.

Tukey 95.0% Simultaneous Confidence Intervals

Response Variable Cq Prime

All Pairwise Comparisons among Levels of Time Point

Time Point = 3 subtracted from:

Time

Point Lower Center Upper -----+---------+---------+---------+-

4 -0.309 0.9768 2.263 (----------*----------)

5 -0.692 0.5938 1.880 (----------*----------)

6 -1.027 0.2587 1.545 (----------*----------)

7 -0.581 0.7049 1.991 (----------*----------)

8 -0.643 0.6435 1.930 (---------*----------)

9 -0.656 0.6298 1.916 (---------*----------)

10 -0.431 0.8553 2.141 (----------*----------)

-----+---------+---------+---------+-

-1.2 0.0 1.2 2.4

Time Point = 4 subtracted from:

Time

Point Lower Center Upper -----+---------+---------+---------+-

5 -1.517 -0.3830 0.7512 (---------*--------)

6 -1.852 -0.7181 0.4161 (--------*--------)

7 -1.406 -0.2719 0.8624 (---------*--------)

8 -1.468 -0.3333 0.8009 (--------*---------)

9 -1.481 -0.3470 0.7872 (--------*---------)

10 -1.256 -0.1215 1.0127 (--------*--------)

-----+---------+---------+---------+-

-1.2 0.0 1.2 2.4

Time Point = 5 subtracted from:

Time

Point Lower Center Upper -----+---------+---------+---------+-

6 -1.469 -0.3352 0.7990 (--------*---------)

7 -1.023 0.1111 1.2453 (---------*--------)

8 -1.085 0.0496 1.1838 (--------*---------)

9 -1.098 0.0359 1.1701 (--------*---------)

10 -0.873 0.2615 1.3957 (--------*---------)

-----+---------+---------+---------+-

-1.2 0.0 1.2 2.4

Time Point = 6 subtracted from:

Time

Point Lower Center Upper -----+---------+---------+---------+-

7 -0.6879 0.4463 1.581 (---------*--------)

8 -0.7494 0.3848 1.519 (--------*---------)

9 -0.7631 0.3711 1.505 (--------*---------)

10 -0.5375 0.5967 1.731 (--------*--------)

-----+---------+---------+---------+-

-1.2 0.0 1.2 2.4

Time Point = 7 subtracted from:

Time

Point Lower Center Upper -----+---------+---------+---------+-

8 -1.196 -0.06148 1.073 (--------*---------)

9 -1.209 -0.07519 1.059 (--------*---------)

10 -0.984 0.15037 1.285 (--------*---------)

-----+---------+---------+---------+-

-1.2 0.0 1.2 2.4

Time Point = 8 subtracted from:

Time

Point Lower Center Upper -----+---------+---------+---------+-

9 -1.148 -0.01370 1.121 (---------*--------)

10 -0.922 0.21185 1.346 (---------*--------)

-----+---------+---------+---------+-

-1.2 0.0 1.2 2.4

Time Point = 9 subtracted from:

Time

Point Lower Center Upper -----+---------+---------+---------+-

10 -0.9086 0.2256 1.360 (---------*--------)

-----+---------+---------+---------+-

-1.2 0.0 1.2 2.4

Tukey Simultaneous Tests

Response Variable Cq Prime

All Pairwise Comparisons among Levels of Time Point

Time Point = 3 subtracted from:

Time Difference SE of Adjusted

Point of Means Difference T-Value P-Value

4 0.9768 0.3602 2.7122 0.2018

5 0.5938 0.3602 1.6488 0.7165

6 0.2587 0.3602 0.7182 0.9947

7 0.7049 0.3602 1.9574 0.5402

8 0.6435 0.3602 1.7866 0.6383

9 0.6298 0.3602 1.7486 0.6602

10 0.8553 0.3602 2.3749 0.3267

Time Point = 4 subtracted from:

Time Difference SE of Adjusted

Point of Means Difference T-Value P-Value

5 -0.3830 0.3176 -1.206 0.9164

6 -0.7181 0.3176 -2.261 0.3792

7 -0.2719 0.3176 -0.856 0.9854

8 -0.3333 0.3176 -1.049 0.9570

9 -0.3470 0.3176 -1.093 0.9475

10 -0.1215 0.3176 -0.382 0.9999

Time Point = 5 subtracted from:

Time Difference SE of Adjusted

Point of Means Difference T-Value P-Value

6 -0.3352 0.3176 -1.055 0.9558

7 0.1111 0.3176 0.350 0.9999

8 0.0496 0.3176 0.156 1.0000

9 0.0359 0.3176 0.113 1.0000

10 0.2615 0.3176 0.823 0.9883

Time Point = 6 subtracted from:

Time Difference SE of Adjusted

Point of Means Difference T-Value P-Value

7 0.4463 0.3176 1.405 0.8401

8 0.3848 0.3176 1.212 0.9146

9 0.3711 0.3176 1.168 0.9277

10 0.5967 0.3176 1.879 0.5853

Time Point = 7 subtracted from:

Time Difference SE of Adjusted

Point of Means Difference T-Value P-Value

8 -0.06148 0.3176 -0.1936 1.0000

9 -0.07519 0.3176 -0.2367 1.0000

10 0.15037 0.3176 0.4734 0.9996

Time Point = 8 subtracted from:

Time Difference SE of Adjusted

Point of Means Difference T-Value P-Value

9 -0.01370 0.3176 -0.04314 1.0000

10 0.21185 0.3176 0.66699 0.9966

Time Point = 9 subtracted from:

Time Difference SE of Adjusted

Point of Means Difference T-Value P-Value

10 0.2256 0.3176 0.7101 0.9951

**Residual Plots for Cq Prime**

**Main Effects Plot for Cq Prime**

Psn1428:

**————— 9/11/2012 7:56:24 PM ————————————————————**

**General Linear Model: Cq Prime versus Biounit, Time Point**

Factor Type Levels Values

Biounit random 3 1, 2, 3

Time Point fixed 8 3, 4, 5, 6, 7, 8, 9, 10

Analysis of Variance for Cq Prime, using Adjusted SS for Tests

Source DF Seq SS Adj SS Adj MS F P

Biounit 2 0.8632 1.1198 0.5599 1.80 0.204

Time Point 7 39.3871 39.3871 5.6267 18.08 0.000

Error 13 4.0457 4.0457 0.3112

Total 22 44.2959

S = 0.557857 R-Sq = 90.87% R-Sq(adj) = 84.54%

Term Coef SE Coef T P

Constant 3.8104 0.1179 32.33 0.000

Biounit

1 -0.2208 0.1722 -1.28 0.222

2 -0.0845 0.1639 -0.52 0.615

Time Point

3 -3.9208 0.3690 -10.63 0.000

4 -0.3211 0.3028 -1.06 0.308

5 0.4326 0.3028 1.43 0.177

6 0.4863 0.3028 1.61 0.132

7 0.4393 0.3028 1.45 0.171

8 1.0322 0.3028 3.41 0.005

9 1.1641 0.3028 3.84 0.002

Unusual Observations for Cq Prime

Obs Cq Prime Fit SE Fit Residual St Resid

1 -1.24000 -0.19493 0.41839 -1.04507 -2.83 R

2 1.24000 0.19493 0.41839 1.04507 2.83 R

R denotes an observation with a large standardized residual.

Expected Mean Squares, using Adjusted SS

Expected Mean Square

Source for Each Term

1 Biounit (3) + 7.5000 (1)

2 Time Point (3) + Q[2]

3 Error (3)

Error Terms for Tests, using Adjusted SS

Synthesis

Source Error DF Error MS of Error MS

1 Biounit 13.00 0.3112 (3)

2 Time Point 13.00 0.3112 (3)

Variance Components, using Adjusted SS

Estimated

Source Value

Biounit 0.03316

Error 0.31120

Grouping Information Using Bonferroni Method and 95.0% Confidence

Time

Point N Mean Grouping

9 3 4.9744 A

8 3 4.8426 A

10 3 4.4978 A

6 3 4.2967 A

7 3 4.2496 A

5 3 4.2430 A

4 3 3.4893 A

3 2 -0.1104 B

Means that do not share a letter are significantly different.

Bonferroni 95.0% Simultaneous Confidence Intervals

Response Variable Cq Prime

All Pairwise Comparisons among Levels of Time Point

Time Point = 3 subtracted from:

Time

Point Lower Center Upper --------+---------+---------+--------

4 1.579 3.600 5.620 (------*------)

5 2.333 4.353 6.374 (------*-----)

6 2.387 4.407 6.427 (------*-----)

7 2.340 4.360 6.380 (------*-----)

8 2.933 4.953 6.973 (------*-----)

9 3.064 5.085 7.105 (------*------)

10 2.588 4.608 6.629 (-----*------)

--------+---------+---------+--------

0.0 3.0 6.0

Time Point = 4 subtracted from:

Time

Point Lower Center Upper --------+---------+---------+--------

5 -1.028 0.7537 2.536 (-----*----)

6 -0.974 0.8074 2.589 (-----*-----)

7 -1.021 0.7604 2.542 (-----*----)

8 -0.428 1.3533 3.135 (-----*----)

9 -0.297 1.4852 3.267 (-----*-----)

10 -0.773 1.0085 2.790 (-----*-----)

--------+---------+---------+--------

0.0 3.0 6.0

Time Point = 5 subtracted from:

Time

Point Lower Center Upper --------+---------+---------+--------

6 -1.728 0.053704 1.836 (-----*-----)

7 -1.775 0.006667 1.788 (-----*-----)

8 -1.182 0.599630 2.381 (-----*-----)

9 -1.050 0.731481 2.513 (-----*-----)

10 -1.527 0.254815 2.037 (-----*-----)

--------+---------+---------+--------

0.0 3.0 6.0

Time Point = 6 subtracted from:

Time

Point Lower Center Upper --------+---------+---------+--------

7 -1.829 -0.04704 1.735 (-----*-----)

8 -1.236 0.54593 2.328 (-----*-----)

9 -1.104 0.67778 2.460 (-----*-----)

10 -1.581 0.20111 1.983 (-----*-----)

--------+---------+---------+--------

0.0 3.0 6.0

Time Point = 7 subtracted from:

Time

Point Lower Center Upper --------+---------+---------+--------

8 -1.189 0.5930 2.375 (-----*-----)

9 -1.057 0.7248 2.507 (-----*-----)

10 -1.534 0.2481 2.030 (-----*-----)

--------+---------+---------+--------

0.0 3.0 6.0

Time Point = 8 subtracted from:

Time

Point Lower Center Upper --------+---------+---------+--------

9 -1.650 0.1319 1.914 (----*-----)

10 -2.127 -0.3448 1.437 (-----*-----)

--------+---------+---------+--------

0.0 3.0 6.0

Time Point = 9 subtracted from:

Time

Point Lower Center Upper --------+---------+---------+--------

10 -2.258 -0.4767 1.305 (-----*-----)

--------+---------+---------+--------

0.0 3.0 6.0

Bonferroni Simultaneous Tests

Response Variable Cq Prime

All Pairwise Comparisons among Levels of Time Point

Time Point = 3 subtracted from:

Time Difference SE of Adjusted

Point of Means Difference T-Value P-Value

4 3.600 0.5165 6.970 0.0003

5 4.353 0.5165 8.429 0.0000

6 4.407 0.5165 8.533 0.0000

7 4.360 0.5165 8.442 0.0000

8 4.953 0.5165 9.590 0.0000

9 5.085 0.5165 9.845 0.0000

10 4.608 0.5165 8.922 0.0000

Time Point = 4 subtracted from:

Time Difference SE of Adjusted

Point of Means Difference T-Value P-Value

5 0.7537 0.4555 1.655 1.0000

6 0.8074 0.4555 1.773 1.0000

7 0.7604 0.4555 1.669 1.0000

8 1.3533 0.4555 2.971 0.3030

9 1.4852 0.4555 3.261 0.1736

10 1.0085 0.4555 2.214 1.0000

Time Point = 5 subtracted from:

Time Difference SE of Adjusted

Point of Means Difference T-Value P-Value

6 0.053704 0.4555 0.11790 1.000

7 0.006667 0.4555 0.01464 1.000

8 0.599630 0.4555 1.31645 1.000

9 0.731481 0.4555 1.60593 1.000

10 0.254815 0.4555 0.55943 1.000

Time Point = 6 subtracted from:

Time Difference SE of Adjusted

Point of Means Difference T-Value P-Value

7 -0.04704 0.4555 -0.1033 1.000

8 0.54593 0.4555 1.1986 1.000

9 0.67778 0.4555 1.4880 1.000

10 0.20111 0.4555 0.4415 1.000

Time Point = 7 subtracted from:

Time Difference SE of Adjusted

Point of Means Difference T-Value P-Value

8 0.5930 0.4555 1.3018 1.000

9 0.7248 0.4555 1.5913 1.000

10 0.2481 0.4555 0.5448 1.000

Time Point = 8 subtracted from:

Time Difference SE of Adjusted

Point of Means Difference T-Value P-Value

9 0.1319 0.4555 0.2895 1.000

10 -0.3448 0.4555 -0.7570 1.000

Time Point = 9 subtracted from:

Time Difference SE of Adjusted

Point of Means Difference T-Value P-Value

10 -0.4767 0.4555 -1.046 1.000

Grouping Information Using Sidak Method and 95.0% Confidence

Time

Point N Mean Grouping

9 3 4.9744 A

8 3 4.8426 A

10 3 4.4978 A

6 3 4.2967 A

7 3 4.2496 A

5 3 4.2430 A

4 3 3.4893 A

3 2 -0.1104 B

Means that do not share a letter are significantly different.

Sidak 95.0% Simultaneous Confidence Intervals

Response Variable Cq Prime

All Pairwise Comparisons among Levels of Time Point

Time Point = 3 subtracted from:

Time

Point Lower Center Upper --------+---------+---------+--------

4 1.586 3.600 5.613 (------*------)

5 2.340 4.353 6.367 (------*-----)

6 2.393 4.407 6.421 (------*-----)

7 2.346 4.360 6.374 (------*-----)

8 2.939 4.953 6.967 (------*-----)

9 3.071 5.085 7.098 (------*------)

10 2.595 4.608 6.622 (-----*------)

--------+---------+---------+--------

0.0 3.0 6.0

Time Point = 4 subtracted from:

Time

Point Lower Center Upper --------+---------+---------+--------

5 -1.022 0.7537 2.530 (-----*----)

6 -0.968 0.8074 2.583 (-----*-----)

7 -1.016 0.7604 2.536 (-----*----)

8 -0.423 1.3533 3.129 (-----*----)

9 -0.291 1.4852 3.261 (-----*-----)

10 -0.767 1.0085 2.784 (-----*-----)

--------+---------+---------+--------

0.0 3.0 6.0

Time Point = 5 subtracted from:

Time

Point Lower Center Upper --------+---------+---------+--------

6 -1.722 0.053704 1.830 (-----*-----)

7 -1.769 0.006667 1.783 (-----*-----)

8 -1.176 0.599630 2.376 (-----*-----)

9 -1.044 0.731481 2.507 (----*-----)

10 -1.521 0.254815 2.031 (-----*-----)

--------+---------+---------+--------

0.0 3.0 6.0

Time Point = 6 subtracted from:

Time

Point Lower Center Upper --------+---------+---------+--------

7 -1.823 -0.04704 1.729 (-----*-----)

8 -1.230 0.54593 2.322 (-----*-----)

9 -1.098 0.67778 2.454 (-----*-----)

10 -1.575 0.20111 1.977 (-----*-----)

--------+---------+---------+--------

0.0 3.0 6.0

Time Point = 7 subtracted from:

Time

Point Lower Center Upper --------+---------+---------+--------

8 -1.183 0.5930 2.369 (-----*-----)

9 -1.051 0.7248 2.501 (-----*-----)

10 -1.528 0.2481 2.024 (-----*-----)

--------+---------+---------+--------

0.0 3.0 6.0

Time Point = 8 subtracted from:

Time

Point Lower Center Upper --------+---------+---------+--------

9 -1.644 0.1319 1.908 (----*-----)

10 -2.121 -0.3448 1.431 (-----*-----)

--------+---------+---------+--------

0.0 3.0 6.0

Time Point = 9 subtracted from:

Time

Point Lower Center Upper --------+---------+---------+--------

10 -2.253 -0.4767 1.299 (-----*-----)

--------+---------+---------+--------

0.0 3.0 6.0

Sidak Simultaneous Tests

Response Variable Cq Prime

All Pairwise Comparisons among Levels of Time Point

Time Point = 3 subtracted from:

Time Difference SE of Adjusted

Point of Means Difference T-Value P-Value

4 3.600 0.5165 6.970 0.0003

5 4.353 0.5165 8.429 0.0000

6 4.407 0.5165 8.533 0.0000

7 4.360 0.5165 8.442 0.0000

8 4.953 0.5165 9.590 0.0000

9 5.085 0.5165 9.845 0.0000

10 4.608 0.5165 8.922 0.0000

Time Point = 4 subtracted from:

Time Difference SE of Adjusted

Point of Means Difference T-Value P-Value

5 0.7537 0.4555 1.655 0.9738

6 0.8074 0.4555 1.773 0.9472

7 0.7604 0.4555 1.669 0.9711

8 1.3533 0.4555 2.971 0.2626

9 1.4852 0.4555 3.261 0.1598

10 1.0085 0.4555 2.214 0.7270

Time Point = 5 subtracted from:

Time Difference SE of Adjusted

Point of Means Difference T-Value P-Value

6 0.053704 0.4555 0.11790 1.0000

7 0.006667 0.4555 0.01464 1.0000

8 0.599630 0.4555 1.31645 0.9987

9 0.731481 0.4555 1.60593 0.9812

10 0.254815 0.4555 0.55943 1.0000

Time Point = 6 subtracted from:

Time Difference SE of Adjusted

Point of Means Difference T-Value P-Value

7 -0.04704 0.4555 -0.1033 1.0000

8 0.54593 0.4555 1.1986 0.9997

9 0.67778 0.4555 1.4880 0.9926

10 0.20111 0.4555 0.4415 1.0000

Time Point = 7 subtracted from:

Time Difference SE of Adjusted

Point of Means Difference T-Value P-Value

8 0.5930 0.4555 1.3018 0.9989

9 0.7248 0.4555 1.5913 0.9831

10 0.2481 0.4555 0.5448 1.0000

Time Point = 8 subtracted from:

Time Difference SE of Adjusted

Point of Means Difference T-Value P-Value

9 0.1319 0.4555 0.2895 1.000

10 -0.3448 0.4555 -0.7570 1.000

Time Point = 9 subtracted from:

Time Difference SE of Adjusted

Point of Means Difference T-Value P-Value

10 -0.4767 0.4555 -1.046 1.000

Grouping Information Using Tukey Method and 95.0% Confidence

Time

Point N Mean Grouping

9 3 4.9744 A

8 3 4.8426 A

10 3 4.4978 A

6 3 4.2967 A

7 3 4.2496 A

5 3 4.2430 A

4 3 3.4893 A

3 2 -0.1104 B

Means that do not share a letter are significantly different.

Tukey 95.0% Simultaneous Confidence Intervals

Response Variable Cq Prime

All Pairwise Comparisons among Levels of Time Point

Time Point = 3 subtracted from:

Time

Point Lower Center Upper --------+---------+---------+--------

4 1.755 3.600 5.444 (------*-------)

5 2.509 4.353 6.198 (------*-------)

6 2.563 4.407 6.251 (-------*------)

7 2.516 4.360 6.204 (------*-------)

8 3.109 4.953 6.797 (-------*------)

9 3.241 5.085 6.929 (------*-------)

10 2.764 4.608 6.452 (------*-------)

--------+---------+---------+--------

0.0 2.5 5.0

Time Point = 4 subtracted from:

Time

Point Lower Center Upper --------+---------+---------+--------

5 -0.8728 0.7537 2.380 (-----*------)

6 -0.8191 0.8074 2.434 (-----*------)

7 -0.8661 0.7604 2.387 (-----*------)

8 -0.2732 1.3533 2.980 (-----*------)

9 -0.1413 1.4852 3.112 (------*-----)

10 -0.6180 1.0085 2.635 (-----*------)

--------+---------+---------+--------

0.0 2.5 5.0

Time Point = 5 subtracted from:

Time

Point Lower Center Upper --------+---------+---------+--------

6 -1.573 0.053704 1.680 (-----*------)

7 -1.620 0.006667 1.633 (-----*------)

8 -1.027 0.599630 2.226 (-----*------)

9 -0.895 0.731481 2.358 (------*-----)

10 -1.372 0.254815 1.881 (-----*------)

--------+---------+---------+--------

0.0 2.5 5.0

Time Point = 6 subtracted from:

Time

Point Lower Center Upper --------+---------+---------+--------

7 -1.674 -0.04704 1.579 (------*-----)

8 -1.081 0.54593 2.172 (-----*------)

9 -0.949 0.67778 2.304 (------*-----)

10 -1.425 0.20111 1.828 (------*-----)

--------+---------+---------+--------

0.0 2.5 5.0

Time Point = 7 subtracted from:

Time

Point Lower Center Upper --------+---------+---------+--------

8 -1.034 0.5930 2.219 (-----*------)

9 -0.902 0.7248 2.351 (------*-----)

10 -1.378 0.2481 1.875 (------*-----)

--------+---------+---------+--------

0.0 2.5 5.0

Time Point = 8 subtracted from:

Time

Point Lower Center Upper --------+---------+---------+--------

9 -1.495 0.1319 1.758 (------*-----)

10 -1.971 -0.3448 1.282 (------*-----)

--------+---------+---------+--------

0.0 2.5 5.0

Time Point = 9 subtracted from:

Time

Point Lower Center Upper --------+---------+---------+--------

10 -2.103 -0.4767 1.150 (-----*------)

--------+---------+---------+--------

0.0 2.5 5.0

Tukey Simultaneous Tests

Response Variable Cq Prime

All Pairwise Comparisons among Levels of Time Point

Time Point = 3 subtracted from:

Time Difference SE of Adjusted

Point of Means Difference T-Value P-Value

4 3.600 0.5165 6.970 0.0002

5 4.353 0.5165 8.429 0.0000

6 4.407 0.5165 8.533 0.0000

7 4.360 0.5165 8.442 0.0000

8 4.953 0.5165 9.590 0.0000

9 5.085 0.5165 9.845 0.0000

10 4.608 0.5165 8.922 0.0000

Time Point = 4 subtracted from:

Time Difference SE of Adjusted

Point of Means Difference T-Value P-Value

5 0.7537 0.4555 1.655 0.7132

6 0.8074 0.4555 1.773 0.6464

7 0.7604 0.4555 1.669 0.7051

8 1.3533 0.4555 2.971 0.1350

9 1.4852 0.4555 3.261 0.0842

10 1.0085 0.4555 2.214 0.4022

Time Point = 5 subtracted from:

Time Difference SE of Adjusted

Point of Means Difference T-Value P-Value

6 0.053704 0.4555 0.11790 1.0000

7 0.006667 0.4555 0.01464 1.0000

8 0.599630 0.4555 1.31645 0.8773

9 0.731481 0.4555 1.60593 0.7400

10 0.254815 0.4555 0.55943 0.9989

Time Point = 6 subtracted from:

Time Difference SE of Adjusted

Point of Means Difference T-Value P-Value

7 -0.04704 0.4555 -0.1033 1.0000

8 0.54593 0.4555 1.1986 0.9186

9 0.67778 0.4555 1.4880 0.8010

10 0.20111 0.4555 0.4415 0.9998

Time Point = 7 subtracted from:

Time Difference SE of Adjusted

Point of Means Difference T-Value P-Value

8 0.5930 0.4555 1.3018 0.8829

9 0.7248 0.4555 1.5913 0.7478

10 0.2481 0.4555 0.5448 0.9990

Time Point = 8 subtracted from:

Time Difference SE of Adjusted

Point of Means Difference T-Value P-Value

9 0.1319 0.4555 0.2895 1.0000

10 -0.3448 0.4555 -0.7570 0.9928

Time Point = 9 subtracted from:

Time Difference SE of Adjusted

Point of Means Difference T-Value P-Value

10 -0.4767 0.4555 -1.046 0.9576

**Residual Plots for Cq Prime**

**Main Effects Plot for Cq Prime**
